# PhaLP: A Database for the Study of Phage Lytic Proteins and Their Evolution

**DOI:** 10.3390/v13071240

**Published:** 2021-06-26

**Authors:** Bjorn Criel, Steff Taelman, Wim Van Criekinge, Michiel Stock, Yves Briers

**Affiliations:** 1 Laboratory of Applied Biotechnology, Department of Biotechnology, Ghent University, 9000 Gent, Belgium; bjorn.criel@ugent.be; 2 BIOBIX, Department of Data Analysis and Mathematical Modelling, Ghent University, 9000 Gent, Belgium; steff.taelman@ugent.be (S.T.); wim.vancriekinge@ugent.be (W.V.C.); michiel.stock@ugent.be (M.S.); 3 KERMIT, Department of Data Analysis and Mathematical Modelling, Ghent University, 9000 Gent, Belgium

**Keywords:** phage lytic proteins, endolysins, machine learning, biological database, conserved protein domains, protein architectures

## Abstract

Phage lytic proteins are a clinically advanced class of novel enzyme-based antibiotics, so-called enzybiotics. A growing community of researchers develops phage lytic proteins with the perspective of their use as enzybiotics. A successful translation of enzybiotics to the market requires well-considered selections of phage lytic proteins in early research stages. Here, we introduce PhaLP, a database of phage lytic proteins, which serves as an open portal to facilitate the development of phage lytic proteins. PhaLP is a comprehensive, easily accessible and automatically updated database (currently 16,095 entries). Capitalizing on the rich content of PhaLP, we have mapped the high diversity of natural phage lytic proteins and conducted analyses at three levels to gain insight in their host-specific evolution. First, we provide an overview of the modular diversity. Secondly, datamining and interpretable machine learning approaches were adopted to reveal host-specific design rules for domain architectures in endolysins. Lastly, the evolution of phage lytic proteins on the protein sequence level was explored, revealing host-specific clusters. In sum, PhaLP can act as a starting point for the broad community of enzybiotic researchers, while the steadily improving evolutionary insights will serve as a natural inspiration for protein engineers.

## 1. Introduction

Bacteriophages are infective viral particles targeting bacterial cells. During their lytic replication cycle, phages face twice the challenge to cross the bacterial cell wall of their host [1]. Therefore, phages make use of two types of phage lytic proteins: virion-associated lysins (VALs) and endolysins [2]. A VAL is part of the virion and forms a local and small pore in the peptidoglycan layers at the site of infection, while the cell remains intact. The phage genome is ejected through this pore. VALs are mostly a structural part of the virion but can also occur as internal capsid proteins [3,4]. Endolysins are responsible for the massive degradation of the peptidoglycan layer at the end of the lytic cycle. In the canonical phage lysis system, they accumulate in large numbers in the cytoplasm and their release into the periplasmic space is timed by pore-forming holins in the cytoplasmic membrane. The sudden degradation of the peptidoglycan, assisted by the high osmotic pressure inside the cell, causes cell lysis and the concomitant release of newly matured phage particles [5,6].

**Figure 1:**
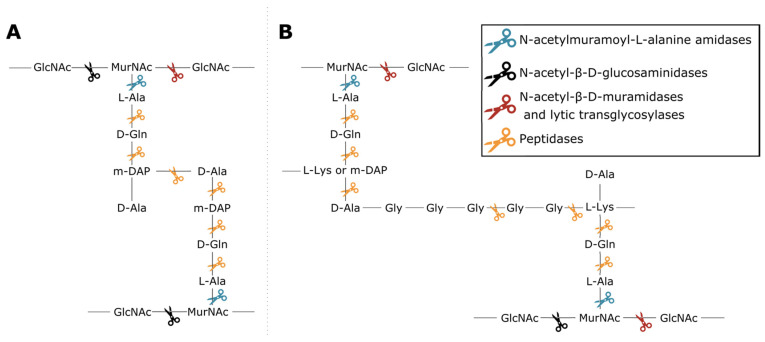
Cleavage sites of the different enzymatic classes of EADs on the primary structures of two common peptidoglycan types. (**A**) peptidoglycan chemotype A1γ is the most common in Gram-negative bacteria; (**B**) peptidoglycan chemotype A3, either α or γ depending on the presence of L-Lys or mDAP in the third position of the peptide subunit, is a type example for Gram-positive bacteria such as *Staphylococcus aureus* [10]. The colored scissors indicate cleavage sites of different classes of EADs. GlcNAc and MurNAc in the glycan strands of each structure refer to N-acetylglucosamine and N-acetylmuramic acid, respectively.

Phage lytic proteins comprise one or more functional domains categorized into two classes: enzymatically active domains (EADs) and cell wall binding domains (CBDs) [7]. Additionally, VALs contain domains with a function other than peptidoglycan degradation such as structural anchoring to the viral particle. At the biochemical level all phage lytic proteins have the same purpose, i.e., peptidoglycan degradation, yet variation in environment and host has led to highly diverse domains and architectures [1,3,4,8]. This variety largely springs from the diversity of peptidoglycan chemotypes among bacterial species, along with the high diversity of secondary cell wall-associated carbohydrate polymers [9,10]. Endolysins have either a globular (one EAD) or modular architecture (multiple domains; at least one EAD and optionally one or more CBDs) [11]. CBDs target the glycan or peptide moieties of peptidoglycan, or specific components of (lipo)teichoic acids, increasing the proximity of EAD to its substrate [12,13]. Five classes of EADs are distinguished based on the bond they cleave in peptidoglycan (Figure 1). (i) N-acetylmuramoyl-L-alanine amidases (EC 3.5.1.28) hydrolyze the amide bond between N-acetylmuramic acid (MurNAc) and L-alanine residues, effectively cleaving the peptide moiety from the glycan strand. (ii) N-acetyl-β-D-glucosaminidases catalyze the hydrolysis of glycosidic β-1,4 linkages between N-acetylglucosamine (GlcNac) and MurNAc. (iii) N-acetyl-β-D-muramidases (EC 3.2.1.17) and (iv) lytic transglycosylases (EC 4.2.2.n2) cleave these linkages between MurNAc and GlcNAc. N-acetyl-β-D-glucosaminidases and N-acetyl-β-D-muramidases are glycosidases (EC 3.2.1.-) that use a hydrolytic mechanism resulting in a terminal reducing GlcNAc or MurNAc residue, respectively. Lytic transglycosylases, on the other hand, use an intramolecular mechanism that creates a 1,6-anhydro bond at the MurNAc residue. Peptidases (EC 3.4.-.-) cleave the bond between two amino acids within the peptidoglycan stem peptide, cross-link or cross-bridge [14]. The specific epitope and chemical bond targeted by the CBD and the EAD, respectively, brings about a well-defined spectrum of activity for phage lytic proteins [15,16]. The modularity of phage lytic proteins, along with the diverse range of domains, has been exploited by protein engineers to modulate the specificity, activity and solubility by domain swapping [11,17].

As early as 1957, it was observed that phage lytic proteins can cause “lysis from without” upon exogenous addition to bacteria [18,19]. It was not until 2001 that their use as enzyme-based antibacterial agents, coined “enzybiotics”, was demonstrated in a murine model against Gram-positive bacteria [20]. The presence of an outer membrane was initially prohibitive for the use of phage lytic proteins as enzybiotics against Gram-negative bacteria, but meanwhile various protein engineering approaches have been developed to overcome this barrier, including the use of outer membrane permeabilizing peptides (Artilysins^®^) [11], bacteriocin domains (lysocins) [21] and phage receptor binding proteins (Innolysins) [22]. Today, enzybiotics are considered the most advanced alternative class of antibacterials under clinical investigation [23]. They offer a necessary response to the alarming threat of antibiotic resistance across global health care systems. Their mode of action is fundamentally different from any existing class of antibiotics. Enzybiotics actively degrade the peptidoglycan component without the need for an active bacterial metabolism, unlike classic antibiotics. This is reflected in a faster cell death and effectiveness against metabolically inactive cells like persisters. In addition, their spectrum is typically narrower (genus, species or strain level) compared to classic antibiotics, causing less harm to beneficial microflora. Finally, the conserved molecular target makes enzybiotics less prone to the inevitable fate of many traditional antibiotics: the emergence and spread of resistance mechanisms [11,24]. A growing community of researchers and companies is therefore investigating their applications, including clinical trials, and engineering their properties to kill a broad diversity of bacteria [8]. Phage lytic proteins have the inherent potential to be developed against any bacterial species, thus representing an unprecedented extensive class of antibiotics with narrow spectrum.

To be successful in the development of phage lytic proteins for such specific applications, it is crucial to make well-considered selections of phage lytic proteins during early research stages. Due to the rich diversity among phage lytic proteins and their annotations, querying databases to retrieve a collection of phage lytic proteins is often cumbersome and requires expert knowledge. Additionally, relevant information is scattered across many specialized databases and often biased by automatic annotation tools. It is therefore challenging to get an overview of this diversity and make proper selections using generic databases. To facilitate this, a dedicated database of phage lytic proteins is needed [16]. Two databases of phage lytic proteins are currently available, i.e., phiBIOTICS [25] and EnzyBase [26]. However, with respectively 21 and 1844 entries, they are still far from covering the diversity available in biological sequence databases. Moreover, their data are collected manually and therefore updating is time intensive.

Here, we introduce PhaLP, a database of currently 16,095 phage lytic proteins. PhaLP is a comprehensive database that is automatically updated with each new UniProt release. PhaLP aims to serve as a portal for researchers to access all relevant information of the current diversity of phage lytic proteins, facilitated by easily accessible search engines. With a focus on protein architecture, evolution, and bacterial hosts of the corresponding phages, we conducted a quantitative analysis of the phage lytic proteins present in the PhaLP database. Datamining approaches reveal host-specific design rules in relation to the protein domain architecture, providing insights in the natural evolution process of phage lytic proteins.

## 2. Materials and Methods

### 2.1. Database Structure and Construction

The PhaLP database runs on MySQL 5.7.32-0ubuntu0.16.04.1 with an InnoDB storage engine. The schema structure of PhaLP is visualized with an enhanced entity-relationship (EER) diagram (Figure S1). It provides users who want to query the database on the MySQL level with a detailed overview of all tables and table relationships. Per table, the corresponding column names, MySQL data types, primary and foreign key information are listed. The process to collect data from primary source databases, process them and store them in PhaLP is automated in Python 3.6.12. The UniProt query (PhaLP.py lines 63–138) is composed of a taxonomic part, to include only sequences of known phage taxons, and a functional part, to filter proteins with a function (gene ontology; GO) or protein domain (InterPro) that indicates a phage lytic protein.

The version number of each PhaLP release corresponds to that of the UniProt version. The analyses described in this manuscript are based on PhaLP v2019_10. This version comprises 11,838 curated protein entries. The latest version of PhaLP is v2021_02 and contains 16,095 protein entries.

### 2.2. Type Classification

First, a training dataset was constructed with examples of both classes, using a semi-manual classification. Based on the presence of specific UniProt annotations, GOs, conserved domain profiles and protein names in the UniProt record itself and all identical proteins, 1604 VALs and 2492 endolysins were manually classified out of 7957 unique protein sequences. The classifying annotations were carefully selected to include as many proteins as possible of each specific class without introducing false positives. 

Next, all 7957 protein sequences were represented by a 1024-dimensional continuous vector, a SeqVec embedding, with the bio_embeddings package (v0.1.3) [27]. A random forest classifier with 100 estimators and balanced weights was trained on the training dataset, using the scikit-learn package (v0.22.2.post1) [28]. First, the performance of this method was estimated in a stratified 10-fold cross-validation, rendering an average F1 score of 0.98433. The “UniProt” table contains the PhaLP type, the evidence source and the predicted probability for the annotated type. For the proteins from the training dataset, the type of the annotation used for classification is specified as evidence source. Their class probability is predicted by the model from the stratified 10-fold cross-validation where the corresponding protein is withheld from the training subset. The type of the remaining 3861 proteins was predicted by a model trained on the complete training dataset. Further elaboration on the methodology is available on GitHub (https://github.com/bjcriel/PhaLP/blob/master/type-classification/PhaLP-type-classification.ipynb; accessed on 25 June 2021)

### 2.3. Accessing Data

The Django web framefork (v2.2.2) was used to build the website (https://www.phalp.org; accessed on 25 June 2021) [29]. BioMart (v0.9) was used to build the advanced user interface [30].

### 2.4. Quantitative Exploration of the Modular Composition

The domains table in PhaLP contains signatures as annotated by InterPro [31]. The latter is a database consortium grouping fourteen member databases, but only four member databases (Pfam, CDD, SMART, PROSITE profiles) were included for the analyses of PhaLP. The remaining ten member databases were omitted because they describe small motifs that are either not actual protein domains, full-length proteins, too specific/broad domain signatures or had no matches in PhaLP. Still, many domain profiles of these four member databases overlap with each other because they are related. To obtain an unambiguous domain architecture for each protein, we have resolved these overlaps, either by joining the overlapping domains in one cluster or by omitting one of the domain profiles. A cluster analysis based on the overlap of each two domains (fraction between overlapping section and largest domain) across all phage lytic proteins was used to manually make domain clusters (File S1). The clustering was done with the “clustermap” function from the seaborn package (v0.11.0) and correlation as distance metric [32]. The resulting clusters were manually curated and adjusted where needed. The resulting domain clusters, together with the absolute number of annotations in PhaLP are described for EADs and CBDs in Table 1 and Table 2, respectively.

### 2.5. Host-Specific Evolution of Phage Lytic Proteins by Recombination

The probability $P(d_{i}|h_{j})$ for all CBD and EAD domain clusters $d_{i}\;\in \left[d_{1}\cdots d_{39}\right]$ (Table 1) given a host genus $h_{j}\;\in \;\left[h_{1}\cdots h_{172}\right]$ was set out and visualized using the seaborn package in Python 3.7.4. The supplementary material also holds analogous figures for all 105 domain clusters, as well as for the probabilities $P(h_{j}|d_{i})$ for both CBDs and EADs as for all domains (Figures S2–S4).

### 2.6. Modular Organization: Architectures and Adjacency

The probability $P(a_{i}|g_{j})$ for all architectures $a_{i}$ given a host Gram-type $g_{j}$ was set out and visualized using the seaborn package in Python 3.7.4. This was done using the 4434 unique endolysin sequences with known architectures consisting out of three domains or less. For this figure, solely endolysins classified with a probability of at least 75% were used. CBD homorepeats were condensed to a single CBD.

Count-based directional adjacency matrices were also generated for these endolysins based on their composition and arrangement of the domains. Visualizations were made using the circlize package (v0.4.12) in R version 3.6.1 [33].

### 2.7. Aggregating Differences in Modular Compositions and Orders into Nature’s Design Rules

Host-specific designs were examined in a two-pronged approach. The first one was based on a machine learning model built to estimate the importance of each domain (and combinations thereof) to a protein’s ability to target a clade of hosts. The second approach used an exhaustive search for each branch to determine the most widely applicable architecture for it. These approaches were selectively applied on a set of 36 bacterial species as this allowed a sample size of at least 25 endolysins per host species. The results were manually combined.

#### 2.7.1. Machine Learning Approach

First, the patterns in domain occurrence and combinations thereof were sought after on each branch. To this end, a decision rule-based classifier implemented in Python 3.7.4 in the SkopeRules package (v1.0.1) was utilized [34]. This model was chosen not only for its predictive power, but also for its high translucency, as it enabled to extract the decisions made by the predictive model and their ensuing precision and recall.

Counts of occurrence of individual domains, as well of duos and trios of adjacent domains were used to construct 466 features for the set of 5052 endolysins for which the host species is annotated. Each feature corresponds to a specific domain or domain combination that is present in the PhaLP database. Depending on the level on which predictions were made, host taxonomy on phylum-, class-, order-, family-, genus- or species-level were used as labels. The resulting set of extracted decision rules projected onto the host phylogenetic tree is available in Figure S5. For the sake of relevancy, only decision rules of both precision and recall above 0.5 were taken into account.

#### 2.7.2. Data Mining Approach

Since the order of domains in endolysins carries non-negligible information, an exhaustive search of all possible architectures was employed to complement the results of the machine learning approach. To this end, regular expressions were set up in Julia 1.3.1 consisting of three blocks (representing adjacent domains) which were each filled by a maximum of three domains. Modifiers were set in place to allow the second and/or third block(s) to be ignored as to accommodate for single or bi-modular architectures. All possible combinations of these blocks, with and without modifiers were tested to match the largest possible selection of architectures (or substructures of architectures) within a subset of endolysins targeting a clade of hosts. The best performing expression, based on an F-score calculated on the precision and recall with which endolysins and the largest possible fraction of their architectures were matched, was extracted.

### 2.8. Vertical Evolution of Endolysins

Through local pairwise sequence alignment of 7957 distinct protein sequences in PhaLP, conservation was more closely examined. The BLOSUM62 substitution matrix was used as it is optimized for average similarities of 20% to 40%. This alignment was performed in BioJulia 1.2. Alignment scores were subsequently extracted, scaled on sequence length and clustered on similarity via the SciPy (v1.3.1) implementation of the UPGMA agglomerative hierarchical clustering algorithm [35]. Visualization was performed in the seaborn package in Python 3.7.4. A detailed overview of the first 44 clusters and the data they envelop is supplied in File S3. The remaining clusters are grouped under cluster 45.

## 3. Results and Discussion

### 3.1. Database Structure and Construction

**Figure 2:**
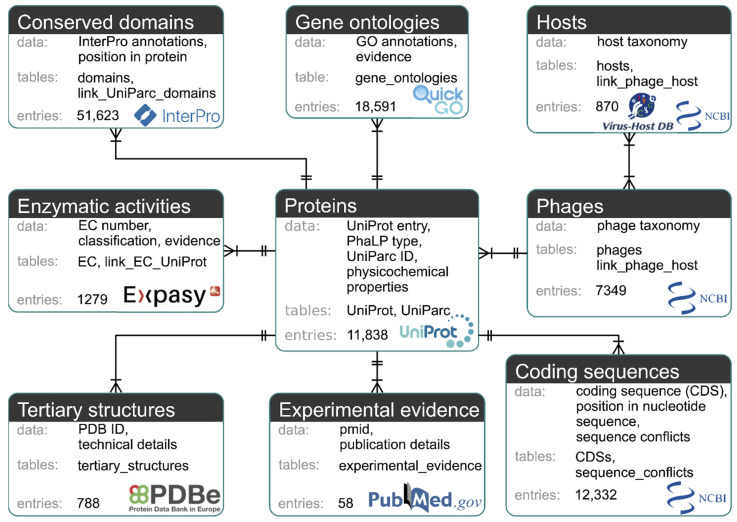
Diagram of the nine data types of PhaLP. Each data type is represented by a box containing a description of the data, the corresponding MySQL table(s), the number of entries in PhaLP v2019_10 and the source database. Relationships between data types are indicated with a crow’s foot notation. A relationship is indicated by a line between two data types with a double perpendicular line at a “one” side and a crow’s foot at a “many” side. The “one-to-many” relationship between for example “phages” and “proteins” can be interpreted as: one phage entry can be linked to multiple protein entries, but each protein entry can only be associated with one phage entry. The “many-to-many” relationship between “hosts” and “phages” can be interpreted as: a phage can have multiple hosts, but a host can also be associated with multiple phages.

The MySQL-based PhaLP database (https://www.phalp.org; accessed on 25 June 2021) integrates nine data types (proteins, phages, hosts, conserved domains, coding sequences (CDSs), GO annotations, enzymatic activities (ECs), tertiary structures, experimental evidence) originating from multiple sources databases (UniProt, UniParc [36], NCBI taxonomy, Virus-Host DB [37], InterPro [31], GenBank, QuickGO, ExPASy ENZYME database, PDB and PubMed). Figure 2 provides an overview of these data types and their mutual relationships.

The protein data form the central hub of PhaLP and describe single phage lytic proteins, corresponding to UniProt entries. UniProt was chosen as primary data source because it provides high-quality, curated and functionally annotated sequence data [36]. To collect a set of phage lytic proteins, UniProt is programmatically queried. The query is carefully constructed to include as much phage lytic proteins as possible without including other proteins. The resulting dataset was manually curated.

Due to increasing sequencing efforts, the amount of available sequence data in biological databases is increasing at an exponential rate [36]. A common problem with many so-called secondary databases is that, while their data sources (primary databases) keep expanding, the authors stop updating the database, resulting in outdated secondary databases. Therefore, the algorithm to gather data from primary databases for PhaLP is automated in a Python script. With every eight-weekly UniProt release, the algorithm is rebooted, adding new entries and updating data that have changed in the source databases. Curation of the new entries remains essential for continuous fine-tuning of the initial query. The latter task can be facilitated by users through the online contact form by reporting new, non-curated entries that are suspected of not being actual phage lytic proteins.

For efficient storage and querying, the nine data types are stored in fourteen tables on the MySQL level (Figure S1). The protein data of single UniProt entries are stored in the “UniProt” table. The protein sequence-related data for each unique protein sequence are stored in the “UniParc” table. These include a stable and unique identifier (UPI) as defined in UniParc, the protein sequence and its predicted physicochemical properties (ProtParam). As such, redundancy is avoided in the database, while conserving the original source information. For each unique sequence, the domain annotations predicted by InterPro are stored in the “link_UniParc_domains” table. The information on each InterPro annotation is stored in the “Domains” table. Although a major part of InterPro annotations are protein domains, other functional annotations such as short motifs, conserved residues, protein (super)families are also included. Info on the phage encoding the phage lytic protein and its host(s) is stored in tables with the “phages” and “hosts” tables, respectively. Info on which phage can infect which host(s) is stored in the “link_phage_host” table. Additional features of phage lytic proteins, including the CDS, experimental evidence published in literature, tertiary structures, enzymatic activity (EC number) and GO terms, are stored in the respective tables: “CDSs”, “experimental_evidence”, “tertiary_structures”, “link_EC_UniProt”, and “gene_ontologies”. Identified sequence conflicts between the protein sequence from UniProt and the translation of the CDS from GenBank are stored in the “sequence_conflicts” table. More detailed info on each EC number is stored in an additional “EC” table, again to reduce data redundancy.

### 3.2. Type Classification

**Figure 3:**
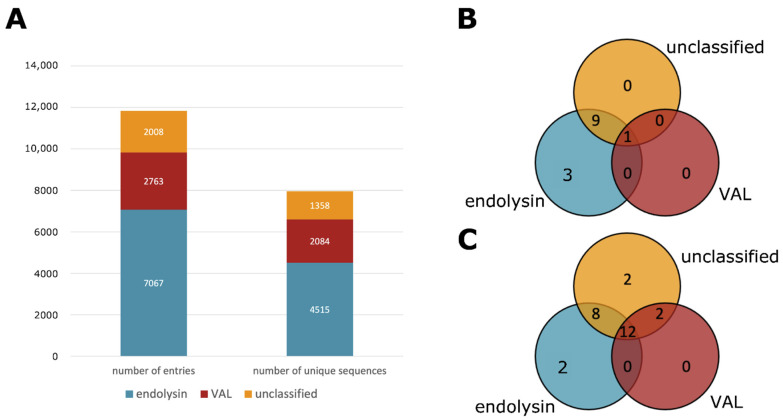
Overview of the types of proteins in PhaLP and the domain clusters observed in them. (**A**) A bar plot of the total number of entries and unique amino acid sequences divided into endolysins and VALs; (**B**) a Venn diagram of the number of CBDs unique to each protein type; (**C**) a Venn diagram of the number of EADs unique to each protein type. The third group (unclassified) denotes proteins that were not classified as either endolysin or VAL with a certainty of at least 75%.

There is a clear discrepancy between VALs and endolysins because of their biological function. However, annotating the correct type of a phage lytic protein is not trivial. Phage lytic proteins whose type is described in literature are relatively low compared to the number of entries in the PhaLP database. It is possible to make an educated guess based on protein annotations, protein length, physicochemical properties, the presence or absence of certain conserved domains or the genomic environment. However, such manual classification implies a time-consuming effort, the outcome would depend on the expert and this approach is not in line with the automated nature of PhaLP. Therefore, a machine learning approach was adopted for this classification task. The random forest classifier is described in more detail in the materials and methods section. Using this classification approach, 8487 endolysins and 3351 VALs were annotated in PhaLP, with 5477 and 2480 unique sequences, respectively (Figure 3A).

Caution should be taken in using the annotated types shown in PhaLP. Although the annotations used to construct the training dataset were carefully selected and originate from one of the highest quality biological databases available (UniProt), the majority of them are electronically assigned. The same is true for the phage lytic proteins classified by the abovementioned predictive model. Therefore, the probability of the predicted type is included in the database. The difficulty in classifying phage lytic proteins is inherent to their rich biological diversity. Only a good overview of this diversity combined with experimental verification can provide the knowledge required to provide a high-quality annotation of phage lytic protein type. Therefore, the remainder of the results concerning the PhaLP type only include proteins with a predicted probability of at least 75%.

### 3.3. Accessing Data

PhaLP can be consulted through two user-friendly web interfaces. The first is a basic searchable and sortable table that displays basic info on each protein entry and the phage encoding the protein (https://www.phalp.org/database; accessed on 25 June 2021). Upon clicking on the UniProt accession number, the user is sent to an overview page with all data linked to that entry. Additionally, the page contains links to the original data sources, as well as two interactive graphical viewers: a representation of the conserved domains on the sequence and a representation of the genomic neighborhood for every CDS linked to the protein.

The second interface is a BioMart that allows the user to customize the selection of attributes that is shown from all tables in the database as well as filter on all these attributes (https://www.phalp.org/biomart; accessed on 25 June 2021). The resulting customized dataset is provided in a tabular format that can be viewed in the interface or downloaded as a “tab-separated values” file. The latter can be loaded into any software of choice to perform further analyses. To allow even more advanced querying, the database is available for download as a MySQL dump file, making it possible to integrate the PhaLP database in customized workflows. Both user interfaces will display the latest version of PhaLP. Older versions will remain available for download as a MySQL dump file.

### 3.4. Quantitative Exploration of the Modular Composition

PhaLP v2019_10 encompasses 11,838 phage lytic proteins with 7957 unique amino acid sequences. The machine learning classifier described above with a cutoff of 75% on the predicted probability, results in 7067 endolysins, 2763 VALs and 2008 unclassified proteins. Each class contains 4515, 2084 and 1358 unique sequences, respectively (Figure 3).

The annotated domain profiles (InterPro) were clustered in 105 custom domain clusters (File S1). The latter are grouped into 26 EAD clusters (Table 1), 13 CBD clusters (Table 2) and 66 miscellaneous domain clusters. They were obtained by grouping similar domain profiles that would otherwise overlap, thereby allowing the unambiguous annotation of modular architectures. These domain clusters will be used in the analyses below and will further be referred to as domains. The InterPro domain annotations described by InterPro and those available in the PhaLP database are further referred to as domain profiles. 

A proportion of 60.7% (*n =* 7188) of the entries comprise a single annotated domain, which is most often an EAD. Phage lytic proteins with multiple annotated domains in their architecture (37.1%, or *n =* 4396) mostly carry both CBDs and EADs. Nonetheless, around 8.8% (*n =* 1046) contain multiple distinct EADs and no annotated CBDs. Multiple distinct EADs may not only augment efficacy of the enzymatic digestion but may also add to the robustness of phage lytic proteins against bacterial resistance as two EADs are presumed to be more resilient to resistance development than one. Even if a bacterial cell gains resistance against one EAD, it will still be susceptible to the other one [38,39]. Two EADs of the same type in a single phage lytic protein have not been described in literature and are neither present in the PhaLP database. The observation of multiple CBDs in a phage lytic protein is rare, occurring only in 4.1% (*n* = 490). Mostly, these are repeats of the same CBD (3.6%; *n* = 429), specifically for CW_1, CW_7, LysM, LGFP, PG_1, SH3 and PSA_CBD. Such repeats can occur up to seven-fold in the case of CW_1. The low occurrence of multiple distinct CBDs (0.5%; *n* = 61) is likely due to their strong influence on the host-specificity of the phage lytic protein. Finally, in 2.2% (*n* = 254) of the phage lytic proteins, no domain is annotated thus far. These phage lytic proteins were identified based on the presence of at least one more general (super)family profile annotation from non-included InterPro member databases like CATH-Gene3D and SUPERFAMILY, for which the selected domain profile databases (Pfam, CDD, SMART, PROSITE profiles) do not contain a domain profile yet, likely due to a too low number of occurrences.

So far, CBDs were only described in endolysins. In VALs, they are considered unnecessary because the structural mechanism of phage infection is responsible for bringing the EAD in close proximity of the peptidoglycan [3,40]. In PhaLP, however, 16 VALs are identified that do contain a LysM domain accompanied by an SLT_related domain. They are described by the following UniProt accession numbers: V9VF10, A0A0A7DMV4, A0A0D3MT00, A0A0E3T6B7, A0A1W6JJ84, A0A1W6MV9, A0A1X9SI30, A0A1W6JJQ3, A0A1W6JKB3, A0A1W6JKP8, A0A1W6JL93, A0A1W6JLT2, A0A1W6JME4, A0A1W6JNB9, A0A3G6JGR4 and E0YJ17. They are all encoded by Siphoviridae phages with *Lactococcus*, *Lactobacillus* or *Bacillus* as a host. Similarly, baseplate proteins with LysM-like structures have been identified in Gram-negative-infecting Myoviridae phages [41]. One possibility is that LysM (or even CBDs in general) may have a receptor-binding role in VALs rather than an assisting role to the EAD, as they have in endolysins. VALs are on average nearly four times as large as endolysins (endolysins have a median length of 250 amino acids compared to 954 amino acids for VALs). While they generally lack a CBD, VALs often comprise structural domains that are part of the virion particle [3]. Due to the large variation in virion morphologies, the domain compositions of VALs are more diverse than those of endolysins.

### 3.5. Host-Specific Evolution of Phage Lytic Proteins by Recombination

**Figure 4:**
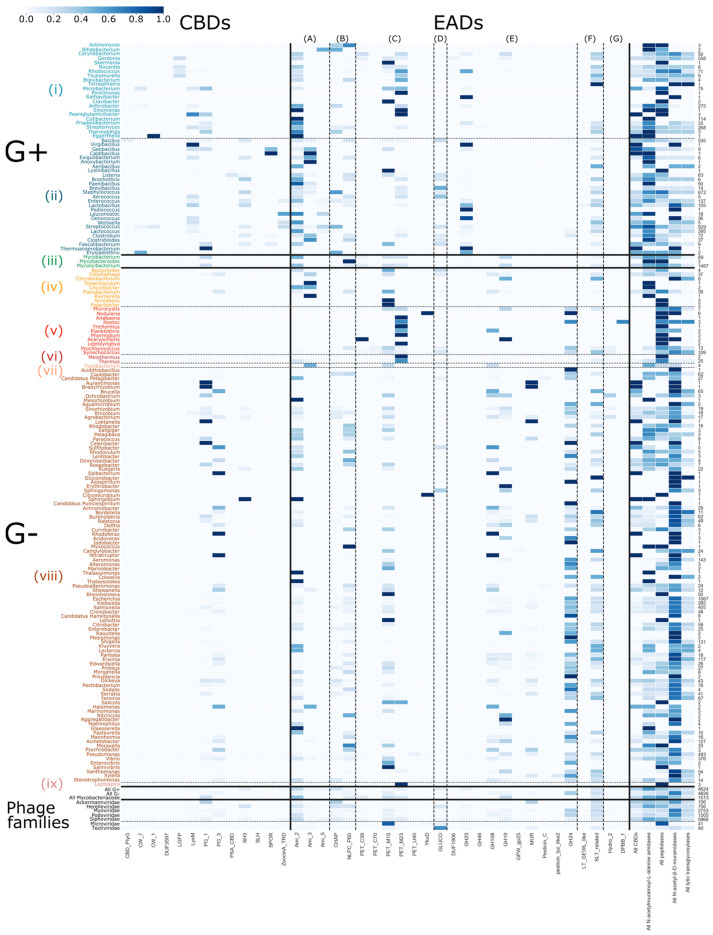
Distribution of EADs and CBDs across bacterial genera. The color bar on the right denotes the probability that a domain occurs in a phage lytic protein, given its bacterial host (dark blue = 1; white = 0). The examined host phyla from top to bottom, separated by dashed and full lines, are: (i) Actinobacteria without Mycobacteriaceae (family), (ii) Firmicutes, (iii) Mycobacteriaceae (family), (iv) Bacteroidetes, (v) Cyanobacteria, (vi) Deinococcus-Thermus, (vii) Fusobacteria, (viii) Proteobacteria and (ix) Spirochaetes. The enzymatic domains from left to right, separated by dashed lines, are: (**A**) N-acetylmuramoyl-L-alanine amidases, (**B**) domains with mixed N-acetylmuramoyl-L-alanine amidases and peptidase activity, (**C**) peptidase domains, (**D**) N-acetyl-β-D-glucosaminidase domains, (**E**) N-acetyl-β-D-muramidase domains, (**F**) domains with N-acetyl-β-D-muramidase and lytic transglycosylase activity and (**G**) lytic transglycosylase domains. On the bottom, probabilities are grouped given the host Gram-types as well as the phage families and on the right, the overall probability of domains of a given activity are set out for each bacterial host. The column of the right shows the number of proteins in PhaLP that target each host genus. Due to the sparsity in occurrence of the miscellaneous domains, only CBDs and EADs were examined in this figure. Figures S2–S4 illustrate the inverse relation, and the distributions including miscellaneous domains, respectively. Due to their size, these figures are best viewed digitally.

Evolution is driven by the accumulation of mutations and recombination of gene fragments. Natural domain recombination driven by horizontal transfer events has played a major role in shaping the existing diversity of phage lytic proteins [11,42]. Analogously, synthetic domain recombination is a proven protein engineering approach to create enzybiotics with increased activity, altered host specificity or other improved properties [11,42,43,44,45]. While plausibly all modular combinations have been tested in nature, those recombination events that resulted in modular combinations with the highest fitness to exert the biological function during phage replication have been retained throughout natural evolution (survival of the fittest). Based on an analysis of the modular composition of all phage lytic proteins in the PhaLP database, we aimed to grasp nature’s design rules that eventually determined the composition of the (currently mapped) diversity of phage lytic proteins. First, we analyzed the presence and absence of the composing domains in relation to the different genera that are targeted by the corresponding phages. PhaLP includes proteins targeting 518 distinct bacterial species across eight different phyla. A number of 4624 entries are encoded by phages that infect Gram-positive hosts and 4826 from phages with a Gram-negative host. The remaining 1510 entries with an annotated host are encoded by phages that infect Mycobacteriaceae. Substantial correlations between the presence of a specific CBD or EAD and the specific genus are observed (Figure 4). For instance, the likelihood of a CBD occurring in a phage lytic protein if the corresponding phage has a Gram-negative host is 7.48%, while for a Gram-positive host it is 32.03%. The current hypothesis for this differential occurrence of CBDs is that a binding domain prevents the uncontrolled diffusion of the endolysin that could otherwise kill nearby potentially new hosts. In Gram-negative bacteria, the outer membrane naturally protects the peptidoglycan layer, eliminating this risk [7]. CBDs of phage lytic proteins from Gram-positive infecting phages also show the highest diversity with 11 out of 13 CBDs present. PG_3 appears to be exclusively present in phage lytic proteins from Gram-negative background. In Figure 4, many genera are linked to a single annotated CBD. In a few cases, this may be due to the limited number of phage lytic proteins reported for this genus (e.g., *Nocardia*, *Brochotrix*), but others (e.g., *Lactococcus*: LysM; *Staphylococcus*: SH3) appear to preferably comprise a specific CBD. Yet, this does not exclude that the latter species also rely on other unknown CBD(s) that remain to be discovered in an unannotated region of the protein, as was recently shown for the CBD of LysSA97, the endolysin of a *S. aureus* phage [46]. Other host genera are associated with two or three CBD types. The *Bacillus* genus corresponds to phage lytic proteins with the highest diversity in CBDs (six out of 13 CBDs occur). Some CBDs are also unique for certain host genera. For example, DUF3597 and PSA_CBD are only present in endolysins of *Bacillus* and *Listeria* phages, respectively. On the contrary, more prevalent CBDs such as SH3 and LysM do convey less specificity, as they bind peptidoglycan, which is a more generic ligand. Modular phage lytic proteins occur less frequently in Gram-negative infecting phages, and only contain nonspecific CBDs, such as PG_3, PG_1, SH3 and LysM. Note that protein entries in PhaLP are typically linked to a single host species, i.e., the species infected by the phage that encodes the respective phage lytic protein. Therefore, this does not exclude that the specific protein can also be active against other host species, as a phage lytic protein can typically have a broader spectrum than its corresponding phage [38]. Indeed, other and additional determinants including the presence of a suitable bacterial receptor and intracellular phage defense mechanisms further constrain the host spectrum of the parental phage compared to the activity spectrum of its phage lytic proteins.

A much higher diversity of EADs is known and annotated. Some domains appear to be nearly ubiquitous in phage lytic proteins across all host genera, such as Ami_2, NPLC_P60, PET_M15 and SLT_related, whereas other EADs are more strictly related to the cell wall structure of either Gram-positives or Gram-negatives. N-acetyl-β-D-muramidase domains GH25 and GH108, for instance, exclusively occur in phage proteins targeting Gram-positive and Gram-negative hosts, respectively. The MUR domain is exclusive to those targeting Proteo- and Cyanobacteria. N-acetyl-β-D-glucosaminidase domains are the rarest but occur in phage lytic proteins associated to both Gram-positive and -negative genera. The Gram-type summary in the lower right-hand corner (Figure 4) indicates that N-acetyl-β-D-muramidases are predominantly associated with Gram-negative genera, whereas Gram-positive-targeting phage lytic proteins contain domains more evenly spread across the domain classes but with N-acetylmuramoyl-L-alanine amidases and peptidases being the most prevalent. In contrast to CBDs, there are no EAD types linked to specific genera, showing that EADs contribute less to specificity compared to CBDs.

### 3.6. Modular Organization: Architectures and Adjacency

**Figure 5:**
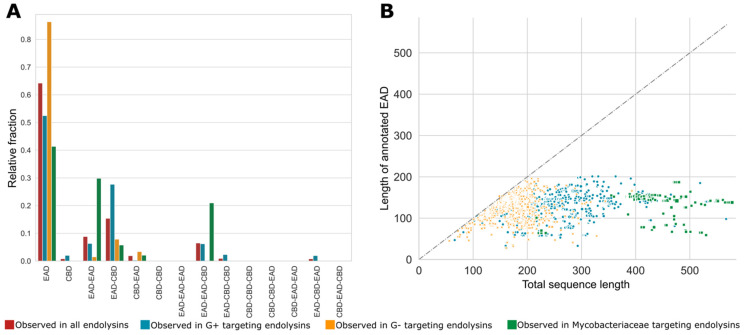
Relative fractions of specific single, bi- and tri-modular architectures for endolysins with different host specificities and the putative presence of unannotated domains. (**A**) The relative occurrence of each of the theoretically possible single, bi- and trimodular architecture comprising EADs and CBDs is set out for all unique endolysin sequences (red) and split up for endolysins from phages infecting Gram-positive bacteria (blue), Gram-negative bacteria (yellow) and Mycobacteriaceae (green). These fractions sum to one for each group of hosts; (**B**) the length of the annotated domain versus the total sequence length of EAD-only architectures. To accommodate for the variable numbers of repeats of a single CBD, they were condensed into a single occurrence of the domain. This analysis shows that mainly endolysins targeting Gram-positive and Mycobacteriaceae hosts comprise unannotated domains.

Natural evolution has not only resulted in the selection of genera-dependent specific domains in the corresponding phage lytic proteins but has also retained specific architectures of CBDs and EADs. As mentioned earlier, the diversity among VALs is higher than among endolysins, often containing at least one of the many miscellaneous domains. Therefore, the following sections will focus solely on architectures of endolysins (151 unique architectures). Figure 5A shows the relative fractions of different architectures with one, two and three annotated domains for all endolysins, and subdivided for endolysins from phages infecting Gram-positive, Gram-negative and Mycobacteriaceae hosts. The single domain architecture is by far the most prominent architecture in the PhaLP database. However, note that these architectures do not take into account potential unannotated domains and thus may reflect a simplification of the true architecture. The majority of the single domain endolysins (1569 out of 2816) have stretches of unannotated sequence of at least 40 amino acids in length, potentially containing one or more unannotated domains. A scatter plot comparing the annotated length and the full length of the endolysins with a single annotated domain indicates that such unannotated regions occur substantially more frequently in endolysins of phages with Gram-positive and Mycobacteriaceae hosts (Figure 5B). On the contrary, endolysins from Gram-negative infecting phages with a single annotated domain are on average smaller and the EAD almost covers the full length in most cases, leaving no space for an unknown domain. We hypothesize that many host-specific CBDs, not covered by the domain profiles in Table 2, remain to be discovered in the C-terminal unannotated region of endolysins from Gram-positive and Mycobacteriaceae infecting phages. In a few cases, such domains have been experimentally verified, but there is no corresponding domain profile available in InterPro, and are thus not covered by our domain clusters [46,47]. Some endolysins apparently only containing a single CBD can also be found (*n* = 30). These possibly include (i) endolysins with a yet unknown or unannotated EAD, (ii) separate CBDs for multimer phage lytic proteins (such as PlyC [48]), (iii) non-functional proteins truncated due to a frameshift or mutations in start or stop codon, or (iv) wrong delineation of the CDS due to sequencing errors.

When a CBD is present in a bi- or trimodular phage endolysin, it is mainly positioned at the C-terminus (EAD-CBD, EAD-EAD-CBD, or EAD-CBD-CBD). In the case of Gram-negatives, for which modular endolysins are scarcer, more entries adopt an EAD-CBD architecture than a CBD-EAD architecture. This contrasts with the preceding assumption that a CBD is most commonly positioned N-terminally in modular endolysins targeting Gram-negatives [11]. A CBD-EAD architecture is nearly inexistent for Gram-positives, an observation corroborated by earlier works [5,24]. In the case of Gram-positive-active endolysins, the CBD can also occupy the middle position (EAD-CBD-CBD, EAD-CBD-EAD). EAD-EAD architectures have mainly been described for Gram-positive and Mycobacteriaceae infecting phages [16]. However, a small group of Gram-negative Cyanobacteria phage endolysins (*n* = 40) display an EAD-EAD architecture as well. The presence of this architecture can be explained by the thicker peptidoglycan of Cyanobacteria compared to other Gram-negatives. For the genus *Synechoccus* in particular, a more extensive cross-linking of the peptidoglycan has been reported as well [49], putatively necessitating the presence of two EADs.

**Figure 6:**
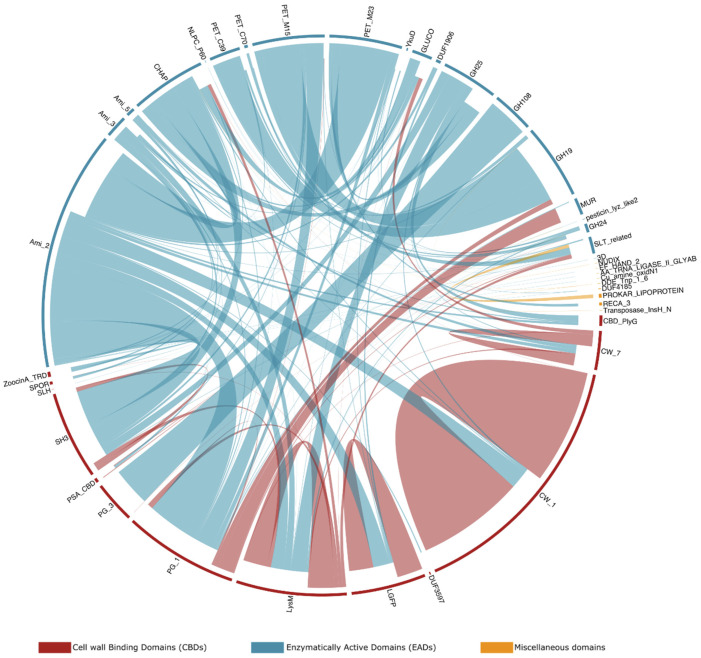
Frequency of occurrence of adjacency of domains in endolysins. Connections are drawn for two domains if they appear consecutively in at least one entry in PhaLP. The frequency of observation of each connection is denoted by thickness. The links carry the color of the domains closest to the N-terminus and stop closer inwards of the domains closest to the C-terminus. Domains are color-coded based on their annotation as EAD, CBD or miscellaneous. Although miscellaneous domains are not expected in endolysins, the few that occur here are suspected to be yet uncharacterized CBDs/EADs or to be erroneously introduced by an incorrect delineation of the CDS in GenBank.

Besides domain architectures, some specific domain combinations are observed more frequently than others. Figure 6 sets out the observed adjacent domains in the endolysins in PhaLP, as well as the frequency of those adjacencies. For example, GH108, an EAD with N-acetyl-β-D-muramidase activity, is in 94.3% (*n* = 216) of its occurrences in endolysins observed N-terminally to a PG_3 domain. In the remaining cases, it is observed as a single domain architecture. Furthermore, while CW_1 appears mostly in repeats (83.0% of its occurrences in endolysins; *n* = 606), it is also often observed (14.1% of its occurrences in endolysins; *n* = 103) C-terminal to Ami_2. Ami_2 is the most ubiquitous EAD among endolysins and occurs N-terminally to *12* out of *13* CBDs (PG_3 being the only one excluded since this domain is only found in combination with GH108). Repeats of domains are fairly uncommon, occurring in only 5.8% (*n* = 401) of endolysins in PhaLP, and are exclusively CBD repeats in endolysins (notably of CW_1, LGFP or LysM domains).

### 3.7. Aggregating Differences in Modular Compositions and Orders into Nature’s Design Rules

The aforementioned selective pressures steering domain composition, domain architecture and domain adjacencies in endolysins have strong ties with the host range of the respective phages. Here, we leverage the size of PhaLP to model the modular composition of endolysins in relation to the phage’s host, through data mining and machine learning approaches. As such, components which are determinant or co-evolutionary signals for the host range can be revealed. These components should not only help to better understand phage-bacteria interaction and co-evolution, but will help set in place guidelines for effective, host-specific engineering of endolysins as enzybiotics. In this analysis, 36 different host species were examined.

**Figure 7:**
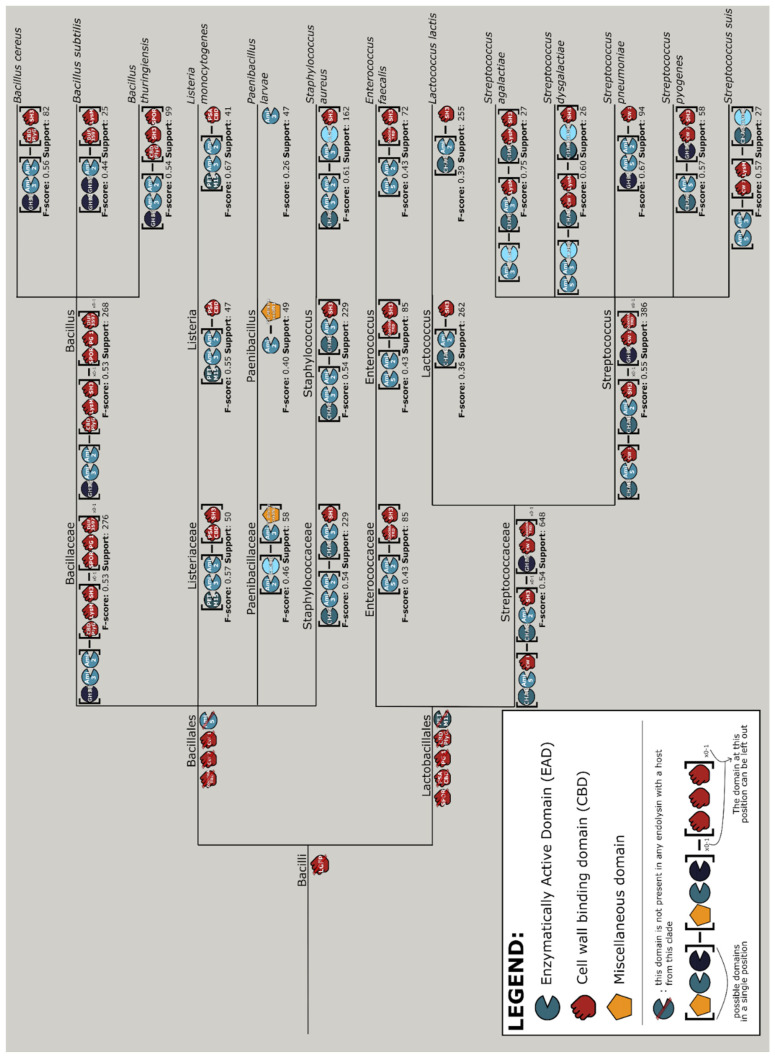
Design tree of phage lytic proteins targeting bacteria from the Bacilli class. Per position, square brackets contain different domains that can occur at that position. To simplify the designs, CBD homorepeats were condensed to a single occurrence of the domain. To accommodate for architectures of one up to three domains, the subscript “x0-1” has been added to indicate domains that either do not occur or occur once. Domains are color-coded based on their annotation as EAD (blue), CBD (red) or miscellaneous (yellow). The figure also provides the F-score as a measure of how many of the actual architectures fit the rule, as well the support, signifying the total amount of proteins corresponding to this branch. Due to its size, this figure is best viewed digitally. An analogous design tree of phage lytic proteins targeting bacteria from all phyla in PhaLP is available in the supplementary material in text-based format (File S2).

The interpretable machine learning approach outputs discriminatory rules for the various taxonomic branches of bacterial hosts (Figure S5). Combining the results of the machine learning and data mining approaches yields a design tree, a guideline of the most prominent endolysin architectures with the corresponding domains for the different clades of hosts. Note the prominence of exclusion rules on the higher clades (i.e., phylum and class), meaning that the machine learning model predicts hosts mostly based on the absence of certain domains rather than their presence, whereas more concrete design rules are specified for the lower clades up until species level. The design tree for the Bacilli class is shown in Figure 7. These design rules were generated through an exhaustive search within the endolysins of phages with a specific clade of host. This was set up to identify the rule to which the highest possible fraction of architectures corresponded. For a good understanding, this also implies that not all endolysins belonging to that specific clade follow the specific design rule. Completely clade-exclusive domains are most noticeably present among the CBDs (File S2). For instance, LGFP for the Corynebacteriales order (likely to target the arabinogalactan covalently linked to their peptidoglycan [50]), SH3 for the Bacilli class and ZoocinA_TRD for the Lactobacillales order. Among *Streptococcus* species, the species-specific CBD, CW_1, appears exclusively in endolysins of phages infecting *Streptococcus pneumoniae*, and most often C-terminally to an Ami_2 domain.

### 3.8. Vertical Evolution of Endolysins

**Figure 8:**
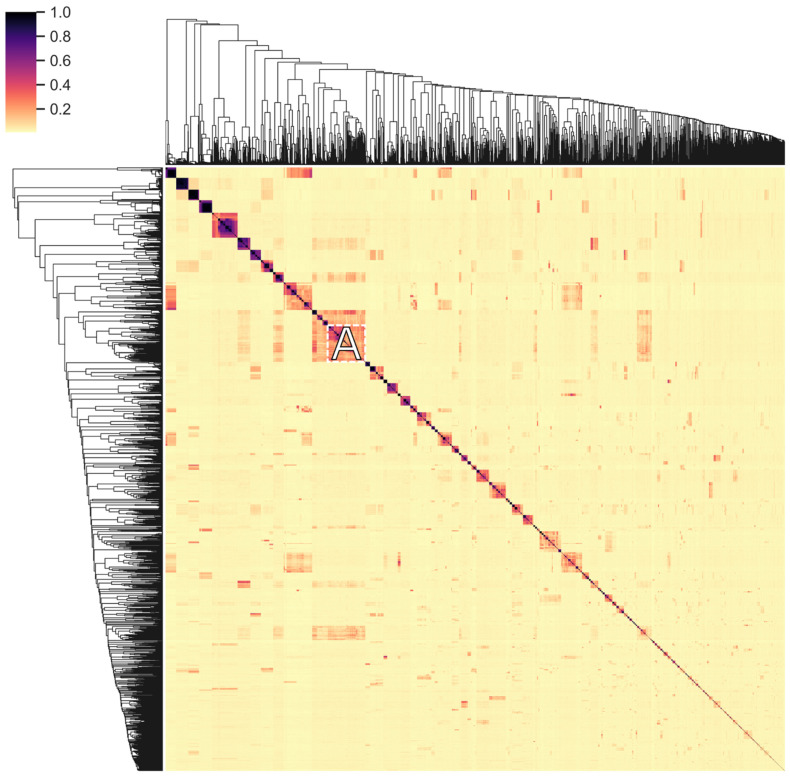
Clustered heatmap of the pairwise protein sequence similarity in endolysins. Every unique endolysin sequence in PhaLP was pairwise aligned against every other endolysin sequence to locate conserved subsequences in the pair. The resulting 4515 × 4515 alignment scores were subsequently normalized on length and clustered to find the most conserved subsequences in the database overall. This figure shows a heatmap of these alignment scores. The cluster discussed in the text is marked by the letter A. The complete set of clustered endolysins with annotation on architecture, accession and host is available in File S3.

The distribution and exchange of conserved domains of endolysins reflects their evolution to fit to their respective environment (horizontal evolution). This diversification is further enhanced by the accumulation of mutations (vertical evolution), especially given the relatively high turnover and mutation rates of phages [51,52], further conveying an increased fitness to the endolysins. Sequence-level conservation in endolysins was examined to gain insight into this vertical evolution process. All unique amino acid sequences of endolysins in PhaLP were clustered on conservation of subsequences (Figure 8 and File S3). These clusters generally contain sequences targeting only one genus or family of hosts, indicating an increased fit for specific sequences in relation to their host or the accumulation of permissive mutations. In general, sequence conservation is less stringent for proteins targeting Gram-negative hosts, resulting in clusters with hosts from different classes of Proteobacteria. Vertical (and horizontal) transfer likely has a lesser impact on host range for these proteins as peptidoglycan in Gram-negative bacteria is rather uniform (A1γ-chemotype) [10], and when a CBD is present, they all target the peptidoglycan itself.

In Figure 8 we note the peculiar cluster A, which contains 243 endolysins targeting Proteobacteria (Gram-negative) and six targeting *Bacillus* species (Gram-positive). The Proteobacteria-targeting proteins all have a single GH24 domain, whose amino acid sequence is identical to the same domain in the GH24-LysM-LysM architecture of the proteins targeting *Bacillus*. The latter shares the A1γ-chemotype with Gram-negatives. These observations suggest a recent horizontal transfer of either the gene fragment encoding the GH24 domain or that encoding the LysM domains, thereby enabling the endolysin to switch host specificity and corresponding Gram-type.

## 4. Conclusions

While resistance against conventional antibiotics is at an all-time high, research into new antimicrobial is sparse, and often niche in application [23]. To facilitate large-scale analyses into one of the most promising novel antimicrobials, we present PhaLP: a database of phage lytic proteins. With PhaLP, we aim to provide a well-considered selection of phage lytic proteins as a starting point for a successful translation of enzybiotics to the market. PhaLP provides a central data hub to access, explore and collect data relevant for one’s own research interests. A BioMart user interface allows users to customize and filter the data to obtain a custom dataset to conduct bioinformatic or computational analyses. 

While automation of curation and incomplete or inconsistent annotation from source databases remain stumbling blocks—some of which have been tackled in this work—the analyses conducted here already showcase the potential of PhaLP. Our initial analyses provide new insights in the modular diversity of phage lytic proteins and might guide the development of host-specific enzybiotics. However, we have only scratched the surface and, depending on one’s research interests, the possible findings in PhaLP are endless.

## Figures and Tables

**Table 1:** EAD clusters observed in phage lytic proteins and their related domain accessions from various protein databases, grouped per enzymatic activity.

Enzymatic Activity ^a^	Domain Cluster Name ^b^	Occurrences in PhaLP ^c^	Domain Accessions (Occurrences in PhaLP) ^d^
(A)	Ami_2	2309	SM00644 (2078), SM00701 (302), cd06583 (1923), PF01510 (2291)
	Ami_3	294	SM00646 (269), cd02696 (290), PF01520 (294)
	Ami_5	314	PF05382 (314)
(B)	CHAP	1127	PF05257 (955), PS50911 (958)
	NLPC_P60	747	PF00877 (747)
(C)	PET_M15	1081	cd14814 (103), cd14852 (2), PF02557 (55), cd14849 (1), cd14844 (210), PF08291 (248), cd14845 (632), PF13539 (724)
	PET_M23	782	PF01551 (782)
	PET_C39	260	PF13529 (260)
	PET_U40	38	PF10464 (38)
	PET_C70	19	PF12385 (19)
	YkuD	10	PF10908 (1), cd16913 (10), PF03734 (4)
(D)	GLUCO	541	PF01832 (541), SM00047 (504)
(E)	GH24	1834	cd00737 (466), cd00736 (181), cd16900 (424), cd16901 (121), PF00959 (1546), cd00735 (642)
	GH19	748	cd00325 (743), PF00182 (283)
	GH25	388	cd00599 (90), cd06417 (1), cd06415 (83), cd06414 (4), cd06525 (3), PF01183 (388), SM00641 (304), cd06522 (1), cd06523 (32)
	GH108	254	cd13926 (254), PF05838 (250)
	MUR	162	PF11860 (162)
	DUF1906	30	PF08924 (30)
	GH46	10	cd00978 (10), PF01374 (10)
	Pesticin_C	6	cd16902 (6), PF16754 (6)
	GPW_gp25	1	PF04965 (1)
	Pesticin_lyz_like2	1	cd16904 (1)
(F)	SLT_related	2202	PS51348 (1), cd16899 (16), PF00062 (1), cd13399 (21), cd16896 (17), PF01464 (1138), cd00254 (517), cd13401 (52), cd01021 (33), cd16893 (21), cd16894 (101), cd13400 (94), cd13403 (79), cd13925 (273), PF06737 (203), cd13402 (529), PF18013 (329)
	LT_GEWL_like	24	cd16891 (24), PF13702 (24)
(G)	Hydro_2	32	PF07486 (32)
	DPBB_1	2	PF03330 (2)

^a^ The enzymatic activity, ^b^ the cluster name, ^c^ the number of occurrences in PhaLP v2019_10, ^d^ the accession numbers of the associated domain profiles with the number of occurrences in PhaLP v2019_10 between brackets. (A) N-acetylmuramoyl-L-alanine amidase, (B) N-acetylmuramoyl-L-alanine amidase and peptidase, (C) peptidase, (D) N-acetyl-β-D-glucosaminidase, (E) N-acetyl-β-D-muramidase, (F) N-acetyl-β-D-muramidase, lytic transglycosylase, (G) lytic transglycosylase. Domain accessions have a hyperlink to the InterPro page of the respective domain profile in digital versions.

**Table 2:** CBD clusters observed in phage lytic proteins and their related domain accessions from various protein databases.

Domain Cluster Name ^a^	Occurrences in PhaLP ^b^	Domain Accessions (Occurrences in PhaLP) ^c^
CBD_PlyG	54	PF12123 (54)
CW_7	186	PF08230 (186), SM01095 (186)
CW_1	757	PF01473 (628), PS51170 (757)
DUF3597	8	PF12200 (8)
LGFP	276	PF08310 (276)
LysM	427	PF01476 (422), cd00118 (425), PS51782 (425), SM00257 (415)
PG_1	640	PF01471 (640)
PG_3	235	PF09374 (235)
PSA_CBD	18	PF18341 (18)
SH3	552	PF08239 (34), PF08460 (462), PS51781 (179), SM00287 (414)
SLH	2	PS51272 (1), PF00395 (1)
SPOR	22	PF05036 (21), PS51724 (17)
ZoocinA_TRD	250	PF16775 (250)

^a^ The cluster name, ^b^ the number of occurrences in PhaLP v2019_10, ^c^ the accession numbers of the associated domain profiles with the number of occurrences in PhaLP v2019_10 between brackets. Domain accessions have a hyperlink to the InterPro page of the respective domain profile in digital versions.

## Data Availability

The data underlying this article (PhaLP v2019_10) are available as a MySQL dump file from https://www.phalp.org/static/PhaLP/dumps/PhaLP_dump_v2019_10.sql; accessed on 24 June 2021. The code and additional files used to construct the database and perform the analyses is available from https://github.com/bjcriel/PhaLP; accessed on 24 June 2021. The files are sorted in folders per section.

## Supplementary Materials

The following are available online at https://www.mdpi.com/article/10.3390/v13071240/s1, Figure S1: Enhanced entity-relationship (EER) diagram of the MySQL-based PhaLP database, Figure S2: Distribution of bacterial genera across EADs and CBDs, Figure S3: Distribution of all domains across bacterial genera, Figure S4: Distribution of bacterial genera across all domains, Figure S5: Decision rules for phage lytic proteins targeting bacteria from all clades that have more than 25 related proteins in PhaLP as predicted by SkopeRules, File S1: Pairwise comparison of overlapping domain profiles, File S2: Design tree for phage lytic proteins targeting bacteria from all clades that have more than 25 related proteins in PhaLP; File S3: Cluster analysis of the pairwise protein sequence similarity between endolysins.
