# Supplementary material for: PhaLP: A Database for the Study of Phage Lytic Proteins and Their Evolution
Source: Viruses. 2021 Jun 26;13(7):1240. doi: 10.3390/v13071240 (PMC8310338; doi:10.3390/v13071240)
Supplement: Supplementary file 1 [file viruses-13-01240-s001.zip › S1 File.pdf]

# of total hits	accession	custom cluster
1	cd17926	Helicase_ATP-bd
1	cd18012	Helicase_ATP-bd
2	PF00176	Helicase_ATP-bd
10	SM00487	Helicase_ATP-bd
4	cd18011	Helicase_ATP-bd
7	PS51192	Helicase_ATP-bd
1	cd17919	Helicase_ATP-bd
2	PF04851	Helicase_ATP-bd
330	PF01476	LysM
332	cd00118	LysM
333	PS51782	LysM
327	SM00257	LysM
46	cd00599	GH25
1	cd06417	GH25
61	cd06415	GH25
4	cd06414	GH25
3	cd06525	GH25
275	PF01183	GH25
207	SM00641	GH25
1	cd06522	GH25
24	cd06523	GH25
20	PF05036	SPOR
16	PS51724	SPOR
194	SM00646	Ami_3
209	cd02696	Ami_3
213	PF01520	Ami_3
4	PS50222	EF_HAND_2
1239	SM00644	Ami_2
202	SM00701	Ami_2
1173	cd06583	Ami_2
1407	PF01510	Ami_2
1	PF00565	SNase
1	PS50830	SNase
1	SM00318	SNase
1	cd02883	NUDIX
1	PF00293	NUDIX
1	PS51462	NUDIX
1	PF10908	YkuD
7	cd16913	YkuD
4	PF03734	YkuD

7	PS51194	Helicase_C
8	cd18793	Helicase_C
4	SM00490	Helicase_C
1	PS51348	SLT-related
12	cd16899	SLT-related
1	PF00062	SLT-related
14	cd13399	SLT-related
14	cd16896	SLT-related
837	PF01464	SLT-related
334	cd00254	SLT-related
46	cd13401	SLT-related
24	cd01021	SLT-related
15	cd16893	SLT-related
91	cd16894	SLT-related
50	cd13400	SLT-related
71	cd13403	SLT-related
182	cd13925	SLT-related
152	PF06737	SLT-related
462	cd13402	SLT-related
232	PF18013	SLT-related
94	cd14814	Peptidase_M15
2	cd14852	Peptidase_M15
47	PF02557	Peptidase_M15
1	cd14849	Peptidase_M15
131	cd14844	Peptidase_M15
156	PF08291	Peptidase_M15
228	PF06714	Gp5_OB_N
179	cd13926	GH108
172	PF05838	GH108
349	cd00737	Phage_lysozyme
1	cd16904	pesticin_lyz_like2
130	cd00736	Phage_lysozyme
282	cd16900	Phage_lysozyme
73	cd16901	Phage_lysozyme
1003	PF00959	Phage_lysozyme
422	cd00735	Phage_lysozyme
3	PF07669	MTases
3	cd02440	MTases
2	PF05175	MTases
21	cd16891	LT_GEWL_like
21	PF13702	LT_GEWL_like
42	cd00229	SGNH_hydro
42	PF13472	SGNH_hydro
2	PF02368	Big_2

4	SM00635	Big_2
136	PF08230	CPL7
136	SM01095	CPL7
7	cd00978	GH46
7	PF01374	GH46
6	cd16902	Pesticin_C
6	PF16754	Pesticin_C
473	cd00325	GH19
202	PF00182	GH19
380	cd14845	Peptidase_M15
428	PF13539	Peptidase_M15
14	cd14667	3D
7	PF06725	3D
1	cd14688	bZIP_YAP
31	PF13884	S74_ICA
43	PS51688	S74_ICA
70	cd06503	ATP-synt_Fo_b
2	cd18586	ABC_6TM_PrtD_like
1	cd18577	ABC_6TM_Pgp_ABCB1_D1_like
1	cd18028	Helicase_ATP-bd
1	cd17504	MFS_MMR_MDR_like
3	cd17477	MFS_YcaD_like
1	cd17330	MFS_SLC46_TetA_like
1	cd17324	MFS_NepI_like
2	cd17321	MFS_MMR_MDR_like
1	cd17040	Ubl_MoaD_like
7	cd17320	MFS_MdfA_MDR_like
12	cd14830	Delta_COP_N
1	cd08173	Gro1PDH
2	cd06180	MFS_YjiJ
3	cd06174	MFS
3	cd06173	MFS_MefA_like
1	cd04631	CBS_archAMPK_gamma-repeat[illegible]
1	cd02775	MopB_CT
8	cd00890	Prefoldin
1	cd00063	FN3
1	cd00442	Lyz_like
1	PS51272	SLH
1	PS50861	AA_TRNA_LIGASE_II_GLYAB
1	PS51186	GNAT
4	PS50853	FN3
1	PS50817	INTEIN_N_TER
8	PS50163	RECA_3
7	PF18858	LPD39

5	PF18857	LPD38
2	PF18853	LPD37
3	PF18823	InPase
2	PF18809	PBECR1
1	PF18760	ART-PolyVal
5	PF18798	LPD3
17	PF18341	PSA_CBD
1	PF17969	Ldt_C
3	PF16838	Caud_tail_N
186	PF16775	ZoocinA_TRD
8	PF15711	ILEI
3	PF13810	DUF4185
1	PF13751	DDE_Tnp_1_6
1	PF13550	Phage-tail_3
1	PF13385	Laminin_G_3
156	PF13529	Peptidase_C39_2
1	PF13365	Trypsin_2
1	PF13354	Beta-lactamase2
1	PF13199	Glyco_hydro_66
19	PF12385	Peptidase_C70
6	PF12200	DUF3597
31	PF12123	Amidase02_C
90	PF11860	Muramidase
2	PF11650	Tail_accessory_Gp4
214	PF10145	Phage_tape_meas
37	PF10464	Peptidase_U40
2	PF09718	Phage_lambda_GpH_tape_mea
6	PF09588	YqaJ
157	PF09374	PG_3
23	PF08924	DUF1906
1	PF08481	GBS_Bsp-like
220	PF08310	LGFP
31	PF07902	Gp58
1	PF07833	Cu_amine_oxidN1
1	PF06832	BiPBP_C
32	PF07486	Hydrolase_2
1	PF02384	N6_Mtase
2	PF02018	CBM_4_9
1	PF01664	Reo_sigma1
339	PF01471	PG_1
36	PF01391	Collagen
1	PF01129	ART
1	PF00912	Transgly
1	PF00905	Transpeptidase

1	PF00395	SLH
1	PF00753	Lactamase_B
6	PF06791	TMP_2
230	PF06715	Gp5_C
1	PF05598	Transposase_InsH_N
4	PF05435	DNA_terminal_Gp3
231	PF05382	Ami_5
1	PF05203	Hom_end_hint
1	PF05106	Phage_lambda_GpS_holin
1	PF04965	IraD/Gp25-like
2	PF03330	RlpA-like_DPBB
1	PF04466	Terminase_L_N
84	PS50249	MPN
77	SM00232	MPN
185	cd08073	MPN
183	PF14464	MPN
469	PF00877	NLPC_P60
499	PF01551	Peptidase_M23
548	PF01473	CW
662	PS51170	CW
23	PS51257	PROKAR_LIPOPROTEIN
403	PF01832	Glucosaminidase
371	SM00047	Glucosaminidase
32	PF08239	SH3
321	PF08460	SH3
155	PS51781	SH3
290	SM00287	SH3
634	PF05257	CHAP
657	PS50911	CHAP

**accession - [InterPro name - source db name]**	average fraction of the overl							
	cd17926	cd18012	PF00176	SM00487	cd18011	PS51192	cd17919	PF04851
cd17926- [DEXH-box helicase domain of DEAD-like helicase restrict	1			0		0		
cd18012- [DEAQ-box helicase domain of archaeal and bacterial SNF		1	0	0				
PF00176- [SNF2-related, N-terminal domain (SNF2_N) - SNF2 family		0	1	1	1	1		
SM00487- [Helicase superfamily 1/2, ATP-binding domain (Helicase	0	0	1	1	1	1	0	1
cd18011- [DEXH-box helicase domain of RapA (DEXDc_RapA)]			1	1	1	1		1
PS51192- [Helicase superfamily 1/2, ATP-binding domain (Helicase_	0		1	1	1	1	0	1
cd17919- [DEXH/Q-box helicase domain of DEAD-like helicase Snf f				0		0	1	1
PF04851- [Helicase/UvrB, N-terminal (Helicase/UvrB_N) - Type III re				1	1	1	1	1
PF01476- [LysM domain (LysM_dom) - LysM domain (LysM)]								
cd00118- [LysM domain (LysM_dom) - Lysin Motif is a small domai								
PS51782- [LysM domain (LysM_dom) - LysM domain profile (LYSM)								
SM00257- [LysM domain (LysM_dom) - Lysin motif (LysM)]								
cd00599- [Endo-N-acetylmuramidases (muramidases) are lysozyme								
cd06417- [LysA is a cell wall endolysin produced by Lactobacillus fe								
cd06415- [Cpl-1 lysin (also known as Cpl-9 lysozyme / muramidase)								
cd06414- [The LytC lysozyme of Streptococcus pneumoniae is a bac								
cd06525- [Lyc muramidase is an autolytic lysozyme (autolysin) from								
PF01183- [Glycoside hydrolase, family 25 (Glyco_hydro_25) - Glyco								
SM00641- [Glycoside hydrolase, family 25 subgroup (Glyco_hydro_								
cd06522- [AtlA is an autolysin found in Gram-positive lactic acid ba								
cd06523- [PlyB is a bacteriophage endolysin that displays potent ly								
PF05036- [Sporulation-like domain (SPOR-like_dom) - Sporulation r								
PS51724- [Sporulation-like domain (SPOR-like_dom) - SPOR domain								
SM00646- [N-acetylmuramoyl-L-alanine amidase, catalytic domain								
cd02696- [N-acetylmuramoyl-L-alanine amidase, catalytic domain (								
PF01520- [N-acetylmuramoyl-L-alanine amidase, catalytic domain (								
PS50222- [EF-hand domain (EF_hand_dom) - EF-hand calcium-bind								
SM00644- [N-acetylmuramoyl-L-alanine amidase domain (Amidase								
SM00701- [Peptidoglycan recognition protein family domain, meta								
cd06583- [N-acetylmuramoyl-L-alanine amidase domain (Amidase_								
PF01510- [N-acetylmuramoyl-L-alanine amidase domain (Amidase_								
PF00565- [Staphylococcal nuclease (SNase-like), OB-fold (Staphyloc								
PS50830- [Staphylococcal nuclease (SNase-like), OB-fold (Staphyloc								
SM00318- [Staphylococcal nuclease (SNase-like), OB-fold (Staphylo								
cd02883- [Nudix hydrolase is a superfamily of enzymes found in all								
PF00293- [NUDIX hydrolase domain (NUDIX_hydrolase_dom) - NUD								
PS51462- [NUDIX hydrolase domain (NUDIX_hydrolase_dom) - Nuc								
PF10908- [Protein of unknown function DUF2778 (DUF2778) - Prot								
cd16913- [L,D-transpeptidases/carboxypeptidases similar to Bacillu								
PF03734- [L,D-transpeptidase catalytic domain (LD_TPept_cat_dom								

PS51194- [Helicase, C-terminal (Helicase_C) - Superfamilies 1 and 2
cd18793- [C-terminal helicase domain of the SNF family helicases (S
SM00490- [Helicase, C-terminal (Helicase_C) - helicase superfamily
PS51348- [Glycoside hydrolase, family 22 (Glyco_hydro_22) - Alpha
cd16899- [C-type invertebrate lysozyme (LYZ_C_invert)]
PF00062- [Glycoside hydrolase, family 22 (Glyco_hydro_22) - C-type
cd13399- [Slt35-like lytic transglycosylase (Slt35_like)]
cd16896- [Uncharacterized lytic transglycosylase subfamily with sin
PF01464- [Transglycosylase SLT domain 1 (Transglycosylase_SLT_do
cd00254- [Lytic transglycosylase (LT) and goose egg-white lysozyme
cd13401- [70kDa soluble lytic transglycosylase (Slt70) and similar p
cd01021- [Goose egg-white lysozyme (GEWL)]
cd16893- [Membrane-bound lytic murein transglycosylases MltC an
cd16894- [Membrane-bound lytic murein transglycosylase D and si
cd13400- [IagB-like protein (LT_IagB_like)]
cd13403- [Membrane-bound lytic murein transglycosylase F (MLTF
cd13925- [Core lysozyme-like domain of resuscitation-promoting fa
PF06737- [Transglycosylase-like (Transglycosylas) - Transglycosylase
cd13402- [lytic transglycosylase-like domain of tail fiber-like protein
PF18013- [Phage tail lysozyme (Phage_lysozyme2) - Phage tail lysoz
cd14814- [Metalloproteases including zinc D-Ala-D-Ala carboxypept
cd14852- [L,D-carboxypeptidase DacB and LdcB, and related protei
PF02557- [Peptidase M15B (Pept_M15B) - D-alanyl-D-alanine carbo
cd14849- [D-Ala-D-Ala dipeptidase/D-Ala-D-Ala carboxypeptidase (
cd14844- [Proteins similar to the zinc-containing D-Ala-D-Ala dipep
PF08291- [Peptidase M15A, C-terminal (Peptidase_M15A_C) - Pept
PF06714- [Protein Gp5, N-terminal OB-fold domain (Gp5_OB_N) - G
cd13926- [N-acetylmuramidase domain of the glycosyl hydrolase 10
PF05838- [Protein of unknown function DUF847 (DUF847) - Glycosy
cd00737- [Endolysin/autolysin (Endolysin_autolysin) - endolysin an
cd16904- [pesticin C-terminal-like domain of uncharacterized prote
cd00736- [Bacteriophage lambda lysozyme and similar proteins (lam
cd16900- [endolysin R21-like proteins (endolysin_R21_like)]
cd16901- [P1 lysozyme Lyz-like proteins (lyz_P1)]
PF00959- [Glycoside hydrolase, family 24 (Glyco_hydro_24) - Phage
cd00735- [Bacteriophage T4-like lysozymes (T4_like_lys)]
PF07669- [Restriction modification methylase Eco57I (RM_methyla
cd02440- [S-adenosylmethionine-dependent methyltransferases (S
PF05175- [Methyltransferase small domain (Small_mtfrase_dom) -
cd16891- [CwlT-like N-terminal lysozyme domain and similar doma
PF13702- [Lysozyme-like (Lysozyme_like)]
cd00229- [SGNH_hydrolase, or GDSL_hydrolase, is a diverse family
PF13472- [SGNH hydrolase-type esterase domain (SGNH_hydro) - G
PF02368- [Bacterial Ig-like, group 2 (Big_2) - Bacterial Ig-like domain

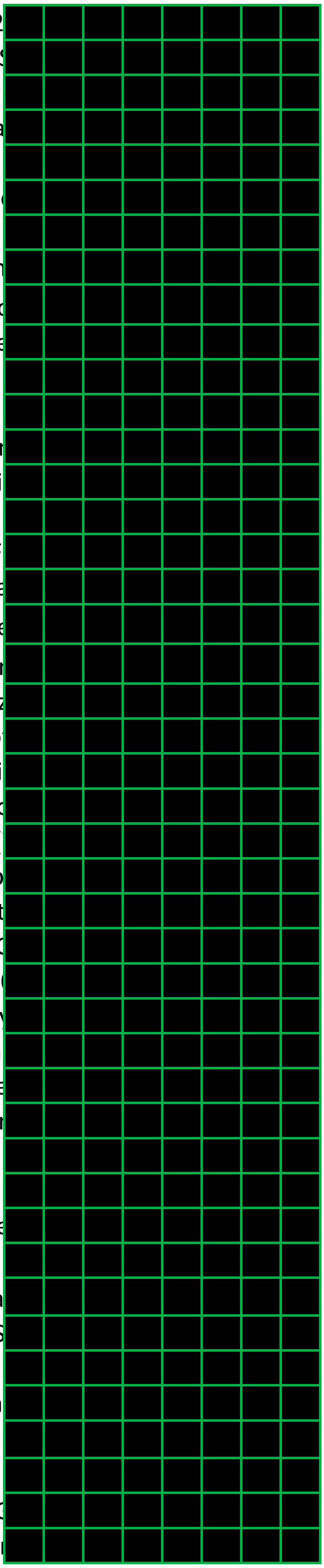

SM00635- [Bacterial Ig-like, group 2 (Big_2) - Bacterial Ig-like doma
PF08230- [Cpl-7 lysozyme, C-terminal (Cpl_7_lyso_C) - CW_7 repea
SM01095- [Cpl-7 lysozyme, C-terminal (Cpl_7_lyso_C) - Cpl-7 lysozy
cd00978- [Glycoside hydrolase, family 46 (Glyco_hydro_46) - chitos
PF01374- [Glycoside hydrolase, family 46 (Glyco_hydro_46) - Glyco
cd16902- [lysozyme-like C-terminal domain of pesticin (pesticin_lyz
PF16754- [Bacterial toxin homologue of phage lysozyme, C-termina
cd00325- [Glycoside hydrolase family 19, chitinase domain (chitina
PF00182- [Glycoside hydrolase, family 19, catalytic (Glyco_hydro_1
cd14845- [L-Ala-D-Glu peptidase, also known as L-alanyl-D-glutama
PF13539- [Peptidase M15C (Peptidase_M15C) - D-alanyl-D-alanine
cd14667- [Non-mltA associated 3D domain containing proteins, na
PF06725- [3D domain (3D_dom) - 3D domain (3D)]
cd14688- [Basic leucine zipper (bZIP) domain of Yeast Activator Pro
PF13884- [Intramolecular chaperone auto-processing domain (S74_
PS51688- [Intramolecular chaperone auto-processing domain (S74_
cd06503- [F-type ATP synthase, membrane subunit b (ATP-synt_Fo
cd18586- [Six-transmembrane helical domain (6TM) domain of the
cd18577- [Six-transmembrane helical domain 1 (TMD1) of P-glycop
cd18028- [DEXH-box helicase domain of archaeal Ski2-type helicase
cd17504- [Methylenomycin A resistance protein (also called MMR
cd17477- [YcaD and similar transporters of the Major Facilitator Su
cd17330- [Eukaryotic Solute carrier 46 (SLC46) family, Bacterial Tet
cd17324- [Purine ribonucleoside efflux pump NepI and similar tran
cd17321- [Methylenomycin A resistance protein (also called MMR
cd17040- [ubiquitin-like (Ubl) domain found in a group of small sulf
cd17320- [Multidrug transporter MdfA and similar multidrug resist
cd14830- [delta subunit of the F-COPI complex, N-terminal domain
cd08173- [Sn-glycerol-1-phosphate dehydrogenase (Gro1PDH) cata
cd06180- [Uncharacterized protein YjiJ and similar proteins of the
cd06174- [Major Facilitator Superfamily (MFS)]
cd06173- [Macrolide efflux protein A and similar proteins of the M
cd04631- [CBS pair domains found in archeal 5'-AMP-activated pro
cd02775- [Molybdopterin-Binding, C-terminal (MopB_CT) domain
cd00890- [Prefoldin is a hexameric molecular chaperone complex,
cd00063- [Fibronectin type III (FN3_dom) - Fibronectin type 3 dom
cd00442- [Lysozyme-like domains (Lyz_like)]
PS51272- [S-layer homology domain (SLH_dom) - S-layer homology
PS50861- [Glycine-tRNA synthetase, heterodimeric (Gly-tRNA-synth
PS51186- [GNAT domain (GNAT_dom) - Gcn5-related N-acetyltrans
PS50853- [Fibronectin type III (FN3_dom) - Fibronectin type-III dom
PS50817- [Intein N-terminal splicing region (Intein_N) - Intein N-ter
PS50163- [DNA recombination and repair protein RecA, monomer-
PF18858- [Large polyvalent protein-associated domain 39 (LPD39)

PF18857- [Large polyvalent protein associated domain 38 (LPD38) -
PF18853- [Large polyvalent protein associated domain 37 (LPD37) -
PF18823- [Inorganic pyrophosphatase domain (Inorganic_Pase) - In
PF18809- [Phage-Barnase-EndoU-ColicinE5/D-RelE-like nuclease (P
PF18760- [ADP-Ribosyltransferase in polyvalent proteins (ART-Poly
PF18798- [Large polyvalent protein-associated domain 3 (LPD3) - L
PF18341- [PSA endolysin, cell wall binding domain (PSA_CBD) - PSA
PF17969- [L,D-transpeptidase C-terminal domain (Ldt_C) - L,D-tran
PF16838- [Distal tube protein, N-terminal (Caud_tail_N) - Caudovir
PF16775- [Lytic exoenzyme, target recognition domain (ZoocinA_T
PF15711- [ILEI/PANDER domain (ILEI/PANDER_dom) - Interleukin-li
PF13810- [Domain of unknown function DUF4185 (DUF4185) - Don
PF13751- [Transposase DDE domain (Tnp_DDE_dom) - Transposase
PF13550- [Tip attachment protein J (J_dom) - Putative phage tail pr
PF13385- [Concanavalin A-like lectin/glucanases superfamily (Lami
PF13529- [Peptidase C39-like (Peptidase_C39-like) - Peptidase_C39
PF13365- [Trypsin-like peptidase domain (Trypsin_2)]
PF13354- [Beta-lactamase enzyme family (Beta-lactamase2)]
PF13199- [Glycosyl hydrolase family 66 (Glyco_hydro_66) - Glycosy
PF12385- [Peptidase C70, AvrRpt2 (Peptidase_C70_AvrRpt2) - Papa
PF12200- [Domain of unknown function DUF3597 (DUF3597) - Don
PF12123- [N-acetylmuramoyl-l-alanine amidase (Amidase02_C) - N
PF11860- [N-acetylmuramidase (Muramidase) - N-acetylmuramida
PF11650- [Tail accessory factor GP4 (Tail_accessory_Gp4) - P22 tail
PF10145- [Phage tail tape measure protein (Phage_tape_meas) - P
PF10464- [Peptidase U40 (Peptidase_U40) - Peptidase U40 (Peptid
PF09718- [Bacteriophage lambda, GpH, tail tape measure, C-termin
PF09588- [YqaJ viral recombinase (YqaJ_viral_recombinase) - YqaJ-
PF09374- [Peptidoglycan binding domain (Peptidoglycan-bd_3) - Pr
PF08924- [Protein of unknown function DUF1906 (DUF1906) - Dom
PF08481- [GBS Bsp-like (GBS_Bsp-like) - GBS Bsp-like repeat (GBS_
PF08310- [LGFP (LGFP) - LGFP repeat (LGFP)]
PF07902- [Gp58-like (Gp58) - gp58-like protein (Gp58)]
PF07833- [Copper amine oxidase-like, N-terminal (Cu_amine_oxida
PF06832- [Penicillin-binding, C-terminal (PBP_C) - Penicillin-Binding
PF07486- [Cell wall hydrolase, SleB (Cell_wall_hydrolase_SleB) - Ce
PF02384- [DNA methylase, adenine-specific (DNA_methylase_A-5)
PF02018- [Carbohydrate-binding, CenC-like (CenC_carb-bd) - Carbo
PF01664- [Viral attachment sigma 1, reoviral (Vir_attach_sigma1_r
PF01471- [Peptidoglycan binding-like (Peptidoglycan-bd-like) - Puta
PF01391- [Collagen triple helix repeat (Collagen) - Collagen triple h
PF01129- [NAD:arginine ADP-ribosyltransferase, ART (ART) - NAD:a
PF00912- [Glycosyl transferase, family 51 (Glyco_trans_51) - Transg
PF00905- [Penicillin-binding protein, transpeptidase (PCN-bd_Tpep

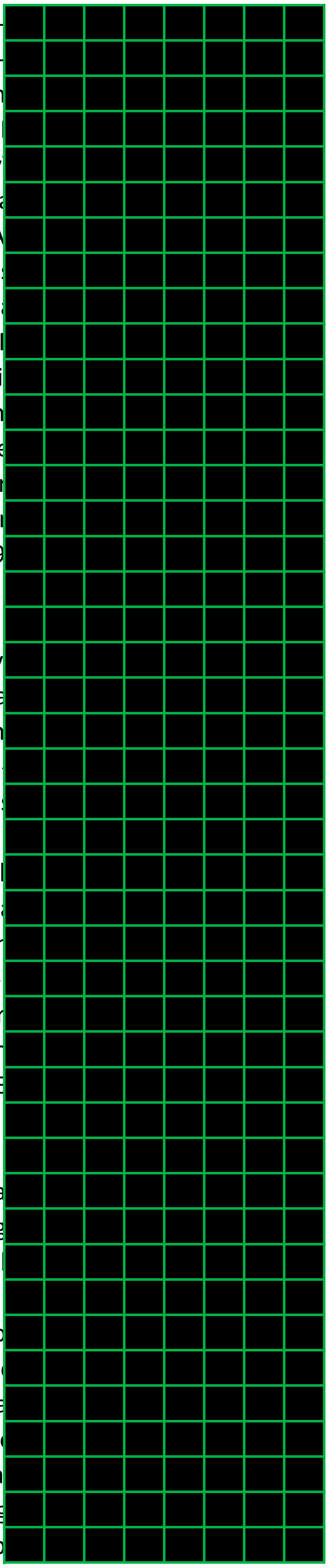

PF00395- [S-layer homology domain (SLH_dom) - S-layer homology
PF00753- [Metallo-beta-lactamase (Metallo-B-lactamas) - Metallo-
PF06791- [Bacteriophage lambda, GpH, tail tape measure, N-termi
PF06715- [Gp5, C-terminal (Gp5_C) - Gp5 C-terminal repeat (3 copi
PF05598- [Transposase InsH, N-terminal (Transposase_InsH_N) - Tr
PF05435- [DNA terminal protein Gp3 (DNA_terminal_Gp3) - Phi-29
PF05382- [Bacteriophage lysin (Phage_lysin) - Bacteriophage peptic
PF05203- [Hom-end-associated Hint (Hom_end_hint) - Hom_end-a
PF05106- [Bacteriophage lambda, GpS, holin (Phage_lambda_GpS_
PF04965- [IraD/Gp25-like (IraD/Gp25-like) - Gene 25-like lysozyme
PF03330- [RlpA-like protein, double-psi beta-barrel domain (RlpA-li
PF04466- [Phage terminase large subunit, N-terminal (Terminase_L
PS50249- [MPN domain (MPN) - MPN domain profile (MPN)]
SM00232- [JAB1/MPN/MOV34 metalloenzyme domain (JAMM/MP
cd08073- [Mpr1p, Pad1p N-terminal (MPN) domains with catalytic
PF14464- [JAB domain, prokaryotic (JAB_dom_prok) - Prokaryotic h
PF00877- [Endopeptidase, NLPC/P60 domain (NLP_P60_dom) - Nlp
PF01551- [Peptidase M23 (Peptidase_M23) - Peptidase family M23
PF01473- [Cell wall/choline-binding repeat (Cell_wall/Cho-bd_repe
PS51170- [Cell wall/choline-binding repeat (Cell_wall/Cho-bd_repe
PS51257- [Prokaryotic membrane lipoprotein lipid attachment site
PF01832- [Mannosyl-glycoprotein endo-beta-N-acetylglucosamidas
SM00047- [Mannosyl-glycoprotein endo-beta-N-acetylglucosamida
PF08239- [SH3-like domain, bacterial-type (SH3-like_bac-type) - Ba
PF08460- [SH3-like domain, bacterial-type (SH3-like_bac-type) - Ba
PS51781- [SH3-like domain, bacterial-type (SH3-like_bac-type) - SH
SM00287- [SH3-like domain, bacterial-type (SH3-like_bac-type) - Ba
PF05257- [CHAP domain (CHAP_dom) - CHAP domain (CHAP)]
PS50911- [CHAP domain (CHAP_dom) - CHAP domain profile (CHAP

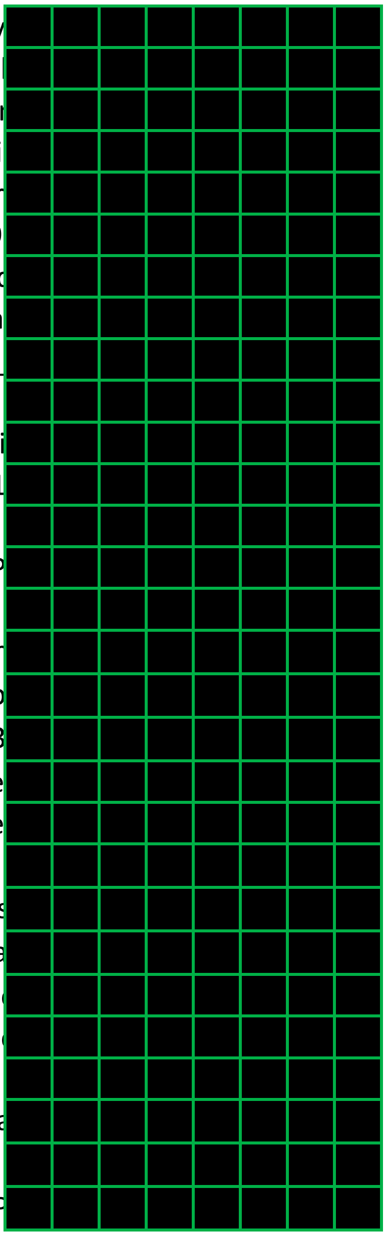

ap (average fraction between overlapping section en length of largest domain)

	PF01476	cd00118	PS51782	SM00257	cd00599	cd06417	cd06415	cd06414	cd06525	PF01183	SM00641	cd06522	cd06523	PF05036	PS51724	SM00646	cd02696	PF01520	PS50222	SM00644	SM00701	cd06583	PF01510	PF00565	PS50830	SM00318	cd02883
	1	1	1	1																							
	1	1	1	1																							
	1	1	1	1																							
	1	1	1	1																							
					1					1	1																
						1				1	1																
							1			1	1																
								1		1	1																
									1	1	1																
					1	1	1	1	1	1	1	1	1														
					1	1	1	1	1	1	1																
										1		1															
										1			1														
														1	1	0	0	0		0							
														1	1												
														0		1	1	1									
														0		1	1	1									
														0		1	1	1									
																			1	0							
														0					0	1	1	1	1				
																				1	1	1	1				
																				1	1	1	1				
																				1	1	1	1				
																								1	1	1	
																								1	1	1	
																								1	1	1	
																											1
																											1
																											1

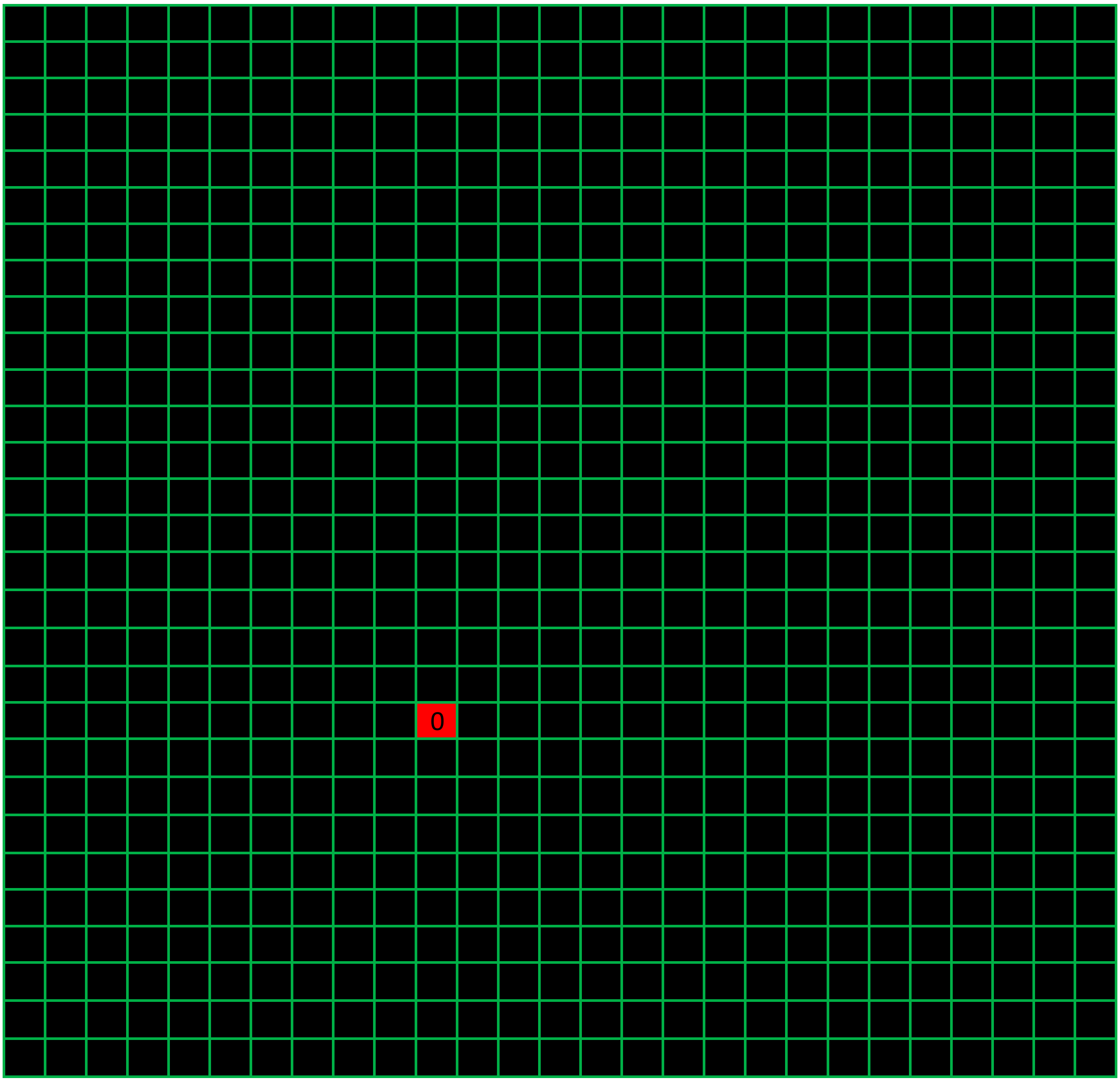
0

PF00293	PS51462	PF10908	cd16913	PF03734	PS51194	cd18793	SM00490	PS51348	cd16899	PF00062	cd13399	cd16896	PF01464	cd00254	cd13401	cd01021	cd16893	cd16894	cd13400	cd13403	cd13925	PF06737	cd13402	PF18013	cd14814	cd14852
1	1																									
1	1																									
1	1																									
		1	1	1																						
		1	1	1																						
		1	1	1																						

					1	1	1																			
					1	1	1																			
					1	1	1																			
								1	0	0																
								0	1	1			1													
								0	1	1																
											1		1													
												1	1	0												
									1		1	1	1	1	1	1	1	1	1	1	1		1			
												0	1	1	0									0		
													1	0	1											
													1			1										
													1				1									
													1					1								
													1						1							
													1							1						
													1								1	1				
																					1	1	1			
													1									1	1	1		
														0									1	1		
																									1	
																										1
																									1	1
																									1	

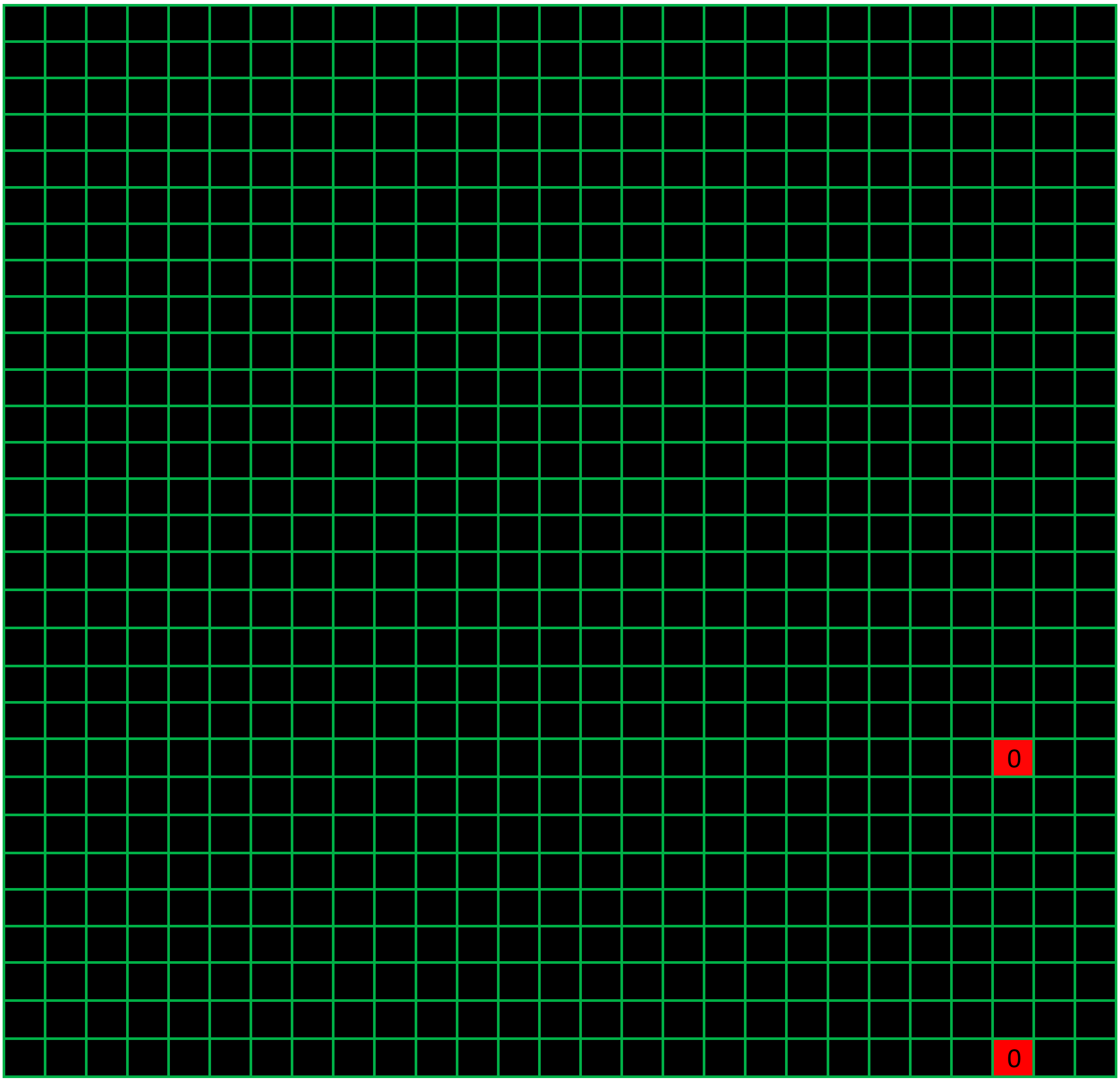
0
0

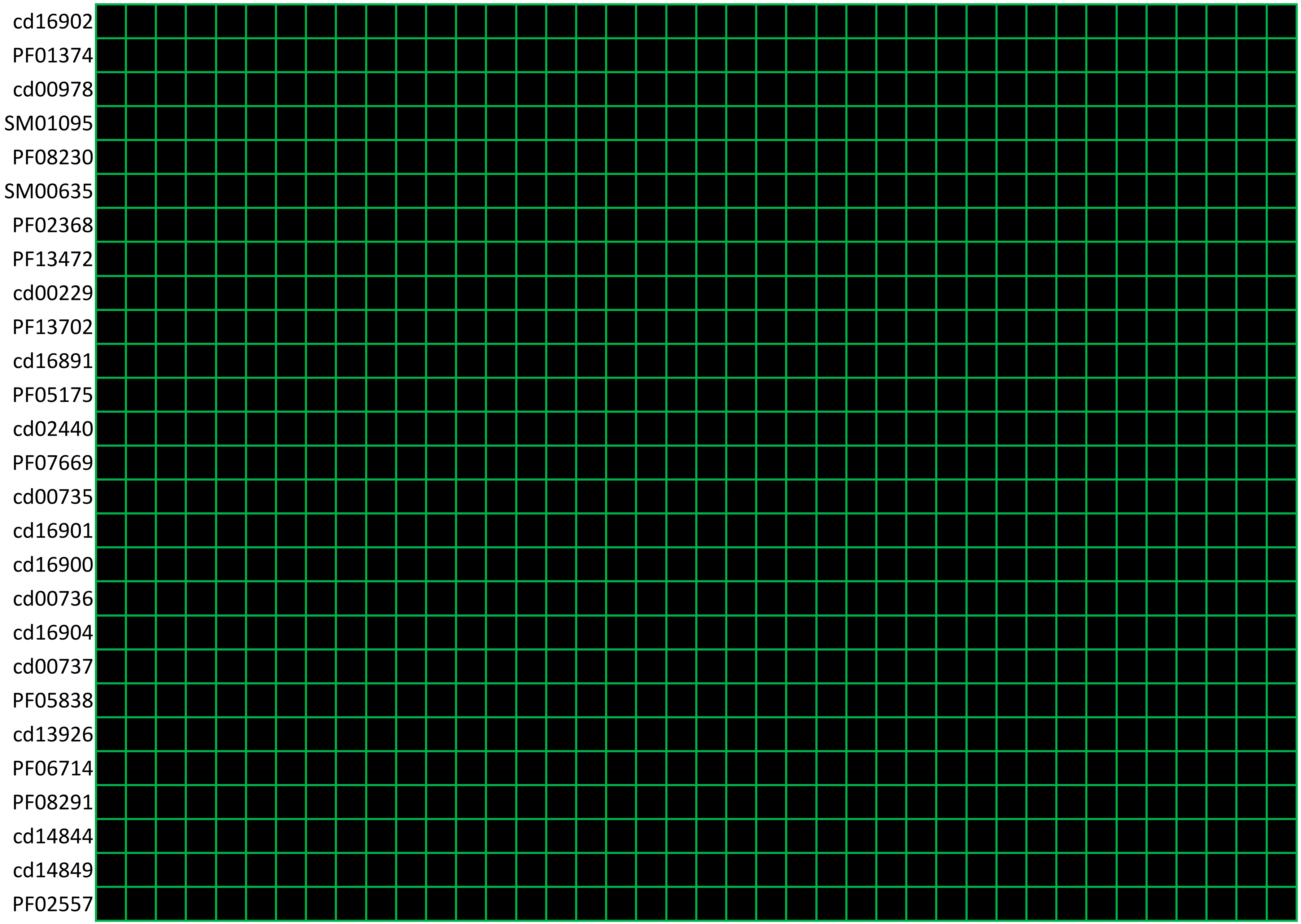
cd16902
PF01374
cd00978
SM01095
PF08230
SM00635
PF02368
PF13472
cd00229
PF13702
cd16891
PF05175
cd02440
PF07669
cd00735
cd16901
cd16900
cd00736
cd16904
cd00737
PF05838
cd13926
PF06714
PF08291
cd14844
cd14849
PF02557

1			1																							
1																										
1																										
	1		1																							
		1	1																							
	1	1	1																							
				1								0														
					1	1	0																			
					1	1																				
					0		1	0																		
							0	1																		
									1																	
										1																
											1															
							1		1	1	1	1														
				0								1														
													1	0												
													0	1	1											
														1	1											
																1	1									
																1	1									
																		1	1							
																		1	1							
																				1	1					

																				1	1					
																						1	1			
																						1	1			
																								1	1	
																								1	1	
																										1
																										1

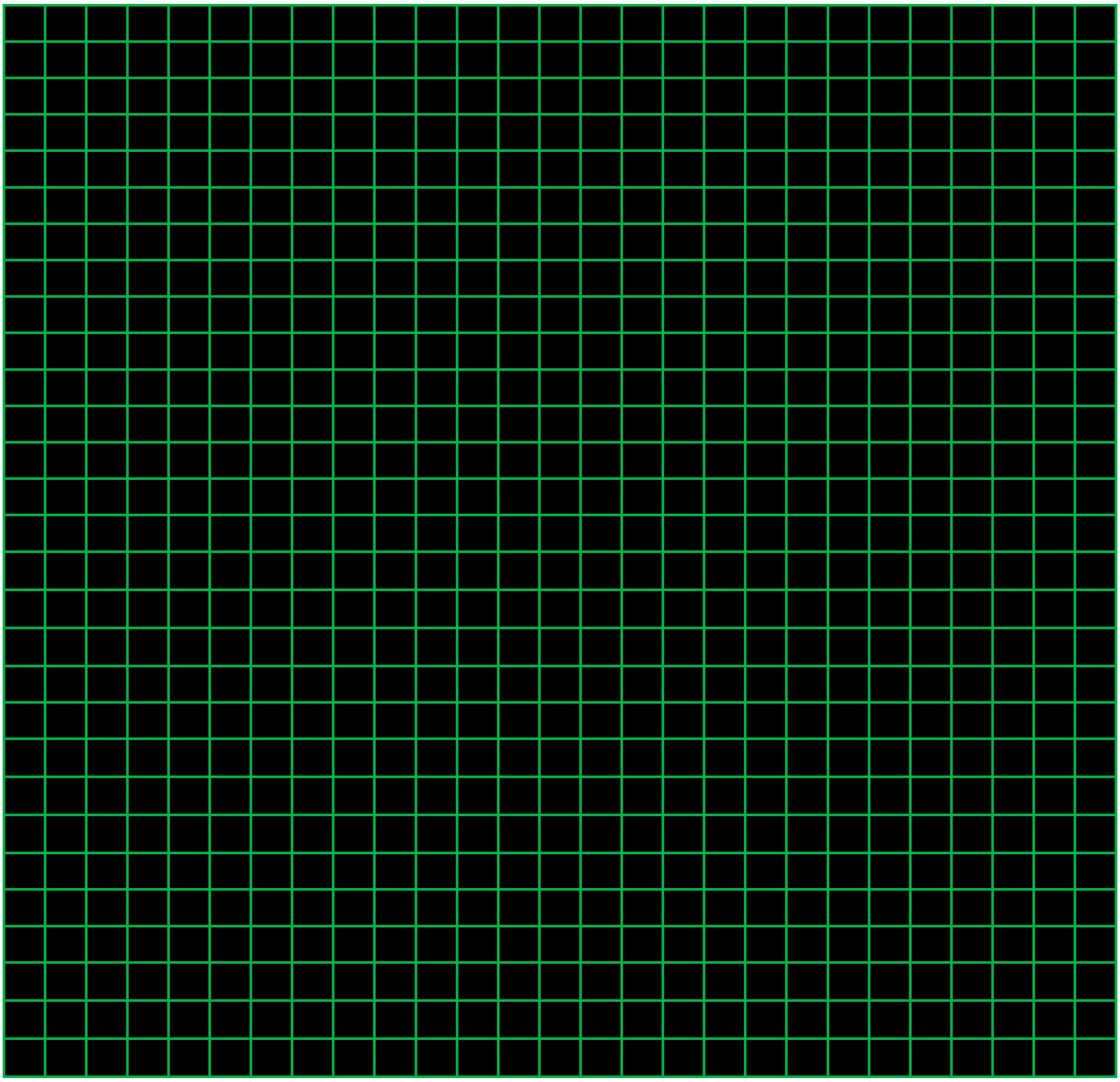

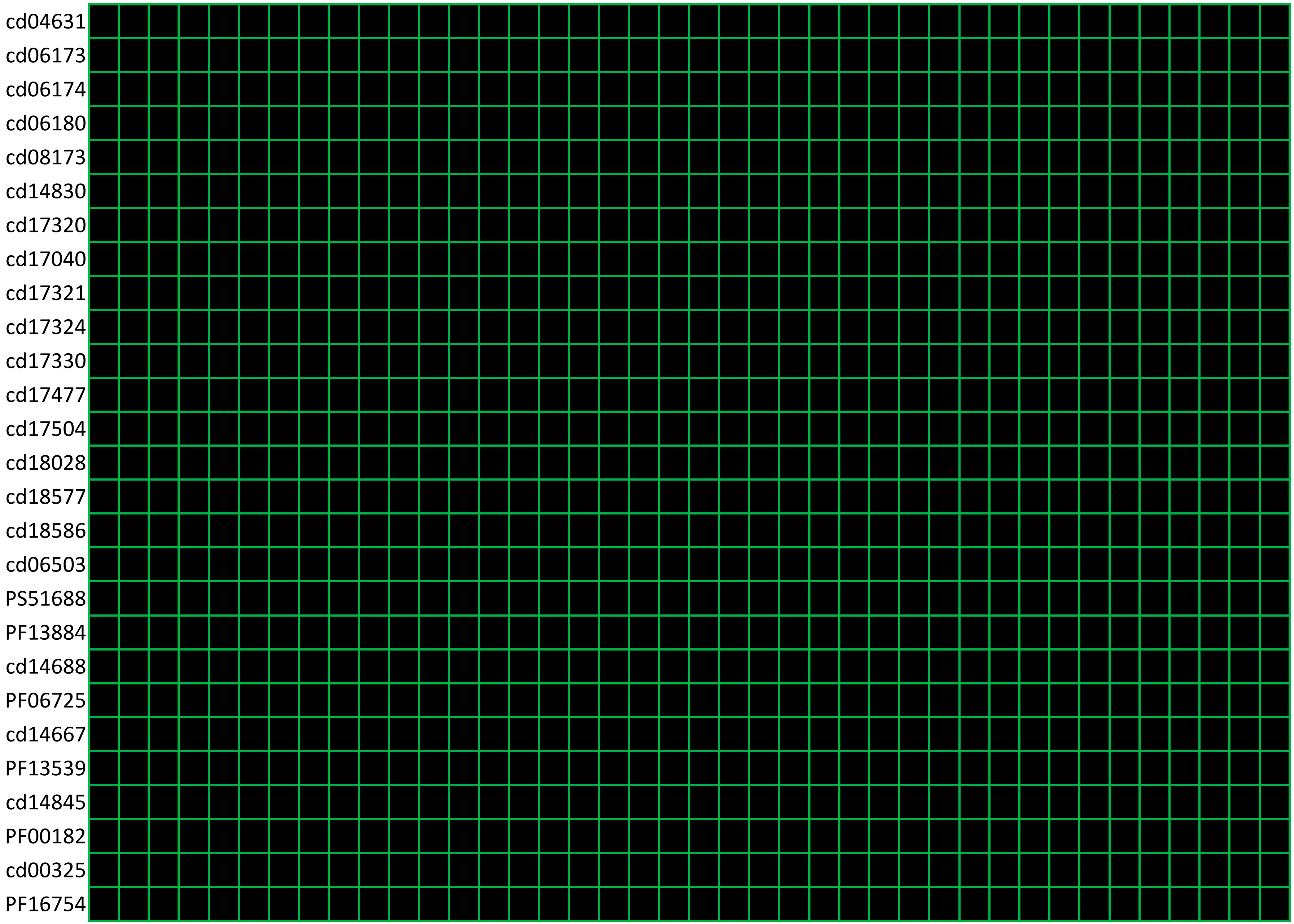
cd04631
cd06173
cd06174
cd06180
cd08173
cd14830
cd17320
cd17040
cd17321
cd17324
cd17330
cd17477
cd17504
cd18028
cd18577
cd18586
cd06503
PS51688
PF13884
cd14688
PF06725
cd14667
PF13539
cd14845
PF00182
cd00325
PF16754

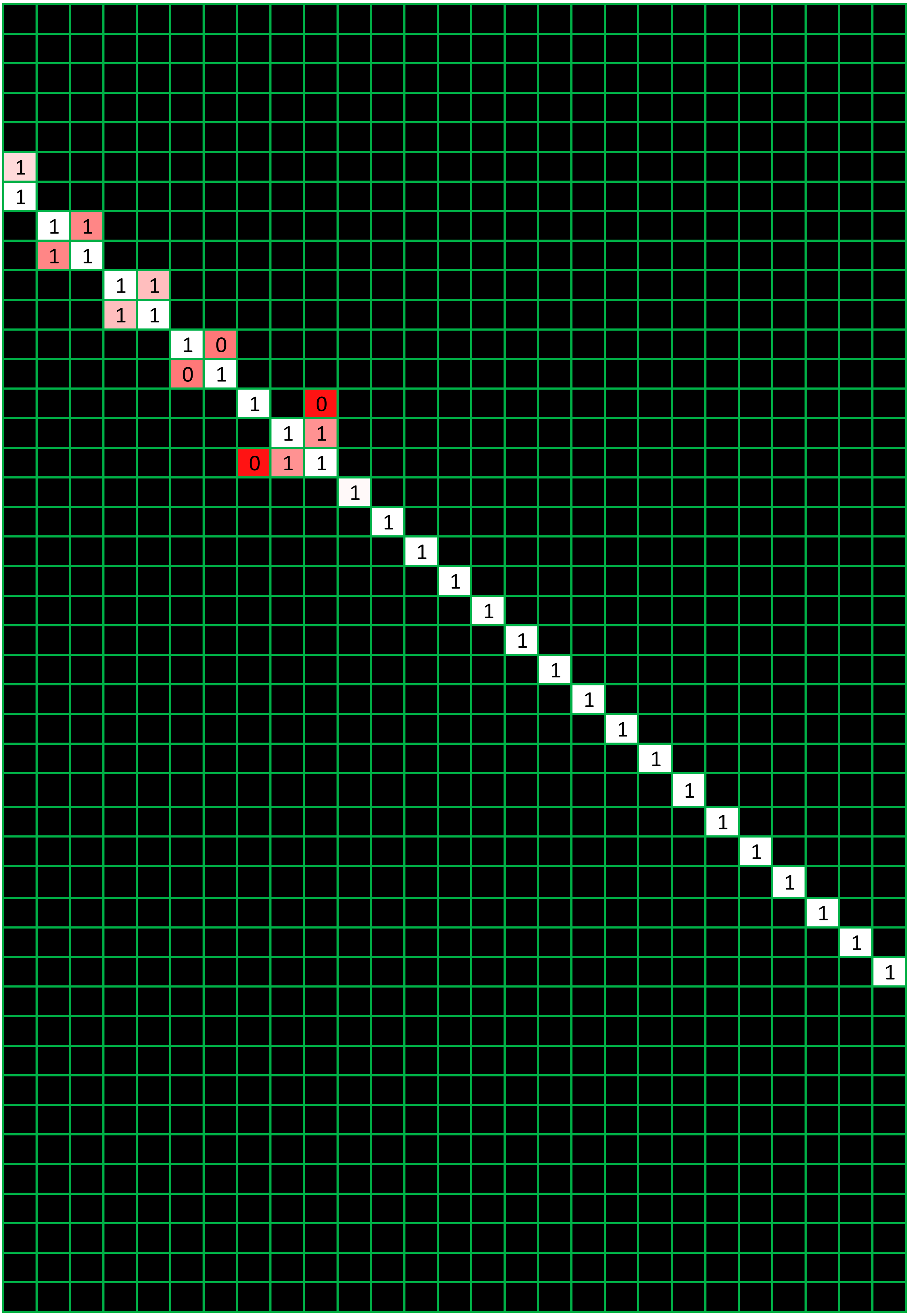

1
1
1 1
1 1
1 1
1 1
1 0
0 1
1 0
1 1
0 1 1
1
1
1
1
1
1
1
1
1
1
1
1
1
1
1
1
1

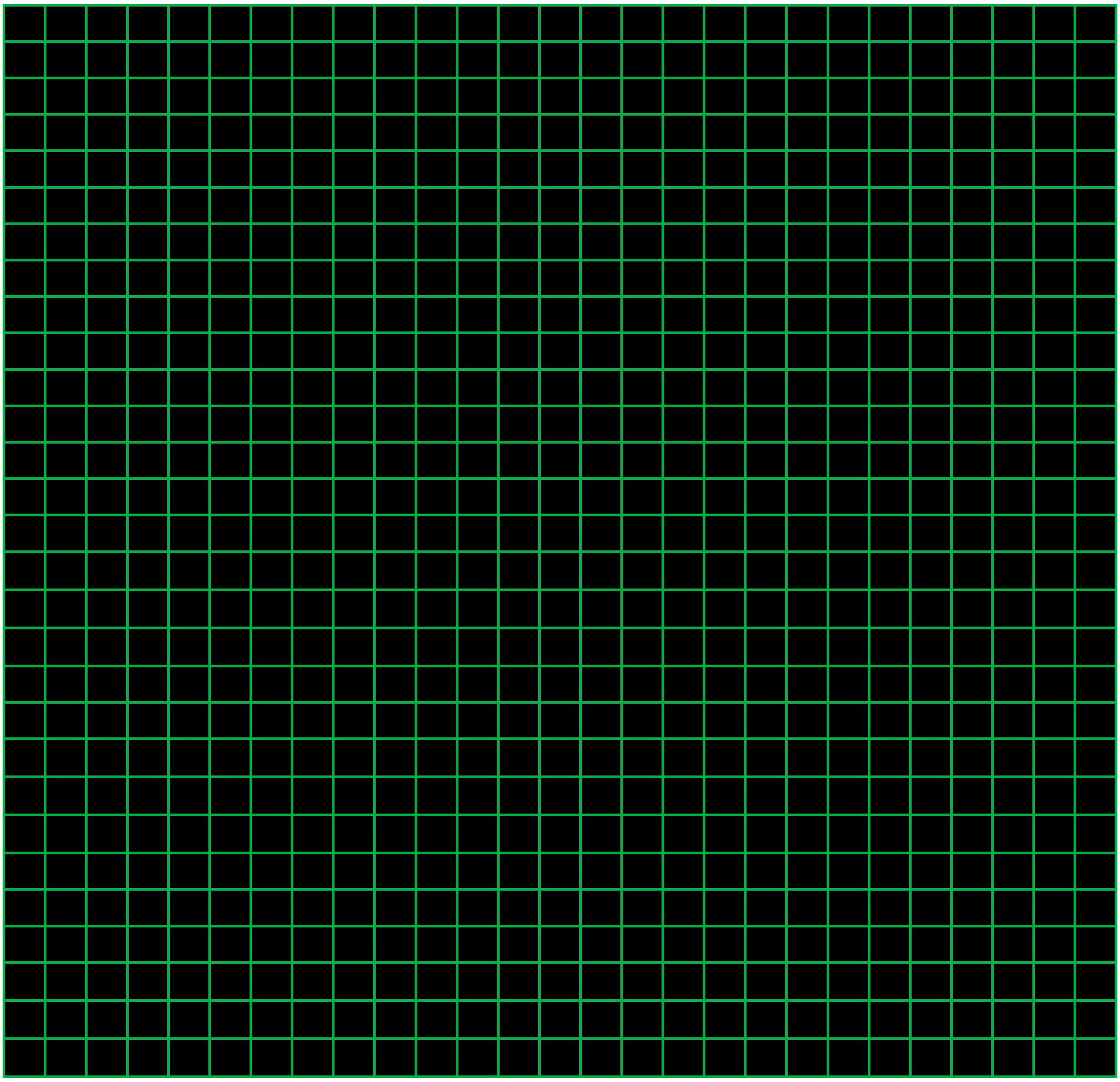

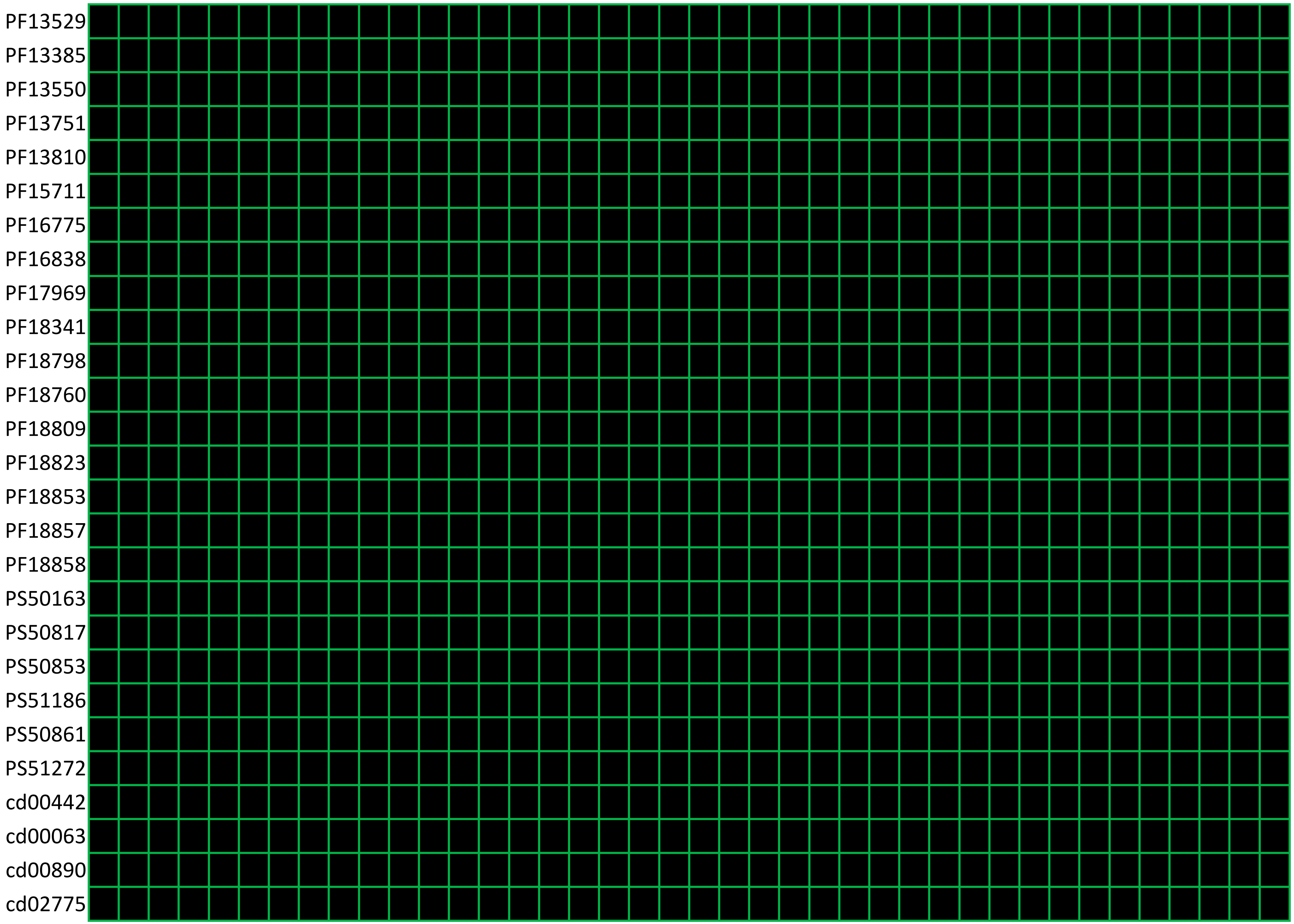
PF13529
PF13385
PF13550
PF13751
PF13810
PF15711
PF16775
PF16838
PF17969
PF18341
PF18798
PF18760
PF18809
PF18823
PF18853
PF18857
PF18858
PS50163
PS50817
PS50853
PS51186
PS50861
PS51272
cd00442
cd00063
cd00890
cd02775

1																										
	1																									
		1																								
			1																							
				1																						
					1																					
						1																				
							1																			
								1																		
									1																	
										1																

											1															
												1														
													1													
														1												
															1											
																1										
																	1									
																		1								
																			1							
																				1						
																					1					
																						1				
																							1			
																								1		
																									1	
																										1

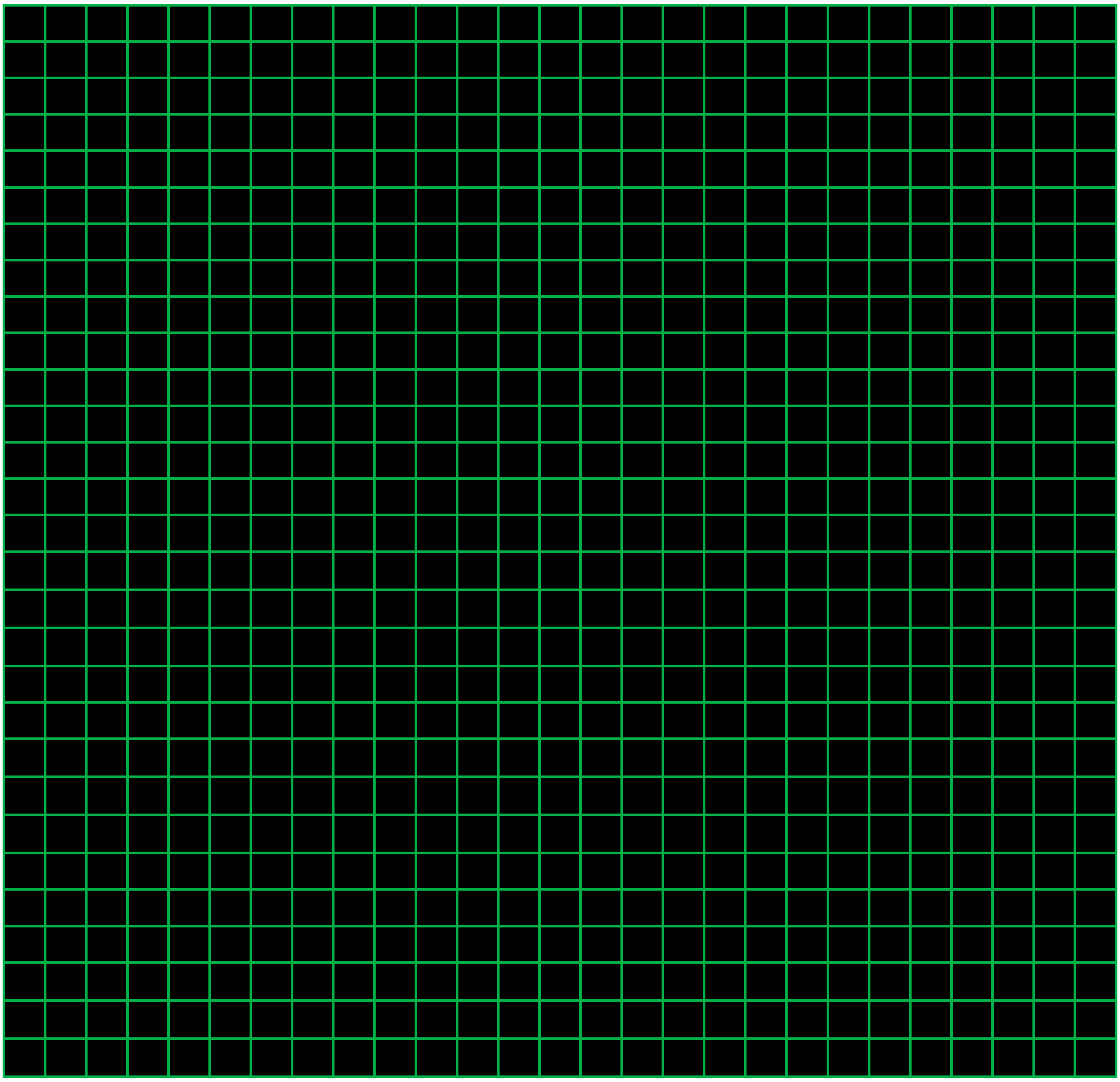

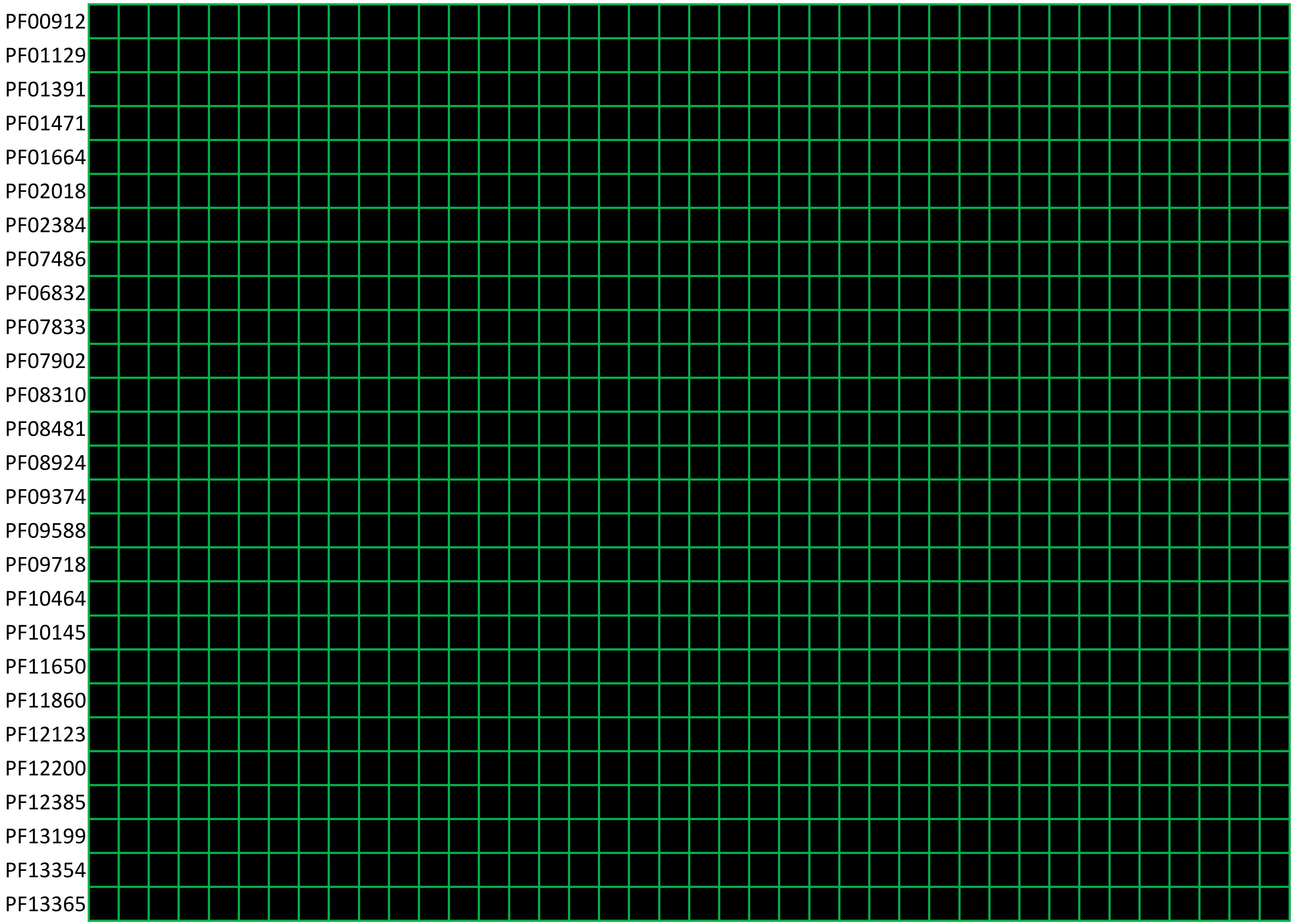
PF00912
PF01129
PF01391
PF01471
PF01664
PF02018
PF02384
PF07486
PF06832
PF07833
PF07902
PF08310
PF08481
PF08924
PF09374
PF09588
PF09718
PF10464
PF10145
PF11650
PF11860
PF12123
PF12200
PF12385
PF13199
PF13354
PF13365

1																										
	1																									
		1																								
			1																							
				1																						
					1																					
						1																				
							1																			
								1																		
									1																	
										1																
											1															
												1														
													1													
														1												
															1											
																1										
																	1									
																		1								
																			1							
																				1						
																					1					
																						1				
																							1			
																								1		
																									1	
																										1

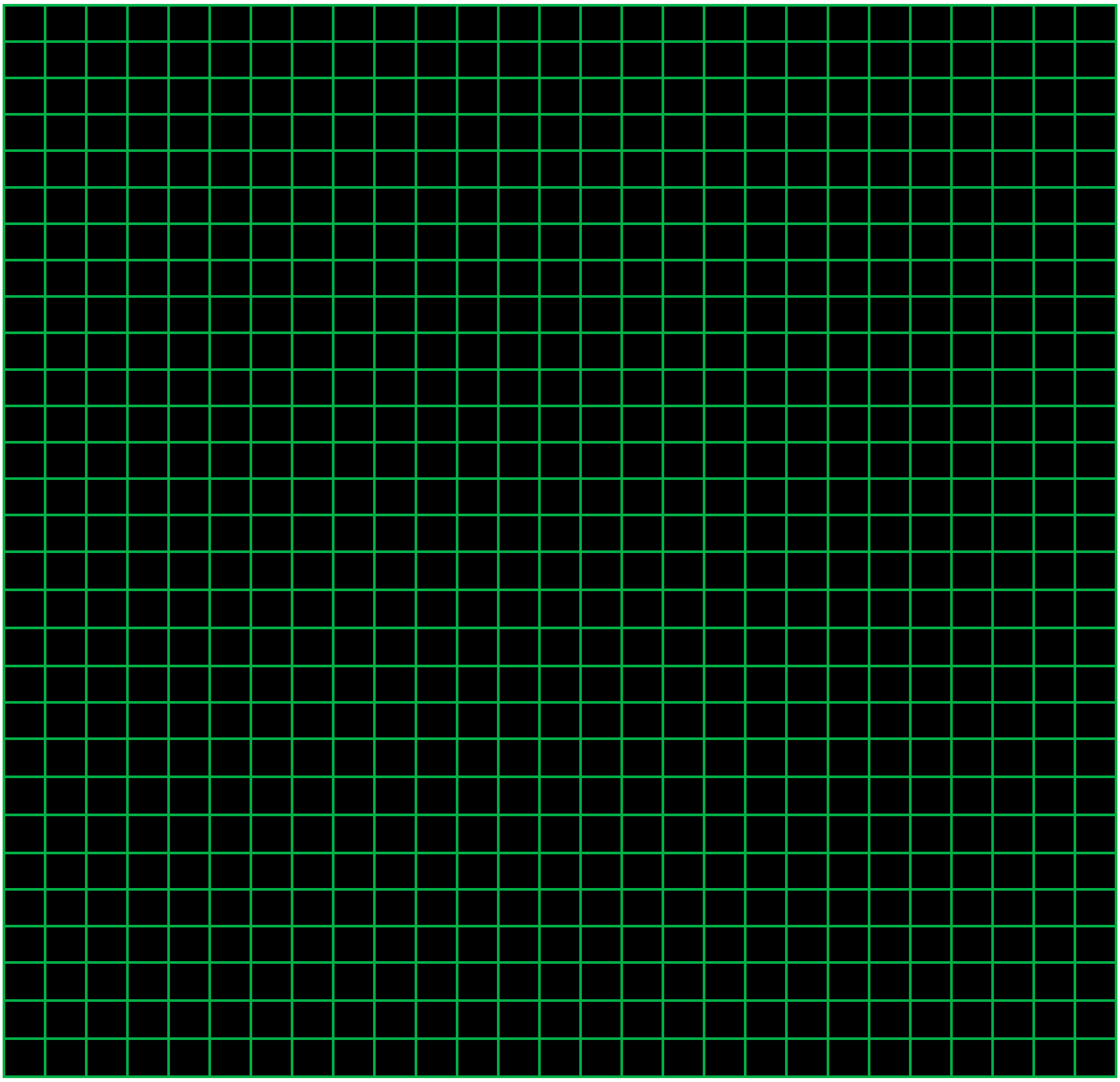

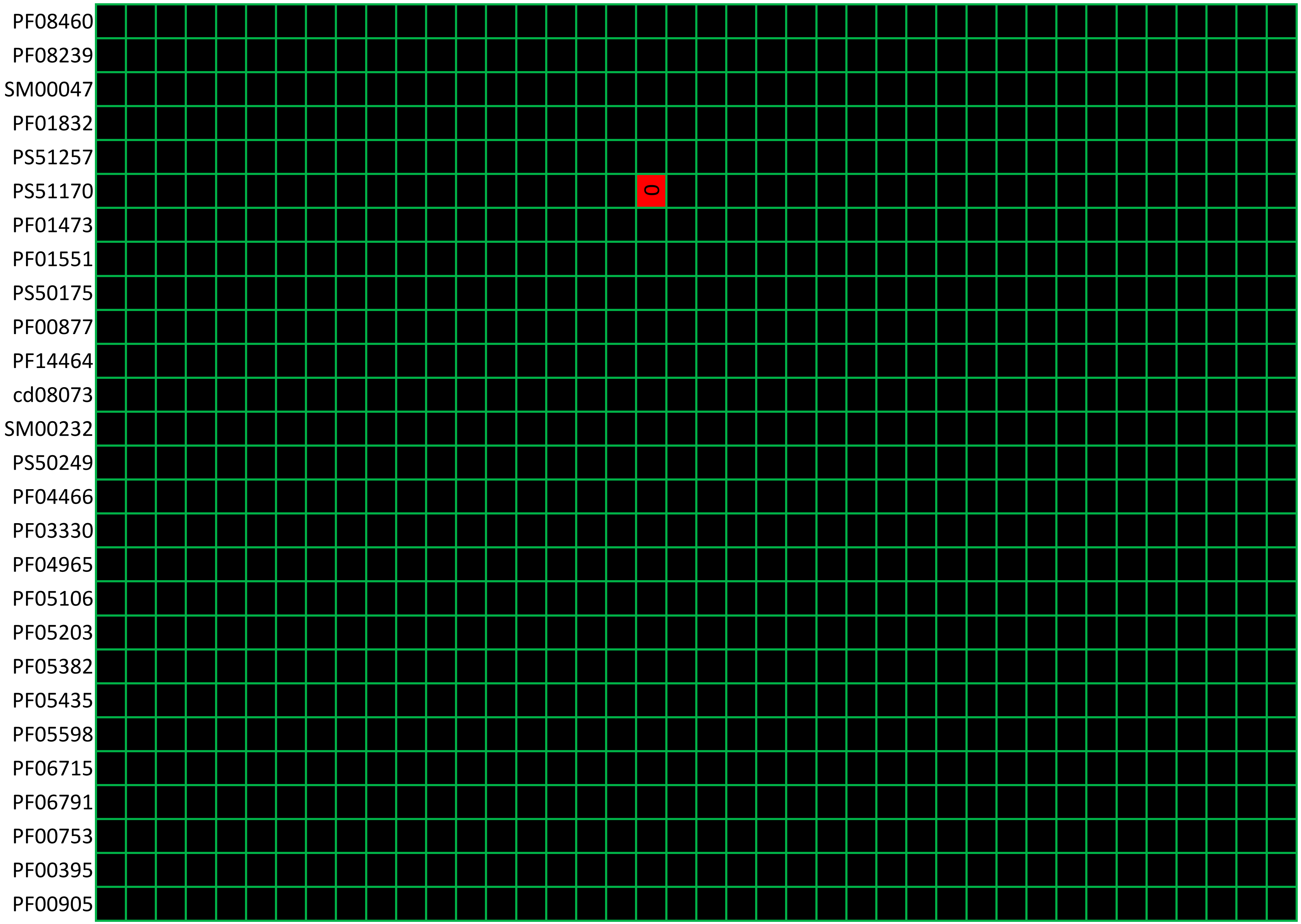
PF08460
PF08239
SM00047
PF01832
PS51257
PS51170
PF01473
PF01551
PS50175
PF00877
PF14464
cd08073
SM00232
PS50249
PF04466
PF03330
PF04965
PF05106
PF05203
PF05382
PF05435
PF05598
PF06715
PF06791
PF00753
PF00395
PF00905
0

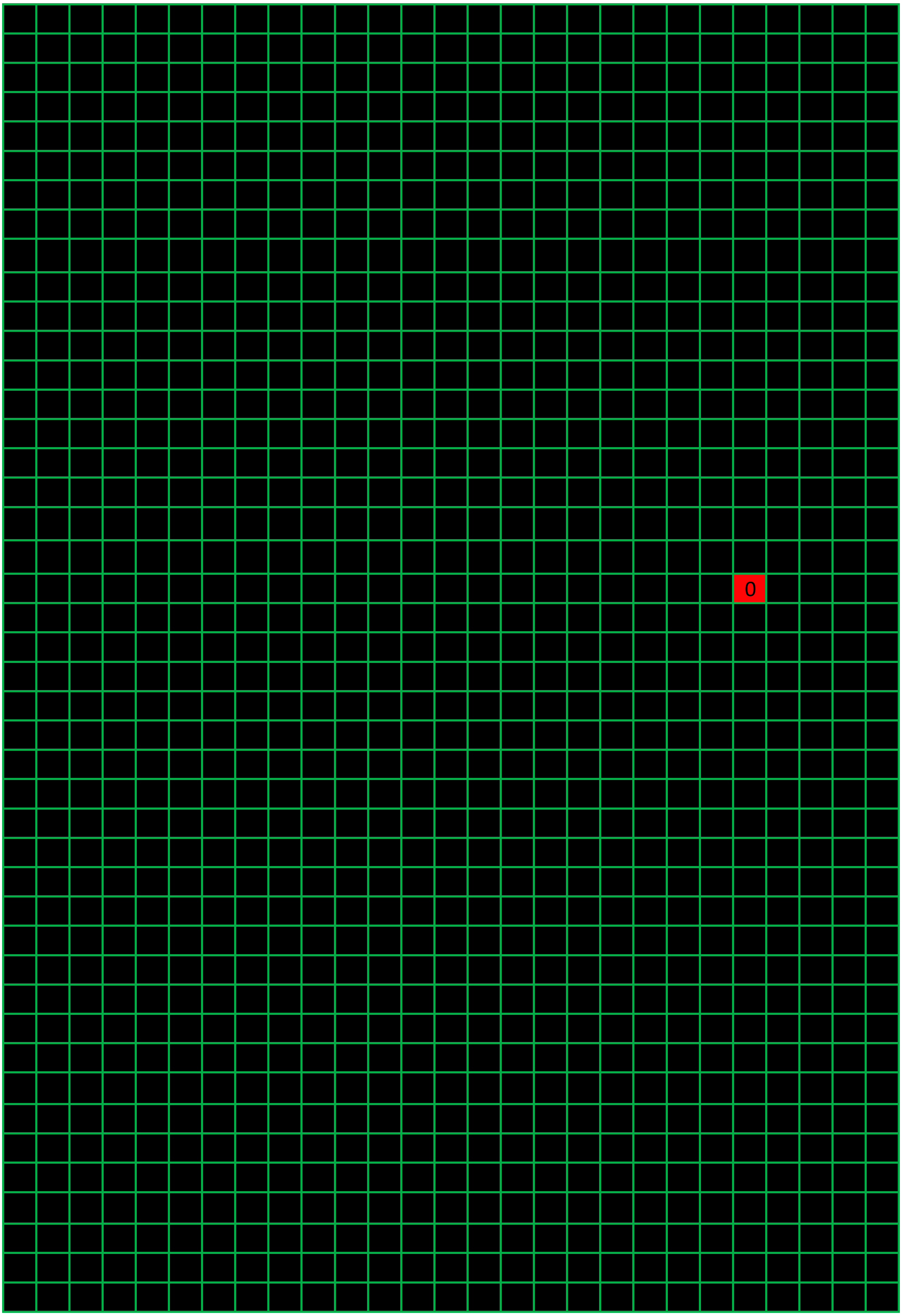
0

1

	1																									
		1																								
			1																							
				1																						
					1																					
						1																				
							1																			
								1																		
									1																	
										1																
											1															
												1														
													1	1	1	1	0									
													1	1	1	1	0									
													1	1	1	1										
													1	1	1	1										
													0	0			1									
																			1							
																				1	1					
																				1	1					
																						1				
																							1	1		
																							1	1		
																									1	
																										1
																									1	1
																									1	1
																					0					
																					0	0				

PS51781	SM00287	PF05257	PS50911

absolute number of overlaps between each pair of do

cd17926	cd18012	PF00176	SM00487	cd18011	PS51192	cd17919	PF04851	PF01476	cd00118	PS51782	SM00257	cd00599	cd06417	cd06415
1			1		1									
	1	1	1											
	1	2	2	1	1									
1	1	2	10	4	7	1	2							
		1	4	4	2		1							
1		1	7	2	7	1	2							
			1		1	1	1							
			2	1	2	1	2							
								330	330	330	327			
								330	332	331	327			
								330	331	333	327			
								327	327	327	327			
												46		
													1	
														61
												46	1	61
												41	1	69

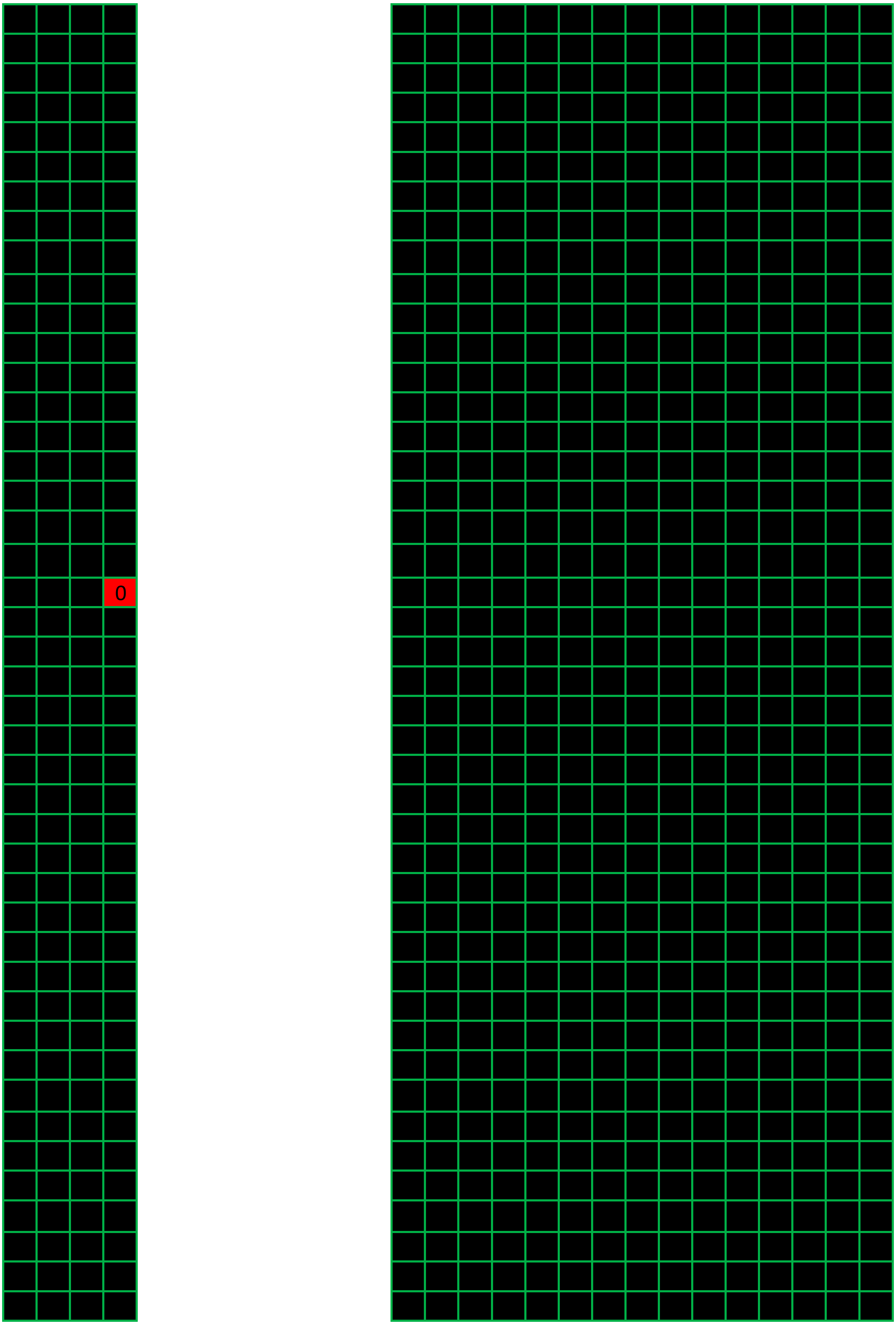
0

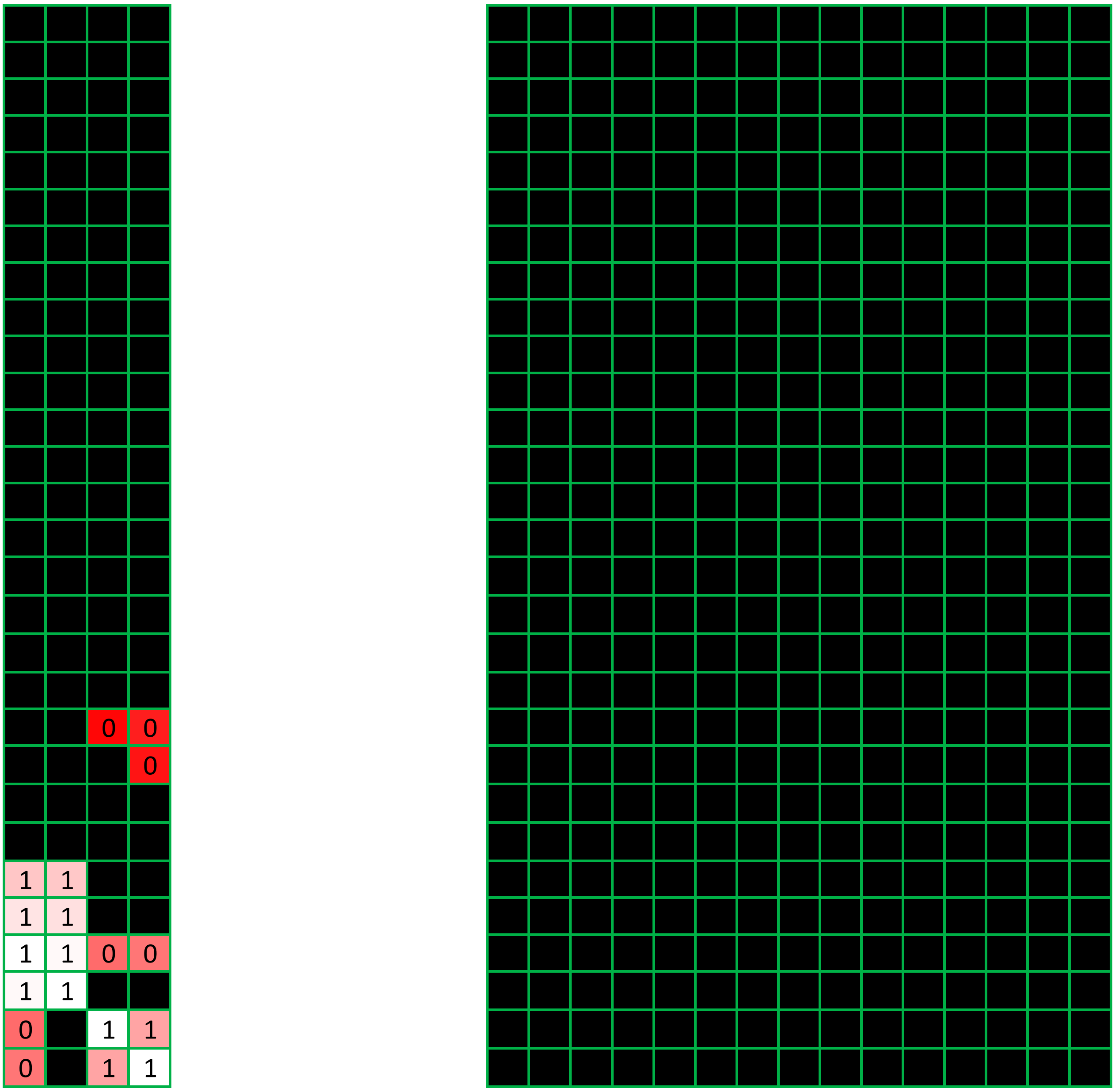
0 0
0
1 1
1 1
1 1 0 0
1 1
0 1 1
0 1 1

main profiles in PhaLP

cd06414	cd06525	PF01183	SM00641	cd06522	cd06523	PF05036	PS51724	SM00646	cd02696	PF01520	PS50222	SM00644	SM00701	cd06583	cd18098	cd18184	PF00177	SM00488	cd18012	PS51193	cd17920	PF01899	PF05274	cd00119	PS51783	SM00258
		46	41																							
		1	1																							
		61	69																							
4		4	1																							
	3	3	3																							
4	3	275	207	1	24																					
1	3	207	207																							
		1		1																						
		24			24																					
						20	15	2	2	2		1														
						15	16																			
						2		194	193	194																
						2		193	209	209																
						2		194	209	213																
											4	1														
						1					1	###	185	###	###											
												185	202	202	201											
												###	202	###	###											
												###	201	###	###											
																1	1	1								
																1	1	1								
																1	1	1								
																			1	1	1					
																			1	1	1					
																			1	1	1					
																						1	1	1		
																						1	7	4		
																						1	4	4		

7	5
5	8
2	4

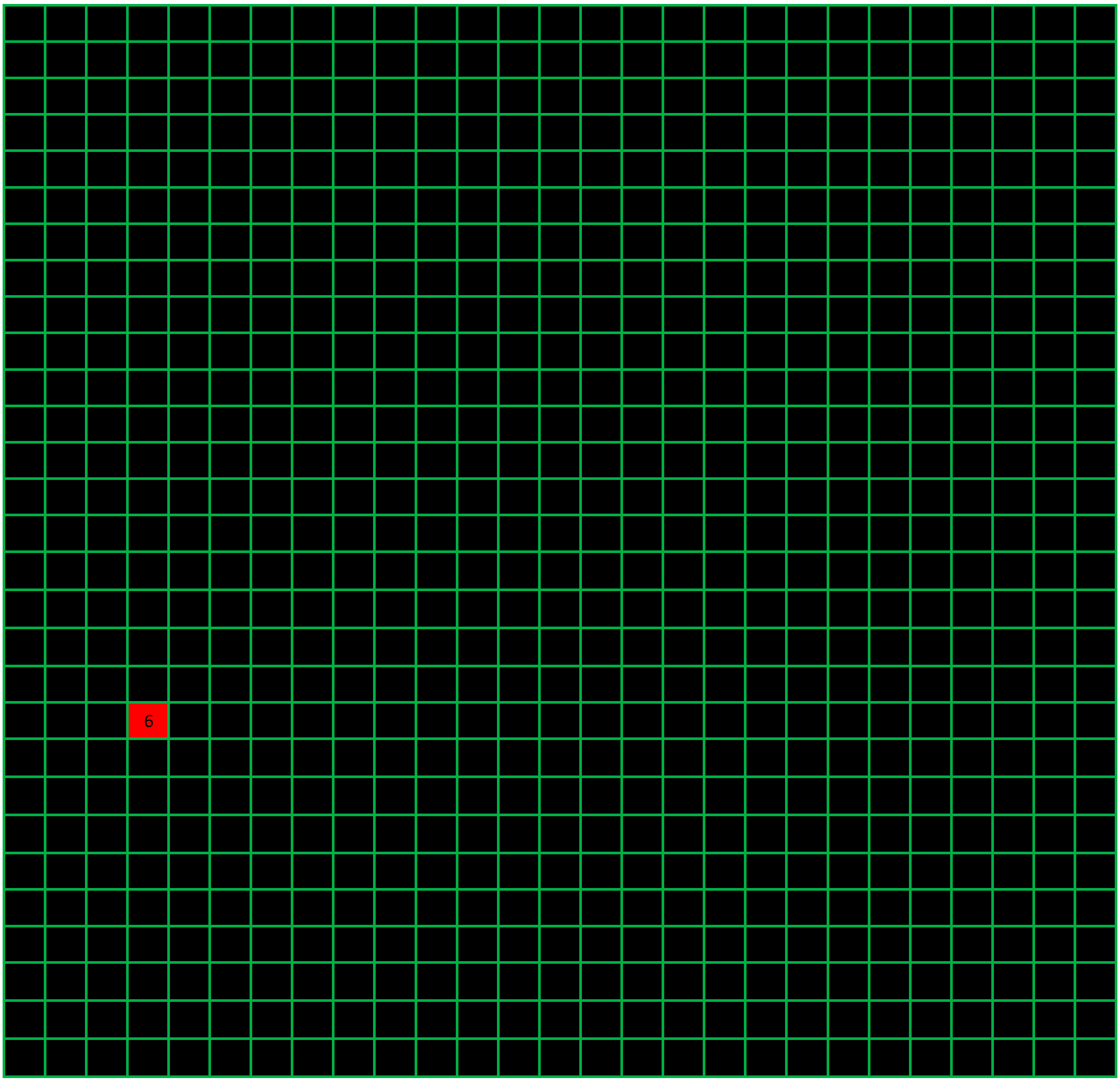
6

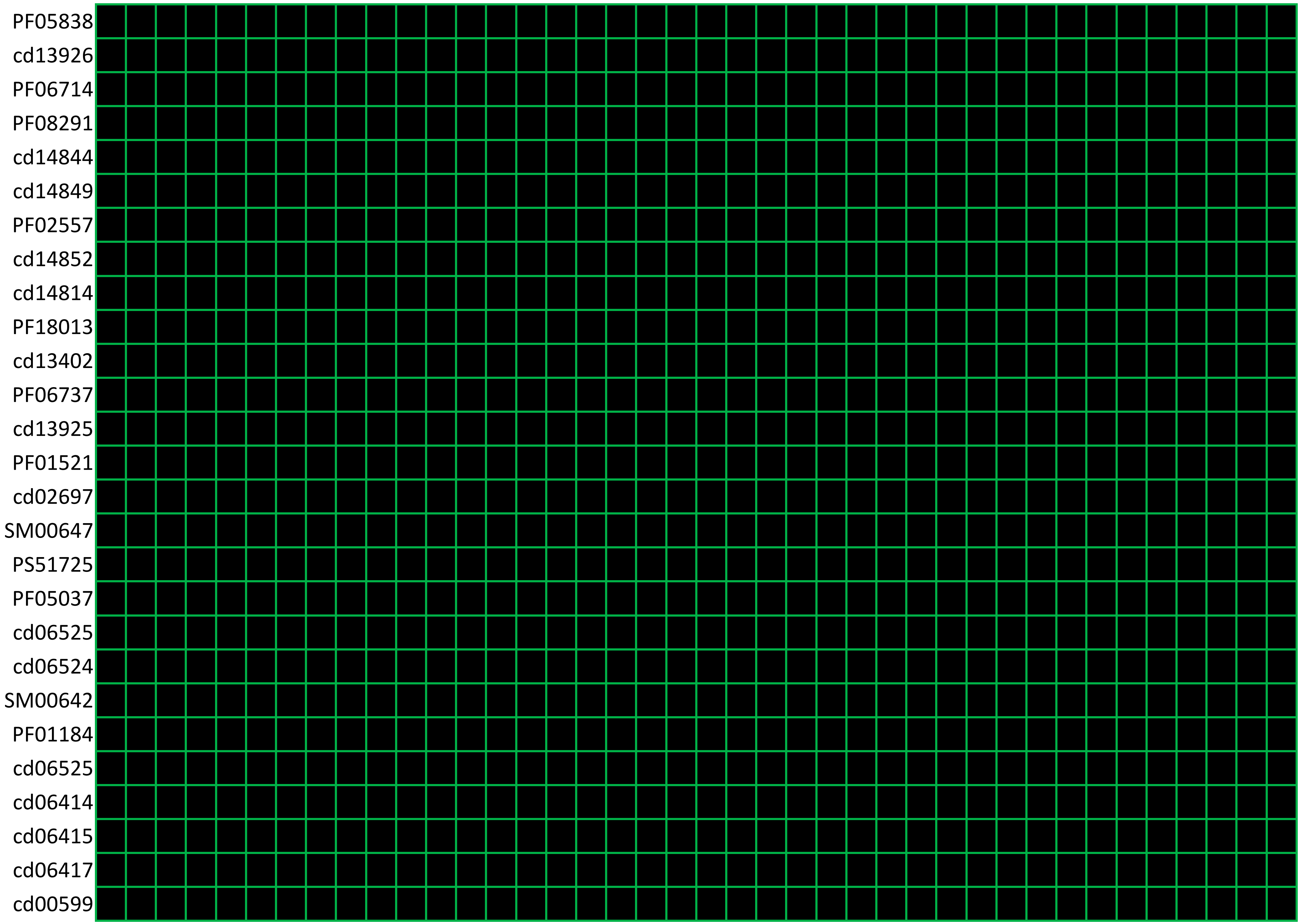
PF05838
cd13926
PF06714
PF08291
cd14844
cd14849
PF02557
cd14852
cd14814
PF18013
cd13402
PF06737
cd13925
PF01521
cd02697
SM00647
PS51725
PF05037
cd06525
cd06524
SM00642
PF01184
cd06525
cd06414
cd06415
cd06417
cd00599

2																										
4																										
4																										
	1	1	1																							
	1	12	1			7																				
	1	1	1																							
				14		10																				
					14	12	1																			
		7		10	12	837	274	43	3	14	85	17	47	14		251										
					1	274	334	1									2									
						43	1	46																		
						3			24																	
						14				15																
						85					91															
						17						50														
						47							71													
						14								182	137											
														137	152	15										
						251									15	462	1									
							2									1	232									
																		94		45			4			
																			2	2						
																		45	2	47						
																					1		1			
																						131	131			
																		4			1	131	156			
																								228		
																									179	173
																									173	172
																									3	
																								1		

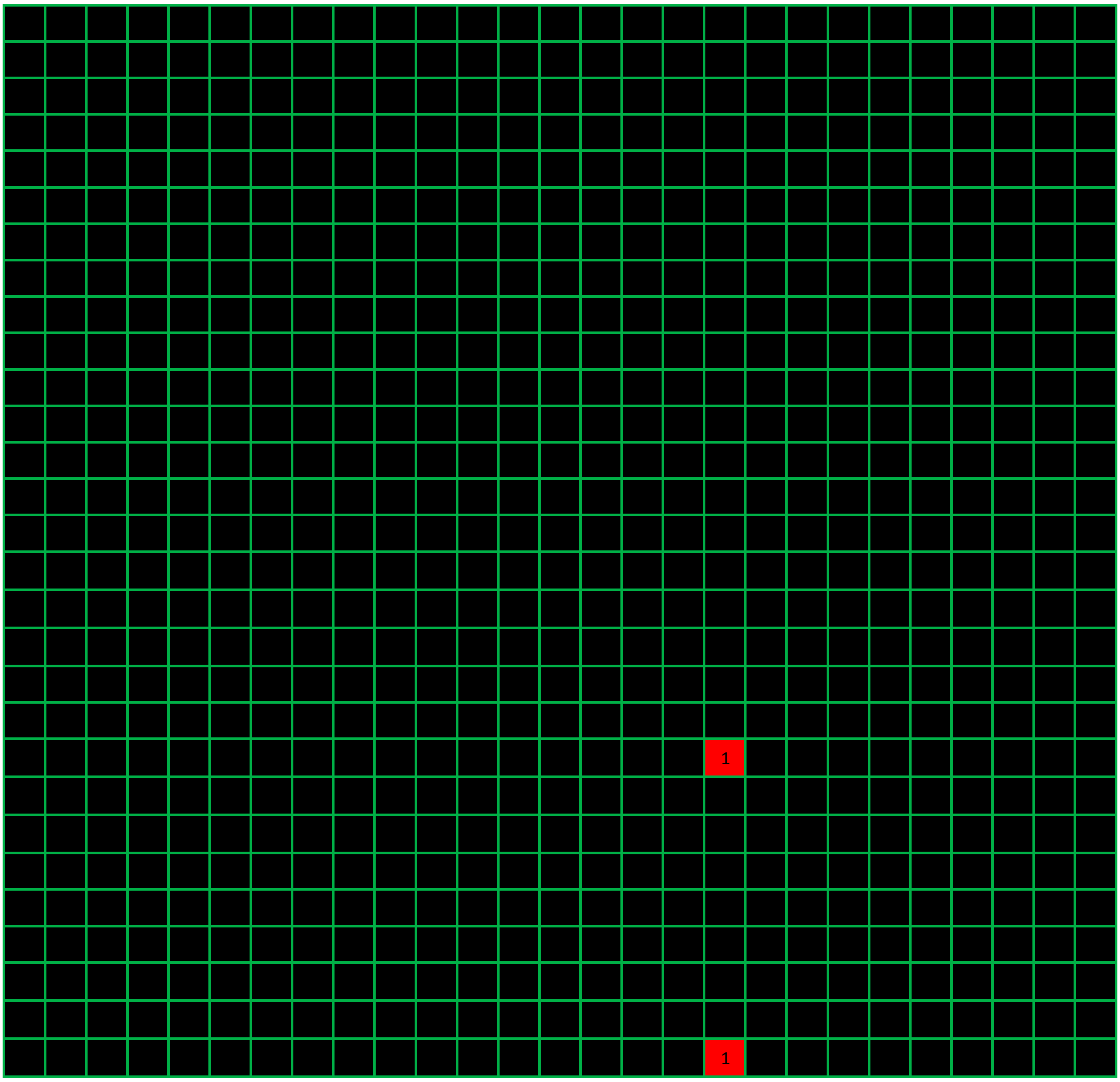
1
1

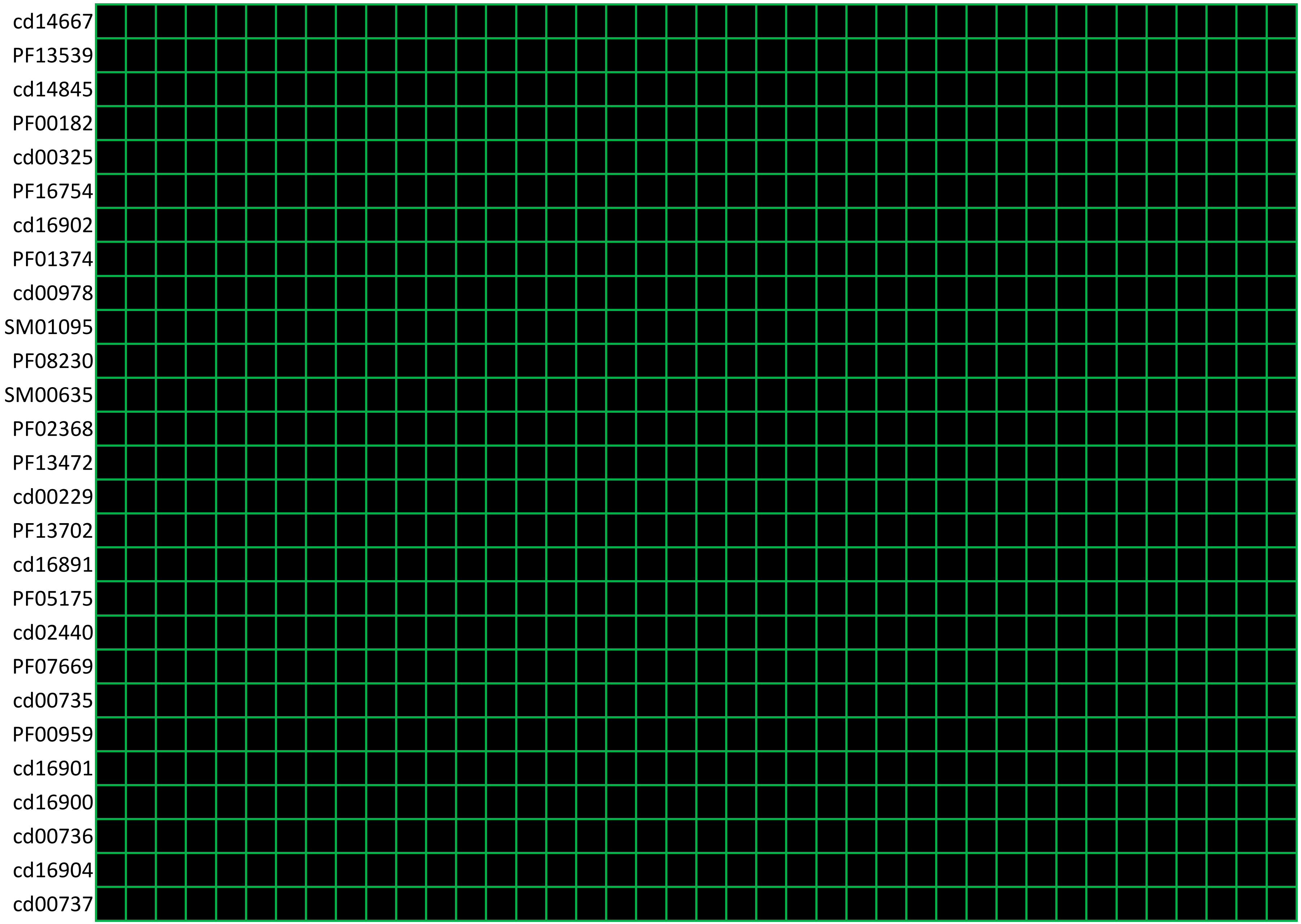
cd14667
PF13539
cd14845
PF00182
cd00325
PF16754
cd16902
PF01374
cd00978
SM01095
PF08230
SM00635
PF02368
PF13472
cd00229
PF13702
cd16891
PF05175
cd02440
PF07669
cd00735
PF00959
cd16901
cd16900
cd00736
cd16904
cd00737

						1																				
3																										
349	*1*				268																					
1	1																									
		130			70																					
			282		239																					
				73	64																					
268		70	239	64	###	362																				
					362	422																				
							3	1																		
							1	3	2																	
								2	2																	
										21	21															
										21	21															
												42	42													
												42	42													
														2	2											

														2	4											
																136	136									
																136	136									
																		7	7							
																		7	7							
																				6	6					
																				6	6					
																						473	197			
																						197	202			
																								380	372	
																								372	428	
																										14
																										7

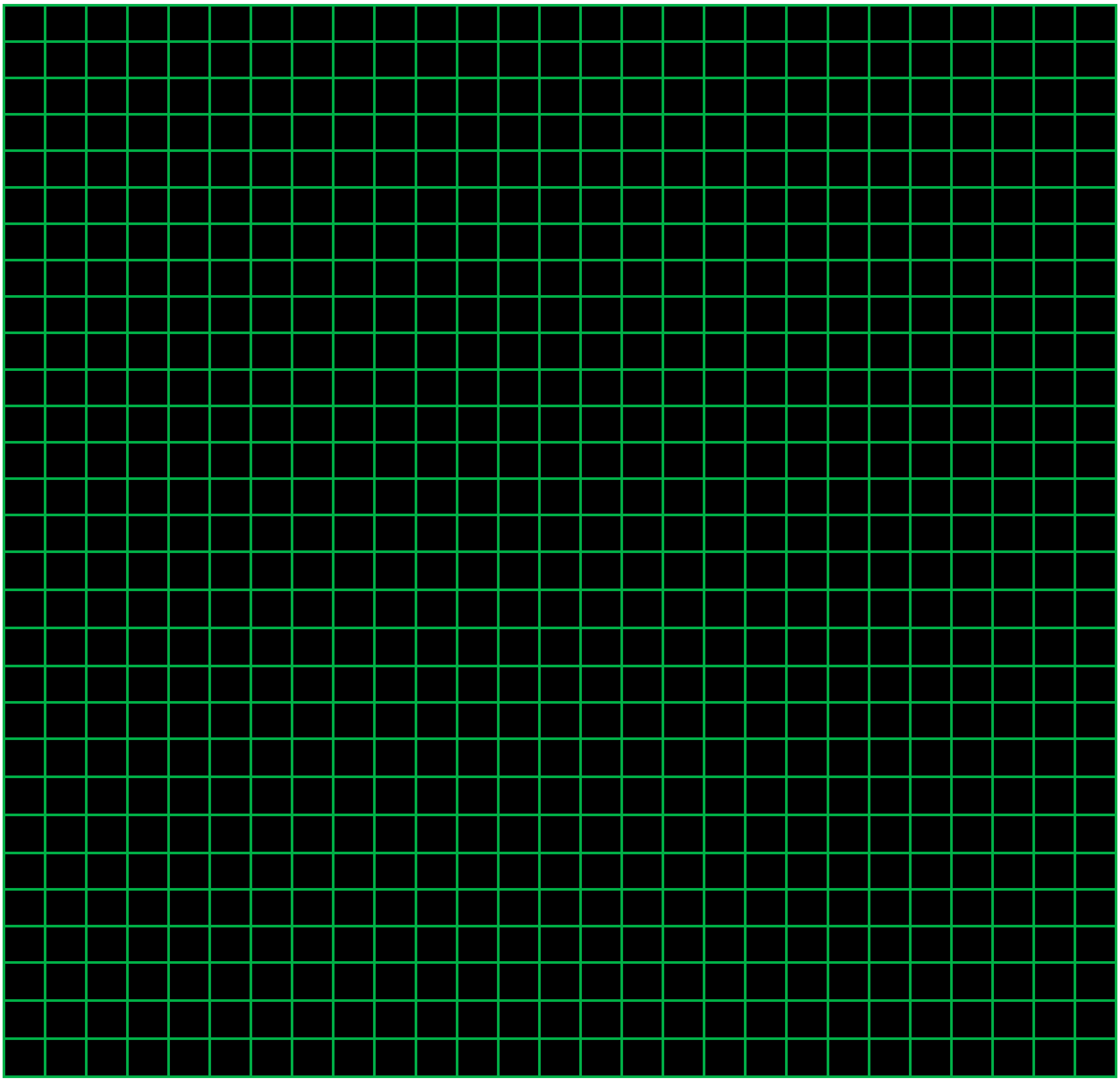

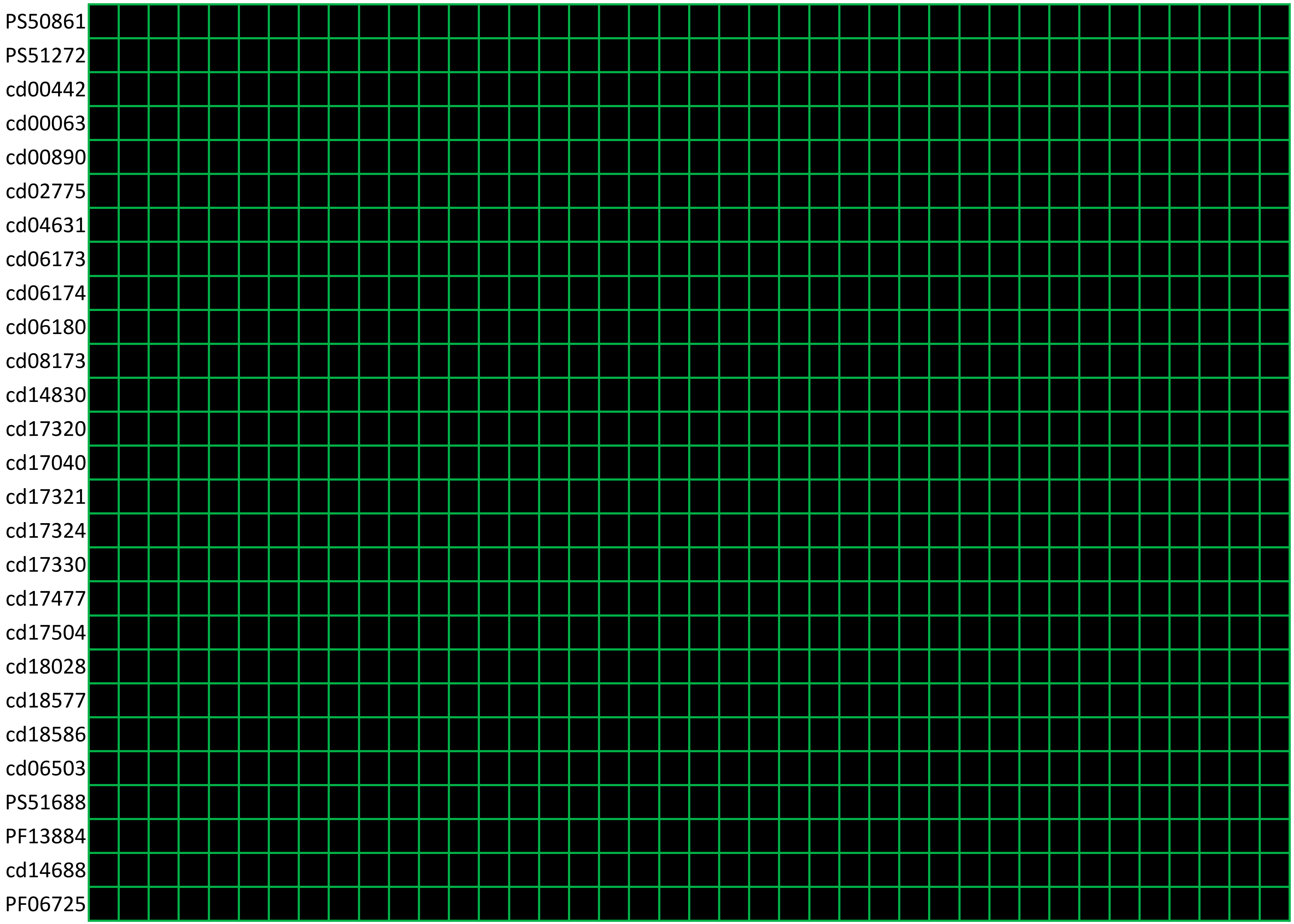
PS50861
PS51272
cd00442
cd00063
cd00890
cd02775
cd04631
cd06173
cd06174
cd06180
cd08173
cd14830
cd17320
cd17040
cd17321
cd17324
cd17330
cd17477
cd17504
cd18028
cd18577
cd18586
cd06503
PS51688
PF13884
cd14688
PF06725

7																										
7																										
	1		1																							
		31	31																							
	1	31	43																							
				70																						
					2																					
						1																				
							1																			
								1																		
									3																	
										1																
											1															
												2														
													1													
														7												
															12											
																1										
																	2									
																		3								
																			3							
																				1						
																					1					
																						8				
																							1			
																								1		
																									1	
																										1

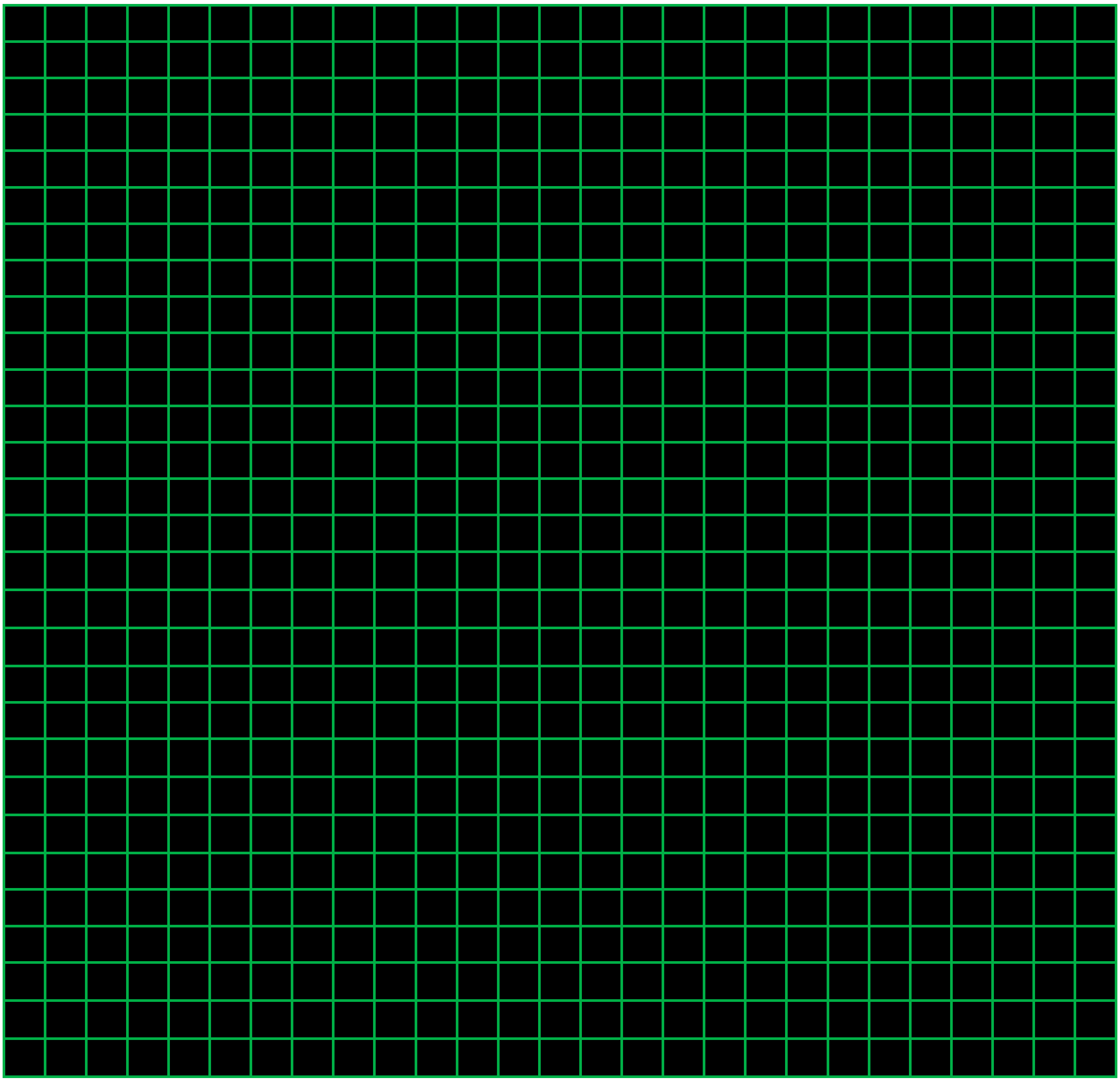

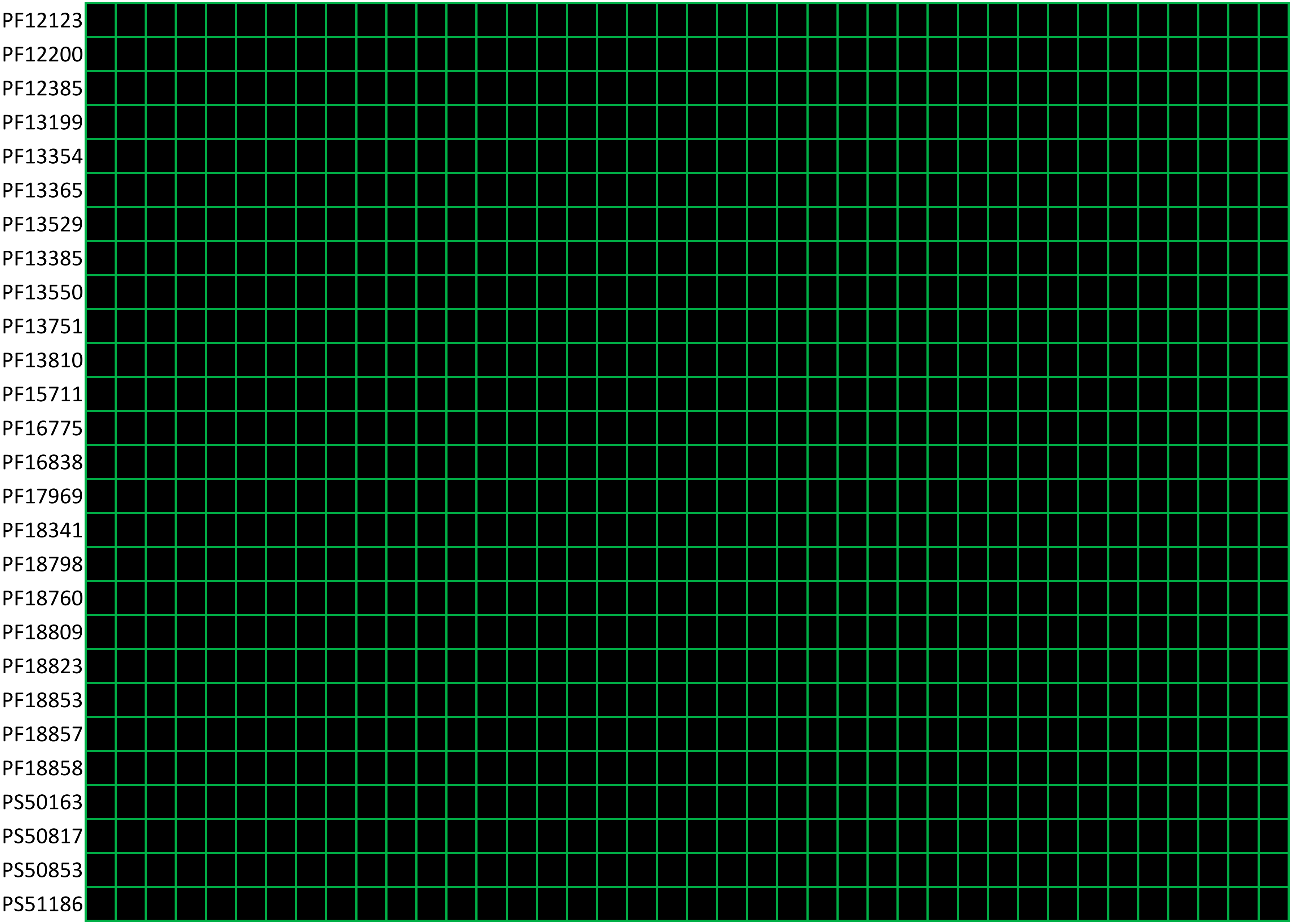
PF12123
PF12200
PF12385
PF13199
PF13354
PF13365
PF13529
PF13385
PF13550
PF13751
PF13810
PF15711
PF16775
PF16838
PF17969
PF18341
PF18798
PF18760
PF18809
PF18823
PF18853
PF18857
PF18858
PS50163
PS50817
PS50853
PS51186

1
4
1
8
7

					5																					
						2																				
							3																			
								2																		
									1																	
										5																
											17															
												1														
													3													
														186												
															8											
																3										
																	1									
																		1								
																			1							
																				156						
																					1					
																						1				
																							1			
																								19		
																									6	
																										31

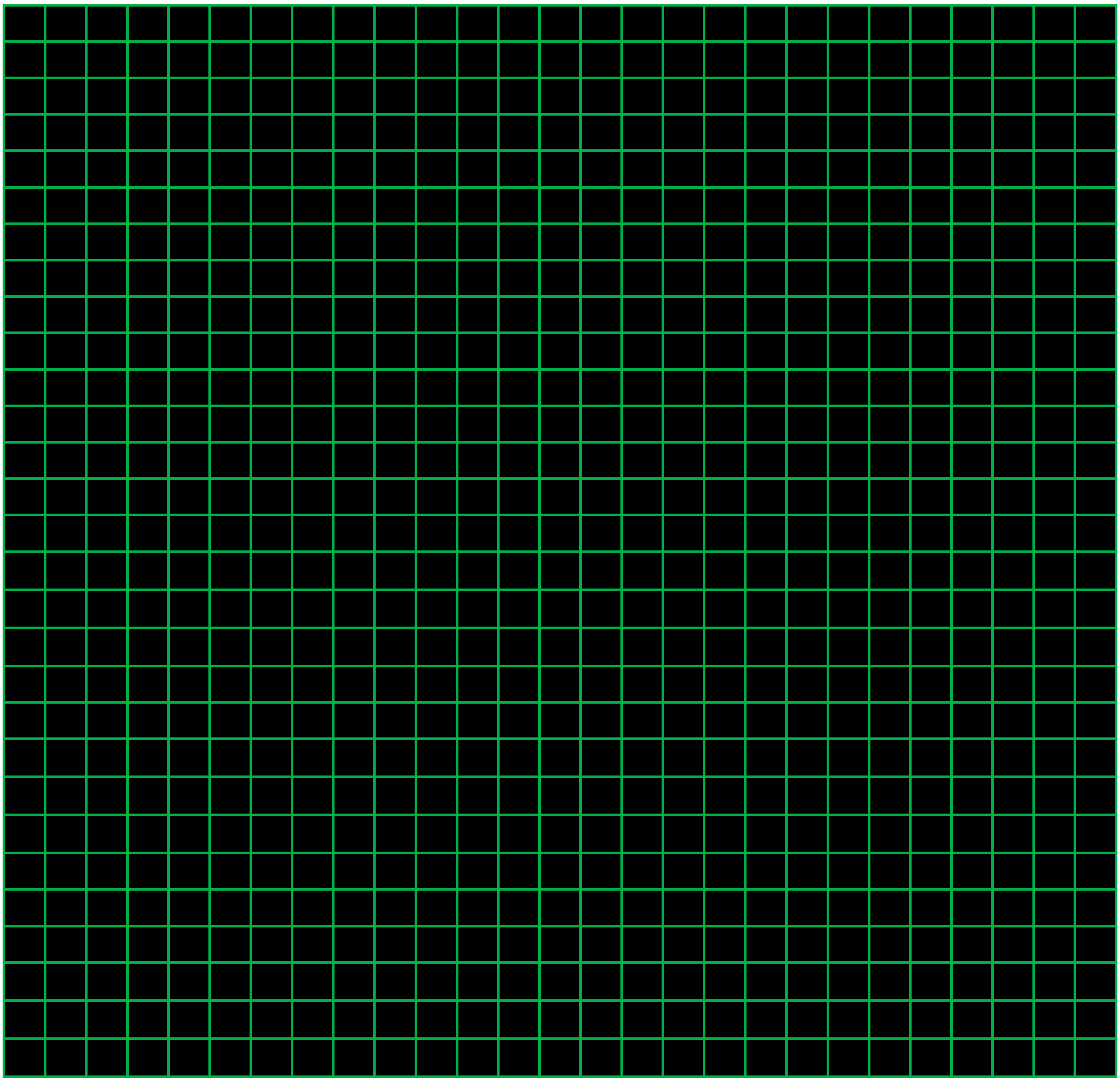

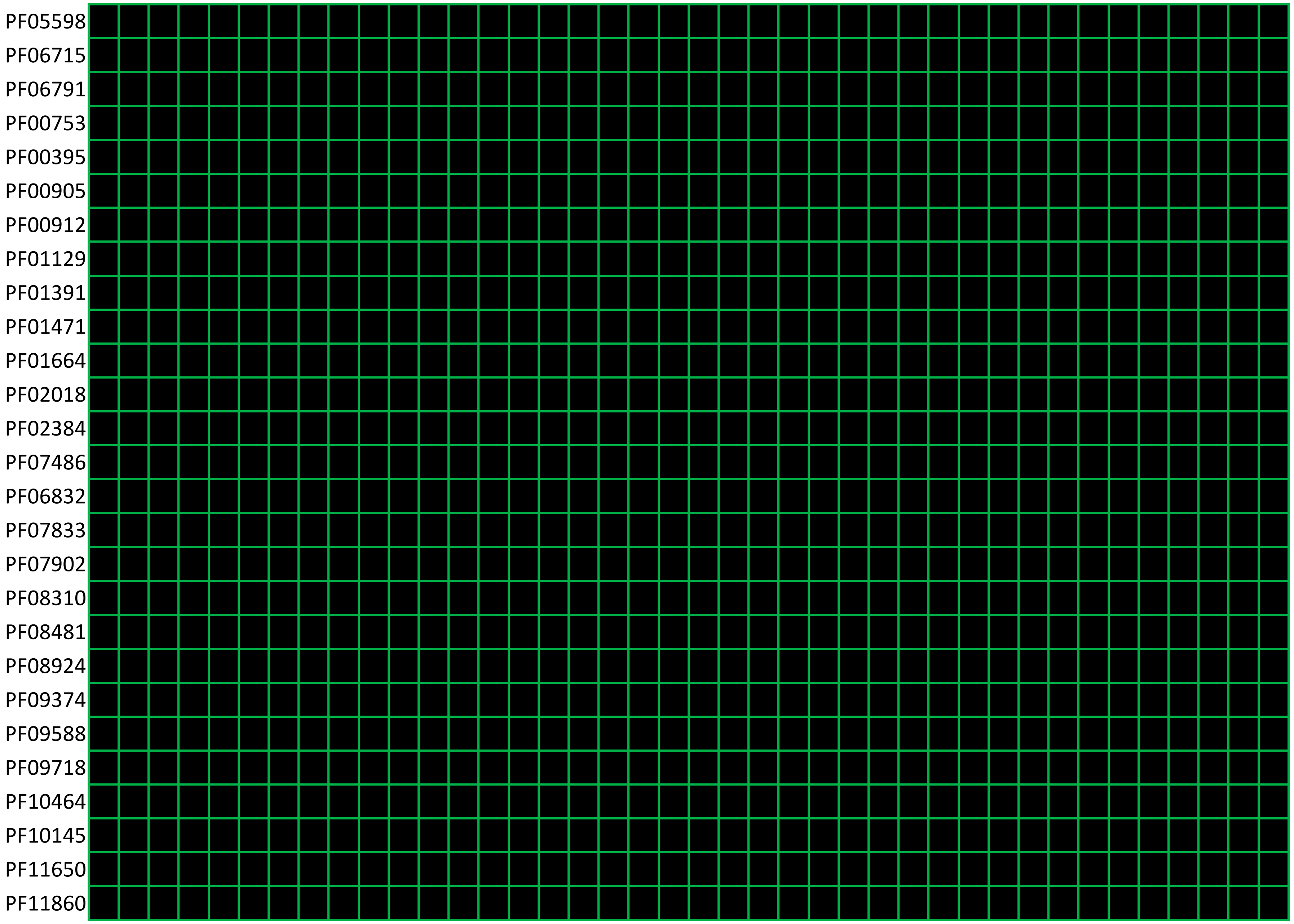
PF05598
PF06715
PF06791
PF00753
PF00395
PF00905
PF00912
PF01129
PF01391
PF01471
PF01664
PF02018
PF02384
PF07486
PF06832
PF07833
PF07902
PF08310
PF08481
PF08924
PF09374
PF09588
PF09718
PF10464
PF10145
PF11650
PF11860

90																										
	2																									
		214																								
			37																							
				2																						
					6																					
						157																				
							23																			
								1																		
									220																	
										31																
											1															
												1														
													32													
														1												
															2											
																1										
																	339									
																		36								
																			1							
																				1						
																					1					

																						1				
																							1			
																								6		
																									230	
																										1

PS50911																																								
PF05257																																								
SM00287																																								
PS51781																																								
PF08460																																								
PF08239																																								
SM00047																																								
PF01832																																								
PS51257																																								
PS51170																			6																					
PF01473																																								
PF01551																																								
PF00877																																								
PF14464																																								
cd08073																																								
SM00232																																								
PS50249																																								
PF04466																																								
PF03330																																								
PF04965																																								
PF05106																																								
PF05203																																								
PF05382																																								
PF05435																																								

														1								1

4																							
	231																						
		1																					
			1																				
				1																			
					2																		
						1																	
							84	48	83	80	*81*												
							48	77	77	77	*61*												
							83	77	185	179													
							80	77	179	183													
							81	*61*			469												
												499											
													548	548									
													548	662								*1*	*1*
															23								*1*
																403	371						
																371	371						
																		32		21	22		
																			321	99	240		
																		21	99	155	115	*1*	*1*
																		22	240	115	290		
														1						*1*		634	512
														1	*1*					*1*		512	657
