# Supplementary material for: PhaLP: A Database for the Study of Phage Lytic Proteins and Their Evolution
Source: Viruses. 2021 Jun 26;13(7):1240. doi: 10.3390/v13071240 (PMC8310338; doi:10.3390/v13071240)
Supplement: Supplementary file 1 [file viruses-13-01240-s001.zip › S2 File.pdf]

Class Name	Name
Actinobacteria	Corynebacteriales
	Micrococcales
	Propionibacteriales
	Streptomycetales
Cyanophyceae	Synechococcales
Bacilli	Bacillales
	Lactobacillales
Gammaproteobacteria	Aeromonadales
	Enterobacterales
	Pseudomonadales
	Vibrionales

Order
Rule
[GH19|PET_M23|DUF1906][Ami_2|PET_C39|GH25]{0,1}[LGFP|PG_1|RECA_3]{0,1}
[PET_M15|PET_M23|LysM][LysM|CW_7]{0,1}[Ami_2|PG_1]{0,1}
[Ami_2][PG_1]
[Ami_2|SLT_related|CHAP][SLT_related|LysM|PG_1]
[YkuD|PET_C70|PET_C39][YkuD|GH24]{0,1}[GLUCO|GH19]{0,1}
[Ami_2|SH3|Ami_3][CHAP|LysM|Ami02_C]{0,1}[SH3|PG_1|PSA_CBD]{0,1}
[CHAP|GH25|Ami_5][Ami_2|CW_1|SH3]{0,1}[LysM|ZoocinA_TRD|CW_7]{0,1}
[PET_M15|GH108|GH19][GH24]{0,1}[PG_3]{0,1}
[GH108|MUR|NUDIX][GH24]{0,1}[PG_3]{0,1}
[SLT_related|GH108|MUR][PG_3]{0,1}[AA_TRNA_LIGASE_II_GLYAB]{0,1}
[GH108|Pesticin_C|NLPC_P60][GH108]{0,1}[PG_3]{0,1}

F-score	Support	Name
		**Gordoniaceae**
0.65217	1743	**Mycobacteriaceae**
		**Nocardiaceae**
		**Microbacteriaceae**
0.47972	312	
		**Micrococcaceae**
0.37500	127	**Propionibacteriaceae**
0.57576	176	**Streptomycetaceae**
0.56962	147	**Synechococcaceae**
		**Bacillaceae**
0.59119	617	
		**Listeriaceae**
		**Paenibacillaceae**
		**Staphylococcaceae**
		**Enterococcaceae**
0.58915	839	
		**Streptococcaceae**
0.34286	67	**Aeromonadaceae**
		**Enterobacteriaceae**
0.52941	1416	
		**Erwiniaceae**
		**Pectobacteriaceae**
		**Moraxellaceae**
0.47458	394	
		**Pseudomonadaceae**
0.42857	269	**Vibrionaceae**

Family

Rule	F-score
[PET_M23|DUF1906|LGFP][Ami_2|RECA_3]{0,1}[PET_M23|LGFP]{0,1}	0.64020
[PET_M15|GH19|PET_C39][GH19|Ami_2]{0,1}[PG_1|SLT_related]{0,1}	0.62687
[LGFP|PET_M23|PET_M15][LGFP|LysM]{0,1}[GH25|PG_1]{0,1}	0.46612
[PET_M15|CW_7][LysM|Ami_2|PG_1]	0.45714
[PET_M23|CHAP|GH25][LysM|Ami_2|PG_1]	0.51948
[Ami_2][PG_1]	0.37500
[Ami_2|SLT_related|CHAP][SLT_related|LysM|PG_1]	0.57576
[YkuD|PET_C70|PET_C39][YkuD|GH24]{0,1}[GLUCO|GH19]{0,1}	0.56962
[GH25|Ami_2|Ami_3][LysM|SH3|Ami02_C]{0,1}[PG_1|DUF3597|SPOR]{0,1}	0.53066
[Ami_2|PET_M15|Ami_3][PSA_CBD|SH3]	0.56604
[GLUCO|Ami_2][Ami_3|Cu_amine_oxidN1]	0.46154
[CHAP|Ami_2|Ami_3][CHAP|SH3|Ami_3]	0.54194
[Ami_2|Ami_5][ZoocinA_TRD|SH3]	0.42857
[CW_1|CHAP|Ami_5][Ami_2|CHAP|SH3]{0,1}[CW_1|GH25|ZoocinA_TRD]{0,1}	0.54248
[PET_M15|GH108|GH19][GH24]{0,1}[PG_3]{0,1}	0.34286
[GH108|MUR|NUDIX][GH24]{0,1}[PG_3]{0,1}	0.56250
[GH108|MUR|PROKAR_LIPOPROTEIN][GH108]{0,1}[PG_3]{0,1}	0.55102
[PG_1|GH108][MUR|PG_3]	0.50000
[GH108|PG_1][GH24|PG_3]	0.40000
[SLT_related|GH108|MUR][PG_3]{0,1}[AA_TRNA_LIGASE_II_GLYAB]{0,1}	0.50909
[GH108|Pesticin_C|NLPC_P60][GH108]{0,1}[PG_3]{0,1}	0.42857

Support	Name
392	**Gordonia**
1250	**Mycolicibacterium**
65	**Rhodococcus**
75	**Microbacterium**
234	**Arthrobacter**
127	**Cutibacterium**
176	**Streptomyces**
142	**Synechococcus**
276	**Bacillus**
50	**Listeria**
58	**Paenibacillus**
229	**Staphylococcus**
85	**Enterococcus**
	**Lactococcus**
648	**Streptococcus**
67	**Aeromonas**
	**Esccherichia**
1169	**Klebsiella**
	**Salmonella**
	**Shigella**
73	**Erwinia**
84	**Pectobacterium**
95	**Acinetobacter**
299	**Pseudomonas**
269	**Vibrio**

Genus

Rule	F-score
[PET_M23|DUF1906|LGFP][Ami_2|RECA_3]{0,1}[PET_M23|LGFP]{0,1}	0.64020
[PET_M15|GH19|PET_C39][GH19|Ami_2]{0,1}[PG_1|SLT_related]{0,1}	0.62687
[LGFP|PET_M23|PET_M15][LGFP|LysM]{0,1}[GH25|PG_1]{0,1}	0.46612
[CW_7|PET_M15][LysM|Ami_2|PG_1]	0.50000
[CHAP|GH25|Ami_2][LysM|Ami_2|CW_7]	0.53125
[Ami_2]	0.12834
[Ami_2|SLT_related|CHAP][SLT_related|LysM|PG_1]	0.57576
[YkuD|PET_C70|PET_C39][YkuD|GH24]{0,1}[GLUCO|GH19]{0,1}	0.56962
[GH25|Ami_2|Ami_3][LysM|SH3|Ami02_C]{0,1}[PG_1|DUF3597|SPOR]{0,1}	0.53066
[Ami_2|PET_M15|Ami_3][PSA_CBD]	0.54545
[Ami_2][Cu_amine_oxidN1]	0.40000
[CHAP|Ami_2|Ami_3][CHAP|SH3|Ami_3]	0.54194
[Ami_2|Ami_5][ZoocinA_TRD|SH3]	0.42857
[Ami_2|CHAP][SH3]	0.35821
[CW_1|CHAP|Ami_5][Ami_2|CHAP|SH3]{0,1}[CW_1|GH25|ZoocinA_TRD]{0,1}	0.55333
[PET_M15|GH108|GH19][GH24]{0,1}[PG_3]{0,1}	0.34286
[GH108|MUR|NUDIX][GH24]{0,1}[PG_3]{0,1}	0.46667
[GH19|GH108|MUR][GH24]{0,1}[PG_3]{0,1}	0.42424
[PG_1|GH108][MUR|PG_3]	0.44444
[GH108|PG_1][PG_3|MUR]	0.57143
[GH108|MUR|PROKAR_LIPOPROTEIN][GH108]{0,1}[PG_3]{0,1}	0.48837
[PG_1|GH108][MUR|PG_3]	0.50000
[GH108|PG_1][GH24|PG_3]	0.40000
[SLT_related|GH108|MUR][PG_3]{0,1}[AA_TRNA_LIGASE_II_GLYAB]{0,1}	0.50909
[GH108|Pesticin_C|NLPC_P60][GH108]{0,1}[PG_3]{0,1}	0.42857

Support	Name
391	***Gordonia terrae***
1212	***Mycolicibacterium smegmatis***
62	***Rhodococcus erythropolis***
71	***Microbacterium foliorum***
230	***Arthrobacter globiformis***
	Arthrobacter sp. ATCC 21022
113	***Cutibacterium acnes***
176	***Streptomyces griseus***
142	***Synechococcus sp.***
	Synechococcus sp. WH 7803
268	***Bacillus cereus***
	Bacillus subtilis
	Bacillus thuringiensis
47	***Listeria monocytogenes***
49	***Paenibacillus larvae***
229	***Staphylococcus aureus***
85	***Enterococcus faecalis***
262	***Lactococcus lactis***
386	***Streptococcus agalactiae***
	Streptococcus dysgalactiae
	Streptococcus pneumoniae
	Streptococcus pyogenes
	Streptococcus suis
67	***Aeromonas salmonicida***
566	***Esccherichia coli***
174	***Klebsiella pneumoniae***
289	***Salmonella enterica***
65	***Shigella flexneri***
60	***Erwinia amylovora***
57	***Pectobacterium atrosepticum***
94	***Acinetobacter baumannii***
299	***Pseudomonas aeruginosa***
266	***Vibrio breoganii***
	Vibrio cholerae
	Vibrio cyclitrophicus
	Vibrio lentus

Species

Rule	F-score
[PET_M23|LGFP|DUF1906][Ami_2|PET_M23]{0,1}[LGFP|RECA_3]{0,1}	0.61856
[PET_M15|GH19|PET_C39][GH19|Ami_2]{0,1}[PG_1|SLT_related]{0,1}	0.62687
[PET_M23|Ami_2|PET_M15][GH25|LGFP|DUF4185]	0.47312
[CW_7|PET_M15][LysM|Ami_2|PG_1]	0.50000
[CHAP|GH25][LysM]	0.54545
[Ami_2|CHAP][Ami_2|CW_7|GH25]	0.43243
[Ami_2]	0.12834
[Ami_2][Ami_2|LysM][PG_1|SLT_related]	0.44444
[GH24|PROKAR_LIPOPROTEIN|PET_C39][GH24|SLT_related|PET_M15]	0.46154
[GH24|PET_C39|NLPC_P60][GH19]{0,1}[PET_M15]{0,1}	0.54545
[GH25|Ami_3|Ami_2][Ami02_C|SH3]	0.56250
[GH25|Ami_2|GH24][LysM|DUF3597]	0.44444
[GH25|Ami_2|Ami_3][Ami02_C|SH3|SPOR]	0.53846
[Ami_2|PET_M15|Ami_3][PSA_CBD]	0.66667
[Ami_3]	0.25532
[CHAP|Ami_2|Ami_3][SH3|Ami_3|GLUCO]	0.61314
[Ami_2|Ami_5][ZoocinA_TRD|SH3]	0.42857
[Ami_2|CHAP][SH3]	0.39344
[GLUCO|Ami_3][CHAP|Ami_3|LysM][CHAP|LysM|SH3]	0.75000
[GLUCO|Ami_3|Ami_5][CHAP|LysM|CW_7][GLUCO|CHAP|SH3]	0.60000
[Ami_2|GH25|Ami_5][CW_1]	0.66667
[CHAP|Ami_5][SH3|CW_7|GH25]	0.56790
[Ami_3|Ami_5][LysM|CW_7][CHAP|GLUCO]	0.57143
[SLT_related|PET_M15|GH19]	0.31579
[GH108|MUR|NUDIX][GH24]{0,1}[PG_3]{0,1}	0.46667
[GH19|GH108|MUR][SLT_related]{0,1}[PG_3]{0,1}	0.42424
[GH108|PG_1][PG_3|MUR]	0.44444
[GH24]	0.24000
[GH108|PROKAR_LIPOPROTEIN|MUR][GH108]{0,1}[PG_3]{0,1}	0.48837
[PET_M15|GH24]	0.30000
[GH108|PG_1][GH24|PG_3]	0.40000
[PROKAR_LIPOPROTEIN|GH108|PG_1][SLT_related|MUR|PG_3]	0.50000
[PET_M15]	0.26667
[GH108][PG_3]	0.33333
[GH108][PG_3]	0.50000
[GH108|PET_M15|SLT_related][GH108]{0,1}[PG_3]{0,1}	0.31579

Support
359
1208
50
57
30
189
113
76
53
67
82
25
99
41
47
162
72
255
27
26
94
58
27
37
551
160
264
38
52
34
78
220
30
47
34
29
