# Supplementary material for: PhaLP: A Database for the Study of Phage Lytic Proteins and Their Evolution
Source: Viruses. 2021 Jun 26;13(7):1240. doi: 10.3390/v13071240 (PMC8310338; doi:10.3390/v13071240)
Supplement: Supplementary file 1 [file viruses-13-01240-s001.zip › S3 File.pdf]

Accession	Index	Architecture
G8IDH4	2729	['Ami_2']
A0A346N934	2730	['Ami_2']
A0A411CJS4	2731	['Ami_2']
A0A0A0YSC3	2732	['Ami_2']
A0A411CMX7	2733	['Ami_2']
A0A411CCV4	2734	['Ami_2']
A0A411CH49	2735	['Ami_2']
A0A411CFG6	2736	['Ami_2']
A0A411AZJ8	2737	['Ami_2']
A0A3G8FNF9	2738	['Ami_2']
A0A3G3BZH6	2739	['Ami_2']
A0A346FDZ5	2740	['Ami_2']
A0A411C1Y0	2741	['Ami_2']
A0A2D1GIG5	2742	['Ami_2']
A0A411CC32	2743	['Ami_2']
A0A1D8EYF9	2744	['Ami_2']
A0A0K1Y763	2745	['Ami_2']
G1BLA0	2746	['Ami_2']
V5UQ97	2747	['Ami_2']
G1BPF4	2748	['Ami_2']
A0A481VQV4	2749	['Ami_2']
T2A8A7	2750	['Ami_2']
A0A345KWK5	2751	['Ami_2']
B3VLX1	2752	['Ami_2']
G8I9G3	2753	['Ami_2']
A0A0F6WEB5	2754	['Ami_2']
Q1A072	2755	['Ami_2']
T2A7Y9	2756	['Ami_2']
A0A514DFT8	2757	['Ami_2', 'LGFP']
A0A142KA09	2758	['Ami_2', 'LGFP']
A0A3G8FFL9	2759	['Ami_2', 'LGFP']
A0A3G8FEN5	2760	['Ami_2', 'LGFP']
A0A345KRY4	2761	['Ami_2', 'LGFP']
A0A142K8I6	2762	['Ami_2', 'LGFP']
A0A4Y6EHX7	2763	['Ami_2', 'LGFP']
A0A2Z4Q3T0	2764	['Ami_2', 'LGFP']
A0A3S9URD9	2765	['Ami_2', 'LGFP']
A0A3S9UR36	2766	['Ami_2', 'LGFP']
A0A4Y5NZK9	2767	['Ami_2', 'LGFP']
A0A2U8UKC2	2768	['Ami_2', 'LGFP']
A0A4D6T682	2769	['Ami_2', 'LGFP']
A0A162E1U7	2770	['Ami_2', 'LGFP', 'LGFP', 'LGFP']
A0A346FD11	2771	['Ami_2', 'LGFP', 'LGFP', 'LGFP']
A0A4D6E276	2772	['Ami_2', 'LGFP', 'LGFP']
A0A514U119	2773	['Ami_2', 'LGFP', 'LGFP']

A0A515MH83	2774	['Ami_2', 'LGFP', 'LGFP', 'LGFP']
Q856D3	2775	['Ami_2', 'LGFP', 'LGFP']
A0A3S9UQK1	2776	['Ami_2', 'LGFP', 'LGFP']
A0A2P1A0T5	2777	['Ami_2', 'LGFP', 'LGFP', 'LGFP']
A0A516KPP4	2778	['Ami_2', 'LGFP', 'LGFP', 'LGFP']
A0A159B6C4	2779	['Ami_2', 'LGFP', 'LGFP', 'LGFP']
A0A1B3AZZ3	2780	['Ami_2', 'LGFP', 'LGFP', 'LGFP']
A0A3Q9R8M1	2781	['Ami_2', 'LGFP', 'LGFP', 'LGFP']
A0A411CUP2	2782	['Ami_2', 'LGFP', 'LGFP', 'LGFP']
A0A3S9UAG8	2783	['Ami_2', 'LGFP', 'LGFP', 'LGFP']
A0A2K9VH40	2784	['Ami_2', 'LGFP', 'LGFP', 'LGFP']
A0A4Y6EGW4	2785	['Ami_2', 'LGFP', 'LGFP', 'LGFP']
A0A2U8UNQ7	2786	['Ami_2', 'LGFP', 'LGFP', 'LGFP']
A0A514DIC5	2787	['Ami_2', 'LGFP', 'LGFP', 'LGFP']
A0A2U8UKP6	2788	['Ami_2', 'LGFP', 'LGFP']
A0A4Y6EFS0	2789	['Ami_2', 'LGFP', 'LGFP']
A0A2Z4Q8W9	2790	['Ami_2', 'LGFP', 'LGFP', 'LGFP']
A0A1B3B1F8	2791	['Ami_2', 'LGFP']
A0A2Z4Q823	2792	['Ami_2', 'LGFP']
A0A2H4PEH7	2793	['Ami_2']
A0A0K2FNX7	2794	['Ami_2']
Q856M5	2795	['Ami_2']
A0A3G3LYI7	2796	['Ami_2']
S5Y3I1	2797	['Ami_2']
A0A2P1N4E4	2798	['Ami_2']
G8I449	2799	['Ami_2']
A0A345MF39	2800	['Ami_2']
A0A2Z5H6D8	2801	['Ami_2']
A0A385DWR7	2802	['Ami_2']
R4JPZ6	2803	['Ami_2']
A0A2S1PB03	2804	['Ami_2']
A0A2P1JY73	2805	['PET_M23', 'Ami_2']
A0A514CXM0	2806	['PET_M23', 'Ami_2']
A0A410TCC5	2807	['PET_M23', 'Ami_2']
A0A410TBB4	2808	['PET_M23', 'Ami_2']
A8WA19	2809	['PET_M23', 'Ami_2', 'PG_1']
A0A385UEY8	2810	['PET_M23', 'Ami_2', 'PG_1']
A0A385D1U0	2811	['PET_M23', 'Ami_2', 'PG_1']
A0A0H4THJ6	2812	['PET_M23', 'Ami_2', 'PG_1']
A0A2D1GEV9	2813	['PET_M23', 'Ami_2', 'PG_1']
A0A386KGY7	2814	['PET_M23', 'Ami_2', 'PG_1']
A0A482JAE8	2815	['PET_M23', 'Ami_2', 'PG_1']
W8ED04	2816	['PET_M23', 'Ami_2', 'PG_1']
A0A1D8EVX2	2817	['PET_M23', 'Ami_2', 'PG_1']
Q19Y11	2818	['GH19', 'Ami_2']
A0A0B5A4W2	2819	['GH19', 'Ami_2']

A0A2I2MPE9	2820	['GH19', 'Ami_2']
G8I4E0	2821	['GH19', 'Ami_2']
Q855S8	2822	['GH19', 'Ami_2']
I3WWS1	2823	['GH19', 'Ami_2']
A0A385DN04	2824	['Ami_2']
A0A2P1JR63	2825	['Ami_2']
A0A0K2FMY5	2826	['Ami_2']
A0A0K2FNU8	2827	['Ami_2']
A0A2D1GA73	2828	['Ami_2']
A0A2S1PCZ0	2829	['Ami_2']
A0A385UK50	2830	['Ami_2']
A0A385UKB6	2831	['Ami_2']
A0A192YBS4	2832	['Ami_2']
A0A411AYD0	2833	['Ami_2']
A0A3G8FHF6	2834	['Ami_2']
A0A023ZWD2	2835	['Ami_2']
A0A249XT90	2836	['Ami_2']
A0A0A0RKX1	2837	['Ami_2']
B5U576	2838	['Ami_2']
A0A2U8UMJ2	2839	['Ami_2']
A0A0U4IDK0	2840	['Ami_2', 'LysM', 'LysM']
A0A3G2KF58	2841	['Ami_2']
A0A3G2KFA3	2842	['Ami_2']
A0A3G2KET2	2843	['Ami_2']
A0A3G2KIR8	2844	['Ami_2']
A0A3G2KCX9	2845	['Ami_2']
A0A3G2KH20	2846	['Ami_2']
A0A0K0N6L3	2847	['Ami_2', 'LGFP', 'LGFP']
A0A2D1A400	2848	['Ami_2', 'LGFP', 'LGFP', 'LGFP', 'LGFP']
A0A2P1CC23	2849	['Ami_2']
A0A2Z4Q5C9	2850	['Ami_2']
A0A4D6E3J8	2851	['Ami_2']
A0A2H4J9K9	2852	['Ami_2']
D4P7C1	2853	['Ami_2']
D4P7N0	2854	['Ami_2']
A0A455LM17	2855	['Ami_2']
A0A346FC93	2856	['Ami_2']
A0A386KB78	2857	['Ami_2']
A0A3G3M0R1	2858	['Ami_2']
A0A0M5M0S0	2859	['Ami_2']
A0A3G2KDX8	2860	['Ami_2']
Q9ZX49	2861	['Ami_2']
R4JD41	2862	['Ami_2']
A0A0E3X9U3	2863	['Ami_2']
A0A0E3T6F9	2864	['Ami_2']
A0A0K0NKI0	2865	['Ami_2']

A0A1B3B0M4	2866	['Ami_2']
A0A166Y6M9	2867	['Ami_2']
A0A142KCB0	2868	['Ami_2']
A0A482JCA2	2869	['Ami_2']
G8FS21	2870	['Ami_2']
A0A0K0N5F1	2871	Unknown
A0A0K0N5D8	2872	Unknown
A0A166Y5E2	2873	['Ami_2']
A0A411B2T5	2874	['Ami_2']
A0A514A5Z4	2875	['Ami_2']
A0A411B1T4	2876	['Ami_2']
A0A2P1N5E0	2877	['Ami_2']
A0A482JIE1	2878	['Ami_2']
A0A2K9VED9	2879	['Ami_2']
A0A4Y6EHN5	2880	['Ami_2']
A0A2K9VDJ1	2881	['Ami_2']
A0A411BQU5	2882	['Ami_2']
A0A515MJL0	2883	['Ami_2']
A0A166Y601	2884	['Ami_2']
A0A4Y6EQL6	2885	['Ami_2']
A0A385IKI2	2886	['GH19']
A0A0U3E1L6	2887	['GH19']
A0A514CTD2	2888	Unknown
R9ZZL3	2889	['PG_1', 'GH19']
A0A385A117	2890	['GH19', 'PG_1']
A0A067XQI3	2891	Unknown
A0A0K1LLX7	2892	Unknown
A0A1P8VVH0	2893	['GH19']
A0A2R3UA80	2894	['GH19']
A0A023NGE0	2895	['GH19']
A0A2U7NMW4	2896	['GH19']
A0A2D2W2B5	2897	['GH19']
A0A076G6J8	2898	['GH19', 'PG_1']
A0A291AUV5	2899	['GH19', 'PG_1']
G9I9L2	2900	['GH19']
A0A4Y5TM42	2901	['GH19']
A0A482IHJ0	2902	['PET_C39', 'GH19']
A0A482IAM1	2903	['GH19']
A0A510E8H5	2904	['GH19']
M4T440	2905	Unknown
G8EYC4	2906	['GH19']
Q8W6X9	2907	['GH19']
A0A3G1GLF3	2908	['GH19']
A0A3S7I6E5	2909	['GH19']
A0A3S7HFC5	2910	['GH19']
Q2NPA7	2911	['GH19']

A0A3S7HPK5	2912	['GH19']
A0A3S7I6N4	2913	['GH19']
A0A4Y5TZN0	2914	['GH19']
A0A514A7K5	2915	['GH19']
G3BM11	2916	['GH19']
G3BM12	2917	['GH19']
A0A3G8F0Y3	2918	['GH19']
A0A2H4YGM8	2919	['GH19']
K4F6U2	2920	['GH19']
V5KSW7	2921	['GH19']
A0A2I6PHU8	2922	['GH19']
A0A481W7S9	2923	['GH19']
B5TK83	2924	['GH19']
A0A2H4JHX4	2925	['GH19']
A0A059VA40	2926	['GH19']
A0A345BNC6	2927	['GH19']
A0A7I3	2928	['GH19']
A0A075BS11	2929	['GH19']
A0A219VHE4	2930	['GH19', 'PG_1']
A0A346FDB4	2931	['GH19']
A0A2H4JAK8	2932	['GH19']
A0A2H4JDB4	2933	['GH19']
A0A2H4J3F6	2934	['GH19']
Q5ZGC9	2935	['GH19']
A0A0B5A729	2936	['GH19']
A0A193GYJ3	2937	['GH19']
A0A0F6R8M1	2938	['GH19']
G9L6E8	2939	['GH19']
A0A2I6TCF1	2940	['GH19']
Q858F0	2941	['GH19']
M1F227	2942	['GH19']
F1C591	2943	['GH19']
K7PJS7	2944	['GH19']
A0A482J1N9	2945	['GH19']
A0A2Z2FAL4	2946	['GH19']
A0A482MFC8	2947	['GH19']
A0A482MF60	2948	['GH19']
A0A482IL61	2949	['GH19']
A0A2Z2FBK1	2950	['GH19']
G8C7W0	2951	['GH19']
A0A2Z5H035	2952	['GH19']
Q8HA86	2953	['GH19']
A0A192Y6G4	2954	['GH19']
A0A514A8T2	2955	['GH19']
A0A482MUT9	2956	['GH19']
I7ATQ5	2957	['GH19']

A0A494RAL2	2958	['GH19']
A0A386KJF2	2959	['GH19']
A0A482MV99	2960	['GH19']
A0A2H4P6U7	2961	['GH19']
A0A482MGV2	2962	['GH19']
A1YZZ8	2963	Unknown
A0A2S1GSQ6	2964	['GH19']
A0A3S7L8K0	2965	['GH19']
A0A481UM06	2966	['GH19']
A0A1B0Z086	2967	['GH19']
A0A0U4JP23	2968	['GH19']
A0A3S6RNH2	2969	['GH19']
A0A481URM7	2970	['GH19']
A0A1W6JTC9	2971	['GH19']
A0A481UMS2	2972	['GH19']
A0A481UPC1	2973	['GH19']
X5IEK4	2974	['GH19']
A0A0S3UG46	2975	['GH19']
A0A2H4J3Y3	2976	['GH19']
A0A2H4J3N1	2977	['GH19']
A0A345MV58	2978	['GH19']
A0A125RNP1	2979	['GH19']
J7I4M6	2980	['GH19']
A0A481UYN2	2981	['GH19']
A0A0M3LPP0	2982	['GH19']
Q7Y5V0	2983	['GH19']
D0UIK2	2984	['GH19']
A0A292GC73	2985	['GH19']
C9DFY3	2986	['GH19']
A0A2H4N7Z7	2987	['GH19']
F1C5D2	2988	['GH19']
A0A248XCW5	2989	['GH19']
A0A411EZQ3	2990	['GH19']
A0A2D1GNI0	2991	['GH19']
A0A411CU33	2992	['GH19']
A0A410TDJ6	2993	['GH19']
A0A385DS23	2994	['GH19']
A0A410TBH8	2995	['GH19']
A0A385DU67	2996	['GH19']
A0A2D2W4L6	2997	['GH19']
A0A4Y6EKA7	2998	['GH19']
A0A0K0N6C3	2999	['GH19']
A0A386KG61	3000	['GH19']
A0A345L4X6	3001	['GH19']
A0A386KFT4	3002	['GH19']
A0A2L1IZV9	3003	['GH19']

A0A410TE38	3004	['GH19']
A0A386KD63	3005	['GH19']
Q2TLS9	3006	['GH19']
G8EJQ6	3007	['GH19']
G8EJZ6	3008	['GH19']
A0A2L1IX84	3009	['GH19']
A0A1J0MDM1	3010	['PG_1', 'GH19']
A0A2Z5X8L4	3011	['PG_1', 'GH19']
A0A2P1JRH1	3012	['PG_1', 'GH19']
A0A482JFJ5	3013	['PG_1', 'GH19']
A0A3G3LWH1	3014	['GH19', 'PET_C39']
A0A4Y6EME5	3015	['GH19', 'PET_C39']
A0A2H4P8Q9	3016	['GH19', 'PET_C39']
A0A2H4P8D8	3017	['GH19', 'PET_C39']
A0A3G3MA99	3018	['GH19', 'PET_C39']
A0A2H4P918	3019	['GH19', 'PET_C39']
A7IYC3	3020	['GH19', 'PET_C39']
A0A514DKF1	3021	['GH19', 'PET_C39']
A0A4Y6ENE9	3022	['GH19', 'PET_C39']
A0A2H4PIY8	3023	['PET_M23']
A0A2H4PIV9	3024	['PET_M23']
A0A142K909	3025	['GH19']
A0A160DEU2	3026	['GH19']
A0A345L3D0	3027	['GH19']
A0A1C9EHQ1	3028	['PROKAR_LIPOPROTEIN', 'GH19']
A0A515MKD7	3029	['GH19']
A0A142KC32	3030	['GH19']
A0A345KW49	3031	['GH19']
A0A410TCT3	3032	['GH19']
A0A411AWR9	3033	['GH19']
A0A345L130	3034	['GH19']
A0A385E1X5	3035	['GH19']
A0A142F2G1	3036	['GH19']
A0A4Y6ETU2	3037	Unknown
A0A068CCD7	3038	['Ami_2']
B6DQH9	3039	['Ami_2']
J3RJW9	3040	['Ami_2']
A0A068CAR6	3041	['Ami_2']
Q38653	3042	['Ami_2']
A0A059T8T6	3043	['Ami_2']
A0A088FNU9	3044	['Ami_2']
M4GZZ1	3045	['Ami_2']
A0A060AC40	3046	['Ami_2']
A0A060AFE7	3047	['Ami_2']
A0A1B0Y4R9	3048	['Ami_2']
Q9MCC8	3049	['Ami_2']

Q9MCC6	3050	['Ami_2']
U5U4H7	3051	['Ami_2']
A0A0N7IR99	3052	['Ami_2']
A0A0P0I386	3053	['Ami_2']
A0A0P0IX40	3054	['Ami_2']
A6MAD8	3055	['Ami_2']
A0A1W6JKF0	3056	['Ami_2']
V9VHX3	3057	['Ami_2']
A0A0D3MSS4	3058	['Ami_2']
E0YIU2	3059	['Ami_2']
A0A1W6JLI9	3060	['Ami_2']
A0A1W6JM05	3061	['Ami_2']
Q38135	3062	['Ami_2']
A0A1W6JN27	3063	['Ami_2']
V5US95	3064	['Ami_2', 'ZoocinA_TRD']
A0A0S2MY87	3065	['Ami_2', 'ZoocinA_TRD']
C0LZY2	3066	['Ami_2', 'ZoocinA_TRD']
A0A4D6DT61	3067	['Ami_2', 'ZoocinA_TRD']
A0A411B6N5	3068	['Ami_2', 'ZoocinA_TRD']
A0A249XUP0	3069	['Ami_2', 'ZoocinA_TRD']
I7B7C3	3070	['Ami_2', 'ZoocinA_TRD']
A0A249XVK1	3071	['Ami_2', 'ZoocinA_TRD']
A0A2I7QHI7	3072	['Ami_2', 'ZoocinA_TRD']
A0A2S1GSK4	3073	['Ami_2', 'ZoocinA_TRD']
A0A1D3SNQ1	3074	['Ami_2', 'SH3']
A0A1G5SA77	3075	['Ami_2', 'SH3']
A0A4D6DUL4	3076	['Ami_2', 'SH3']
A0A2Z5H386	3077	['Ami_2', 'SH3']
A0A4D6DVX4	3078	['Ami_2', 'SH3']
A0A3S9U820	3079	['Ami_2', 'SH3']
A0A4D6DSH8	3080	['Ami_2', 'SH3']
A0A249XUE1	3081	['Ami_2', 'SH3']
A0A1B1PA07	3082	['Ami_2', 'SH3']
A0A139ZVY1	3083	['Ami_2', 'SH3']
A0A4D6DV21	3084	['Ami_2', 'SH3']
A0A2S1PFE2	3085	['Ami_2', 'SH3']
A0A2S1PF52	3086	['Ami_2', 'SH3']
A0A2Z4Q002	3087	['Ami_2', 'SH3']
A0A1S6L127	3088	['CHAP']
A0A1L7DQ41	3089	['CHAP']
G1C4Z3	3090	['CHAP']
A0A4D6DT42	3091	['CHAP']
A0A4D6DSL8	3092	['CHAP']
S6CUC3	3093	['CHAP']
S5MRN1	3094	['CHAP']
A0A411BIU2	3095	['CHAP']

X2KRT0	3096	['CHAP']
A0A4Y5TMC4	3097	['CHAP']
I4DSK2	3098	['CHAP']
A0A0S2MXW6	3099	['CHAP']
A0A4D6BKV8	3100	['CHAP']
A0A4D6BJN0	3101	['GH24']
I6ZID6	3102	['GH24']
A0A223LGG9	3103	['GH24']
E1A185	3104	['GH24']
A0A2S1PEA0	3105	['GH24']
Q19CF2	3106	['GH24']
A0A2S1PGU3	3107	['GH24']
J7KKQ3	3108	['GH24']
A0A219YC19	3109	['GH24']
A0A291LDA0	3110	['GH24']
A0A291LEA5	3111	['GH24']
A0A411B871	3112	['GH24']
E1A2L4	3113	['GH24']
A0A2H4YFB5	3114	['GH24']
Q76YA6	3115	['GH24']
E5DQQ0	3116	['GH24']
A0A411BD81	3117	['GH24']
Q5G7N2	3118	['GH24']
R9TN12	3119	['GH24']
A0A2R4ALG9	3120	['GH24']
A0A3G8F2M1	3121	['GH24']
A0A2I7S3P8	3122	['GH24']
A0A2I7RTX0	3123	['GH24']
A0A2I7S0L3	3124	['GH24']
A0A2I7R9W0	3125	['GH24']
R9UAZ1	3126	['GH24']
A0A2I7S8G4	3127	['GH24']
A0A385GK24	3128	['GH24']
K0IMR6	3129	['GH24']
A0A2L0HPR8	3130	['GH24']
A0A2K9VHI2	3131	['GH24']
I2FLU0	3132	['GH24']
A0A2Z4Q123	3133	['GH24']
A0A4Y1NTQ4	3134	['GH24']
M1IDZ3	3135	['GH24']
A0A4Y1NUD1	3136	['GH24']
A0A0H4A794	3137	['GH24']
A0A3G2YTL2	3138	['GH24']
A0A1J0GVQ7	3139	['GH24']
A0A1Q1PW74	3140	['GH24']
A0A4Y1NTQ2	3141	['GH24']

A0A4Y1NTU6	3142	['GH24']
A0A3Q9R6C9	3143	['GH24']
A0A4Y5P1M6	3144	['GH24']
I3UMC3	3145	['GH24']
A0A2S1GN54	3146	['GH24']
S4S2D7	3147	['GH24']
A0A1B1INP2	3148	['GH24']
A0A1I9L2K1	3149	['GH24']
A0A513ZZU2	3150	['GH24']
A0A514A0L5	3151	['GH24']
A0A514A0W5	3152	['GH24']
H9C148	3153	['GH24']
A0A2I7R771	3154	['GH24']
A0A2Z4Q150	3155	['Ami_2', 'LGFP', 'LGFP']
A0A515MHN7	3156	['Ami_2', 'LGFP', 'LGFP']
A0A160DG57	3157	['Ami_2', 'LGFP', 'LGFP']
A0A166YCP7	3158	['Ami_2', 'LGFP', 'LGFP']
A0A249XQ90	3159	['Ami_2', 'LGFP', 'LGFP']
A0A1B3B0A6	3160	['Ami_2', 'LGFP', 'LGFP']
A0A1B3B0T6	3161	['Ami_2', 'LGFP', 'LGFP']
A0A2P1JSD2	3162	['Ami_2', 'LGFP', 'LGFP']
A0A4Y5TYE7	3163	['Ami_2', 'LGFP', 'LGFP']
A0A0E3T679	3164	['Ami_2', 'LGFP', 'LGFP']
A0A385E048	3165	['Ami_2', 'LGFP', 'LGFP']
A0A160DD15	3166	['LGFP', 'LGFP']
A0A385D1L3	3167	['LGFP', 'LGFP']
A0A1B3B095	3168	['LGFP', 'LGFP', 'LGFP']
A0A160DCJ5	3169	['LGFP']
A0A3T0IL62	3170	['LGFP', 'LGFP']
A0A4Y5TZF9	3171	['LGFP']
A0A385DSB3	3172	['LGFP']
A0A4Y5TZ44	3173	['LGFP']
A0A411AXN0	3174	['LGFP']
A0A514CXC3	3175	['LGFP']
A0A1B3B1V0	3176	['LGFP', 'LGFP']
A0A1C9EHY4	3177	['LGFP']
A0A481VUU7	3178	['LGFP']
D6QWN3	3179	['Ami_3', 'SH3']
A0A1I9S5W9	3180	['Ami_3', 'SH3']
M4HNC8	3181	['Ami_3', 'SH3']
A0A385E7J7	3182	['Ami_3', 'SH3']
A0A219UQL8	3183	['Ami_3', 'SH3']
A0A3Q8CHX2	3184	['Ami_3', 'SH3']
A0A0E3DEU5	3185	['Ami_3', 'SH3']
A0A346TNH1	3186	['Ami_3', 'SH3']
G9J241	3187	['Ami_3', 'SH3']

A0A218KD91	3188	['Ami_3', 'SH3']
A0A4D6AUD3	3189	['Ami_5', 'CW_7', 'GLUCO']
A0A4D6B6E8	3190	['Ami_5', 'CW_7', 'GLUCO']
A0A4D6BA45	3191	['Ami_5', 'CW_7', 'GLUCO']
A0A4D6AE71	3192	['Ami_5', 'CW_7', 'GLUCO']
A0A4D6AJQ7	3193	['Ami_5', 'CW_7', 'GLUCO']
A0A4D6AF42	3194	['Ami_5', 'CW_7', 'GLUCO']
A0A4D6AEB7	3195	['Ami_5', 'CW_7', 'GLUCO']
A0A4D6ADY3	3196	['Ami_5', 'CW_7', 'GLUCO']
A0A4D6A067	3197	['Ami_5', 'CW_7', 'GLUCO']
A0A4D5ZZT7	3198	['Ami_5', 'CW_7', 'GLUCO']
A0A4D6AQV3	3199	['Ami_5', 'CW_7', 'GLUCO']
A1EAB6	3200	['Ami_5', 'CW_7', 'CW_7', 'GLUCO']
A0A4D6AJS3	3201	['Ami_5', 'CW_7', 'CW_7', 'GLUCO']
A0A4D6AMV4	3202	['Ami_5', 'CW_7', 'CW_7', 'GLUCO']
A0A4D6AJE4	3203	['Ami_5', 'CW_7', 'CW_7', 'GLUCO']
A0A2L1IVJ9	3204	['Ami_2', 'SLT_related']
A0A4D6E0V0	3205	['Ami_2', 'SLT_related']
A0A222YYD3	3206	['Ami_2', 'LysM', 'SLT_related']
A0A222YZS4	3207	['Ami_2', 'LysM', 'SLT_related']
A0A223FZU2	3208	['Ami_2', 'LysM', 'SLT_related']
A0A411B613	3209	['Ami_2', 'LysM', 'SLT_related']
A0A482JFS3	3210	['Ami_2', 'LysM', 'SLT_related']
A0A514U1V4	3211	['Ami_2', 'LysM', 'SLT_related']
A0A222Z0G3	3212	['Ami_2', 'LysM', 'SLT_related']
A0A222YYH6	3213	['Ami_2', 'LysM', 'SLT_related']
A0A0A0RQG1	3214	['Ami_2', 'LysM', 'SLT_related']
A0A221SAT9	3215	['Ami_2', 'LysM', 'SLT_related']
A0A514DK00	3216	['Ami_2', 'LysM', 'SLT_related']
A0A514DET7	3217	['Ami_2']
A0A411CFY3	3218	['Ami_2', 'LysM']
A0A411C4A9	3219	['Ami_2', 'LysM']
A0A345M9R9	3220	['Ami_2', 'LysM']
A0A345L1L5	3221	['Ami_2', 'LysM']
A0A345MDV6	3222	['Ami_2', 'LysM']
A0A385DV03	3223	['Ami_2', 'LysM']
A0A345GT70	3224	['Ami_2', 'LysM']
A0A3Q9R4P8	3225	['Ami_2', 'LysM']
A0A222YW92	3226	['Ami_2', 'LysM']
A0A345MG90	3227	['Ami_2', 'LysM']
A0A345M8H2	3228	['Ami_2', 'LysM']
A0A4Y6EPA6	3229	['Ami_2']
A0A345MH82	3230	['Ami_2']
A0A411AXW9	3231	['Ami_2']
A0A4D6E2L4	3232	['Ami_2']
A0A2Z4QC94	3233	['Ami_2', 'PG_1']

A0A291LGZ3	3234	['Ami_2', 'PG_1']
A0A291LHE3	3235	['Ami_2', 'PG_1']
A0A291LHU5	3236	['Ami_2', 'PG_1']
A0A345MI49	3237	['Ami_2', 'PG_1']
A0A411CPT1	3238	['Ami_2', 'PG_1']
K4HYE4	3239	['Ami_2']
A0A1J0MCN5	3240	['Ami_2']
A0A1J0MD01	3241	['Ami_2']
A0A1J0MBU5	3242	['Ami_2']
A0A411AXF9	3243	['Ami_2']
A0A4D6E0A3	3244	['Ami_2', 'LysM']
A0A386K7V8	3245	['Ami_2', 'LysM']
D0UWE9	3246	['Ami_2']
A0A386KBJ2	3247	['Ami_2']
A0A142K646	3248	['Ami_2']
A0A2P1N078	3249	['Ami_2']
A0A2D1GP54	3250	['Ami_2']
A0A0U4K3I2	3251	['Ami_2']
A0A0E3JQE4	3252	['Ami_2']
A0A125RNW2	3253	['Ami_2']
A0A0S2MU54	3254	['Ami_2']
A0A4D6E4T0	3255	['Ami_2']
A0A0M4RBB1	3256	['Ami_2', 'LysM']
A0A2P1N0I7	3257	['Ami_2']
A0A411AY00	3258	['Ami_2']
A0A067XGV1	3259	['Ami_2']
A0A4D6DTU0	3260	['Ami_2']
A0A4D6DRS8	3261	['Ami_2']
A0A4D6DT44	3262	['Ami_2']
A0A4D6DT39	3263	['Ami_2']
A0A4D6DTJ0	3264	['Ami_2']
A0A1M4NDT8	3265	['Ami_2']
A0A4D6DSZ3	3266	['Ami_2']
A0A0S2MY35	3267	['Ami_2']
A0A4D6DSR9	3268	['Ami_2']
A0A4D6DTK3	3269	['Ami_2']
A0A4D6DT87	3270	['Ami_2']
A0A288TXU9	3271	['Ami_2']
A0A3B8DXR3	3272	['Ami_2']
A0A411B7H0	3273	['Ami_2']
A0A096XT38	3274	['Ami_2']
A0A2Z6FZA5	3275	['Ami_2']
A0A4D6DTJ6	3276	['Ami_2']
A0A3P5VQX7	3277	['Ami_2']
A0A0E3TBA5	3278	['Ami_2']
A0A4D6DUM0	3279	['Ami_2']

A8E261	3280	['Ami_2']
A0A2Z6FZW5	3281	['Ami_2']
B1ABI9	3282	['Ami_2']
D4N4K8	3283	['Ami_2', 'ZoocinA_TRD']
A0A0D3MK08	3284	['Ami_2', 'ZoocinA_TRD']
A0A059PAL4	3285	['Ami_2', 'ZoocinA_TRD']
A0A0D3MKG1	3286	['Ami_2', 'ZoocinA_TRD']
A0A4D6A680	3287	['Ami_2', 'ZoocinA_TRD']
A0A4D6AWQ3	3288	['CHAP', 'Ami_2']
A0A291I9H5	3289	['GH25', 'SH3', 'LysM']
A0A2K9VDG6	3290	['GH25', 'SH3', 'LysM']
A0A3Q8CA00	3291	['GH25', 'SH3', 'LysM']
A0A3S7UPN4	3292	['GH25', 'SH3', 'LysM']
A0A3Q8HZL4	3293	['GH25', 'SH3', 'LysM']
A0A2K9VCM3	3294	['GH25', 'SH3', 'LysM']
A0A3S7UPH0	3295	['GH25', 'SH3', 'LysM']
A0A2K9V5A4	3296	['GH25', 'SH3', 'LysM']
A0A2Z2E736	3297	['GH25', 'SH3', 'LysM']
A0A1I9KK58	3298	['GH25', 'SH3', 'LysM']
A0A2H4PB98	3299	['GH25', 'SH3', 'LysM']
A0A2K9VCD7	3300	['GH25', 'SH3', 'LysM']
Q5ULM6	3301	['GH25', 'SH3', 'LysM']
K4I1Y4	3302	['GH25', 'SH3', 'LysM']
A0A1S5RCQ2	3303	['GH25', 'SH3', 'LysM']
A0A2P0ZKX1	3304	['GH25', 'SH3', 'LysM']
A0A4Y5FFC4	3305	['GH25', 'LysM']
A0A4Y5FEB1	3306	['GH25', 'LysM']
O03950	3307	['GH25', 'SH3', 'LysM']
A0A4Y5FEU1	3308	['GH25', 'LysM']
A0A4Y5FF73	3309	['GH25', 'LysM']
A0A385E984	3310	['GH25', 'Ami02_C']
A0A222Z5F8	3311	['GH25', 'Ami02_C']
A0A024AZX0	3312	['GH25', 'Ami02_C']
A0A024B030	3313	['GH25', 'Ami02_C']
A0A482IGD8	3314	['GH25', 'LysM', 'LysM', 'LysM']
A0A386K875	3315	['GH25', 'LysM', 'LysM']
A0A172JIA8	3316	['GH25', 'LysM', 'LysM', 'LysM']
A0A223LD43	3317	['GH25', 'LysM', 'LysM', 'LysM']
A0A160LKP9	3318	['GH25']
A0A1Z1LZN8	3319	['GH25']
A0A2H4J966	3320	['GH25', 'Ami02_C']
A7KV11	3321	['GH25']
A0A218KCJ1	3322	['GH25']
A9QTG1	3323	['GH25', 'SH3']
J9PQX3	3324	['GH25', 'SH3']
A0A0E3D9B5	3325	['GH25', 'SH3']

X2JN49	3326	['GH25', 'SH3']
A0A385EAG5	3327	['GH25', 'SH3']
A0A4P8N7H3	3328	['GH25', 'Ami02_C']
I3WTZ2	3329	['GH25', 'Ami02_C']
A0A1B1P783	3330	['GH25', 'Ami02_C']
D2XR33	3331	['GH25', 'Ami02_C']
A0A386KSS7	3332	['GH25', 'Ami02_C']
A0A1B1PAP0	3333	['GH25', 'Ami02_C']
A0A3G8F584	3334	['GH25', 'Ami02_C']
A0A1B1PAF8	3335	['GH25', 'Ami02_C']
A0A1X9SHN6	3336	['GH25', 'Ami02_C']
A0A514AAM8	3337	['GH25', 'Ami02_C']
A0A1I9S6P7	3338	['GH25', 'Ami02_C']
A0A1B1PAK9	3339	['GH25', 'Ami02_C']
A0A223LI94	3340	['GH25', 'Ami02_C']
A0A3T0IIX9	3341	['GH25', 'Ami02_C']
A0A3T0IJ07	3342	['GH25', 'Ami02_C']
A0A3T0IJ27	3343	['GH25', 'Ami02_C']
Q0GXT8	3344	['Ami_2']
A0A455VPF3	3345	['Ami_2']
C3U2S5	3346	['Ami_2']
A0A1W6JHI7	3347	['Ami_2']
H9AWU1	3348	['Ami_2']
A0A097BY70	3349	['Ami_2']
D7RWE7	3350	['Ami_2']
D7RWK6	3351	['Ami_2', 'SH3']
A0A249XZR2	3352	['Ami_2']
A0A0S2MYH4	3353	['Ami_2']
A0A411B6T3	3354	['Ami_2']
A0A4D6AAM3	3355	['Ami_2', 'CW_7', 'CW_7']
D9J0J9	3356	['Ami_2', 'SH3']
A0A060AHF9	3357	['Ami_2']
A0A0M4S679	3358	['Ami_2']
A0A1J0MFY3	3359	['PET_M23', 'Ami_2', 'SH3']
A0A4P6QXB6	3360	['PET_M23', 'Ami_2', 'SH3']
Q4ZD58	3361	['PET_M23', 'Ami_2', 'SH3']
A0A499SHK0	3362	['PET_M23', 'Ami_2', 'SH3']
A0A499SJP3	3363	['PET_M23', 'Ami_2', 'SH3']
A0A1Q1PW31	3364	['Ami_2']
A0A1Q1PVY4	3365	['Ami_2']
A0A1S6KVC6	3366	['Ami_2']
A0A3G2YSK6	3367	['Ami_2']
A0A3G2YSP8	3368	['Ami_2']
A0A2D0W917	3369	['SLT_related']
A0A2D0W9X7	3370	['SLT_related']
A0A2D0W9N2	3371	['SLT_related']

A0A2D0W9F2	3372	['SLT_related']
A0A2D0W997	3373	['SLT_related']
A9J597	3374	['SLT_related']
A0A411BAT0	3375	['SLT_related']
A0A411BCU2	3376	['SLT_related']
I6WMZ3	3377	['SLT_related']
A0A0N9ERA0	3378	['SLT_related']
A0A0U1VU02	3379	['SLT_related']
A0A1B0Z008	3380	['SLT_related']
A0A2K8HPC2	3381	['SLT_related']
G4KKM7	3382	['SLT_related']
A0A291LA05	3383	['PROKAR_LIPOPROTEIN', 'SLT_related']
A0A4D6ALT9	3384	['LysM', 'CHAP']
D0R0F0	3385	['Ami_3', 'LysM']
A0A4D6A394	3386	['Ami_3', 'LysM', 'CHAP']
A0A1X9I678	3387	['Ami_3', 'LysM', 'CHAP']
A0A1B0RXF2	3388	['Ami_3', 'LysM', 'CHAP']
A0A4D6AP35	3389	['Ami_3', 'LysM', 'CHAP']
A0A1B0RXL4	3390	['Ami_3', 'LysM', 'CHAP']
A0A4D6ACU6	3391	['Ami_3', 'LysM', 'CHAP']
A0A4D6AQ16	3392	['Ami_3', 'LysM', 'CHAP']
A0A4D6A315	3393	['Ami_3', 'LysM', 'CHAP']
A0A4D6ALE7	3394	['Ami_3', 'LysM', 'CHAP']
A0A4D5ZY97	3395	['Ami_3', 'LysM', 'CHAP']
A0A4D6A4W3	3396	['Ami_3', 'LysM', 'CHAP']
A0A4D6AN23	3397	['Ami_3']
A0A4D6B7G6	3398	['Ami_3', 'LysM']
A0A4D6BGQ7	3399	['GLUCO']
A0A4D6BCB3	3400	['GLUCO', 'Ami_3']
A0A4D6AHN4	3401	['GLUCO', 'Ami_3', 'LysM']
A0A4D6AIQ0	3402	['GLUCO', 'Ami_3', 'LysM']
A0A2Z5HEY5	3403	['PET_M23', 'PG_1']
A0A2Z5H8S4	3404	['PET_M23', 'PG_1']
A0A386KGP3	3405	['PET_M23', 'PG_1']
A0A2Z5H4J3	3406	['PET_M23', 'PG_1']
A0A4Y6EBV5	3407	['PET_M23', 'PG_1']
A0A4Y6EEQ2	3408	['PET_M23', 'PG_1']
A0A2Z5H5J0	3409	['PET_M23', 'PG_1']
A0A2R4A059	3410	['PET_M23', 'PG_1']
A0A3B8DKH0	3411	['PET_M23', 'PG_1']
A0A140G755	3412	['CHAP']
A0A386KNJ3	3413	['CHAP']
A0A222ZIX0	3414	['CHAP']
A0A222Z9J2	3415	['CHAP']
A0A0M4RB81	3416	['CHAP']
A0A0U4K087	3417	['CHAP']

A0A222Z9B2	3418	['CHAP']
A0A222Z7U7	3419	['CHAP', 'Ami_2']
A0A0U4JEI5	3420	['CHAP']
A0A0U4KRD4	3421	['CHAP', 'Ami_2']
A0A288WFW6	3422	['Ami_2', 'SH3']
A0A288WFT1	3423	['Ami_2', 'SH3']
C7DT94	3424	['Ami_2', 'SH3']
D3WK93	3425	['Ami_2', 'SH3']
A9QTG3	3426	['Ami_2', 'SH3']
A0A288WFQ3	3427	['Ami_2', 'SH3']
A0A0A7AQV3	3428	['Ami_2', 'SH3']
A0A1B1P878	3429	['Ami_2']
A0A2H4JAS1	3430	['Ami_2', 'SH3', 'SH3']
M1HNA7	3431	['Ami_3', 'LysM', 'LysM']
U5PW24	3432	['Ami_3', 'LysM', 'LysM']
U5PTK7	3433	['Ami_3', 'LysM', 'LysM']
M1IEI0	3434	['Ami_3', 'LysM', 'LysM']
M1IDI0	3435	['Ami_3', 'LysM', 'LysM']
U5PWK4	3436	['Ami_3', 'LysM', 'LysM']
M1IQ67	3437	['Ami_3', 'LysM', 'LysM']
A0A1S5QTQ1	3438	['Ami_3', 'LysM', 'LysM']
A0A0B5KNE7	3439	['GH24']
A0A4D6CHP3	3440	['GH24']
A0A0B5L5F7	3441	['GH24']
A0A0B5L1J1	3442	['GH24']
L7TJR2	3443	['GH24']
Q5QF59	3444	['GH24']
A0A2K8HZR8	3445	['GH24']
A0A481V389	3446	['GH24']
A0A481V442	3447	['GH24']
A0A345ANS5	3448	['GH24']
I6NW41	3449	['GH24']
Q6V7L5	3450	['GH24']
C5IHR6	3451	['GH24']
I6X6V3	3452	['GH24']
A0A345BQX2	3453	['GH24']
A0A345MKX1	3454	['GH24']
K4JQD1	3455	['GH24']
A0A1V0E9P9	3456	['GH24']
J3SVU8	3457	['GH24']
A0A1V0EBQ3	3458	['GH24']
K4JS66	3459	['GH24']
A0A385E9F7	3460	['GH24']
A0A385EBE6	3461	['GH24']
A0A385EFZ0	3462	['GH24']
K4JRQ4	3463	['GH24']

A0A385EAH6	3464	['GH24']
A0A1W5P530	3465	['GH24']
A0A3G8F2G6	3466	['GH24']
K9RZW8	3467	['GH24']
A0A0K8IXP8	3468	['GH24']
H6UK32	3469	['GH24']
A0A513ZZ78	3470	['GH24']
A0A1S5Q8G1	3471	['GH24']
A0A249XLC1	3472	['GH24']
A0A2P9JZU6	3473	['GH24']
I7K3G5	3474	['GH24']
A0A1L7DQJ6	3475	['GH24']
A0A1K2IXT0	3476	['GH24']
A0A0H4TH14	3477	['GH24']
A0A2S1PFQ6	3478	['GH24']
A0A411B6J4	3479	['GH24']
A9ZT37	3480	['GH24']
R9TQ00	3481	['GH24']
A0A2I7RNI5	3482	['GH24']
A0A2S1GLD1	3483	['GH24']
I6PBN2	3484	['GH24']
A0A2Z4Q029	3485	['GH24']
A0A482IMA4	3486	['GH24']
A0A2Z2FFX8	3487	['GH24']
A0A482IEL8	3488	['GH24']
A0A386K4Y0	3489	['GH24']
A0A4D6DXT9	3490	['GH24']
A0A140XG79	3491	['GH24']
A0A513ZWS7	3492	['GH24']
A0A1B0V7G8	3493	['GH24']
A0A077SLF3	3494	['GH24']
Q71TF3	3495	['GH24']
A0A499QMM1	3496	['GH24']
Q94MY6	3497	['GH24']
P51728	3498	['GH24']
Q1I0Z1	3499	['GH24']
A0A0M3LPP5	3500	['GH24']
Q19UR6	3501	['GH24']
A0A0M3LPQ1	3502	['GH24']
M4W600	3503	['GH24', 'SH3']
A0A1W6JSI0	3504	['GH24', 'SH3']
A0A1X9SGL2	3505	['GH24', 'SH3']
A0A482MHR6	3506	['GH24', 'YkuD']
A0A1L2BWX1	3507	['GH24', 'YkuD']
A0A482MIV2	3508	['GH24', 'YkuD']
K4I410	3509	['GH24']

A0A509EQQ2	3510	['GH24', 'PG_1']
F4YCN5	3511	['GH24', 'PET_C70']
Q9FZS7	3512	['GH24']
A0A2K9V500	3513	['GH24']
H6VUC2	3514	['GH24']
A0A2D2W2U6	3515	['GH24']
A0A345BR99	3516	['GH24']
A0A345BRQ0	3517	['GH24']
L7TM06	3518	['GH24']
A0A0S0N859	3519	['GH24']
F8TVP0	3520	['GH24']
K7ZLE2	3521	['GH24']
A0A3B8DKK9	3522	['GH24']
A0A0C4K550	3523	['GH24']
A0A3G1L3E6	3524	['GH24']
F8TV87	3525	['GH24']
G8GWD3	3526	['GH24']
M4ST54	3527	['GH24']
A0A2I5ARC6	3528	['GH24', 'PET_M15']
F4YCT6	3529	['GH24', 'PET_M15']
G8EY22	3530	['GH24', 'PET_M15']
Q5GQI3	3531	['GH24']
A0A1D8KUS6	3532	['GH24']
F5B3Y3	3533	['GH24']
I3ULI0	3534	['GH24']
A0A514CVS2	3535	['GH24']
A0A514CW31	3536	['GH24']
A0A514CTT7	3537	['GH24']
A0A2S1GMS1	3538	['GH24']
A0A2S1GN17	3539	['GH24']
A0A2H4P843	3540	['GH24']
A0A2S1GML4	3541	['GH24']
A0A2S1GMZ6	3542	['GH24']
Q0E5W1	3543	['GH24']
D2EBU1	3544	['GH24']
M4SLT8	3545	['PET_C39', 'GH24']
A0A1D7SIM9	3546	['PET_C39', 'GH24']
A0A1D7SHE4	3547	['PET_C39', 'GH24']
A0A1D7SJE3	3548	['PET_C39', 'GH24']
A0A1D7SMF6	3549	['PET_C39', 'GH24']
A0A1D7SM75	3550	['PET_C39', 'GH24']
A0A1D7SK56	3551	['PET_C39', 'GH24']
O56785	3552	['CHAP', 'GLUCO']
Q4ZE13	3553	['CHAP', 'GLUCO']
A0A1J0MFI1	3554	['CHAP', 'GLUCO']
A0A494WA46	3555	['CHAP', 'GLUCO']

A0A499SJZ7	3556	['CHAP', 'GLUCO']
A0A499SIF7	3557	['CHAP', 'GLUCO']
A0A1J0MF68	3558	['CHAP', 'GLUCO']
A0A494WCU8	3559	['CHAP', 'GLUCO']
A0A494WIP8	3560	['CHAP', 'GLUCO']
Q4ZC50	3561	['CHAP', 'GLUCO']
A1BU73	3562	['CHAP', 'GLUCO']
A1BTX6	3563	['CHAP', 'GLUCO']
A0A1W6JPD6	3564	['CHAP', 'GLUCO']
A0A513ZXM8	3565	['GLUCO']
Q4ZCC7	3566	['CHAP', 'GLUCO']
A0A1W6JNR1	3567	['CHAP', 'GLUCO']
K4NZK4	3568	['Ami_2']
A0A0K1Y9U1	3569	['Ami_2', 'LysM', 'PG_1']
A0A0K1Y9L3	3570	['Ami_2', 'LysM', 'PG_1']
A0A0K1Y9C5	3571	['Ami_2', 'LysM', 'PG_1']
A0A1B1PAB5	3572	['Ami_2', 'LysM', 'PG_1']
A0A291AVF5	3573	['Ami_2', 'LysM', 'PG_1']
A0A291AV60	3574	['Ami_2', 'LysM', 'PG_1']
A0A514U360	3575	['Ami_2', 'LysM', 'PG_1']
A0A291AW37	3576	['Ami_2', 'LysM', 'PG_1']
A0A2P1JTQ5	3577	['Ami_2']
A0A291LHM6	3578	['Ami_2', 'PG_1']
A0A411CPR5	3579	['Ami_2', 'PG_1', 'PG_1']
A0A1J0MC10	3580	['Ami_2']
A0A1J0MCP8	3581	['Ami_2']
A0A1V0E659	3582	['Ami_2']
K4I2E3	3583	['Ami_2']
I7A9I3	3584	['Ami_2']
A0A385DNT4	3585	['Ami_2']
A0A0A0RNQ7	3586	['PET_M15', 'PG_1']
A0A0S2SXK4	3587	['PET_M15', 'PG_1']
A0A0A0RPZ6	3588	['PET_M15', 'PG_1']
A0A0C5AFG6	3589	['PET_M15', 'PG_1']
U5PW86	3590	['PET_M15', 'PG_1']
U5PWC4	3591	['PET_M15', 'PG_1']
U5PVY5	3592	['PET_M15', 'PG_1']
A0A0S2SY37	3593	['PET_M15', 'SH3']
S5Y0K1	3594	['PET_M15', 'SH3']
A0A514A8Z7	3595	['PET_M15', 'SH3']
A0A1X9SG19	3596	['PET_M15', 'SH3']
H9NAL3	3597	['PET_M15', 'SH3']
U5PWR9	3598	['PET_M15', 'SH3']
A0A059T5H8	3599	['PET_M15']
A0A514U6Z2	3600	['PET_M15']
J9QRT9	3601	['PET_M15']

S4U8N6	3602	['PET_M15']
Q0PRU8	3603	['PET_M15', 'PSA_CBD']
A0A2U8UV78	3604	['PET_M15', 'PSA_CBD']
A8ASL5	3605	['PET_M15', 'PSA_CBD']
A8ATW4	3606	['PET_M15']
U5PRL9	3607	['PET_M15', 'SH3', 'SH3']
A0A140HM31	3608	['PET_M15', 'SH3', 'SH3']
A0A1V0DZZ3	3609	['Ami_3']
A0A0A8WIC2	3610	['Ami_3']
A0A0A8WE15	3611	['Ami_3']
E2ELK8	3612	['Ami_3']
F6K8M2	3613	['Ami_3']
A0A0A8WHU0	3614	['Ami_3']
J9QED4	3615	['Ami_3']
A0A3G1E396	3616	['Ami_3']
X5JAJ5	3617	['Ami_3']
A0A090D823	3618	['Ami_3']
B6SBV8	3619	['Ami_3']
A0A0A8WFX1	3620	['Ami_3']
A0A3G1E3H7	3621	['Ami_3']
A0A090D803	3622	['Ami_3']
D6QWL9	3623	['Ami_3', 'PG_1']
D6QWM4	3624	['Ami_3', 'PG_1']
D6QWM3	3625	['Ami_3', 'PG_1']
D6QWM2	3626	['Ami_3', 'PG_1']
D6QWM1	3627	['Ami_3', 'PG_1']
D6QWM5	3628	['Ami_3', 'PG_1']
D6QWM0	3629	['Ami_3', 'PG_1']
D6QWP5	3630	['Ami_3', 'PG_1']
A0A514A702	3631	['SLT_related']
A0A0K1LQC3	3632	['SLT_related']
A0A0K2FHY9	3633	['SLT_related']
K7NPW3	3634	['SLT_related']
A0A1G4GQA5	3635	['SLT_related']
M9V198	3636	['SLT_related']
A0A1W5P0K6	3637	['SLT_related']
J7I134	3638	Unknown
E1A1M0	3639	Unknown
I6YMG0	3640	Unknown
J7KES7	3641	Unknown
A0A223LFC0	3642	Unknown
A0A240F3V1	3643	['SLT_related']
A0A4D6BJX6	3644	Unknown
Q6U9J5	3645	['SLT_related']
Q56EQ6	3646	['SLT_related']
A0A482G9U5	3647	['SLT_related']

A0A482GGJ4	3648	['SLT_related']
C4MYR3	3649	['SLT_related']
A0A482MM99	3650	['SLT_related']
A0A482GDB5	3651	['SLT_related']
E3SFK4	3652	Unknown
E5DID2	3653	['SLT_related']
A0A1D3RKJ3	3654	['SLT_related']
A0A410T5W4	3655	['SLT_related']
E5EPS2	3656	['SLT_related']
A0A3Q9R716	3657	Unknown
A0A4Y1NKH2	3658	['SLT_related']
E5E4C1	3659	['SLT_related']
D9I658	3660	Unknown
E5EYR4	3661	Unknown
A0A126DI76	3662	['GH19']
A0A386KDH5	3663	['GH19']
A0A4P2UQ66	3664	['GH19']
A0A386KLA0	3665	['GH19']
A0A386KJS9	3666	['GH19']
A0A075DXC4	3667	['GH19']
A0A3B8DZ61	3668	['GH19']
K4I3B7	3669	['GH19']
A0A2S1GTV8	3670	['GH19']
W6AQZ3	3671	['GH19']
A0A481S282	3672	['GH19']
D6QWP0	3673	['GH19']
E2GM11	3674	['GH19']
A0A1V0DZ63	3675	['GH19']
A0A0F6WBX9	3676	Unknown
A0A0F6YPI2	3677	Unknown
L7TRW2	3678	Unknown
A0A291AUN6	3679	Unknown
A0A0X8XCA1	3680	['PET_C39', 'GH19']
A0A125T2V9	3681	['PET_C39', 'GH19']
A0A2Z4QGM7	3682	['GH19']
A0A2Z4QHK0	3683	['GH19']
B4UTR7	3684	['GH19']
A0A1B1IY38	3685	['GH19']
M4Q3E2	3686	['GH19']
R9U4A9	3687	['GH19']
A0A0N9SIT8	3688	['LT_GEWL_like']
A0A1W6JSC2	3689	['LT_GEWL_like']
A0A1X9SGN0	3690	['LT_GEWL_like']
A0A386KT13	3691	['LT_GEWL_like']
A0A1B1PAN2	3692	['LT_GEWL_like']
A0A1B1PAK0	3693	['LT_GEWL_like']

A0A1X9SHL4	3694	['LT_GEWL_like']
A0A1I9S6P5	3695	['LT_GEWL_like']
A0A223LJE1	3696	['LT_GEWL_like']
A0A514AAR3	3697	['LT_GEWL_like']
A0A411B580	3698	['CHAP', 'PG_1']
A0A385DSJ0	3699	['CHAP', 'PG_1']
A0A411CP72	3700	['CHAP', 'PG_1']
A0A385E0L2	3701	['CHAP', 'PG_1']
A0A411CPI2	3702	['CHAP', 'PG_1']
A0A1J0GNX0	3703	['CHAP', 'PG_1']
A0A2H4PGE4	3704	['CHAP']
A0A2H5BLQ0	3705	['CHAP', 'PG_1']
A0A481W0D0	3706	['CHAP']
K4IBM9	3707	['CHAP']
A0A411CYF9	3708	['CHAP']
A0A4D6E3U7	3709	['CHAP']
A0A411B102	3710	['CHAP']
A0A411B3S5	3711	['CHAP']
A0A411AYU7	3712	['CHAP']
A0A411B2Y7	3713	['CHAP']
A0A2H5BLZ5	3714	['CHAP']
A0A291LH93	3715	['CHAP']
A0A2H5BLB7	3716	['CHAP']
A0A2P1JSZ8	3717	['CHAP']
A0A221J6Q6	3718	['Ami_2']
A0A0U4IPM3	3719	['Ami_2']
A0A482J991	3720	['Ami_2']
A0A4Y5U0A0	3721	['Ami_2']
A0A222ZH75	3722	['Ami_2']
A0A3G3M107	3723	['Ami_2']
A0A3G3LY06	3724	['Ami_2']
A0A286S1R3	3725	['CHAP', 'Ami_2']
A0A0U4JPJ5	3726	['CHAP', 'Ami_2']
A0A0U4IHI0	3727	['CHAP', 'Ami_2']
A0A0U4IT54	3728	['CHAP', 'Ami_2']
A0A0U4K335	3729	['PET_M23']
A0A0M4R0N4	3730	['PET_M23']
A0A222ZHW0	3731	['PET_M23']
A0A222ZHL5	3732	['PET_M23']
A0A221J6Q1	3733	['PET_M23']
A0A4Y5U044	3734	['PET_M23']
Q5ZQZ1	3735	['SLT_related']
J9SGR6	3736	['PROKAR_LIPOPROTEIN', 'SLT_related']
A0A4Y6EJS6	3737	['PROKAR_LIPOPROTEIN', 'SLT_related']
A0A0S4L2W4	3738	['PROKAR_LIPOPROTEIN', 'SLT_related']
J9SHG5	3739	['PROKAR_LIPOPROTEIN', 'SLT_related']

A0A481V836	3740	['PROKAR_LIPOPROTEIN', 'SLT_related']
J9SH25	3741	['PROKAR_LIPOPROTEIN', 'SLT_related']
A0A481V669	3742	['PROKAR_LIPOPROTEIN', 'SLT_related']
A0A2H4J7R8	3743	['SLT_related']
A0A2H4J9H7	3744	['SLT_related']
Q6QIC7	3745	['SLT_related']
A4JWP4	3746	['SLT_related']
A0A1C3NFR9	3747	['SLT_related']
A0A2P9JZI1	3748	['SLT_related']
B5TA73	3749	['SLT_related']
U6C6A5	3750	['SLT_related']
A0A2D1GPR8	3751	['Ami_2']
A0A385D2Z7	3752	['Ami_2']
G1D5A2	3753	['Ami_2']
A0A1L6BYI8	3754	['Ami_2']
A0A222YWG6	3755	['Ami_2']
A0A1C9EHM2	3756	['Ami_2']
G1JWF2	3757	['Ami_2']
A0A0K1LS76	3758	['Ami_2']
A0A345KTX9	3759	['DUF1906', 'LGFP', 'LGFP']
A0A160DGW5	3760	['DUF1906', 'LGFP', 'LGFP']
A0A514TZ74	3761	['DUF1906', 'LGFP', 'LGFP']
F1B2S6	3762	['Ami_2', 'LGFP', 'LGFP', 'LGFP']
A0A1C9EI81	3763	['LGFP', 'LGFP', 'LGFP']
A0A159B6I5	3764	['Ami_2', 'LGFP', 'LGFP', 'LGFP']
G9FHJ0	3765	['Ami_2', 'LGFP', 'LGFP']
G9FI08	3766	['Ami_2', 'LGFP', 'LGFP', 'LGFP']
M9MUE0	3767	['Ami_2', 'LGFP', 'LGFP', 'LGFP']
A0A1I9SA50	3768	['Ami_2', 'LGFP', 'LGFP', 'LGFP', 'LGFP']
A0A2U8ULA6	3769	['Ami_2', 'LGFP', 'LGFP', 'LGFP', 'LGFP']
A0A2U8UJS9	3770	['Ami_2', 'LGFP', 'LGFP', 'LGFP', 'LGFP']
A0A160DHD6	3771	['Ami_2']
Q9MBI0	3772	['Ami_2']
G9FH47	3773	['Ami_2']
D4P733	3774	['Ami_2']
A0A0K0N6L7	3775	['Ami_2']
A0A142KAY0	3776	['PET_M23', 'Ami_2']
A0A385E0G9	3777	['Ami_2']
A0A3G3MB35	3778	['Ami_2']
A0A4Y6EMZ2	3779	['PET_M23', 'Ami_2']
A0A385UI25	3780	['PET_M23', 'Ami_2']
A0A3G3LXC7	3781	['PET_M23', 'Ami_2']
A0A514DJG8	3782	['PET_M23', 'Ami_2']
A0A4Y6EMJ0	3783	['PET_M23', 'Ami_2']
A0A385UCI0	3784	['PET_M23', 'Ami_2']
A0A515MK78	3785	['PET_M23']

A0A4Y6EIU7	3786	['GH25']
A0A1W6JQH9	3787	['PET_M23', 'GH25']
A0A4Y6EMV4	3788	['PET_M23', 'GH25']
A0A2Z4Q8R8	3789	['PET_M23', 'GH25']
D4P815	3790	['PET_M23', 'GH25']
A0A1P8D5Q3	3791	['PET_M23', 'GH25']
H9EB52	3792	['SLT_related']
K7RVN3	3793	['SLT_related']
A0A345MCZ0	3794	['SLT_related']
A0A343UT88	3795	['SLT_related']
A0A384WYL7	3796	['SLT_related']
A0A384WX53	3797	['SLT_related']
A0A384WK54	3798	['SLT_related']
A0A411BJH6	3799	['SLT_related']
A0A0B5A075	3800	['Ami_2']
A0A4D6ALQ3	3801	['Ami_2']
A0A4D6AC33	3802	['Ami_2']
A0A4D6B3L2	3803	['Ami_2']
A0A4D6B353	3804	['Ami_2']
A0A4D6A9N0	3805	['Ami_2']
A0A4D6B1N0	3806	['Ami_2']
A0A386K8H2	3807	['CHAP', 'GH25', 'LysM', 'LysM', 'LysM']
A0A0U4JD12	3808	['CHAP', 'GH25', 'LysM', 'LysM', 'LysM']
A0A249XND5	3809	['CHAP', 'GH25', 'LysM', 'LysM', 'LysM']
A0A1I9SCW9	3810	['CHAP', 'GH25', 'LysM', 'LysM', 'LysM']
A0A0A7HER1	3811	['CHAP', 'GH25', 'LysM', 'LysM', 'LysM']
A0A286N2U4	3812	['CHAP', 'GH25', 'LysM', 'LysM', 'LysM']
A0A075KJD2	3813	['GH25', 'SH3']
U3PJ04	3814	['GH25', 'SH3']
F7V9F0	3815	['GH25', 'SH3']
A0A2P1CCE6	3816	['GH25', 'SH3']
F8J1A5	3817	['GH25', 'SH3']
A0A075KJI2	3818	['GH25', 'SH3']
A0A075KQK4	3819	['GH25', 'SH3']
A0A3Q8I7V4	3820	Unknown
A0A0A7DMK9	3821	['GH25', 'LysM', 'SH3']
A0A3G6JGZ1	3822	['GH25', 'LysM', 'SH3']
E3W8H6	3823	['GH25']
A9UJV4	3824	['GH25', 'LysM']
P33486	3825	['GH25']
A0A3G6JME2	3826	['GH25']
Q38373	3827	['GH25']
U3PCM2	3828	['GH25']
Q38359	3829	['GH25']
A0A3G6JGS6	3830	['GH25']
Q71JA9	3831	['GH25']

L0P6H6	3832	['GH25']
Q6SE63	3833	['GH25', 'SH3']
Q6SEB5	3834	['GH25', 'SH3']
Q38317	3835	['GH25', 'SH3']
Q65YV6	3836	['GH25', 'SH3']
X2CXX8	3837	['GH25', 'SH3']
A0A0D4DD04	3838	['Ami_2', 'CHAP']
A0A1W6JNM6	3839	['Ami_2', 'CHAP']
A0A1W6JQL2	3840	['Ami_2', 'CHAP']
A0A2H4J5A3	3841	['Ami_2', 'CHAP']
I6T7G5	3842	['Ami_2', 'CHAP']
H9A0Q2	3843	['Ami_2', 'CHAP']
A0A1X9SIB3	3844	['Ami_2']
A0A1P8CMK9	3845	['Ami_2']
S4T909	3846	['Ami_2']
A0A455VCL2	3847	['Ami_2']
I7DN74	3848	['Ami_2']
A0A0U1ZY89	3849	['Ami_2']
A0A1W6JPY4	3850	['Ami_2', 'CHAP']
A0A499SSY5	3851	['Ami_2']
A0A0K0N745	3852	['PET_C39']
A0A4Y6E9P9	3853	['PET_C39']
A0A1B3AYA2	3854	['PET_C39']
A0A345MIN7	3855	['PET_C39']
A0A2D1GFQ1	3856	['PET_C39']
A0A410TBZ5	3857	['PET_C39']
A0A1B3AZG3	3858	['PET_C39']
A0A410TD07	3859	['PET_C39']
A0A160DET0	3860	['PET_C39']
A0A482JH44	3861	['PET_C39']
A0A4Y6E7D0	3862	['CHAP']
Q4ZCZ2	3863	['CHAP']
A0A075LZV3	3864	['CHAP']
A0A385IRL8	3865	['CHAP']
Q9MBN4	3866	['CHAP']
A0A1X9SIB5	3867	['CHAP', 'SH3']
A0A1X9I9I9	3868	['CHAP']
A0A2S0X9X1	3869	['CHAP']
A0A2H4JBI2	3870	['Ami_3']
A0A1W6JNV3	3871	['CHAP', 'Ami_3']
A0A2H4J4H5	3872	['CHAP', 'Ami_3', 'SH3']
A0A2H4JCA1	3873	['CHAP', 'Ami_3', 'SH3']
A1BU77	3874	['CHAP', 'Ami_3']
A0A1W6JPE2	3875	['CHAP', 'Ami_3']
I6TG35	3876	['CHAP', 'Ami_3']
A0A1D6Z273	3877	['CHAP', 'Ami_3']

A0A2H4JBD0	3878	['CHAP', 'Ami_3', 'SH3']
A0A2H4J7F3	3879	['CHAP', 'Ami_3']
A0A2H4JAF4	3880	['CHAP', 'Ami_3']
A0A0N9SGX6	3881	['CHAP', 'Ami_3']
O80064	3882	['CHAP', 'Ami_3', 'SH3']
A0A1X9I9Y6	3883	['Ami_3']
A0A2R3ZXN5	3884	['Ami_3', 'SH3']
A0A4Y6EB79	3885	['Ami_3', 'SH3']
A0A4D6E508	3886	['Ami_2', 'LysM', 'CW_7', 'CW_7']
A0A2P1CFA9	3887	['Ami_2', 'LysM', 'CW_7', 'CW_7']
A0A4D6E2K7	3888	['Ami_2', 'LysM', 'CW_7', 'CW_7']
A0A514DGM4	3889	['Ami_2', 'LysM', 'CW_7', 'CW_7']
A0A2P1CFS5	3890	['Ami_2', 'LysM', 'CW_7', 'CW_7']
A0A4D6E595	3891	['Ami_2', 'LysM', 'CW_7', 'CW_7']
A0A0U4JY89	3892	['Ami_2', 'CW_7', 'CW_7', 'CW_7']
A0A386KP63	3893	['Ami_2', 'CW_7', 'CW_7', 'CW_7']
A0A221J6E1	3894	['Ami_2', 'CW_7', 'CW_7', 'CW_7']
A0A222ZJ03	3895	['Ami_2', 'CW_7', 'CW_7', 'CW_7']
A0A222ZGI4	3896	['Ami_2', 'CW_7', 'CW_7', 'CW_7', 'CW_7']
A0A0U4K7B3	3897	['Ami_2', 'CW_7', 'CW_7', 'CW_7', 'CW_7']
A0A218M5S7	3898	['Ami_2', 'CW_7', 'CW_7', 'CW_7', 'CW_7']
A0A345KUG3	3899	['Ami_2', 'CW_7', 'CW_7', 'CW_7', 'CW_7']
A0A1B0WN79	3900	['Ami_2']
A0A1B0WNC7	3901	['Ami_2']
I3PV40	3902	['Ami_2', 'SH3']
I3PV70	3903	['Ami_2', 'SH3']
A0A343X847	3904	['Ami_2', 'SH3']
A0A0A7RTQ5	3905	['Ami_2']
A0A0A7RVQ3	3906	['Ami_2']
H9YSE7	3907	['Ami_2']
A0A455ZIK7	3908	['Ami_2']
W8CPL1	3909	['Ami_2']
A0A0M4S688	3910	['Ami_2', 'Ami02_C']
A0A3Q8I6A1	3911	['Ami_2']
W0FBY3	3912	['Ami_2']
A0A088FLK9	3913	['Ami_2']
A0A345MKE4	3914	['Ami_2', 'CW_1', 'CW_1', 'CW_1', 'CW_1', 'CW_1', 'CW_1']
I6PBG3	3915	['Ami_2']
A0A219VG62	3916	['Ami_2']
A0A1B1SDV2	3917	['Ami_2', 'ZoocinA_TRD']
A0A3G1I5S8	3918	['Ami_2']
Q6PVM5	3919	['PET_M15']
A0A1C8HUE2	3920	['PET_M15']
A0A2I7S6J0	3921	['PET_M15']
A0A2I7RVH5	3922	['PET_M15']
A0A2I7S030	3923	['PET_M15']

A0A2I7RAI7	3924	['PET_M15']
R9TNK7	3925	['PET_M15']
M1NW24	3926	['PET_M15']
A0A4Y5TPB3	3927	['PET_M15']
A0A3G8FE65	3928	['PET_M15']
F1BUL3	3929	['PET_M15']
A0A3G8FED8	3930	['PET_M15']
A0A386KPD5	3931	['SLT_related']
A0A2Z4QAY5	3932	['SLT_related']
A0A2Z4QBH3	3933	['SLT_related']
A0A2Z4QC88	3934	['SLT_related']
A0A345L2B9	3935	['SLT_related']
A0A516KPH5	3936	['PET_M15']
A0A2Z4Q640	3937	['PET_M15']
A0A481VXF8	3938	['PET_M15']
A0A2Z4Q7L4	3939	['PET_M15']
A0A2U8UPE1	3940	['PET_M15']
A0A4Y5NZW2	3941	['PET_M15']
A0A345MHX4	3942	['PET_M15']
A0A4Y6ELZ9	3943	['PET_M15']
A0A516KUS8	3944	['PET_M15']
A0A2U8UHV1	3945	['PET_M15']
A0A4P8MUL3	3946	['PET_M15']
A0A2U8UIP4	3947	['PET_M15']
A0A516KV17	3948	['PET_M15']
A0A2Z4QC35	3949	['CHAP']
A0A2Z4QBA7	3950	['CHAP']
A0A2Z4QBI6	3951	['CHAP']
A0A2Z4QC13	3952	['CHAP']
A0A345L2C1	3953	['CHAP']
A0A386KQY2	3954	['CHAP']
A0A2U8UMZ7	3955	['Ami_2']
A0A2H4PQW8	3956	['Ami_2']
A0A2H4PI36	3957	['Ami_2']
A0A2H5BMS6	3958	['Ami_2']
A0A2H5BLW9	3959	['Ami_2']
A0A2H5BMB2	3960	['Ami_2']
A0A2U8UNJ8	3961	['Ami_2']
A0A1J0MCQ0	3962	['Ami_2']
A0A386KNX3	3963	['Ami_2']
A0A0M3LSH2	3964	['GH108', 'PG_3']
A0A2H4J0Z0	3965	['GH108', 'PG_3']
Q775E1	3966	['GH108', 'PG_3']
K4NWI7	3967	['GH108', 'PG_3']
A0A4P2VP99	3968	['GH108', 'PG_3']
A0A0H4ILU0	3969	['GH108', 'PG_3']

A0A1L7R181	3970	['GH108', 'PG_3']
A0A1L7QNW6	3971	['GH108', 'PG_3']
F4N9V8	3972	['GH108']
A0A088FAJ9	3973	['GH108']
A0A4D6DZ51	3974	['GH108']
J7FAQ2	3975	['GH108', 'PG_3']
A0A482MF77	3976	['GH108', 'PG_3']
M1IDQ5	3977	['GH108', 'PG_3']
A0A1V0DY74	3978	['GH108', 'PG_3']
M4QPK9	3979	['GH108', 'PG_3']
A0A1X9HX89	3980	['GH108', 'PG_3']
A0A345N008	3981	['GH108', 'PG_3']
M5AAF4	3982	['GH108', 'PG_3']
A0A345MT49	3983	['GH108']
A0A2I7R9L5	3984	['GH108', 'PG_3']
A0A3T0IEM0	3985	['GH108', 'PG_3']
G9BW83	3986	['GH108']
A0A0H4SQ70	3987	['GH108']
A0A1Y0T0G4	3988	['GH108']
A0A219YFC4	3989	['GH108']
A0A223W052	3990	['GH108']
X4YH20	3991	['Ami_2']
X4YWB9	3992	['Ami_2']
X4YES5	3993	['Ami_2', 'SH3']
X4YGT6	3994	['Ami_2', 'SH3']
B2BTN7	3995	['Ami_2']
A0A4Y5MXB7	3996	['Ami_2']
A0A2K9V4C5	3997	['PET_M15', 'PG_1']
A0A2K9V3Q0	3998	['PET_M15', 'PG_1']
A0A2S1PEK6	3999	['PET_M15']
A0A219YB38	4000	['PET_M15']
A0A2H4F2T7	4001	['PET_M15']
W6AT95	4002	['PET_M15']
A0A2I7RGX8	4003	['PET_M15']
C3VVT1	4004	['PET_M15']
A0A384WJ38	4005	['PET_M15']
A0A1X9Q107	4006	['PET_M15']
A0A1X9Q0X5	4007	['PET_M15']
A0A1X9Q111	4008	['PET_M15']
A0A2K9V3B7	4009	['PET_M15']
A0A2K9V3X1	4010	['PET_M15']
I6R9L6	4011	['PET_M15']
A0A2S1GTR8	4012	['PET_M15']
A0A0B5A6U7	4013	['PET_M15']
A0A0B5A6N7	4014	['PET_M15']
A0A514CUV6	4015	['PET_M15']

A0A514CV07	4016	['PET_M15']
S5VVA0	4017	['PET_M15']
C7BGD8	4018	['PET_M15']
R4JMG7	4019	['PET_M15']
D6PEK2	4020	['PET_M15']
A0A345BNM2	4021	['PET_M15']
B5TR94	4022	['PET_M15']
A0A345N1W6	4023	['PET_M15']
A0A384UIY2	4024	['PET_M15']
A0A514AB28	4025	['PET_M15']
M1I7M3	4026	['PET_M15']
A0A1B1IRA1	4027	['PET_M15']
A0A0K1LKR7	4028	['PET_M15']
A0A0K1LLY2	4029	['PET_M15']
A0A2I7RZD8	4030	['PET_M15']
A0A2I7RH38	4031	['PET_M15']
A0A2I7QJL4	4032	['PET_M15']
A0A2I7RQK6	4033	['PET_M15']
M4T418	4034	['PET_M15']
A0A1J0GV68	4035	['PET_M15']
A0A2I7QMI2	4036	['PET_M15']
A0A1D9C9S9	4037	['PET_M15']
A0A2I7RNV9	4038	['PET_M15']
A0A1J0GWE8	4039	['PET_M15']
A0A3G8F2K6	4040	['PET_M15']
A0A2H5BQ54	4041	['PET_M15']
W6E9J9	4042	['PET_M15']
A0A1W6JTJ9	4043	['PET_M15']
A0A7I2	4044	['PET_M15']
I6R9P9	4045	['PET_M15']
A0A3G3IQS1	4046	['PET_M15']
A0A2I6QQW2	4047	['ZoocinA_TRD']
A0A3G8FCB4	4048	['ZoocinA_TRD']
A0A2P0VGC2	4049	['Ami_5', 'ZoocinA_TRD']
A0A3S5H244	4050	['Ami_5', 'ZoocinA_TRD']
Q9T1D1	4051	['Ami_5', 'ZoocinA_TRD']
Q9T1C9	4052	['Ami_5', 'ZoocinA_TRD']
B3GW20	4053	['Ami_5', 'ZoocinA_TRD']
A0A4D6A9R7	4054	['Ami_5', 'ZoocinA_TRD']
A0A3G8FCV4	4055	['Ami_5', 'ZoocinA_TRD']
A0A516KPW1	4056	['CHAP']
A0A4D6A5T7	4057	['CHAP']
Q6DMS8	4058	['CHAP']
A0A4D6AEM8	4059	['CHAP']
A0A1P8L6B4	4060	['SH3']
A0A4D6BB83	4061	['CHAP', 'SH3']

A0A4D6ANN1	4062	['CHAP', 'SH3']
A0A4D6BC88	4063	['Transposase_InsH_N', 'DDE_Tnp_1_6', 'CHAP', 'SH3']
A0A4D5ZZW3	4064	['CHAP']
A0A4D6ARN8	4065	['CHAP']
D3W0F3	4066	['CHAP', 'SH3']
A0A0K0MWL2	4067	['CHAP']
A0A514TYX9	4068	['PET_M23']
A0A3G2KGI5	4069	['PET_M23']
A0A3G2KI32	4070	['PET_M23']
A0A3G2KJ82	4071	['PET_M23']
A0A3G2KE64	4072	['PET_M23']
A0A4D6BFW0	4073	['Ami_5']
A0A288TY66	4074	['Ami_5', 'SH3']
A0A4Y5TRC1	4075	['Ami_5', 'SH3']
A0A411B7H2	4076	['Ami_5', 'SH3']
A0A068EP83	4077	['Ami_2', 'SH3', 'SH3']
S5M947	4078	['Ami_2', 'SH3', 'SH3']
S5M4J3	4079	['Ami_2', 'SH3', 'SH3']
A0A3G3BVI7	4080	['Ami_2', 'SH3', 'SH3', 'SPOR']
D6QWM6	4081	['Ami_2']
A0A0U4J8N7	4082	['Ami_2']
S6BFI4	4083	['Ami_2', 'LysM']
O64040	4084	['Ami_2', 'SH3', 'SH3']
A0A1P8CWN6	4085	['Ami_2', 'SH3', 'SH3']
A0A0B5CTW5	4086	['Ami_2', 'PSA_CBD']
A0A059T7X1	4087	['Ami_2', 'PSA_CBD']
A0A0B5D123	4088	['Ami_2', 'PSA_CBD', 'PSA_CBD']
Q8W5Y8	4089	['Ami_3', 'PSA_CBD']
A8ATJ1	4090	['Ami_3', 'PSA_CBD', 'PSA_CBD']
Q3T4Y6	4091	['MUR']
Q6EDY3	4092	['MUR']
Q3T548	4093	['MUR']
Q3T4S4	4094	['MUR']
A0A0K2FI39	4095	['SLT_related']
A0A2H4GYF1	4096	['SLT_related']
A0A0S0N5R0	4097	['SLT_related']
A0A172PZR7	4098	['SLT_related']
A0A2H4GY46	4099	['SLT_related']
V9LZ47	4100	Unknown
A0A1Q2U346	4101	['SLT_related']
A0A2I7QI01	4102	Unknown
A0A514A2V6	4103	['SLT_related']
Q2Z0P3	4104	['EF_HAND_2']
A0A223LI74	4105	Unknown
A0A223LIX2	4106	Unknown
A0A411CWC0	4107	['PET_M23']

A0A1L4BKS4	4108	['PET_M23']
A7XXE4	4109	['PET_M23']
A7XXT9	4110	['PET_M23']
I7DKS9	4111	['PG_1', 'SLT_related']
A0A192Y5C9	4112	['PG_1', 'SLT_related']
A0A2D1GR13	4113	['PG_1', 'SLT_related']
A0A2Z4QBH5	4114	['NLPC_P60']
A0A2Z4QBQ0	4115	['NLPC_P60']
A0A345GTN0	4116	['NLPC_P60']
A0A345L231	4117	['NLPC_P60']
A0A0A0RLD4	4118	['NLPC_P60']
A0A222YZ81	4119	['NLPC_P60']
A0A514DK65	4120	['NLPC_P60']
A0A514U234	4121	['NLPC_P60']
A0A2U8UQZ9	4122	['NLPC_P60']
A0A2U8UQM8	4123	['NLPC_P60']
A0A1J0GVU1	4124	['NLPC_P60']
A0A345M702	4125	['NLPC_P60']
R9TEW4	4126	['SLT_related']
A0A0D4DBF6	4127	['SLT_related']
A0A126HGE4	4128	['SLT_related']
Q6WHM4	4129	['SLT_related']
I6W7B3	4130	['SLT_related']
A0A2I7RD74	4131	['SLT_related']
A0A2I7QXY7	4132	Unknown
A0A2I7R1F1	4133	Unknown
A0A088C3L3	4134	['SLT_related']
E1A2G3	4135	['SLT_related']
A0A291LDQ3	4136	['SLT_related']
E5AFT8	4137	['SLT_related']
A0A1W6DYG9	4138	Unknown
A0A4P8W7P5	4139	['PET_M15']
A0A4P8WA06	4140	['PET_M15']
A0A4P8W691	4141	['PET_M15']
A0A4P8W6W7	4142	['PET_M15']
A0A4P8W6K2	4143	['PET_M15']
A0A4P8W628	4144	['PET_M15']
A0A4P8W6M2	4145	['PET_M15']
A0A345BNG5	4146	['PET_M15']
A0A4V1FW13	4147	['PET_M15']
A0A4P8W763	4148	['PET_M15']
A0A4P8W735	4149	['PET_M15']
A0A4P8WA22	4150	['PET_M15']
A0A345BQC9	4151	['PET_M15']
A0A4P8W6X2	4152	['PET_M15']
A0A345MW16	4153	['PET_M15']

A0A3G2YSX9	4154	['PET_M15']
Q37976	4155	['PET_M15']
A0A059T7Y8	4156	['PET_M15']
A8ATB8	4157	['PET_M15', 'SH3', 'SH3']
S5YC98	4158	['PET_M15']
A0A2H4J236	4159	['PET_M15', 'PG_1']
F8WPX5	4160	['PET_M15', 'PG_1']
W8EEW7	4161	['PET_M15', 'PG_1']
A0A0E3T6C0	4162	['PET_M15', 'PG_1']
U5Q1E4	4163	['PET_M15', 'PG_1']
A0A0E3JT47	4164	['PET_M15', 'PG_1']
A8ATR6	4165	['PET_M15', 'PSA_CBD', 'PSA_CBD']
A0A059T5J4	4166	['PET_M15', 'PSA_CBD', 'PSA_CBD']
A0A127AWA8	4167	['PET_M15', 'PG_1', 'PG_1']
A0A3S9X9Y9	4168	['PET_M15', 'PG_1']
A0A217EQM4	4169	['PET_M15', 'PG_1', 'PG_1']
A0A2H4J4U2	4170	['PET_M15', 'PG_1']
C8CHK7	4171	['PET_M15']
A0A088C4T8	4172	['PET_M15']
A0A0A7HAX7	4173	['PET_M15']
A0A2D0Z549	4174	['PET_M15']
A0A2D0YYE9	4175	['PET_M15']
R9ZXQ6	4176	['Ami_3']
R9ZZI3	4177	['Ami_3']
S0A0K5	4178	['Ami_3']
A0A0E3XA78	4179	['DUF1906']
A0A0E3T822	4180	['DUF1906']
A0A0K0NKU4	4181	['DUF1906']
A0A1B3B0M0	4182	['DUF1906']
C4NTF2	4183	['Ami_2']
C4NT73	4184	['Ami_2']
A0A345AYL4	4185	['Ami_2']
A0A191VYM4	4186	['Ami_2']
A0A2Z4QHJ1	4187	['Ami_2']
A0A3T0IK12	4188	['Ami_2']
A0A3Q9R8T9	4189	['Ami_2']
Q8W6M2	4190	['Ami_2']
A0A068CC96	4191	['Ami_2']
A0A2H4PGK4	4192	['SLT_related']
A0A0K1YAG1	4193	['SLT_related']
A0A0K1YA75	4194	['SLT_related']
K4PAU3	4195	['SLT_related']
A0A1J0GQA6	4196	['SLT_related']
A0A2L1IVL5	4197	['SLT_related']
A0A345MG85	4198	Unknown
A0A345M8G7	4199	Unknown

A0A514U1R7	4200	['SLT_related']
A0A0A0RLZ4	4201	['SLT_related']
A0A221SAT8	4202	Unknown
A0A0M3MYZ4	4203	['CHAP']
A0A3Q8L7S6	4204	['CHAP']
H6WTZ1	4205	['CHAP']
A0A140G6T8	4206	['PET_M23']
A0A222ZJJ6	4207	['PET_M23']
A0A222Z8D1	4208	['PET_M23']
A0A4D6B2Q1	4209	['GLUCO']
A0A4D6A666	4210	['GLUCO']
S5MAF1	4211	['GLUCO']
U5P0V3	4212	['GLUCO']
S5M9Y4	4213	['GLUCO', 'Ami_3']
A0A0A7RUN1	4214	['GLUCO']
A0A1P8VWC2	4215	['GLUCO']
A0A2K9V347	4216	['GLUCO']
G3MB92	4217	['Ami_3', 'LysM', 'GLUCO']
G3MB93	4218	['Ami_3', 'LysM']
D6QWP2	4219	['Ami_3', 'SPOR']
D6QWP3	4220	['Ami_3', 'SPOR']
D6QWM8	4221	['Ami_3', 'SPOR']
A0A0U4JE28	4222	['Ami_3']
D6QWM9	4223	['Ami_3', 'SPOR']
D6QWN0	4224	['Ami_3', 'SPOR']
A0A3B6XHD1	4225	['Ami_3']
A0A2H4JCM7	4226	['Ami_3', 'GLUCO']
A0A3G3BWR1	4227	['Ami_3', 'SH3']
S0A148	4228	['Ami_3']
A0A2P1JU23	4229	['Ami_3']
A0A345ASR5	4230	['Ami_3']
A0A0N9SGH1	4231	['Ami_3']
A0A345AVF2	4232	['Ami_3']
A0A481W382	4233	['Ami_3']
A0A059WLJ5	4234	['Ami_3']
A0A067ZJB6	4235	['Ami_3']
A0A3G3BW17	4236	['Ami_3']
A0A482CXV9	4237	['Ami_3']
O48471	4238	['Ami_3']
M4ZRP4	4239	['Ami_3']
A0A0K1LMD7	4240	['Ami_3', 'SH3']
D6QWM7	4241	['Ami_3', 'SPOR']
E5DV68	4242	['Ami_3', 'SPOR']
A0A290GJY1	4243	['Ami_3', 'SPOR']
Q9FZW0	4244	['Ami_3', 'LysM']
A0A0N6W8I1	4245	['Ami_3', 'LysM', 'LysM']

S5M842	4246	['Ami_3', 'Ami02_C']
A0A1B1P7G0	4247	['Ami_3', 'Ami02_C']
A0A1D6X870	4248	['Ami_3']
A0A3Q9R7T7	4249	['Ami_3', 'Ami02_C']
A0A2I7QIN4	4250	['Ami_3']
A0A346FK38	4251	['Ami_3']
P89923	4252	['Ami_3']
I1TLF9	4253	['Ami_3']
Q5ILA1	4254	['Ami_3', 'SPOR']
B6RT63	4255	['Ami_3', 'Ami02_C']
A0A0A8WIP6	4256	['Ami_3']
A0A2R2ZGL3	4257	['Ami_3']
A0A2H4J8A3	4258	['Ami_3', 'SH3']
Q24LG3	4259	['Ami_3']
I1TJX3	4260	['Ami_3']
A0A1L2BYB1	4261	['Ami_3']
A0A2D0WXW5	4262	['Ami_3']
F8UBI4	4263	['Ami_3', 'SPOR']
F8UBD2	4264	['Ami_3']
A0A385ISV1	4265	['Ami_3']
Q8SBN4	4266	['Ami_3']
A0A0A7RUJ1	4267	['Ami_3', 'SPOR']
R9R4A9	4268	['Ami_3']
A0A0B5GYD7	4269	['Ami_3']
A0A2I7S6C9	4270	['Ami_3']
A0A2I7R243	4271	['Ami_3']
A0A0U4I901	4272	['Ami_3']
A0A4P8NLL7	4273	['Ami_3']
E7D0G0	4274	['Ami_3']
A0A1J0GUZ6	4275	['Ami_3']
A0A2P9HY81	4276	['Ami_3']
U5PZX6	4277	['Ami_3']
E5EYD9	4278	['Ami_3']
D9I5X4	4279	['Ami_3']
E5EPF0	4280	['Ami_3']
A0A2R4A182	4281	['PET_M15']
A0A516KTN2	4282	['PET_M15', 'Ami_2']
A0A4D6T6X9	4283	['PET_M15']
A0A2R4A103	4284	['PET_M15']
A0A514DIJ5	4285	['PET_M15']
A0A3G2KD16	4286	['PET_M15', 'PG_1']
A0A3G2KA78	4287	['PET_M15', 'PG_1']
A0A3G2KDB4	4288	['PET_M15', 'PG_1']
A0A2R3ZZI6	4289	['PET_M15']
A0A2Z4Q7D8	4290	['PET_M15']
A0A1J0GVU2	4291	['SLT_related', 'LysM']

A0A2D0ZMX2	4292	['SLT_related']
A0A1I9SA30	4293	['SLT_related']
A0A2U8UJJ8	4294	['SLT_related']
A0A2U8ULA9	4295	['SLT_related']
Q5GQI4	4296	['PET_C39']
I3ULH9	4297	['PET_C39']
I3ULY1	4298	['PET_C70', 'GLUCO']
A0A096VKH0	4299	['PET_C70', 'GLUCO']
C7F4E4	4300	['GLUCO', 'PET_C70']
G8CLC4	4301	['GLUCO', 'PET_C70']
A0A0C4K626	4302	['GLUCO', 'PET_C70']
A0A1L5C2A4	4303	['Pesticin_C']
A0A2H5BFZ4	4304	['Pesticin_C']
M4PYB4	4305	['Pesticin_C']
A0A2I7RBM4	4306	['Pesticin_C']
A0A2P0VGB4	4307	['Ami_5']
A0A3G6JGJ4	4308	['GH25']
A0A514TXN1	4309	['PET_M23']
A0A514TX97	4310	['PET_M23']
A0A3Q9RAN6	4311	['PET_M23', 'PG_1']
A0A4Y6EB99	4312	['PET_M23', 'PG_1']
A0A386KPJ2	4313	['PET_M23', 'PG_1']
A0A4D6T9C0	4314	['PET_M23']
A0A4Y6EE89	4315	['PET_M23']
A0A2U8UQJ7	4316	['Ami_2', 'CW_7', 'CW_7']
A0A2U8UQU9	4317	['Ami_2', 'CW_7', 'CW_7']
A0A4D6B3C2	4318	['Ami_2', 'CW_7', 'CW_7', 'CW_7']
A0A4D6AP61	4319	['Ami_2', 'CW_7', 'CW_7']
A0A4D6A2G5	4320	['Ami_2', 'CW_7', 'CW_7']
A0A2K9V338	4321	['Ami_2', 'SH3']
H7BW58	4322	['Ami_2']
D6QWN4	4323	['Ami_2', 'SPOR']
H7BWC9	4324	['Ami_2', 'LysM']
A0A2H4PI22	4325	Unknown
D6QWN2	4326	['Ami_2', 'LysM']
A0A222ZES2	4327	['LysM', 'LysM']
A0A1D8EUA5	4328	['Ami_2', 'PG_1']
A0A1D8ETE4	4329	['Ami_2', 'PG_1']
A0A1D8ET80	4330	['Ami_2', 'PG_1']
A0A1W6JRQ3	4331	['Ami_2']
A0A1L6BZH5	4332	['Ami_2']
A0A2P1JXS5	4333	['DUF1906', 'Ami_2', 'DUF4185']
A0A0K0NKS2	4334	['DUF1906', 'Ami_2', 'PET_M23', 'DUF4185']
A0A3S9UC96	4335	['DUF1906', 'Ami_2', 'PET_M23', 'DUF4185']
A0A4D6BGJ6	4336	['GH25', 'CW_7', 'CW_7']
A0A2K5B2A3	4337	['GH25', 'CW_7', 'CW_7']

A0A0A1ERA5	4338	['GH25', 'SH3']
Q20DB2	4339	['GH25']
Q9AZ81	4340	['GH25', 'SH3']
A0A173G9G9	4341	['GH25']
A0A1D8ETQ2	4342	['GH25']
A0A1D8ETP4	4343	['GH25']
A0A2P1CIS8	4344	['GH25', 'CW_7', 'LysM']
A0A2P1CIJ7	4345	['GH25', 'CW_7', 'LysM']
I3VYU7	4346	['GH25', 'PG_1']
A0A4D6AST6	4347	['GH25', 'LysM', 'LysM']
A0A141HSE6	4348	['GH25', 'LysM', 'LysM', 'LysM']
Q0SPG7	4349	['GH25', 'SH3', 'SH3']
I2E8W9	4350	['GH25', 'SH3', 'SH3']
A0A2Z2UC06	4351	['GH25']
D6PSS2	4352	['GH25']
A0A4Y5FE66	4353	['GH25']
B8R665	4354	['GH25']
A0A411CQD4	4355	['GH25', 'LysM', 'LysM']
F0PIJ7	4356	['GH25']
A0A2P0ZLG2	4357	['GH25']
A0A2K9V3I9	4358	['GH25']
D9ZNF3	4359	['GH25']
D0R7H8	4360	['GH25']
A0A1U9WQS3	4361	['GH25', 'LysM', 'LysM', 'LysM']
B6D7J9	4362	['GH25', 'SH3', 'LysM']
A0A345MZG2	4363	['GH25', 'CW_7']
A0A4P8W8G2	4364	['GH25']
A0A514DHM4	4365	['PET_M23']
A0A514DI35	4366	['PET_M23']
A0A2Z4Q731	4367	['PET_M23']
A0A514DDD7	4368	['PET_M23']
A0A386KMI0	4369	['PET_M23']
A0A2Z4Q5M5	4370	['PET_M23', 'PG_1', 'PG_1']
A0A2Z4QAM1	4371	['PET_M23', 'PG_1', 'PG_1']
A0A3G3M0S2	4372	['PET_M23']
A0A3G3LY09	4373	['PET_M23']
A0A3G2KFZ3	4374	['PET_M23']
A0A3G3M3K7	4375	['PET_M23']
A0A075BS18	4376	['GH24', 'PET_M23']
L0LC03	4377	['PET_M23']
A0A076G6R1	4378	['PET_M23']
A0A1B0XUH3	4379	['PET_M23']
G9BW84	4380	['PET_M23']
A0A2K9VGT1	4381	['PET_M23']
G9FHH0	4382	['SLT_related', 'PET_M23', 'LysM']
A0A3G2KJ81	4383	['PET_M23', 'LysM']

A0A3G2KCW1	4384	['PET_M23', 'PG_1']
A0A4D6T839	4385	['PET_M23']
A0A3G2KEZ6	4386	['PET_M23']
A0A411CQK1	4387	['PET_M23']
A0A3G2KFM1	4388	['PET_M23', 'Ami_2']
A0A3G3M6D6	4389	['PET_M23']
A0A3G2KJH4	4390	['PET_M23']
A0A3G3M3C4	4391	['PET_M23']
V5R999	4392	['PET_M23']
A0A3G2KHR7	4393	['PET_M23']
A0A386KQ61	4394	['PET_M23', 'PG_1']
W8P064	4395	['PET_M23']
A0A3G3LYQ8	4396	['NLPC_P60', 'PET_M23']
A0A292GAV4	4397	['PET_M23']
Q859T2	4398	['PET_M23']
A0A411CVX5	4399	['PET_M23']
A0A451ED88	4400	['PET_M23']
A0A1S5RCR7	4401	['LysM', 'SLT_related']
A0A2Z2E9B5	4402	['LysM', 'SLT_related']
A0A0A7DN24	4403	['LysM', 'SLT_related']
A0A3G6JGZ9	4404	['LysM', 'SLT_related']
A0A3G1I5V6	4405	['LysM', 'SLT_related']
A0A4Y5FEU0	4406	['LysM', 'SLT_related']
L7TMU6	4407	['GH24']
L7TJA9	4408	['GH24']
A0A1B2IGM4	4409	['SLT_related']
R4IFH0	4410	['SLT_related']
A0A1B2ICU1	4411	['SLT_related']
A0A1B2IEY6	4412	['SLT_related']
A0A482IHN6	4413	['SLT_related']
A0A2H5BHL8	4414	['SLT_related']
A0A1S6L1V1	4415	['SLT_related']
W8CZD4	4416	['SLT_related']
A0A289ZW82	4417	Unknown
A0A4Y5TNW4	4418	['SLT_related']
A0A3T0INY9	4419	['PG_1']
A0A0R8VCQ3	4420	['Ami_2']
A0A3T0IDD9	4421	['Ami_2', 'PG_1']
A0A142F1N7	4422	['Ami_2', 'PG_1', 'PG_1']
A0A3G3BWD6	4423	['Ami_2', 'LysM', 'LysM', 'PG_1']
A0A0S2SXM5	4424	['Ami_2', 'LysM', 'PG_1']
A0A3G3BWP3	4425	['Ami_2', 'LysM', 'LysM', 'SH3']
A0A1B0T6L3	4426	['Ami_2']
A0A1W6DX33	4427	['Ami_2', 'SH3']
A0A1B0UZW5	4428	['Ami_2', 'SH3']
A0A2Z4QGE6	4429	['Ami_2', 'SH3']

A0A0B5A5A2	4430	['PET_M15']
F4YXS2	4431	['PG_1', 'PET_M15']
A0A0K0PVI5	4432	['PG_1', 'PET_M15']
A0A2I7RPW4	4433	['NLPC_P60']
A0A2I7QVY1	4434	['NLPC_P60']
M4Q149	4435	['NLPC_P60']
A0A3Q9R5V9	4436	['NLPC_P60']
A0A220YL75	4437	['GH24']
A0A2I7RY31	4438	['GH24']
D7R9U1	4439	['PET_U40']
A0A1B0WKU4	4440	['GH108', 'PG_3']
Q9MC03	4441	['GH108']
A0A4E0W528	4442	['GH108']
A0A3G2KA11	4443	['Ami_2']
A0A3G2KER3	4444	Unknown
A0A0U4B3G8	4445	Unknown
A0A249XLH4	4446	Unknown
A0A3G3BY37	4447	['Ami_2', 'LysM', 'PG_1']
A0A2Z3EAE0	4448	['Ami_2', 'SH3', 'SH3']
D6QWN1	4449	['Ami_2']
A0A0K2CPP8	4450	['Ami_2']
A0A3G2KAD5	4451	['CHAP', 'LysM']
H7BV84	4452	['GLUCO', 'CHAP', 'CW_7', 'CW_7']
A0A509ER44	4453	['CHAP']
G8EY10	4454	['NLPC_P60']
M4SNU1	4455	['NLPC_P60']
M4QR76	4456	['SLT_related']
A0A345M6Y7	4457	['SLT_related']
A0A386K7V4	4458	['SLT_related']
A0A1J0GVV1	4459	['Ami_2']
A0A1B0XUI0	4460	['PET_M15']
A0A1B0XTX0	4461	['PET_M15']
A0A1B0XUE6	4462	['PET_M15']
A0A514D6Z6	4463	['MUR']
A0A514D4K4	4464	['MUR']
F5B3Y2	4465	['YkuD']
H2BCV4	4466	['GLUCO', 'YkuD']
A0A2Z2GUJ4	4467	['CHAP']
A0A3G3BXK0	4468	Unknown
I6R1D5	4469	['Ami_3']
D1LV09	4470	['GH24']
F4YCR3	4471	['GH24']
A0A482IE72	4472	['GH24']
R9TQ04	4473	['Ami_2', 'Cu_amine_oxidN1']
A0A249XNN9	4474	['Ami_2']
Q24LH4	4475	['3D', 'NLPC_P60']

A0A345M6Y6	4476	['NLPC_P60']
A0A1V0DYB6	4477	['NLPC_P60']
A0A345BR96	4478	['NLPC_P60']
Q9FZT9	4479	['SLT_related']
A0A1U9AK71	4480	['PG_3', 'SLT_related']
U5PT78	4481	['PET_M15']
V5Q8V7	4482	['PROKAR_LIPOPROTEIN', 'pesticin_lyz_like2', 'GH24']
Q94ML5	4483	['SLT_related', 'AA_TRNA_LIGASE_II_GLYAB']
C0KIU7	4484	['GLUCO']
A0A2P1CIB7	4485	['CHAP']
A0A345AR88	4486	['Ami_5']
G4KKR9	4487	['SLT_related']
Q859R5	4488	['SLT_related']
C8CHM9	4489	['SLT_related']
Q7Y4B2	4490	['SLT_related']
A0A345API3	4491	['SLT_related']
A0A0A8J856	4492	['SLT_related']
A0A1B0XUK8	4493	Unknown
A0A1B0XTZ1	4494	['SLT_related']
A0A1B0XUA8	4495	['SLT_related']
A0A3G1GLJ4	4496	Unknown
G4W937	4497	['SLT_related']
A0A2H4PJ51	4498	['YkuD']
A0A3Q8HD45	4499	['Ami_2', 'LysM', 'LysM']
J9PUD3	4500	['GLUCO']
A0A2Z4QA43	4501	['Ami_2', 'SH3']
A0A2K9VH44	4502	['Ami_2', 'GLUCO']
H7BVK7	4503	['Ami_2', 'GLUCO']
H7BUY1	4504	['SLT_related', 'Ami_2', 'SLH']
A0A345BPY8	4505	['SLT_related', 'Ami_2', 'SH3']
A0A2K9V2U2	4506	['SLT_related', 'Ami_2', 'SPOR']
H7BUU5	4507	['SLT_related']
H7BV73	4508	['PROKAR_LIPOPROTEIN', 'SLT_related', 'PG_1']
Q9FZW2	4509	['SLT_related']
A0A2Z4GUC1	4510	['Ami_5', 'SLT_related']
A0A1P8VV77	4511	['GH25']
A0A0U4JCL8	4512	['CHAP']
F0PIL0	4513	['SLT_related']
Q7Y3F1	4514	['CHAP']
A0A4D6DSD5	4515	['CHAP']

Host phylum	Host class	Host order
Actinobacteria	Actinobacteria	Corynebacteriales
Actinobacteria	Actinobacteria	Corynebacteriales
Actinobacteria	Actinobacteria	Corynebacteriales
Actinobacteria	Actinobacteria	Corynebacteriales
Actinobacteria	Actinobacteria	Corynebacteriales
Actinobacteria	Actinobacteria	Corynebacteriales
Actinobacteria	Actinobacteria	Corynebacteriales
Actinobacteria	Actinobacteria	Corynebacteriales
Actinobacteria	Actinobacteria	Corynebacteriales
Actinobacteria	Actinobacteria	Corynebacteriales
Actinobacteria	Actinobacteria	Corynebacteriales
Actinobacteria	Actinobacteria	Corynebacteriales
Actinobacteria	Actinobacteria	Corynebacteriales
Actinobacteria	Actinobacteria	Corynebacteriales
Actinobacteria	Actinobacteria	Corynebacteriales
Actinobacteria	Actinobacteria	Corynebacteriales
Actinobacteria	Actinobacteria	Corynebacteriales
Actinobacteria	Actinobacteria	Corynebacteriales
Actinobacteria	Actinobacteria	Corynebacteriales
Actinobacteria	Actinobacteria	Corynebacteriales
Actinobacteria	Actinobacteria	Corynebacteriales
Actinobacteria	Actinobacteria	Corynebacteriales
Actinobacteria	Actinobacteria	Corynebacteriales
Actinobacteria	Actinobacteria	Corynebacteriales
Actinobacteria	Actinobacteria	Corynebacteriales
Actinobacteria	Actinobacteria	Corynebacteriales
Actinobacteria	Actinobacteria	Corynebacteriales
Actinobacteria	Actinobacteria	Corynebacteriales
Actinobacteria	Actinobacteria	Corynebacteriales
Actinobacteria	Actinobacteria	Corynebacteriales
Actinobacteria	Actinobacteria	Corynebacteriales
Actinobacteria	Actinobacteria	Corynebacteriales
Actinobacteria	Actinobacteria	Corynebacteriales
Actinobacteria	Actinobacteria	Corynebacteriales
Unknown	Unknown	Unknown
Actinobacteria	Actinobacteria	Corynebacteriales
Actinobacteria	Actinobacteria	Corynebacteriales
Actinobacteria	Actinobacteria	Corynebacteriales
Actinobacteria	Actinobacteria	Corynebacteriales
Actinobacteria	Actinobacteria	Corynebacteriales
Actinobacteria	Actinobacteria	Corynebacteriales
Actinobacteria	Actinobacteria	Corynebacteriales
Actinobacteria	Actinobacteria	Corynebacteriales
Actinobacteria	Actinobacteria	Corynebacteriales
Actinobacteria	Actinobacteria	Corynebacteriales

Actinobacteria	Actinobacteria	Corynebacteriales
Actinobacteria	Actinobacteria	Corynebacteriales
Actinobacteria	Actinobacteria	Corynebacteriales
Actinobacteria	Actinobacteria	Corynebacteriales
Actinobacteria	Actinobacteria	Corynebacteriales
Actinobacteria	Actinobacteria	Corynebacteriales
Actinobacteria	Actinobacteria	Corynebacteriales
Actinobacteria	Actinobacteria	Corynebacteriales
Actinobacteria	Actinobacteria	Corynebacteriales
Actinobacteria	Actinobacteria	Corynebacteriales
Actinobacteria	Actinobacteria	Corynebacteriales
Actinobacteria	Actinobacteria	Corynebacteriales
Actinobacteria	Actinobacteria	Corynebacteriales
Actinobacteria	Actinobacteria	Corynebacteriales
Actinobacteria	Actinobacteria	Corynebacteriales
Actinobacteria	Actinobacteria	Corynebacteriales
Actinobacteria	Actinobacteria	Corynebacteriales
Actinobacteria	Actinobacteria	Corynebacteriales
Actinobacteria	Actinobacteria	Corynebacteriales
Actinobacteria	Actinobacteria	Corynebacteriales
Actinobacteria	Actinobacteria	Corynebacteriales
Actinobacteria	Actinobacteria	Corynebacteriales
Actinobacteria	Actinobacteria	Corynebacteriales
Actinobacteria	Actinobacteria	Corynebacteriales
Actinobacteria	Actinobacteria	Corynebacteriales
Actinobacteria	Actinobacteria	Corynebacteriales
Actinobacteria	Actinobacteria	Corynebacteriales
Actinobacteria	Actinobacteria	Corynebacteriales
Actinobacteria	Actinobacteria	Corynebacteriales
Actinobacteria	Actinobacteria	Corynebacteriales
Actinobacteria	Actinobacteria	Corynebacteriales
Actinobacteria	Actinobacteria	Corynebacteriales
Actinobacteria	Actinobacteria	Corynebacteriales
Actinobacteria	Actinobacteria	Corynebacteriales
Actinobacteria	Actinobacteria	Corynebacteriales
Actinobacteria	Actinobacteria	Corynebacteriales
Actinobacteria	Actinobacteria	Corynebacteriales
Actinobacteria	Actinobacteria	Corynebacteriales
Actinobacteria	Actinobacteria	Corynebacteriales
Actinobacteria	Actinobacteria	Corynebacteriales
Actinobacteria	Actinobacteria	Corynebacteriales
Actinobacteria	Actinobacteria	Corynebacteriales
Actinobacteria	Actinobacteria	Corynebacteriales
Actinobacteria	Actinobacteria	Corynebacteriales
Actinobacteria	Actinobacteria	Corynebacteriales
Actinobacteria	Actinobacteria	Corynebacteriales

Actinobacteria	Actinobacteria	Corynebacteriales
Actinobacteria	Actinobacteria	Corynebacteriales
Actinobacteria	Actinobacteria	Corynebacteriales
Actinobacteria	Actinobacteria	Corynebacteriales
Actinobacteria	Actinobacteria	Corynebacteriales
Actinobacteria	Actinobacteria	Corynebacteriales
Actinobacteria	Actinobacteria	Corynebacteriales
Actinobacteria	Actinobacteria	Corynebacteriales
Actinobacteria	Actinobacteria	Corynebacteriales
Actinobacteria	Actinobacteria	Corynebacteriales
Actinobacteria	Actinobacteria	Corynebacteriales
Actinobacteria	Actinobacteria	Corynebacteriales
Actinobacteria	Actinobacteria	Corynebacteriales
Actinobacteria	Actinobacteria	Corynebacteriales
Actinobacteria	Actinobacteria	Corynebacteriales
Actinobacteria	Actinobacteria	Corynebacteriales
Actinobacteria	Actinobacteria	Corynebacteriales
Actinobacteria	Actinobacteria	Corynebacteriales
Actinobacteria	Actinobacteria	Corynebacteriales
Actinobacteria	Actinobacteria	Corynebacteriales
Actinobacteria	Actinobacteria	Micrococcales
Actinobacteria	Actinobacteria	Micrococcales
Actinobacteria	Actinobacteria	Micrococcales
Actinobacteria	Actinobacteria	Micrococcales
Actinobacteria	Actinobacteria	Micrococcales
Actinobacteria	Actinobacteria	Micrococcales
Actinobacteria	Actinobacteria	Micrococcales
Actinobacteria	Actinobacteria	Corynebacteriales
Actinobacteria	Actinobacteria	Corynebacteriales
Actinobacteria	Actinobacteria	Corynebacteriales
Actinobacteria	Actinobacteria	Corynebacteriales
Actinobacteria	Actinobacteria	Corynebacteriales
Unknown	Unknown	Unknown
Actinobacteria	Actinobacteria	Corynebacteriales
Actinobacteria	Actinobacteria	Corynebacteriales
Actinobacteria	Actinobacteria	Corynebacteriales
Actinobacteria	Actinobacteria	Corynebacteriales
Actinobacteria	Actinobacteria	Corynebacteriales
Actinobacteria	Actinobacteria	Corynebacteriales
Actinobacteria	Actinobacteria	Corynebacteriales
Actinobacteria	Actinobacteria	Corynebacteriales
Actinobacteria	Actinobacteria	Corynebacteriales
Actinobacteria	Actinobacteria	Corynebacteriales
Actinobacteria	Actinobacteria	Corynebacteriales
Actinobacteria	Actinobacteria	Corynebacteriales
Actinobacteria	Actinobacteria	Corynebacteriales

Actinobacteria	Actinobacteria	Corynebacteriales
Actinobacteria	Actinobacteria	Corynebacteriales
Actinobacteria	Actinobacteria	Corynebacteriales
Actinobacteria	Actinobacteria	Corynebacteriales
Actinobacteria	Actinobacteria	Corynebacteriales
Actinobacteria	Actinobacteria	Corynebacteriales
Actinobacteria	Actinobacteria	Corynebacteriales
Actinobacteria	Actinobacteria	Corynebacteriales
Actinobacteria	Actinobacteria	Corynebacteriales
Actinobacteria	Actinobacteria	Corynebacteriales
Actinobacteria	Actinobacteria	Corynebacteriales
Actinobacteria	Actinobacteria	Corynebacteriales
Actinobacteria	Actinobacteria	Corynebacteriales
Actinobacteria	Actinobacteria	Corynebacteriales
Actinobacteria	Actinobacteria	Corynebacteriales
Actinobacteria	Actinobacteria	Corynebacteriales
Actinobacteria	Actinobacteria	Corynebacteriales
Actinobacteria	Actinobacteria	Corynebacteriales
Actinobacteria	Actinobacteria	Corynebacteriales
Actinobacteria	Actinobacteria	Corynebacteriales
Proteobacteria	Betaproteobacteria	Burkholderiales
Proteobacteria	Gammaproteobacteria	Pseudomonadales
Proteobacteria	Betaproteobacteria	Burkholderiales
Bacteroidetes	Flavobacteriia	Flavobacteriales
Proteobacteria	Gammaproteobacteria	Vibrionales
Proteobacteria	Alphaproteobacteria	Caulobacterales
Proteobacteria	Alphaproteobacteria	Caulobacterales
Proteobacteria	Alphaproteobacteria	Sphingomonadales
Unknown	Unknown	Unknown
Proteobacteria	Gammaproteobacteria	Oceanospirillales
Proteobacteria	Gammaproteobacteria	Pseudomonadales
Proteobacteria	Alphaproteobacteria	Rhizobiales
Proteobacteria	Alphaproteobacteria	Rhizobiales
Proteobacteria	Alphaproteobacteria	Rhizobiales
Proteobacteria	Gammaproteobacteria	Pseudomonadales
Proteobacteria	Gammaproteobacteria	Aeromonadales
Cyanobacteria	Cyanophyceae	Synechococcales
Cyanobacteria	Cyanophyceae	Synechococcales
Unknown	Unknown	Unknown
Cyanobacteria	Cyanophyceae	Synechococcales
Cyanobacteria	Cyanophyceae	Synechococcales
Cyanobacteria	Cyanophyceae	Synechococcales
Proteobacteria	Gammaproteobacteria	Xanthomonadales
Proteobacteria	Gammaproteobacteria	Xanthomonadales
Proteobacteria	Gammaproteobacteria	Xanthomonadales
Proteobacteria	Gammaproteobacteria	Xanthomonadales

Proteobacteria	Gammaproteobacteria	Xanthomonadales
Proteobacteria	Gammaproteobacteria	Xanthomonadales
Proteobacteria	Gammaproteobacteria	Aeromonadales
Proteobacteria	Gammaproteobacteria	Enterobacterales
Proteobacteria	Gammaproteobacteria	Enterobacterales
Proteobacteria	Gammaproteobacteria	Enterobacterales
Proteobacteria	Gammaproteobacteria	Enterobacterales
Proteobacteria	Gammaproteobacteria	Enterobacterales
Proteobacteria	Gammaproteobacteria	Enterobacterales
Proteobacteria	Gammaproteobacteria	Enterobacterales
Proteobacteria	Gammaproteobacteria	Pseudomonadales
Proteobacteria	Gammaproteobacteria	Pseudomonadales
Proteobacteria	Gammaproteobacteria	Pseudomonadales
Unknown	Unknown	Unknown
Proteobacteria	Gammaproteobacteria	Pseudomonadales
Unknown	Unknown	Unknown
Cyanobacteria	Cyanophyceae	Chroococcales
Cyanobacteria	Cyanophyceae	Chroococcales
Proteobacteria	Alphaproteobacteria	Rhizobiales
Proteobacteria	Alphaproteobacteria	Sphingomonadales
Unknown	Unknown	Unknown
Unknown	Unknown	Unknown
Unknown	Unknown	Unknown
Bacteroidetes	Flavobacteriia	Flavobacteriales
Proteobacteria	Betaproteobacteria	Burkholderiales
Proteobacteria	Gammaproteobacteria	Enterobacterales
Proteobacteria	Gammaproteobacteria	Enterobacterales
Proteobacteria	Gammaproteobacteria	Enterobacterales
Proteobacteria	Gammaproteobacteria	Enterobacterales
Proteobacteria	Gammaproteobacteria	Enterobacterales
Proteobacteria	Gammaproteobacteria	Enterobacterales
Proteobacteria	Gammaproteobacteria	Enterobacterales
Proteobacteria	Gammaproteobacteria	Enterobacterales
Proteobacteria	Gammaproteobacteria	Enterobacterales
Proteobacteria	Gammaproteobacteria	Enterobacterales
Proteobacteria	Gammaproteobacteria	Enterobacterales
Proteobacteria	Gammaproteobacteria	Enterobacterales
Proteobacteria	Gammaproteobacteria	Enterobacterales
Proteobacteria	Gammaproteobacteria	Enterobacterales
Proteobacteria	Gammaproteobacteria	Enterobacterales
Proteobacteria	Gammaproteobacteria	Aeromonadales
Proteobacteria	Gammaproteobacteria	Enterobacterales
Proteobacteria	Gammaproteobacteria	Enterobacterales
Proteobacteria	Gammaproteobacteria	Enterobacterales
Unknown	Unknown	Unknown
Proteobacteria	Gammaproteobacteria	Enterobacterales

Proteobacteria	Gammaproteobacteria	Enterobacterales
Proteobacteria	Gammaproteobacteria	Enterobacterales
Proteobacteria	Gammaproteobacteria	Enterobacterales
Proteobacteria	Gammaproteobacteria	Pseudomonadales
Proteobacteria	Gammaproteobacteria	Enterobacterales
Proteobacteria	Betaproteobacteria	Burkholderiales
Proteobacteria	Gammaproteobacteria	Xanthomonadales
Proteobacteria	Gammaproteobacteria	Xanthomonadales
Proteobacteria	Gammaproteobacteria	Pseudomonadales
Proteobacteria	Gammaproteobacteria	Pseudomonadales
Proteobacteria	Gammaproteobacteria	Pseudomonadales
Proteobacteria	Gammaproteobacteria	Pseudomonadales
Proteobacteria	Gammaproteobacteria	Pseudomonadales
Proteobacteria	Gammaproteobacteria	Pseudomonadales
Proteobacteria	Gammaproteobacteria	Pseudomonadales
Proteobacteria	Gammaproteobacteria	Pseudomonadales
Proteobacteria	Gammaproteobacteria	Pseudomonadales
Proteobacteria	Gammaproteobacteria	Pseudomonadales
Unknown	Unknown	Unknown
Unknown	Unknown	Unknown
Unknown	Unknown	Unknown
Proteobacteria	Gammaproteobacteria	Pseudomonadales
Proteobacteria	Gammaproteobacteria	Pseudomonadales
Proteobacteria	Gammaproteobacteria	Pseudomonadales
Proteobacteria	Gammaproteobacteria	Pasteurellales
Proteobacteria	Gammaproteobacteria	Pasteurellales
Proteobacteria	Gammaproteobacteria	Pasteurellales
Proteobacteria	Gammaproteobacteria	Xanthomonadales
Proteobacteria	Betaproteobacteria	Burkholderiales
Unknown	Unknown	Unknown
Proteobacteria	Gammaproteobacteria	Enterobacterales
Proteobacteria	Gammaproteobacteria	Enterobacterales
Proteobacteria	Gammaproteobacteria	Enterobacterales
Proteobacteria	Gammaproteobacteria	Pseudomonadales
Actinobacteria	Actinobacteria	Corynebacteriales
Actinobacteria	Actinobacteria	Corynebacteriales
Actinobacteria	Actinobacteria	Corynebacteriales
Actinobacteria	Actinobacteria	Corynebacteriales
Actinobacteria	Actinobacteria	Corynebacteriales
Actinobacteria	Actinobacteria	Corynebacteriales
Actinobacteria	Actinobacteria	Corynebacteriales
Actinobacteria	Actinobacteria	Corynebacteriales
Actinobacteria	Actinobacteria	Corynebacteriales
Actinobacteria	Actinobacteria	Corynebacteriales
Actinobacteria	Actinobacteria	Corynebacteriales
Actinobacteria	Actinobacteria	Corynebacteriales

Actinobacteria	Actinobacteria	Corynebacteriales
Actinobacteria	Actinobacteria	Corynebacteriales
Actinobacteria	Actinobacteria	Corynebacteriales
Actinobacteria	Actinobacteria	Corynebacteriales
Actinobacteria	Actinobacteria	Corynebacteriales
Actinobacteria	Actinobacteria	Corynebacteriales
Actinobacteria	Actinobacteria	Corynebacteriales
Unknown	Unknown	Unknown
Actinobacteria	Actinobacteria	Corynebacteriales
Actinobacteria	Actinobacteria	Corynebacteriales
Actinobacteria	Actinobacteria	Corynebacteriales
Actinobacteria	Actinobacteria	Corynebacteriales
Actinobacteria	Actinobacteria	Corynebacteriales
Actinobacteria	Actinobacteria	Corynebacteriales
Actinobacteria	Actinobacteria	Corynebacteriales
Actinobacteria	Actinobacteria	Corynebacteriales
Actinobacteria	Actinobacteria	Corynebacteriales
Actinobacteria	Actinobacteria	Corynebacteriales
Actinobacteria	Actinobacteria	Corynebacteriales
Actinobacteria	Actinobacteria	Corynebacteriales
Actinobacteria	Actinobacteria	Corynebacteriales
Actinobacteria	Actinobacteria	Corynebacteriales
Actinobacteria	Actinobacteria	Corynebacteriales
Actinobacteria	Actinobacteria	Corynebacteriales
Actinobacteria	Actinobacteria	Corynebacteriales
Actinobacteria	Actinobacteria	Corynebacteriales
Actinobacteria	Actinobacteria	Corynebacteriales
Actinobacteria	Actinobacteria	Corynebacteriales
Actinobacteria	Actinobacteria	Corynebacteriales
Actinobacteria	Actinobacteria	Corynebacteriales
Actinobacteria	Actinobacteria	Corynebacteriales
Actinobacteria	Actinobacteria	Corynebacteriales
Actinobacteria	Actinobacteria	Corynebacteriales
Actinobacteria	Actinobacteria	Corynebacteriales
Firmicutes	Bacilli	Bacillales
Unknown	Unknown	Unknown
Firmicutes	Bacilli	Bacillales
Firmicutes	Bacilli	Bacillales
Firmicutes	Bacilli	Bacillales
Firmicutes	Bacilli	Bacillales
Firmicutes	Bacilli	Bacillales
Firmicutes	Bacilli	Bacillales
Firmicutes	Bacilli	Bacillales
Firmicutes	Bacilli	Bacillales
Firmicutes	Bacilli	Lactobacillales
Firmicutes	Bacilli	Lactobacillales

Firmicutes	Bacilli	Lactobacillales
Firmicutes	Bacilli	Lactobacillales
Firmicutes	Bacilli	Lactobacillales
Firmicutes	Bacilli	Lactobacillales
Firmicutes	Bacilli	Lactobacillales
Firmicutes	Bacilli	Lactobacillales
Firmicutes	Bacilli	Lactobacillales
Firmicutes	Bacilli	Lactobacillales
Firmicutes	Bacilli	Lactobacillales
Firmicutes	Bacilli	Lactobacillales
Firmicutes	Bacilli	Lactobacillales
Firmicutes	Bacilli	Lactobacillales
Firmicutes	Bacilli	Lactobacillales
Firmicutes	Bacilli	Lactobacillales
Firmicutes	Bacilli	Lactobacillales
Firmicutes	Bacilli	Lactobacillales
Firmicutes	Bacilli	Lactobacillales
Firmicutes	Bacilli	Lactobacillales
Firmicutes	Bacilli	Lactobacillales
Firmicutes	Bacilli	Lactobacillales
Firmicutes	Bacilli	Lactobacillales
Firmicutes	Bacilli	Lactobacillales
Firmicutes	Bacilli	Lactobacillales
Firmicutes	Bacilli	Lactobacillales
Unknown	Unknown	Unknown
Unknown	Unknown	Unknown
Firmicutes	Bacilli	Lactobacillales
Firmicutes	Bacilli	Lactobacillales
Firmicutes	Bacilli	Lactobacillales
Firmicutes	Bacilli	Lactobacillales
Firmicutes	Bacilli	Lactobacillales
Firmicutes	Bacilli	Lactobacillales
Firmicutes	Bacilli	Lactobacillales
Firmicutes	Bacilli	Lactobacillales
Firmicutes	Bacilli	Lactobacillales
Firmicutes	Bacilli	Lactobacillales
Firmicutes	Bacilli	Lactobacillales
Firmicutes	Bacilli	Lactobacillales
Firmicutes	Bacilli	Lactobacillales
Firmicutes	Bacilli	Lactobacillales
Firmicutes	Bacilli	Lactobacillales
Firmicutes	Bacilli	Lactobacillales
Firmicutes	Bacilli	Lactobacillales
Firmicutes	Bacilli	Lactobacillales
Firmicutes	Bacilli	Lactobacillales
Firmicutes	Bacilli	Lactobacillales

Firmicutes	Bacilli	Lactobacillales
Firmicutes	Bacilli	Lactobacillales
Firmicutes	Bacilli	Lactobacillales
Firmicutes	Bacilli	Lactobacillales
Firmicutes	Bacilli	Lactobacillales
Proteobacteria	Gammaproteobacteria	Aeromonadales
Proteobacteria	Gammaproteobacteria	Aeromonadales
Proteobacteria	Gammaproteobacteria	Aeromonadales
Proteobacteria	Gammaproteobacteria	Aeromonadales
Proteobacteria	Gammaproteobacteria	Aeromonadales
Proteobacteria	Gammaproteobacteria	Aeromonadales
Proteobacteria	Gammaproteobacteria	Aeromonadales
Proteobacteria	Gammaproteobacteria	Aeromonadales
Proteobacteria	Gammaproteobacteria	Aeromonadales
Proteobacteria	Gammaproteobacteria	Aeromonadales
Proteobacteria	Gammaproteobacteria	Aeromonadales
Proteobacteria	Gammaproteobacteria	Aeromonadales
Proteobacteria	Gammaproteobacteria	Aeromonadales
Proteobacteria	Gammaproteobacteria	Aeromonadales
Proteobacteria	Gammaproteobacteria	Aeromonadales
Proteobacteria	Gammaproteobacteria	Aeromonadales
Proteobacteria	Gammaproteobacteria	Aeromonadales
Proteobacteria	Gammaproteobacteria	Vibrionales
Proteobacteria	Gammaproteobacteria	Vibrionales
Proteobacteria	Gammaproteobacteria	Vibrionales
Proteobacteria	Gammaproteobacteria	Vibrionales
Proteobacteria	Gammaproteobacteria	Vibrionales
Proteobacteria	Gammaproteobacteria	Vibrionales
Proteobacteria	Gammaproteobacteria	Vibrionales
Proteobacteria	Gammaproteobacteria	Vibrionales
Proteobacteria	Gammaproteobacteria	Vibrionales
Proteobacteria	Gammaproteobacteria	Vibrionales
Proteobacteria	Gammaproteobacteria	Enterobacterales
Proteobacteria	Gammaproteobacteria	Pseudomonadales
Proteobacteria	Gammaproteobacteria	Pseudomonadales
Proteobacteria	Gammaproteobacteria	Pseudomonadales
Proteobacteria	Gammaproteobacteria	Pseudomonadales
Proteobacteria	Gammaproteobacteria	Pseudomonadales
Proteobacteria	Alphaproteobacteria	Pelagibacterales
Proteobacteria	Alphaproteobacteria	Pelagibacterales
Proteobacteria	Alphaproteobacteria	Pelagibacterales
Proteobacteria	Gammaproteobacteria	Alteromonadales
Proteobacteria	Gammaproteobacteria	Alteromonadales
Proteobacteria	Gammaproteobacteria	Alteromonadales
Proteobacteria	Gammaproteobacteria	Alteromonadales
Proteobacteria	Alphaproteobacteria	Pelagibacterales

Proteobacteria	Alphaproteobacteria	Pelagibacterales
Proteobacteria	Gammaproteobacteria	Enterobacterales
Proteobacteria	Gammaproteobacteria	Enterobacterales
Proteobacteria	Gammaproteobacteria	Alteromonadales
Unknown	Unknown	Unknown
Proteobacteria	Alphaproteobacteria	Unknown
Unknown	Unknown	Unknown
Proteobacteria	Gammaproteobacteria	Xanthomonadales
Proteobacteria	Gammaproteobacteria	Aeromonadales
Proteobacteria	Gammaproteobacteria	Aeromonadales
Proteobacteria	Gammaproteobacteria	Aeromonadales
Proteobacteria	Gammaproteobacteria	Vibrionales
Proteobacteria	Gammaproteobacteria	Vibrionales
Actinobacteria	Actinobacteria	Corynebacteriales
Actinobacteria	Actinobacteria	Corynebacteriales
Actinobacteria	Actinobacteria	Corynebacteriales
Actinobacteria	Actinobacteria	Corynebacteriales
Actinobacteria	Actinobacteria	Corynebacteriales
Actinobacteria	Actinobacteria	Corynebacteriales
Actinobacteria	Actinobacteria	Corynebacteriales
Actinobacteria	Actinobacteria	Corynebacteriales
Actinobacteria	Actinobacteria	Corynebacteriales
Actinobacteria	Actinobacteria	Corynebacteriales
Actinobacteria	Actinobacteria	Corynebacteriales
Actinobacteria	Actinobacteria	Corynebacteriales
Actinobacteria	Actinobacteria	Corynebacteriales
Actinobacteria	Actinobacteria	Corynebacteriales
Actinobacteria	Actinobacteria	Corynebacteriales
Actinobacteria	Actinobacteria	Corynebacteriales
Actinobacteria	Actinobacteria	Corynebacteriales
Actinobacteria	Actinobacteria	Corynebacteriales
Actinobacteria	Actinobacteria	Corynebacteriales
Actinobacteria	Actinobacteria	Corynebacteriales
Actinobacteria	Actinobacteria	Corynebacteriales
Actinobacteria	Actinobacteria	Corynebacteriales
Actinobacteria	Actinobacteria	Corynebacteriales
Actinobacteria	Actinobacteria	Corynebacteriales
Unknown	Unknown	Unknown
Unknown	Unknown	Unknown
Firmicutes	Bacilli	Bacillales
Firmicutes	Bacilli	Bacillales
Firmicutes	Bacilli	Bacillales
Firmicutes	Bacilli	Bacillales
Firmicutes	Bacilli	Bacillales
Firmicutes	Bacilli	Bacillales
Firmicutes	Bacilli	Bacillales

Firmicutes	Bacilli	Bacillales
Firmicutes	Bacilli	Lactobacillales
Firmicutes	Bacilli	Lactobacillales
Firmicutes	Bacilli	Lactobacillales
Firmicutes	Bacilli	Lactobacillales
Firmicutes	Bacilli	Lactobacillales
Firmicutes	Bacilli	Lactobacillales
Firmicutes	Bacilli	Lactobacillales
Firmicutes	Bacilli	Lactobacillales
Firmicutes	Bacilli	Lactobacillales
Firmicutes	Bacilli	Lactobacillales
Firmicutes	Bacilli	Lactobacillales
Firmicutes	Bacilli	Lactobacillales
Firmicutes	Bacilli	Lactobacillales
Firmicutes	Bacilli	Lactobacillales
Firmicutes	Bacilli	Lactobacillales
Actinobacteria	Actinobacteria	Streptomycetales
Actinobacteria	Actinobacteria	Streptomycetales
Actinobacteria	Actinobacteria	Streptomycetales
Actinobacteria	Actinobacteria	Streptomycetales
Actinobacteria	Actinobacteria	Streptomycetales
Actinobacteria	Actinobacteria	Streptomycetales
Actinobacteria	Actinobacteria	Streptomycetales
Actinobacteria	Actinobacteria	Streptomycetales
Actinobacteria	Actinobacteria	Streptomycetales
Actinobacteria	Actinobacteria	Streptomycetales
Actinobacteria	Actinobacteria	Streptomycetales
Actinobacteria	Actinobacteria	Streptomycetales
Actinobacteria	Actinobacteria	Streptomycetales
Actinobacteria	Actinobacteria	Streptomycetales
Actinobacteria	Actinobacteria	Streptomycetales
Actinobacteria	Actinobacteria	Streptomycetales
Actinobacteria	Actinobacteria	Streptomycetales
Actinobacteria	Actinobacteria	Streptomycetales
Actinobacteria	Actinobacteria	Streptomycetales
Actinobacteria	Actinobacteria	Streptomycetales
Actinobacteria	Actinobacteria	Streptomycetales
Actinobacteria	Actinobacteria	Streptomycetales
Actinobacteria	Actinobacteria	Streptomycetales
Actinobacteria	Actinobacteria	Streptomycetales
Actinobacteria	Actinobacteria	Streptomycetales
Actinobacteria	Actinobacteria	Streptomycetales
Actinobacteria	Actinobacteria	Streptomycetales
Actinobacteria	Actinobacteria	Streptomycetales
Actinobacteria	Actinobacteria	Streptomycetales
Actinobacteria	Actinobacteria	Streptomycetales

Actinobacteria	Actinobacteria	Streptomycetales
Actinobacteria	Actinobacteria	Streptomycetales
Actinobacteria	Actinobacteria	Streptomycetales
Actinobacteria	Actinobacteria	Streptomycetales
Actinobacteria	Actinobacteria	Streptomycetales
Actinobacteria	Actinobacteria	Streptomycetales
Actinobacteria	Actinobacteria	Streptomycetales
Actinobacteria	Actinobacteria	Streptomycetales
Actinobacteria	Actinobacteria	Streptomycetales
Actinobacteria	Actinobacteria	Streptomycetales
Actinobacteria	Actinobacteria	Streptomycetales
Actinobacteria	Actinobacteria	Streptomycetales
Actinobacteria	Actinobacteria	Streptomycetales
Actinobacteria	Actinobacteria	Streptomycetales
Actinobacteria	Actinobacteria	Streptomycetales
Actinobacteria	Actinobacteria	Streptomycetales
Actinobacteria	Actinobacteria	Streptomycetales
Actinobacteria	Actinobacteria	Streptomycetales
Actinobacteria	Actinobacteria	Streptomycetales
Actinobacteria	Actinobacteria	Streptomycetales
Actinobacteria	Actinobacteria	Streptomycetales
Actinobacteria	Actinobacteria	Streptomycetales
Actinobacteria	Actinobacteria	Streptomycetales
Actinobacteria	Actinobacteria	Streptomycetales
Actinobacteria	Actinobacteria	Streptomycetales
Firmicutes	Bacilli	Lactobacillales
Firmicutes	Bacilli	Lactobacillales
Firmicutes	Bacilli	Lactobacillales
Firmicutes	Bacilli	Lactobacillales
Firmicutes	Bacilli	Lactobacillales
Firmicutes	Bacilli	Lactobacillales
Unknown	Unknown	Unknown
Firmicutes	Bacilli	Lactobacillales
Firmicutes	Bacilli	Lactobacillales
Firmicutes	Bacilli	Lactobacillales
Firmicutes	Bacilli	Lactobacillales
Firmicutes	Bacilli	Lactobacillales
Firmicutes	Bacilli	Lactobacillales
Firmicutes	Bacilli	Lactobacillales
Firmicutes	Bacilli	Lactobacillales
Firmicutes	Bacilli	Lactobacillales
Unknown	Unknown	Unknown
Firmicutes	Bacilli	Lactobacillales
Unknown	Unknown	Unknown
Firmicutes	Bacilli	Lactobacillales
Firmicutes	Bacilli	Lactobacillales

Firmicutes	Bacilli	Lactobacillales
Unknown	Unknown	Unknown
Firmicutes	Bacilli	Lactobacillales
Firmicutes	Bacilli	Lactobacillales
Firmicutes	Bacilli	Lactobacillales
Firmicutes	Bacilli	Lactobacillales
Firmicutes	Bacilli	Lactobacillales
Firmicutes	Bacilli	Lactobacillales
Firmicutes	Bacilli	Lactobacillales
Firmicutes	Bacilli	Lactobacillales
Firmicutes	Bacilli	Lactobacillales
Firmicutes	Bacilli	Lactobacillales
Firmicutes	Bacilli	Lactobacillales
Firmicutes	Bacilli	Lactobacillales
Firmicutes	Bacilli	Lactobacillales
Firmicutes	Bacilli	Lactobacillales
Firmicutes	Bacilli	Lactobacillales
Firmicutes	Bacilli	Lactobacillales
Firmicutes	Bacilli	Lactobacillales
Firmicutes	Bacilli	Lactobacillales
Firmicutes	Bacilli	Lactobacillales
Firmicutes	Bacilli	Lactobacillales
Firmicutes	Bacilli	Lactobacillales
Firmicutes	Bacilli	Lactobacillales
Firmicutes	Bacilli	Lactobacillales
Firmicutes	Bacilli	Lactobacillales
Firmicutes	Bacilli	Lactobacillales
Firmicutes	Bacilli	Lactobacillales
Firmicutes	Bacilli	Lactobacillales
Firmicutes	Bacilli	Lactobacillales
Firmicutes	Bacilli	Bacillales
Firmicutes	Bacilli	Bacillales
Firmicutes	Bacilli	Bacillales
Firmicutes	Bacilli	Bacillales
Firmicutes	Bacilli	Bacillales
Firmicutes	Bacilli	Bacillales
Firmicutes	Bacilli	Bacillales
Firmicutes	Bacilli	Bacillales
Firmicutes	Bacilli	Bacillales
Firmicutes	Bacilli	Bacillales
Unknown	Unknown	Unknown
Firmicutes	Bacilli	Bacillales
Firmicutes	Bacilli	Bacillales
Unknown	Unknown	Unknown
Firmicutes	Bacilli	Bacillales
Firmicutes	Bacilli	Bacillales

Firmicutes	Bacilli	Bacillales
Firmicutes	Bacilli	Bacillales
Firmicutes	Bacilli	Bacillales
Firmicutes	Bacilli	Bacillales
Firmicutes	Bacilli	Bacillales
Firmicutes	Bacilli	Bacillales
Unknown	Unknown	Unknown
Firmicutes	Bacilli	Bacillales
Firmicutes	Bacilli	Bacillales
Firmicutes	Bacilli	Bacillales
Firmicutes	Bacilli	Bacillales
Firmicutes	Bacilli	Bacillales
Unknown	Unknown	Unknown
Firmicutes	Bacilli	Bacillales
Firmicutes	Bacilli	Bacillales
Firmicutes	Bacilli	Bacillales
Firmicutes	Bacilli	Bacillales
Firmicutes	Bacilli	Bacillales
Firmicutes	Bacilli	Lactobacillales
Firmicutes	Bacilli	Lactobacillales
Firmicutes	Bacilli	Lactobacillales
Firmicutes	Bacilli	Lactobacillales
Firmicutes	Bacilli	Lactobacillales
Firmicutes	Bacilli	Lactobacillales
Firmicutes	Bacilli	Bacillales
Firmicutes	Bacilli	Bacillales
Firmicutes	Bacilli	Lactobacillales
Firmicutes	Bacilli	Lactobacillales
Firmicutes	Bacilli	Lactobacillales
Firmicutes	Bacilli	Lactobacillales
Firmicutes	Bacilli	Bacillales
Firmicutes	Bacilli	Lactobacillales
Firmicutes	Bacilli	Lactobacillales
Firmicutes	Bacilli	Bacillales
Firmicutes	Bacilli	Bacillales
Firmicutes	Bacilli	Bacillales
Firmicutes	Bacilli	Bacillales
Firmicutes	Bacilli	Bacillales
Firmicutes	Bacilli	Bacillales
Firmicutes	Bacilli	Bacillales
Firmicutes	Bacilli	Bacillales
Firmicutes	Bacilli	Bacillales
Firmicutes	Bacilli	Bacillales
Proteobacteria	Betaproteobacteria	Burkholderiales
Proteobacteria	Betaproteobacteria	Burkholderiales
Proteobacteria	Betaproteobacteria	Burkholderiales

Proteobacteria	Betaproteobacteria	Burkholderiales
Proteobacteria	Betaproteobacteria	Burkholderiales
Proteobacteria	Gammaproteobacteria	Pseudomonadales
Proteobacteria	Gammaproteobacteria	Pseudomonadales
Proteobacteria	Gammaproteobacteria	Pseudomonadales
Proteobacteria	Gammaproteobacteria	Pseudomonadales
Proteobacteria	Gammaproteobacteria	Pseudomonadales
Proteobacteria	Gammaproteobacteria	Pseudomonadales
Proteobacteria	Gammaproteobacteria	Pseudomonadales
Unknown	Unknown	Unknown
Proteobacteria	Gammaproteobacteria	Enterobacterales
Proteobacteria	Betaproteobacteria	Burkholderiales
Firmicutes	Bacilli	Lactobacillales
Firmicutes	Bacilli	Lactobacillales
Firmicutes	Bacilli	Lactobacillales
Firmicutes	Bacilli	Lactobacillales
Firmicutes	Bacilli	Lactobacillales
Firmicutes	Bacilli	Lactobacillales
Firmicutes	Bacilli	Lactobacillales
Firmicutes	Bacilli	Lactobacillales
Firmicutes	Bacilli	Lactobacillales
Firmicutes	Bacilli	Lactobacillales
Firmicutes	Bacilli	Lactobacillales
Firmicutes	Bacilli	Lactobacillales
Firmicutes	Bacilli	Lactobacillales
Firmicutes	Bacilli	Lactobacillales
Firmicutes	Bacilli	Lactobacillales
Firmicutes	Bacilli	Lactobacillales
Firmicutes	Bacilli	Lactobacillales
Firmicutes	Bacilli	Lactobacillales
Actinobacteria	Actinobacteria	Micrococcales
Actinobacteria	Actinobacteria	Micrococcales
Actinobacteria	Actinobacteria	Micrococcales
Actinobacteria	Actinobacteria	Micrococcales
Actinobacteria	Actinobacteria	Micrococcales
Actinobacteria	Actinobacteria	Micrococcales
Actinobacteria	Actinobacteria	Micrococcales
Actinobacteria	Actinobacteria	Micrococcales
Actinobacteria	Actinobacteria	Micrococcales
Actinobacteria	Actinobacteria	Micrococcales
Actinobacteria	Actinobacteria	Micrococcales
Actinobacteria	Actinobacteria	Micrococcales
Actinobacteria	Actinobacteria	Micrococcales
Actinobacteria	Actinobacteria	Micrococcales
Actinobacteria	Actinobacteria	Micrococcales

Actinobacteria	Actinobacteria	Micrococcales
Actinobacteria	Actinobacteria	Micrococcales
Actinobacteria	Actinobacteria	Micrococcales
Actinobacteria	Actinobacteria	Micrococcales
Firmicutes	Bacilli	Bacillales
Firmicutes	Bacilli	Bacillales
Firmicutes	Bacilli	Bacillales
Firmicutes	Bacilli	Bacillales
Unknown	Unknown	Unknown
Firmicutes	Bacilli	Bacillales
Firmicutes	Bacilli	Bacillales
Firmicutes	Bacilli	Bacillales
Unknown	Unknown	Unknown
Firmicutes	Bacilli	Bacillales
Firmicutes	Bacilli	Bacillales
Firmicutes	Bacilli	Bacillales
Firmicutes	Bacilli	Bacillales
Firmicutes	Bacilli	Bacillales
Firmicutes	Bacilli	Bacillales
Firmicutes	Bacilli	Bacillales
Firmicutes	Bacilli	Bacillales
Proteobacteria	Gammaproteobacteria	Pseudomonadales
Proteobacteria	Gammaproteobacteria	Pseudomonadales
Proteobacteria	Gammaproteobacteria	Pseudomonadales
Proteobacteria	Gammaproteobacteria	Pseudomonadales
Proteobacteria	Gammaproteobacteria	Pseudomonadales
Proteobacteria	Gammaproteobacteria	Pseudomonadales
Unknown	Unknown	Unknown
Proteobacteria	Gammaproteobacteria	Pseudomonadales
Proteobacteria	Gammaproteobacteria	Pseudomonadales
Proteobacteria	Betaproteobacteria	Burkholderiales
Proteobacteria	Betaproteobacteria	Burkholderiales
Proteobacteria	Betaproteobacteria	Burkholderiales
Proteobacteria	Betaproteobacteria	Burkholderiales
Proteobacteria	Betaproteobacteria	Burkholderiales
Unknown	Unknown	Unknown
Unknown	Unknown	Unknown
Proteobacteria	Alphaproteobacteria	Caulobacterales
Proteobacteria	Alphaproteobacteria	Caulobacterales
Proteobacteria	Alphaproteobacteria	Caulobacterales
Proteobacteria	Alphaproteobacteria	Caulobacterales
Proteobacteria	Alphaproteobacteria	Caulobacterales
Proteobacteria	Alphaproteobacteria	Caulobacterales
Proteobacteria	Alphaproteobacteria	Caulobacterales
Proteobacteria	Alphaproteobacteria	Caulobacterales
Proteobacteria	Alphaproteobacteria	Caulobacterales

Proteobacteria	Alphaproteobacteria	Caulobacterales
Proteobacteria	Gammaproteobacteria	Enterobacterales
Proteobacteria	Gammaproteobacteria	Enterobacterales
Proteobacteria	Gammaproteobacteria	Enterobacterales
Proteobacteria	Gammaproteobacteria	Enterobacterales
Proteobacteria	Gammaproteobacteria	Aeromonadales
Proteobacteria	Gammaproteobacteria	Aeromonadales
Proteobacteria	Gammaproteobacteria	Aeromonadales
Proteobacteria	Gammaproteobacteria	Aeromonadales
Proteobacteria	Gammaproteobacteria	Enterobacterales
Proteobacteria	Gammaproteobacteria	Enterobacterales
Proteobacteria	Gammaproteobacteria	Enterobacterales
Proteobacteria	Gammaproteobacteria	Enterobacterales
Proteobacteria	Gammaproteobacteria	Enterobacterales
Proteobacteria	Gammaproteobacteria	Aeromonadales
Proteobacteria	Gammaproteobacteria	Aeromonadales
Proteobacteria	Gammaproteobacteria	Vibrionales
Proteobacteria	Gammaproteobacteria	Vibrionales
Proteobacteria	Gammaproteobacteria	Vibrionales
Proteobacteria	Gammaproteobacteria	Enterobacterales
Proteobacteria	Gammaproteobacteria	Enterobacterales
Proteobacteria	Gammaproteobacteria	Pasteurellales
Proteobacteria	Gammaproteobacteria	Enterobacterales
Proteobacteria	Gammaproteobacteria	Enterobacterales
Proteobacteria	Gammaproteobacteria	Enterobacterales
Proteobacteria	Gammaproteobacteria	Enterobacterales
Proteobacteria	Gammaproteobacteria	Enterobacterales
Proteobacteria	Gammaproteobacteria	Enterobacterales
Unknown	Unknown	Unknown
Proteobacteria	Gammaproteobacteria	Enterobacterales
Proteobacteria	Gammaproteobacteria	Enterobacterales
Proteobacteria	Gammaproteobacteria	Enterobacterales
Proteobacteria	Gammaproteobacteria	Enterobacterales
Proteobacteria	Gammaproteobacteria	Pasteurellales
Proteobacteria	Gammaproteobacteria	Pasteurellales
Proteobacteria	Gammaproteobacteria	Pasteurellales
Proteobacteria	Gammaproteobacteria	Pasteurellales
Proteobacteria	Gammaproteobacteria	Pasteurellales
Proteobacteria	Gammaproteobacteria	Pasteurellales
Firmicutes	Bacilli	Bacillales
Firmicutes	Bacilli	Bacillales
Firmicutes	Bacilli	Bacillales
Cyanobacteria	Cyanophyceae	Nostocales
Cyanobacteria	Cyanophyceae	Nostocales
Cyanobacteria	Cyanophyceae	Nostocales
Proteobacteria	Acidithiobacillia	Acidithiobacillales

Unknown	Unknown	Unknown
Cyanobacteria	Cyanophyceae	Synechococcales
Proteobacteria	Gammaproteobacteria	Enterobacterales
Proteobacteria	Gammaproteobacteria	Enterobacterales
Proteobacteria	Gammaproteobacteria	Enterobacterales
Proteobacteria	Gammaproteobacteria	Enterobacterales
Unknown	Unknown	Unknown
Unknown	Unknown	Unknown
Proteobacteria	Alphaproteobacteria	Rhizobiales
Proteobacteria	Gammaproteobacteria	Pseudomonadales
Unknown	Unknown	Unknown
Proteobacteria	Gammaproteobacteria	Xanthomonadales
Proteobacteria	Betaproteobacteria	Burkholderiales
Cyanobacteria	Cyanophyceae	Synechococcales
Cyanobacteria	Cyanophyceae	Synechococcales
Unknown	Unknown	Unknown
Proteobacteria	Alphaproteobacteria	Rhodobacterales
Proteobacteria	Alphaproteobacteria	Rhodobacterales
Cyanobacteria	Cyanophyceae	Synechococcales
Cyanobacteria	Cyanophyceae	Synechococcales
Cyanobacteria	Cyanophyceae	Synechococcales
Cyanobacteria	Cyanophyceae	Synechococcales
Cyanobacteria	Cyanophyceae	Synechococcales
Cyanobacteria	Cyanophyceae	Synechococcales
Cyanobacteria	Cyanophyceae	Synechococcales
Proteobacteria	Betaproteobacteria	Burkholderiales
Proteobacteria	Betaproteobacteria	Burkholderiales
Proteobacteria	Betaproteobacteria	Burkholderiales
Proteobacteria	Gammaproteobacteria	Pseudomonadales
Proteobacteria	Gammaproteobacteria	Pseudomonadales
Proteobacteria	Gammaproteobacteria	Pseudomonadales
Proteobacteria	Gammaproteobacteria	Pseudomonadales
Proteobacteria	Gammaproteobacteria	Pseudomonadales
Proteobacteria	Gammaproteobacteria	Pseudomonadales
Proteobacteria	Gammaproteobacteria	Pseudomonadales
Cyanobacteria	Cyanophyceae	Synechococcales
Cyanobacteria	Cyanophyceae	Synechococcales
Cyanobacteria	Cyanophyceae	Synechococcales
Cyanobacteria	Cyanophyceae	Synechococcales
Cyanobacteria	Cyanophyceae	Synechococcales
Cyanobacteria	Cyanophyceae	Synechococcales
Cyanobacteria	Cyanophyceae	Synechococcales
Firmicutes	Bacilli	Bacillales
Firmicutes	Bacilli	Bacillales
Firmicutes	Bacilli	Bacillales
Firmicutes	Bacilli	Bacillales

Firmicutes	Bacilli	Bacillales
Unknown	Unknown	Unknown
Firmicutes	Bacilli	Bacillales
Firmicutes	Bacilli	Bacillales
Firmicutes	Bacilli	Bacillales
Firmicutes	Bacilli	Bacillales
Firmicutes	Bacilli	Bacillales
Firmicutes	Bacilli	Bacillales
Firmicutes	Bacilli	Bacillales
Firmicutes	Bacilli	Bacillales
Firmicutes	Bacilli	Bacillales
Firmicutes	Bacilli	Bacillales
Actinobacteria	Actinobacteria	Streptomycetales
Actinobacteria	Actinobacteria	Streptomycetales
Actinobacteria	Actinobacteria	Streptomycetales
Actinobacteria	Actinobacteria	Streptomycetales
Actinobacteria	Actinobacteria	Streptomycetales
Actinobacteria	Actinobacteria	Streptomycetales
Actinobacteria	Actinobacteria	Streptomycetales
Actinobacteria	Actinobacteria	Streptomycetales
Actinobacteria	Actinobacteria	Streptomycetales
Actinobacteria	Actinobacteria	Streptomycetales
Actinobacteria	Actinobacteria	Streptomycetales
Actinobacteria	Actinobacteria	Streptomycetales
Actinobacteria	Actinobacteria	Streptomycetales
Actinobacteria	Actinobacteria	Streptomycetales
Actinobacteria	Actinobacteria	Streptomycetales
Actinobacteria	Actinobacteria	Streptomycetales
Actinobacteria	Actinobacteria	Streptomycetales
Firmicutes	Bacilli	Bacillales
Firmicutes	Bacilli	Bacillales
Firmicutes	Bacilli	Bacillales
Firmicutes	Bacilli	Bacillales
Firmicutes	Bacilli	Bacillales
Firmicutes	Bacilli	Bacillales
Firmicutes	Bacilli	Bacillales
Firmicutes	Bacilli	Bacillales
Firmicutes	Bacilli	Bacillales
Firmicutes	Bacilli	Bacillales
Firmicutes	Bacilli	Bacillales
Firmicutes	Bacilli	Bacillales
Firmicutes	Bacilli	Bacillales
Firmicutes	Bacilli	Bacillales
Firmicutes	Bacilli	Bacillales
Firmicutes	Bacilli	Bacillales
Firmicutes	Bacilli	Bacillales

Firmicutes	Bacilli	Bacillales
Unknown	Unknown	Unknown
Firmicutes	Bacilli	Bacillales
Firmicutes	Bacilli	Bacillales
Firmicutes	Bacilli	Bacillales
Firmicutes	Bacilli	Bacillales
Firmicutes	Bacilli	Bacillales
Firmicutes	Clostridia	Clostridiales
Firmicutes	Clostridia	Clostridiales
Firmicutes	Clostridia	Clostridiales
Firmicutes	Clostridia	Clostridiales
Firmicutes	Clostridia	Clostridiales
Firmicutes	Clostridia	Clostridiales
Firmicutes	Clostridia	Clostridiales
Firmicutes	Clostridia	Clostridiales
Firmicutes	Clostridia	Clostridiales
Firmicutes	Clostridia	Clostridiales
Firmicutes	Clostridia	Clostridiales
Firmicutes	Clostridia	Clostridiales
Firmicutes	Clostridia	Clostridiales
Firmicutes	Clostridia	Clostridiales
Unknown	Unknown	Unknown
Unknown	Unknown	Unknown
Unknown	Unknown	Unknown
Unknown	Unknown	Unknown
Unknown	Unknown	Unknown
Unknown	Unknown	Unknown
Unknown	Unknown	Unknown
Unknown	Unknown	Unknown
Proteobacteria	Gammaproteobacteria	Enterobacterales
Proteobacteria	Gammaproteobacteria	Enterobacterales
Proteobacteria	Gammaproteobacteria	Enterobacterales
Proteobacteria	Gammaproteobacteria	Enterobacterales
Proteobacteria	Gammaproteobacteria	Enterobacterales
Proteobacteria	Gammaproteobacteria	Enterobacterales
Proteobacteria	Gammaproteobacteria	Enterobacterales
Proteobacteria	Gammaproteobacteria	Xanthomonadales
Proteobacteria	Gammaproteobacteria	Aeromonadales
Proteobacteria	Gammaproteobacteria	Aeromonadales
Proteobacteria	Gammaproteobacteria	Aeromonadales
Proteobacteria	Gammaproteobacteria	Aeromonadales
Proteobacteria	Gammaproteobacteria	Aeromonadales
Proteobacteria	Gammaproteobacteria	Aeromonadales
Proteobacteria	Gammaproteobacteria	Aeromonadales
Proteobacteria	Gammaproteobacteria	Aeromonadales
Proteobacteria	Gammaproteobacteria	Enterobacterales

Proteobacteria	Gammaproteobacteria	Enterobacterales
Proteobacteria	Gammaproteobacteria	Enterobacterales
Proteobacteria	Gammaproteobacteria	Enterobacterales
Proteobacteria	Gammaproteobacteria	Enterobacterales
Proteobacteria	Gammaproteobacteria	Enterobacterales
Proteobacteria	Gammaproteobacteria	Enterobacterales
Proteobacteria	Gammaproteobacteria	Enterobacterales
Proteobacteria	Gammaproteobacteria	Pseudomonadales
Proteobacteria	Gammaproteobacteria	Pseudomonadales
Proteobacteria	Gammaproteobacteria	Pseudomonadales
Proteobacteria	Gammaproteobacteria	Pseudomonadales
Proteobacteria	Gammaproteobacteria	Pseudomonadales
Proteobacteria	Gammaproteobacteria	Pseudomonadales
Proteobacteria	Gammaproteobacteria	Pseudomonadales
Proteobacteria	Gammaproteobacteria	Pseudomonadales
Proteobacteria	Gammaproteobacteria	Pseudomonadales
Proteobacteria	Gammaproteobacteria	Pseudomonadales
Proteobacteria	Gammaproteobacteria	Pseudomonadales
Proteobacteria	Gammaproteobacteria	Pseudomonadales
Proteobacteria	Gammaproteobacteria	Pseudomonadales
Proteobacteria	Gammaproteobacteria	Pseudomonadales
Proteobacteria	Gammaproteobacteria	Pseudomonadales
Proteobacteria	Gammaproteobacteria	Pseudomonadales
Proteobacteria	Gammaproteobacteria	Pseudomonadales
Unknown	Unknown	Unknown
Proteobacteria	Gammaproteobacteria	Pseudomonadales
Proteobacteria	Gammaproteobacteria	Pseudomonadales
Proteobacteria	Alphaproteobacteria	Rhizobiales
Proteobacteria	Alphaproteobacteria	Rhizobiales
Proteobacteria	Alphaproteobacteria	Rhizobiales
Proteobacteria	Alphaproteobacteria	Rhizobiales
Cyanobacteria	Cyanophyceae	Synechococcales
Cyanobacteria	Cyanophyceae	Synechococcales
Proteobacteria	Alphaproteobacteria	Rhodobacterales
Proteobacteria	Alphaproteobacteria	Rhodobacterales
Proteobacteria	Alphaproteobacteria	Rhizobiales
Proteobacteria	Alphaproteobacteria	Unknown
Unknown	Unknown	Unknown
Proteobacteria	Alphaproteobacteria	Rhizobiales
Firmicutes	Bacilli	Bacillales
Firmicutes	Bacilli	Bacillales
Firmicutes	Bacilli	Bacillales
Unknown	Unknown	Unknown
Firmicutes	Bacilli	Bacillales
Firmicutes	Bacilli	Bacillales

Firmicutes	Bacilli	Bacillales
Unknown	Unknown	Unknown
Firmicutes	Bacilli	Bacillales
Firmicutes	Bacilli	Bacillales
Actinobacteria	Actinobacteria	Streptomycetales
Actinobacteria	Actinobacteria	Streptomycetales
Actinobacteria	Actinobacteria	Streptomycetales
Actinobacteria	Actinobacteria	Streptomycetales
Actinobacteria	Actinobacteria	Streptomycetales
Actinobacteria	Actinobacteria	Streptomycetales
Actinobacteria	Actinobacteria	Streptomycetales
Actinobacteria	Actinobacteria	Streptomycetales
Actinobacteria	Actinobacteria	Streptomycetales
Actinobacteria	Actinobacteria	Streptomycetales
Actinobacteria	Actinobacteria	Streptomycetales
Actinobacteria	Actinobacteria	Streptomycetales
Actinobacteria	Actinobacteria	Streptomycetales
Actinobacteria	Actinobacteria	Streptomycetales
Actinobacteria	Actinobacteria	Streptomycetales
Actinobacteria	Actinobacteria	Streptomycetales
Actinobacteria	Actinobacteria	Streptomycetales
Actinobacteria	Actinobacteria	Streptomycetales
Actinobacteria	Actinobacteria	Streptomycetales
Actinobacteria	Actinobacteria	Streptomycetales
Actinobacteria	Actinobacteria	Micrococcales
Actinobacteria	Actinobacteria	Micrococcales
Actinobacteria	Actinobacteria	Micrococcales
Actinobacteria	Actinobacteria	Micrococcales
Actinobacteria	Actinobacteria	Micrococcales
Actinobacteria	Actinobacteria	Micrococcales
Actinobacteria	Actinobacteria	Micrococcales
Actinobacteria	Actinobacteria	Micrococcales
Actinobacteria	Actinobacteria	Micrococcales
Actinobacteria	Actinobacteria	Micrococcales
Actinobacteria	Actinobacteria	Micrococcales
Actinobacteria	Actinobacteria	Micrococcales
Actinobacteria	Actinobacteria	Micrococcales
Actinobacteria	Actinobacteria	Micrococcales
Actinobacteria	Actinobacteria	Micrococcales
Actinobacteria	Actinobacteria	Micrococcales
Actinobacteria	Actinobacteria	Micrococcales
Proteobacteria	Gammaproteobacteria	Pseudomonadales
Proteobacteria	Gammaproteobacteria	Pseudomonadales
Proteobacteria	Gammaproteobacteria	Pseudomonadales
Proteobacteria	Gammaproteobacteria	Pseudomonadales
Proteobacteria	Gammaproteobacteria	Pseudomonadales

Proteobacteria	Gammaproteobacteria	Pseudomonadales
Proteobacteria	Gammaproteobacteria	Pseudomonadales
Proteobacteria	Gammaproteobacteria	Pseudomonadales
Unknown	Unknown	Unknown
Unknown	Unknown	Unknown
Proteobacteria	Betaproteobacteria	Burkholderiales
Proteobacteria	Betaproteobacteria	Burkholderiales
Unknown	Unknown	Unknown
Proteobacteria	Gammaproteobacteria	Alteromonadales
Proteobacteria	Betaproteobacteria	Burkholderiales
Proteobacteria	Betaproteobacteria	Burkholderiales
Actinobacteria	Actinobacteria	Corynebacteriales
Actinobacteria	Actinobacteria	Corynebacteriales
Actinobacteria	Actinobacteria	Corynebacteriales
Actinobacteria	Actinobacteria	Corynebacteriales
Actinobacteria	Actinobacteria	Corynebacteriales
Actinobacteria	Actinobacteria	Corynebacteriales
Actinobacteria	Actinobacteria	Corynebacteriales
Actinobacteria	Actinobacteria	Corynebacteriales
Actinobacteria	Actinobacteria	Corynebacteriales
Actinobacteria	Actinobacteria	Corynebacteriales
Actinobacteria	Actinobacteria	Corynebacteriales
Actinobacteria	Actinobacteria	Corynebacteriales
Actinobacteria	Actinobacteria	Corynebacteriales
Actinobacteria	Actinobacteria	Corynebacteriales
Actinobacteria	Actinobacteria	Corynebacteriales
Actinobacteria	Actinobacteria	Corynebacteriales
Actinobacteria	Actinobacteria	Corynebacteriales
Actinobacteria	Actinobacteria	Corynebacteriales
Actinobacteria	Actinobacteria	Corynebacteriales
Actinobacteria	Actinobacteria	Corynebacteriales
Actinobacteria	Actinobacteria	Corynebacteriales
Actinobacteria	Actinobacteria	Corynebacteriales
Actinobacteria	Actinobacteria	Corynebacteriales
Actinobacteria	Actinobacteria	Corynebacteriales
Actinobacteria	Actinobacteria	Corynebacteriales
Actinobacteria	Actinobacteria	Corynebacteriales
Actinobacteria	Actinobacteria	Corynebacteriales
Actinobacteria	Actinobacteria	Corynebacteriales
Actinobacteria	Actinobacteria	Corynebacteriales
Actinobacteria	Actinobacteria	Corynebacteriales
Actinobacteria	Actinobacteria	Corynebacteriales
Actinobacteria	Actinobacteria	Corynebacteriales
Actinobacteria	Actinobacteria	Corynebacteriales
Actinobacteria	Actinobacteria	Corynebacteriales
Actinobacteria	Actinobacteria	Corynebacteriales

Actinobacteria	Actinobacteria	Corynebacteriales
Actinobacteria	Actinobacteria	Corynebacteriales
Actinobacteria	Actinobacteria	Corynebacteriales
Actinobacteria	Actinobacteria	Corynebacteriales
Actinobacteria	Actinobacteria	Corynebacteriales
Actinobacteria	Actinobacteria	Corynebacteriales
Proteobacteria	Gammaproteobacteria	Vibrionales
Proteobacteria	Gammaproteobacteria	Vibrionales
Proteobacteria	Gammaproteobacteria	Vibrionales
Proteobacteria	Gammaproteobacteria	Vibrionales
Proteobacteria	Gammaproteobacteria	Vibrionales
Proteobacteria	Gammaproteobacteria	Vibrionales
Proteobacteria	Gammaproteobacteria	Vibrionales
Proteobacteria	Gammaproteobacteria	Vibrionales
Firmicutes	Bacilli	Lactobacillales
Firmicutes	Bacilli	Lactobacillales
Firmicutes	Bacilli	Lactobacillales
Firmicutes	Bacilli	Lactobacillales
Firmicutes	Bacilli	Lactobacillales
Firmicutes	Bacilli	Lactobacillales
Firmicutes	Bacilli	Lactobacillales
Actinobacteria	Actinobacteria	Micrococcales
Actinobacteria	Actinobacteria	Micrococcales
Actinobacteria	Actinobacteria	Micrococcales
Actinobacteria	Actinobacteria	Micrococcales
Actinobacteria	Actinobacteria	Micrococcales
Actinobacteria	Actinobacteria	Micrococcales
Firmicutes	Bacilli	Lactobacillales
Firmicutes	Bacilli	Lactobacillales
Firmicutes	Bacilli	Lactobacillales
Firmicutes	Bacilli	Lactobacillales
Firmicutes	Bacilli	Lactobacillales
Firmicutes	Bacilli	Lactobacillales
Firmicutes	Bacilli	Lactobacillales
Firmicutes	Bacilli	Lactobacillales
Firmicutes	Bacilli	Lactobacillales
Firmicutes	Bacilli	Lactobacillales
Firmicutes	Bacilli	Lactobacillales
Firmicutes	Bacilli	Lactobacillales
Firmicutes	Bacilli	Lactobacillales
Firmicutes	Bacilli	Lactobacillales
Firmicutes	Bacilli	Lactobacillales
Firmicutes	Bacilli	Lactobacillales
Firmicutes	Bacilli	Lactobacillales
Firmicutes	Bacilli	Lactobacillales
Unknown	Unknown	Unknown

Firmicutes	Bacilli	Lactobacillales
Firmicutes	Bacilli	Lactobacillales
Firmicutes	Bacilli	Lactobacillales
Firmicutes	Bacilli	Lactobacillales
Firmicutes	Bacilli	Lactobacillales
Firmicutes	Bacilli	Lactobacillales
Firmicutes	Bacilli	Bacillales
Firmicutes	Bacilli	Bacillales
Firmicutes	Bacilli	Bacillales
Unknown	Unknown	Unknown
Firmicutes	Bacilli	Bacillales
Firmicutes	Bacilli	Bacillales
Firmicutes	Bacilli	Bacillales
Firmicutes	Bacilli	Bacillales
Firmicutes	Bacilli	Bacillales
Firmicutes	Bacilli	Bacillales
Firmicutes	Bacilli	Bacillales
Firmicutes	Bacilli	Bacillales
Firmicutes	Bacilli	Bacillales
Firmicutes	Bacilli	Bacillales
Actinobacteria	Actinobacteria	Corynebacteriales
Actinobacteria	Actinobacteria	Corynebacteriales
Actinobacteria	Actinobacteria	Corynebacteriales
Actinobacteria	Actinobacteria	Corynebacteriales
Actinobacteria	Actinobacteria	Corynebacteriales
Actinobacteria	Actinobacteria	Corynebacteriales
Actinobacteria	Actinobacteria	Corynebacteriales
Actinobacteria	Actinobacteria	Corynebacteriales
Actinobacteria	Actinobacteria	Corynebacteriales
Actinobacteria	Actinobacteria	Corynebacteriales
Firmicutes	Bacilli	Bacillales
Firmicutes	Bacilli	Bacillales
Firmicutes	Bacilli	Bacillales
Firmicutes	Bacilli	Bacillales
Firmicutes	Bacilli	Bacillales
Firmicutes	Bacilli	Bacillales
Firmicutes	Bacilli	Bacillales
Unknown	Unknown	Unknown
Unknown	Unknown	Unknown
Firmicutes	Bacilli	Bacillales
Unknown	Unknown	Unknown
Unknown	Unknown	Unknown
Firmicutes	Bacilli	Bacillales
Firmicutes	Bacilli	Bacillales
Firmicutes	Bacilli	Bacillales
Firmicutes	Bacilli	Bacillales

Unknown	Unknown	Unknown
Unknown	Unknown	Unknown
Unknown	Unknown	Unknown
Firmicutes	Bacilli	Bacillales
Firmicutes	Bacilli	Bacillales
Firmicutes	Bacilli	Bacillales
Firmicutes	Bacilli	Bacillales
Firmicutes	Bacilli	Bacillales
Actinobacteria	Actinobacteria	Micrococcales
Actinobacteria	Actinobacteria	Micrococcales
Actinobacteria	Actinobacteria	Micrococcales
Actinobacteria	Actinobacteria	Micrococcales
Actinobacteria	Actinobacteria	Micrococcales
Actinobacteria	Actinobacteria	Micrococcales
Actinobacteria	Actinobacteria	Micrococcales
Actinobacteria	Actinobacteria	Micrococcales
Actinobacteria	Actinobacteria	Micrococcales
Actinobacteria	Actinobacteria	Micrococcales
Actinobacteria	Actinobacteria	Micrococcales
Actinobacteria	Actinobacteria	Micrococcales
Actinobacteria	Actinobacteria	Micrococcales
Actinobacteria	Actinobacteria	Micrococcales
Bacteroidetes	Flavobacteriia	Flavobacteriales
Bacteroidetes	Flavobacteriia	Flavobacteriales
Firmicutes	Clostridia	Clostridiales
Firmicutes	Clostridia	Clostridiales
Firmicutes	Clostridia	Clostridiales
Firmicutes	Clostridia	Clostridiales
Firmicutes	Clostridia	Clostridiales
Unknown	Unknown	Unknown
Bacteroidetes	Bacteroidia	Bacteroidales
Bacteroidetes	Flavobacteriia	Flavobacteriales
Firmicutes	Bacilli	Bacillales
Deinococcus-Thermus	Deinococci	Thermales
Deinococcus-Thermus	Deinococci	Thermales
Deinococcus-Thermus	Deinococci	Thermales
Actinobacteria	Coriobacteriia	Eggerthellales
Firmicutes	Bacilli	Lactobacillales
Firmicutes	Bacilli	Lactobacillales
Firmicutes	Bacilli	Lactobacillales
Firmicutes	Bacilli	Lactobacillales
Proteobacteria	Gammaproteobacteria	Pseudomonadales
Proteobacteria	Gammaproteobacteria	Pseudomonadales
Proteobacteria	Gammaproteobacteria	Vibrionales
Proteobacteria	Gammaproteobacteria	Vibrionales
Proteobacteria	Gammaproteobacteria	Vibrionales

Proteobacteria	Gammaproteobacteria	Vibrionales
Proteobacteria	Gammaproteobacteria	Vibrionales
Cyanobacteria	Cyanophyceae	Synechococcales
Proteobacteria	Gammaproteobacteria	Pseudomonadales
Unknown	Unknown	Unknown
Proteobacteria	Gammaproteobacteria	Enterobacterales
Unknown	Unknown	Unknown
Actinobacteria	Actinobacteria	Streptomycetales
Actinobacteria	Actinobacteria	Streptomycetales
Actinobacteria	Actinobacteria	Streptomycetales
Actinobacteria	Actinobacteria	Streptomycetales
Actinobacteria	Actinobacteria	Streptomycetales
Actinobacteria	Actinobacteria	Micrococcales
Actinobacteria	Actinobacteria	Micrococcales
Actinobacteria	Actinobacteria	Micrococcales
Actinobacteria	Actinobacteria	Micrococcales
Actinobacteria	Actinobacteria	Micrococcales
Actinobacteria	Actinobacteria	Micrococcales
Actinobacteria	Actinobacteria	Micrococcales
Actinobacteria	Actinobacteria	Micrococcales
Actinobacteria	Actinobacteria	Micrococcales
Actinobacteria	Actinobacteria	Micrococcales
Actinobacteria	Actinobacteria	Micrococcales
Actinobacteria	Actinobacteria	Micrococcales
Actinobacteria	Actinobacteria	Micrococcales
Actinobacteria	Actinobacteria	Streptomycetales
Actinobacteria	Actinobacteria	Streptomycetales
Actinobacteria	Actinobacteria	Streptomycetales
Actinobacteria	Actinobacteria	Streptomycetales
Actinobacteria	Actinobacteria	Streptomycetales
Actinobacteria	Actinobacteria	Streptomycetales
Actinobacteria	Actinobacteria	Streptomycetales
Actinobacteria	Actinobacteria	Streptomycetales
Actinobacteria	Actinobacteria	Streptomycetales
Actinobacteria	Actinobacteria	Streptomycetales
Actinobacteria	Actinobacteria	Streptomycetales
Actinobacteria	Actinobacteria	Streptomycetales
Actinobacteria	Actinobacteria	Streptomycetales
Actinobacteria	Actinobacteria	Streptomycetales
Actinobacteria	Actinobacteria	Streptomycetales
Proteobacteria	Gammaproteobacteria	Pasteurellales
Unknown	Unknown	Unknown
Proteobacteria	Betaproteobacteria	Burkholderiales
Proteobacteria	Gammaproteobacteria	Pseudomonadales
Proteobacteria	Gammaproteobacteria	Pseudomonadales
Proteobacteria	Alphaproteobacteria	Rhizobiales

Proteobacteria	Alphaproteobacteria	Rhizobiales
Proteobacteria	Alphaproteobacteria	Rhizobiales
Proteobacteria	Gammaproteobacteria	Enterobacterales
Proteobacteria	Gammaproteobacteria	Enterobacterales
Proteobacteria	Gammaproteobacteria	Enterobacterales
Proteobacteria	Alphaproteobacteria	Rhizobiales
Proteobacteria	Alphaproteobacteria	Rhizobiales
Proteobacteria	Alphaproteobacteria	Pelagibacterales
Proteobacteria	Alphaproteobacteria	Rhodobacterales
Proteobacteria	Alphaproteobacteria	Rhodobacterales
Proteobacteria	Alphaproteobacteria	Rhodobacterales
Unknown	Unknown	Unknown
Proteobacteria	Epsilonproteobacteria	Unknown
Unknown	Unknown	Unknown
Proteobacteria	Gammaproteobacteria	Vibrionales
Proteobacteria	Gammaproteobacteria	Vibrionales
Cyanobacteria	Cyanophyceae	Oscillatoriales
Unknown	Unknown	Unknown
Proteobacteria	Alphaproteobacteria	Rhizobiales
Proteobacteria	Alphaproteobacteria	Rhizobiales
Proteobacteria	Alphaproteobacteria	Rhizobiales
Firmicutes	Bacilli	Lactobacillales
Firmicutes	Bacilli	Lactobacillales
Firmicutes	Bacilli	Lactobacillales
Firmicutes	Bacilli	Lactobacillales
Firmicutes	Bacilli	Lactobacillales
Unknown	Unknown	Unknown
Firmicutes	Clostridia	Clostridiales
Firmicutes	Clostridia	Clostridiales
Proteobacteria	Gammaproteobacteria	Aeromonadales
Proteobacteria	Gammaproteobacteria	Aeromonadales
Proteobacteria	Gammaproteobacteria	Aeromonadales
Proteobacteria	Gammaproteobacteria	Vibrionales
Proteobacteria	Gammaproteobacteria	Vibrionales
Proteobacteria	Gammaproteobacteria	Vibrionales
Proteobacteria	Gammaproteobacteria	Vibrionales
Unknown	Unknown	Unknown
Unknown	Unknown	Unknown
Unknown	Unknown	Unknown
Firmicutes	Clostridia	Clostridiales
Firmicutes	Clostridia	Clostridiales
Bacteroidetes	Flavobacteriia	Flavobacteriales
Proteobacteria	Gammaproteobacteria	Oceanospirillales
Proteobacteria	Betaproteobacteria	Burkholderiales
Proteobacteria	Betaproteobacteria	Burkholderiales
Proteobacteria	Betaproteobacteria	Burkholderiales

Proteobacteria	Betaproteobacteria	Burkholderiales
Proteobacteria	Gammaproteobacteria	Pseudomonadales
Proteobacteria	Betaproteobacteria	Burkholderiales
Proteobacteria	Betaproteobacteria	Burkholderiales
Unknown	Unknown	Unknown
Unknown	Unknown	Unknown
Bacteroidetes	Bacteroidia	Bacteroidales
Unknown	Unknown	Unknown
Proteobacteria	Betaproteobacteria	Burkholderiales
Proteobacteria	Alphaproteobacteria	Caulobacterales
Proteobacteria	Alphaproteobacteria	Pelagibacterales
Unknown	Unknown	Unknown
Proteobacteria	Alphaproteobacteria	Rhodobacterales
Proteobacteria	Alphaproteobacteria	Rhodobacterales
Proteobacteria	Gammaproteobacteria	Vibrionales
Proteobacteria	Gammaproteobacteria	Vibrionales
Proteobacteria	Gammaproteobacteria	Vibrionales
Proteobacteria	Gammaproteobacteria	Vibrionales
Proteobacteria	Gammaproteobacteria	Alteromonadales
Proteobacteria	Gammaproteobacteria	Vibrionales
Proteobacteria	Gammaproteobacteria	Vibrionales
Proteobacteria	Gammaproteobacteria	Vibrionales
Proteobacteria	Gammaproteobacteria	Vibrionales
Proteobacteria	Gammaproteobacteria	Alteromonadales
Cyanobacteria	Cyanophyceae	Chroococcales
Proteobacteria	Gammaproteobacteria	Vibrionales
Proteobacteria	Gammaproteobacteria	Alteromonadales
Proteobacteria	Gammaproteobacteria	Alteromonadales
Cyanobacteria	Cyanophyceae	Chroococcales
Bacteroidetes	Flavobacteriia	Flavobacteriales
Unknown	Unknown	Unknown
Firmicutes	Bacilli	Lactobacillales
Firmicutes	Bacilli	Lactobacillales
Firmicutes	Bacilli	Lactobacillales
Firmicutes	Bacilli	Lactobacillales
Unknown	Unknown	Unknown
Unknown	Unknown	Unknown
Firmicutes	Bacilli	Lactobacillales
Firmicutes	Bacilli	Lactobacillales
Firmicutes	Bacilli	Lactobacillales
Firmicutes	Bacilli	Lactobacillales
Firmicutes	Bacilli	Lactobacillales
Firmicutes	Bacilli	Lactobacillales
Firmicutes	Bacilli	Lactobacillales
Firmicutes	Bacilli	Bacillales
Firmicutes	Bacilli	Lactobacillales

Firmicutes	Bacilli	Lactobacillales
Firmicutes	Bacilli	Lactobacillales
Firmicutes	Bacilli	Lactobacillales
Firmicutes	Bacilli	Lactobacillales
Firmicutes	Bacilli	Lactobacillales
Firmicutes	Bacilli	Lactobacillales
Actinobacteria	Actinobacteria	Micrococcales
Actinobacteria	Actinobacteria	Micrococcales
Actinobacteria	Actinobacteria	Micrococcales
Actinobacteria	Actinobacteria	Micrococcales
Actinobacteria	Actinobacteria	Micrococcales
Firmicutes	Bacilli	Lactobacillales
Firmicutes	Bacilli	Lactobacillales
Firmicutes	Bacilli	Lactobacillales
Firmicutes	Bacilli	Lactobacillales
Firmicutes	Bacilli	Bacillales
Firmicutes	Bacilli	Bacillales
Firmicutes	Bacilli	Bacillales
Firmicutes	Bacilli	Bacillales
Unknown	Unknown	Unknown
Firmicutes	Bacilli	Bacillales
Firmicutes	Bacilli	Bacillales
Firmicutes	Bacilli	Bacillales
Firmicutes	Bacilli	Bacillales
Firmicutes	Bacilli	Bacillales
Firmicutes	Bacilli	Bacillales
Firmicutes	Bacilli	Bacillales
Firmicutes	Bacilli	Bacillales
Firmicutes	Bacilli	Bacillales
Proteobacteria	Gammaproteobacteria	Enterobacterales
Proteobacteria	Gammaproteobacteria	Enterobacterales
Proteobacteria	Gammaproteobacteria	Enterobacterales
Proteobacteria	Gammaproteobacteria	Enterobacterales
Proteobacteria	Betaproteobacteria	Burkholderiales
Proteobacteria	Gammaproteobacteria	Pseudomonadales
Proteobacteria	Gammaproteobacteria	Pseudomonadales
Proteobacteria	Gammaproteobacteria	Pseudomonadales
Proteobacteria	Gammaproteobacteria	Pseudomonadales
Proteobacteria	Gammaproteobacteria	Vibrionales
Proteobacteria	Gammaproteobacteria	Vibrionales
Proteobacteria	Gammaproteobacteria	Vibrionales
Proteobacteria	Gammaproteobacteria	Vibrionales
Proteobacteria	Gammaproteobacteria	Pseudomonadales
Proteobacteria	Gammaproteobacteria	Enterobacterales
Proteobacteria	Gammaproteobacteria	Enterobacterales
Deinococcus-Thermus	Deinococci	Thermales

Deinococcus-Thermus	Deinococci	Thermales
Deinococcus-Thermus	Deinococci	Thermales
Deinococcus-Thermus	Deinococci	Thermales
Proteobacteria	Gammaproteobacteria	Pseudomonadales
Proteobacteria	Gammaproteobacteria	Pseudomonadales
Proteobacteria	Gammaproteobacteria	Pseudomonadales
Actinobacteria	Actinobacteria	Streptomycetales
Actinobacteria	Actinobacteria	Streptomycetales
Actinobacteria	Actinobacteria	Streptomycetales
Actinobacteria	Actinobacteria	Streptomycetales
Actinobacteria	Actinobacteria	Streptomycetales
Actinobacteria	Actinobacteria	Streptomycetales
Actinobacteria	Actinobacteria	Streptomycetales
Actinobacteria	Actinobacteria	Streptomycetales
Actinobacteria	Actinobacteria	Streptomycetales
Actinobacteria	Actinobacteria	Streptomycetales
Actinobacteria	Actinobacteria	Streptomycetales
Actinobacteria	Actinobacteria	Streptomycetales
Proteobacteria	Gammaproteobacteria	Vibrionales
Proteobacteria	Gammaproteobacteria	Vibrionales
Proteobacteria	Gammaproteobacteria	Vibrionales
Proteobacteria	Gammaproteobacteria	Vibrionales
Proteobacteria	Gammaproteobacteria	Vibrionales
Proteobacteria	Gammaproteobacteria	Vibrionales
Proteobacteria	Gammaproteobacteria	Vibrionales
Proteobacteria	Gammaproteobacteria	Vibrionales
Proteobacteria	Gammaproteobacteria	Alteromonadales
Proteobacteria	Gammaproteobacteria	Aeromonadales
Proteobacteria	Gammaproteobacteria	Aeromonadales
Proteobacteria	Gammaproteobacteria	Enterobacterales
Proteobacteria	Gammaproteobacteria	Aeromonadales
Unknown	Unknown	Unknown
Unknown	Unknown	Unknown
Unknown	Unknown	Unknown
Unknown	Unknown	Unknown
Unknown	Unknown	Unknown
Unknown	Unknown	Unknown
Unknown	Unknown	Unknown
Unknown	Unknown	Unknown
Unknown	Unknown	Unknown
Unknown	Unknown	Unknown
Unknown	Unknown	Unknown
Unknown	Unknown	Unknown
Unknown	Unknown	Unknown
Unknown	Unknown	Unknown
Unknown	Unknown	Unknown

Unknown	Unknown	Unknown
Firmicutes	Bacilli	Bacillales
Firmicutes	Bacilli	Bacillales
Firmicutes	Bacilli	Bacillales
Firmicutes	Bacilli	Bacillales
Unknown	Unknown	Unknown
Firmicutes	Bacilli	Bacillales
Firmicutes	Bacilli	Bacillales
Firmicutes	Bacilli	Bacillales
Firmicutes	Bacilli	Bacillales
Firmicutes	Bacilli	Bacillales
Firmicutes	Bacilli	Bacillales
Firmicutes	Bacilli	Bacillales
Firmicutes	Bacilli	Bacillales
Firmicutes	Bacilli	Bacillales
Firmicutes	Bacilli	Bacillales
Unknown	Unknown	Unknown
Deinococcus-Thermus	Deinococci	Thermales
Bacteroidetes	Flavobacteriia	Flavobacteriales
Proteobacteria	Gammaproteobacteria	Vibrionales
Proteobacteria	Gammaproteobacteria	Vibrionales
Proteobacteria	Gammaproteobacteria	Vibrionales
Bacteroidetes	Flavobacteriia	Flavobacteriales
Bacteroidetes	Flavobacteriia	Flavobacteriales
Bacteroidetes	Flavobacteriia	Flavobacteriales
Actinobacteria	Actinobacteria	Corynebacteriales
Actinobacteria	Actinobacteria	Corynebacteriales
Actinobacteria	Actinobacteria	Corynebacteriales
Actinobacteria	Actinobacteria	Corynebacteriales
Proteobacteria	Alphaproteobacteria	Rhodobacterales
Proteobacteria	Alphaproteobacteria	Rhodobacterales
Proteobacteria	Alphaproteobacteria	Rhodobacterales
Proteobacteria	Alphaproteobacteria	Rhodobacterales
Proteobacteria	Alphaproteobacteria	Rhodobacterales
Proteobacteria	Alphaproteobacteria	Rhodobacterales
Proteobacteria	Alphaproteobacteria	Rhodobacterales
Proteobacteria	Alphaproteobacteria	Rhizobiales
Proteobacteria	Alphaproteobacteria	Rhizobiales
Actinobacteria	Actinobacteria	Streptomycetales
Actinobacteria	Actinobacteria	Streptomycetales
Actinobacteria	Actinobacteria	Streptomycetales
Actinobacteria	Actinobacteria	Streptomycetales
Actinobacteria	Actinobacteria	Streptomycetales
Actinobacteria	Actinobacteria	Streptomycetales
Actinobacteria	Actinobacteria	Streptomycetales
Actinobacteria	Actinobacteria	Streptomycetales

Actinobacteria	Actinobacteria	Streptomycetales
Actinobacteria	Actinobacteria	Streptomycetales
Actinobacteria	Actinobacteria	Streptomycetales
Proteobacteria	Gammaproteobacteria	Xanthomonadales
Proteobacteria	Gammaproteobacteria	Pseudomonadales
Proteobacteria	Gammaproteobacteria	Pseudomonadales
Actinobacteria	Actinobacteria	Micrococcales
Actinobacteria	Actinobacteria	Micrococcales
Actinobacteria	Actinobacteria	Micrococcales
Firmicutes	Bacilli	Lactobacillales
Firmicutes	Bacilli	Lactobacillales
Firmicutes	Bacilli	Bacillales
Firmicutes	Bacilli	Bacillales
Firmicutes	Bacilli	Bacillales
Firmicutes	Clostridia	Clostridiales
Bacteroidetes	Flavobacteriia	Flavobacteriales
Firmicutes	Clostridia	Clostridiales
Firmicutes	Bacilli	Bacillales
Firmicutes	Bacilli	Bacillales
Unknown	Unknown	Unknown
Unknown	Unknown	Unknown
Unknown	Unknown	Unknown
Firmicutes	Bacilli	Bacillales
Unknown	Unknown	Unknown
Unknown	Unknown	Unknown
Firmicutes	Bacilli	Bacillales
Unknown	Unknown	Unknown
Firmicutes	Bacilli	Bacillales
Bacteroidetes	Flavobacteriia	Flavobacteriales
Firmicutes	Bacilli	Bacillales
Firmicutes	Bacilli	Bacillales
Firmicutes	Bacilli	Bacillales
Firmicutes	Bacilli	Bacillales
Proteobacteria	Gammaproteobacteria	Vibrionales
Proteobacteria	Gammaproteobacteria	Vibrionales
Proteobacteria	Gammaproteobacteria	Vibrionales
Firmicutes	Bacilli	Bacillales
Firmicutes	Bacilli	Bacillales
Firmicutes	Bacilli	Bacillales
Firmicutes	Bacilli	Bacillales
Firmicutes	Bacilli	Bacillales
Unknown	Unknown	Unknown
Firmicutes	Bacilli	Bacillales
Firmicutes	Bacilli	Bacillales
Firmicutes	Bacilli	Bacillales
Firmicutes	Bacilli	Bacillales

Firmicutes	Bacilli	Bacillales
Firmicutes	Bacilli	Bacillales
Firmicutes	Bacilli	Bacillales
Firmicutes	Bacilli	Bacillales
Firmicutes	Bacilli	Bacillales
Unknown	Unknown	Unknown
Firmicutes	Bacilli	Bacillales
Firmicutes	Bacilli	Bacillales
Firmicutes	Bacilli	Bacillales
Firmicutes	Bacilli	Bacillales
Firmicutes	Clostridia	Clostridiales
Firmicutes	Clostridia	Clostridiales
Unknown	Unknown	Unknown
Firmicutes	Clostridia	Clostridiales
Firmicutes	Clostridia	Clostridiales
Firmicutes	Clostridia	Clostridiales
Firmicutes	Clostridia	Clostridiales
Firmicutes	Clostridia	Clostridiales
Firmicutes	Clostridia	Clostridiales
Firmicutes	Clostridia	Clostridiales
Firmicutes	Clostridia	Clostridiales
Firmicutes	Clostridia	Clostridiales
Proteobacteria	Gammaproteobacteria	Vibrionales
Proteobacteria	Gammaproteobacteria	Vibrionales
Proteobacteria	Gammaproteobacteria	Vibrionales
Proteobacteria	Gammaproteobacteria	Vibrionales
Proteobacteria	Gammaproteobacteria	Vibrionales
Proteobacteria	Gammaproteobacteria	Alteromonadales
Bacteroidetes	Flavobacteriia	Flavobacteriales
Proteobacteria	Gammaproteobacteria	Oceanospirillales
Unknown	Unknown	Unknown
Proteobacteria	Gammaproteobacteria	Pseudomonadales
Proteobacteria	Gammaproteobacteria	Pseudomonadales
Proteobacteria	Gammaproteobacteria	Pseudomonadales
Proteobacteria	Gammaproteobacteria	Pseudomonadales
Actinobacteria	Actinobacteria	Micrococcales
Actinobacteria	Actinobacteria	Micrococcales
Actinobacteria	Actinobacteria	Micrococcales
Actinobacteria	Actinobacteria	Micrococcales
Actinobacteria	Actinobacteria	Micrococcales
Actinobacteria	Actinobacteria	Micrococcales
Actinobacteria	Actinobacteria	Micrococcales
Actinobacteria	Actinobacteria	Micrococcales
Actinobacteria	Actinobacteria	Micrococcales
Actinobacteria	Actinobacteria	Micrococcales
Actinobacteria	Actinobacteria	Streptomycetales

Actinobacteria	Actinobacteria	Corynebacteriales
Actinobacteria	Actinobacteria	Corynebacteriales
Actinobacteria	Actinobacteria	Corynebacteriales
Actinobacteria	Actinobacteria	Corynebacteriales
Cyanobacteria	Cyanophyceae	Synechococcales
Cyanobacteria	Cyanophyceae	Synechococcales
Cyanobacteria	Cyanophyceae	Synechococcales
Cyanobacteria	Cyanophyceae	Synechococcales
Cyanobacteria	Cyanophyceae	Synechococcales
Cyanobacteria	Cyanophyceae	Synechococcales
Cyanobacteria	Cyanophyceae	Synechococcales
Proteobacteria	Gammaproteobacteria	Alteromonadales
Proteobacteria	Gammaproteobacteria	Vibrionales
Proteobacteria	Gammaproteobacteria	Vibrionales
Proteobacteria	Gammaproteobacteria	Vibrionales
Firmicutes	Bacilli	Lactobacillales
Firmicutes	Bacilli	Lactobacillales
Actinobacteria	Actinobacteria	Micrococcales
Actinobacteria	Actinobacteria	Micrococcales
Actinobacteria	Actinobacteria	Micrococcales
Actinobacteria	Actinobacteria	Micrococcales
Actinobacteria	Actinobacteria	Micrococcales
Actinobacteria	Actinobacteria	Micrococcales
Actinobacteria	Actinobacteria	Micrococcales
Actinobacteria	Actinobacteria	Streptomycetales
Actinobacteria	Actinobacteria	Streptomycetales
Firmicutes	Bacilli	Lactobacillales
Firmicutes	Bacilli	Lactobacillales
Firmicutes	Bacilli	Lactobacillales
Firmicutes	Clostridia	Clostridiales
Unknown	Unknown	Unknown
Unknown	Unknown	Unknown
Unknown	Unknown	Unknown
Candidatus Saccharibacteria	Unknown	Unknown
Unknown	Unknown	Unknown
Actinobacteria	Actinobacteria	Micrococcales
Actinobacteria	Actinobacteria	Propionibacteriales
Actinobacteria	Actinobacteria	Propionibacteriales
Actinobacteria	Actinobacteria	Propionibacteriales
Actinobacteria	Actinobacteria	Corynebacteriales
Proteobacteria	Gammaproteobacteria	Pasteurellales
Actinobacteria	Actinobacteria	Corynebacteriales
Actinobacteria	Actinobacteria	Corynebacteriales
Actinobacteria	Actinobacteria	Corynebacteriales
Firmicutes	Bacilli	Lactobacillales
Firmicutes	Erysipelotrichia	Erysipelotrichales

Firmicutes	Bacilli	Lactobacillales
Firmicutes	Bacilli	Lactobacillales
Firmicutes	Bacilli	Lactobacillales
Actinobacteria	Actinobacteria	Propionibacteriales
Actinobacteria	Actinobacteria	Propionibacteriales
Actinobacteria	Actinobacteria	Propionibacteriales
Actinobacteria	Actinobacteria	Micrococcales
Actinobacteria	Actinobacteria	Micrococcales
Firmicutes	Clostridia	Thermoanaerobacterales
Firmicutes	Bacilli	Lactobacillales
Firmicutes	Bacilli	Bacillales
Firmicutes	Clostridia	Clostridiales
Firmicutes	Clostridia	Clostridiales
Firmicutes	Bacilli	Lactobacillales
Firmicutes	Bacilli	Lactobacillales
Firmicutes	Bacilli	Lactobacillales
Firmicutes	Bacilli	Lactobacillales
Actinobacteria	Actinobacteria	Micrococcales
Firmicutes	Bacilli	Lactobacillales
Firmicutes	Bacilli	Lactobacillales
Firmicutes	Clostridia	Clostridiales
Firmicutes	Clostridia	Clostridiales
Firmicutes	Bacilli	Bacillales
Firmicutes	Bacilli	Bacillales
Firmicutes	Bacilli	Bacillales
Unknown	Unknown	Unknown
Unknown	Unknown	Unknown
Actinobacteria	Actinobacteria	Micrococcales
Actinobacteria	Actinobacteria	Micrococcales
Actinobacteria	Actinobacteria	Micrococcales
Actinobacteria	Actinobacteria	Micrococcales
Actinobacteria	Actinobacteria	Micrococcales
Actinobacteria	Actinobacteria	Micrococcales
Actinobacteria	Actinobacteria	Micrococcales
Actinobacteria	Actinobacteria	Micrococcales
Actinobacteria	Actinobacteria	Micrococcales
Actinobacteria	Actinobacteria	Micrococcales
Actinobacteria	Actinobacteria	Micrococcales
Cyanobacteria	Cyanophyceae	Chroococcales
Firmicutes	Bacilli	Bacillales
Firmicutes	Bacilli	Bacillales
Unknown	Unknown	Unknown
Cyanobacteria	Cyanophyceae	Oscillatoriales
Actinobacteria	Actinobacteria	Micrococcales
Actinobacteria	Actinobacteria	Corynebacteriales
Actinobacteria	Actinobacteria	Micrococcales

Actinobacteria	Actinobacteria	Micrococcales
Actinobacteria	Actinobacteria	Micrococcales
Actinobacteria	Actinobacteria	Micrococcales
Actinobacteria	Actinobacteria	Micrococcales
Actinobacteria	Actinobacteria	Micrococcales
Actinobacteria	Actinobacteria	Micrococcales
Actinobacteria	Actinobacteria	Micrococcales
Actinobacteria	Actinobacteria	Micrococcales
Actinobacteria	Actinobacteria	Micrococcales
Actinobacteria	Actinobacteria	Micrococcales
Actinobacteria	Actinobacteria	Micrococcales
Actinobacteria	Actinobacteria	Micrococcales
Actinobacteria	Actinobacteria	Micrococcales
Proteobacteria	Gammaproteobacteria	Xanthomonadales
Deinococcus-Thermus	Deinococci	Thermales
Deinococcus-Thermus	Deinococci	Thermales
Deinococcus-Thermus	Deinococci	Thermales
Firmicutes	Bacilli	Lactobacillales
Firmicutes	Bacilli	Lactobacillales
Firmicutes	Bacilli	Lactobacillales
Firmicutes	Bacilli	Lactobacillales
Firmicutes	Bacilli	Lactobacillales
Firmicutes	Bacilli	Lactobacillales
Proteobacteria	Alphaproteobacteria	Rhizobiales
Proteobacteria	Alphaproteobacteria	Rhizobiales
Proteobacteria	Gammaproteobacteria	Enterobacterales
Proteobacteria	Gammaproteobacteria	Enterobacterales
Proteobacteria	Gammaproteobacteria	Enterobacterales
Proteobacteria	Gammaproteobacteria	Enterobacterales
Proteobacteria	Gammaproteobacteria	Enterobacterales
Proteobacteria	Gammaproteobacteria	Enterobacterales
Proteobacteria	Gammaproteobacteria	Vibrionales
Proteobacteria	Gammaproteobacteria	Enterobacterales
Proteobacteria	Gammaproteobacteria	Enterobacterales
Proteobacteria	Gammaproteobacteria	Enterobacterales
Actinobacteria	Actinobacteria	Micrococcales
Actinobacteria	Actinobacteria	Streptosporangiales
Actinobacteria	Actinobacteria	Streptosporangiales
Firmicutes	Bacilli	Bacillales
Firmicutes	Bacilli	Bacillales
Firmicutes	Bacilli	Bacillales
Firmicutes	Bacilli	Bacillales
Proteobacteria	Alphaproteobacteria	Rhodobacterales
Proteobacteria	Alphaproteobacteria	Sphingomonadales
Proteobacteria	Alphaproteobacteria	Rhodobacterales
Proteobacteria	Alphaproteobacteria	Rhodobacterales

Proteobacteria	Alphaproteobacteria	Rhodobacterales
Proteobacteria	Alphaproteobacteria	Rhodobacterales
Proteobacteria	Alphaproteobacteria	Rhodobacterales
Proteobacteria	Gammaproteobacteria	Vibrionales
Proteobacteria	Gammaproteobacteria	Alteromonadales
Proteobacteria	Gammaproteobacteria	Vibrionales
Proteobacteria	Gammaproteobacteria	Vibrionales
Proteobacteria	Gammaproteobacteria	Alteromonadales
Proteobacteria	Gammaproteobacteria	Vibrionales
Proteobacteria	Gammaproteobacteria	Pseudomonadales
Bacteroidetes	Flavobacteriia	Flavobacteriales
Proteobacteria	Gammaproteobacteria	Pseudomonadales
Unknown	Unknown	Unknown
Actinobacteria	Actinobacteria	Micrococcales
Actinobacteria	Actinobacteria	Micrococcales
Actinobacteria	Actinobacteria	Micrococcales
Actinobacteria	Actinobacteria	Micrococcales
Firmicutes	Bacilli	Bacillales
Firmicutes	Bacilli	Bacillales
Unknown	Unknown	Unknown
Firmicutes	Bacilli	Bacillales
Actinobacteria	Actinobacteria	Micrococcales
Unknown	Unknown	Unknown
Unknown	Unknown	Unknown
Cyanobacteria	Cyanophyceae	Synechococcales
Cyanobacteria	Cyanophyceae	Synechococcales
Unknown	Unknown	Unknown
Actinobacteria	Actinobacteria	Streptomycetales
Actinobacteria	Actinobacteria	Streptomycetales
Actinobacteria	Actinobacteria	Streptomycetales
Unknown	Unknown	Unknown
Unknown	Unknown	Unknown
Unknown	Unknown	Unknown
Unknown	Unknown	Unknown
Unknown	Unknown	Unknown
Cyanobacteria	Cyanophyceae	Synechococcales
Cyanobacteria	Cyanophyceae	Synechococcales
Firmicutes	Bacilli	Lactobacillales
Unknown	Unknown	Unknown
Bacteroidetes	Flavobacteriia	Flavobacteriales
Cyanobacteria	Cyanophyceae	Synechococcales
Cyanobacteria	Cyanophyceae	Synechococcales
Cyanobacteria	Cyanophyceae	Synechococcales
Firmicutes	Bacilli	Bacillales
Actinobacteria	Actinobacteria	Micrococcales
Firmicutes	Clostridia	Clostridiales

Actinobacteria	Actinobacteria	Streptomycetales
Proteobacteria	Alphaproteobacteria	Rhodobacterales
Unknown	Unknown	Unknown
Proteobacteria	Gammaproteobacteria	Pseudomonadales
Proteobacteria	Gammaproteobacteria	Pseudomonadales
Actinobacteria	Actinobacteria	Micrococcales
Proteobacteria	Gammaproteobacteria	Xanthomonadales
Proteobacteria	Gammaproteobacteria	Pseudomonadales
Proteobacteria	Gammaproteobacteria	Pseudomonadales
Actinobacteria	Actinobacteria	Actinomycetales
Actinobacteria	Actinobacteria	Bifidobacteriales
Proteobacteria	Gammaproteobacteria	Enterobacterales
Deinococcus-Thermus	Deinococci	Thermales
Deinococcus-Thermus	Deinococci	Thermales
Firmicutes	Bacilli	Lactobacillales
Firmicutes	Bacilli	Bacillales
Proteobacteria	Betaproteobacteria	Burkholderiales
Unknown	Unknown	Unknown
Unknown	Unknown	Unknown
Unknown	Unknown	Unknown
Proteobacteria	Gammaproteobacteria	Xanthomonadales
Actinobacteria	Actinobacteria	Micrococcales
Proteobacteria	Alphaproteobacteria	Sphingomonadales
Firmicutes	Bacilli	Lactobacillales
Firmicutes	Bacilli	Bacillales
Unknown	Unknown	Unknown
Firmicutes	Clostridia	Clostridiales
Unknown	Unknown	Unknown
Unknown	Unknown	Unknown
Unknown	Unknown	Unknown
Firmicutes	Clostridia	Clostridiales
Unknown	Unknown	Unknown
Unknown	Unknown	Unknown
Firmicutes	Bacilli	Bacillales
Unknown	Unknown	Unknown
Actinobacteria	Actinobacteria	Micrococcales
Actinobacteria	Actinobacteria	Micrococcales
Firmicutes	Bacilli	Lactobacillales
Firmicutes	Bacilli	Lactobacillales
Firmicutes	Bacilli	Lactobacillales

Host family	Host genus
Mycobacteriaceae	Mycolicibacterium
Mycobacteriaceae	Mycolicibacterium
Mycobacteriaceae	Mycolicibacterium
Mycobacteriaceae	Mycolicibacterium
Mycobacteriaceae	Mycolicibacterium
Mycobacteriaceae	Mycolicibacterium
Mycobacteriaceae	Mycolicibacterium
Mycobacteriaceae	Mycolicibacterium
Mycobacteriaceae	Mycolicibacterium
Mycobacteriaceae	Mycolicibacterium
Mycobacteriaceae	Mycolicibacterium
Mycobacteriaceae	Mycolicibacterium
Mycobacteriaceae	Mycolicibacterium
Mycobacteriaceae	Mycolicibacterium
Mycobacteriaceae	Mycolicibacterium
Mycobacteriaceae	Mycolicibacterium
Mycobacteriaceae	Mycolicibacterium
Mycobacteriaceae	Mycolicibacterium
Mycobacteriaceae	Mycolicibacterium
Mycobacteriaceae	Mycolicibacterium
Mycobacteriaceae	Mycolicibacterium
Mycobacteriaceae	Mycolicibacterium
Mycobacteriaceae	Mycolicibacterium
Mycobacteriaceae	Mycolicibacterium
Mycobacteriaceae	Mycolicibacterium
Mycobacteriaceae	Mycolicibacterium
Mycobacteriaceae	Mycobacterium
Mycobacteriaceae	Mycolicibacterium
Gordoniaceae	Gordonia
Gordoniaceae	Gordonia
Gordoniaceae	Gordonia
Gordoniaceae	Gordonia
Gordoniaceae	Gordonia
Gordoniaceae	Gordonia
Unknown	Unknown
Gordoniaceae	Gordonia
Gordoniaceae	Gordonia
Gordoniaceae	Gordonia
Gordoniaceae	Gordonia
Gordoniaceae	Gordonia
Gordoniaceae	Gordonia
Tsukamurellaceae	Tsukamurella
Gordoniaceae	Gordonia
Gordoniaceae	Gordonia
Gordoniaceae	Gordonia

Nocardiaceae	Rhodococcus
Mycobacteriaceae	Mycobacterium
Mycobacteriaceae	Mycolicibacterium
Gordoniaceae	Gordonia
Gordoniaceae	Gordonia
Gordoniaceae	Gordonia
Gordoniaceae	Gordonia
Gordoniaceae	Gordonia
Gordoniaceae	Gordonia
Gordoniaceae	Gordonia
Gordoniaceae	Gordonia
Gordoniaceae	Gordonia
Gordoniaceae	Gordonia
Gordoniaceae	Gordonia
Gordoniaceae	Gordonia
Gordoniaceae	Gordonia
Gordoniaceae	Gordonia
Gordoniaceae	Gordonia
Gordoniaceae	Gordonia
Mycobacteriaceae	Mycolicibacterium
Mycobacteriaceae	Mycolicibacterium
Mycobacteriaceae	Mycobacterium
Mycobacteriaceae	Mycolicibacterium
Mycobacteriaceae	Mycolicibacterium
Mycobacteriaceae	Mycolicibacterium
Mycobacteriaceae	Mycolicibacterium
Mycobacteriaceae	Mycolicibacterium
Mycobacteriaceae	Mycolicibacterium
Mycobacteriaceae	Mycolicibacterium
Mycobacteriaceae	Mycolicibacterium
Mycobacteriaceae	Mycolicibacterium
Gordoniaceae	Gordonia
Gordoniaceae	Gordonia
Gordoniaceae	Gordonia
Gordoniaceae	Gordonia
Mycobacteriaceae	Mycolicibacterium
Mycobacteriaceae	Mycolicibacterium
Mycobacteriaceae	Mycolicibacterium
Mycobacteriaceae	Mycolicibacterium
Mycobacteriaceae	Mycolicibacterium
Mycobacteriaceae	Mycolicibacterium
Mycobacteriaceae	Mycolicibacterium
Mycobacteriaceae	Mycolicibacterium
Mycobacteriaceae	Mycolicibacterium
Mycobacteriaceae	Mycobacterium
Mycobacteriaceae	Mycolicibacterium

Mycobacteriaceae	Mycolicibacterium
Mycobacteriaceae	Mycobacterium
Mycobacteriaceae	Mycobacterium
Mycobacteriaceae	Mycolicibacterium
Mycobacteriaceae	Mycolicibacterium
Mycobacteriaceae	Mycolicibacterium
Mycobacteriaceae	Mycolicibacterium
Mycobacteriaceae	Mycolicibacterium
Mycobacteriaceae	Mycolicibacterium
Mycobacteriaceae	Mycolicibacterium
Mycobacteriaceae	Mycolicibacterium
Mycobacteriaceae	Mycolicibacterium
Mycobacteriaceae	Mycolicibacterium
Mycobacteriaceae	Mycolicibacterium
Mycobacteriaceae	Mycolicibacterium
Mycobacteriaceae	Mycolicibacterium
Mycobacteriaceae	Mycolicibacterium
Mycobacteriaceae	Mycolicibacterium
Mycobacteriaceae	Mycolicibacterium
Mycobacteriaceae	Mycolicibacterium
Micrococcaceae	Arthrobacter
Micrococcaceae	Arthrobacter
Micrococcaceae	Arthrobacter
Micrococcaceae	Arthrobacter
Micrococcaceae	Arthrobacter
Micrococcaceae	Arthrobacter
Micrococcaceae	Arthrobacter
Gordoniaceae	Gordonia
Nocardiaceae	Rhodococcus
Gordoniaceae	Gordonia
Gordoniaceae	Gordonia
Gordoniaceae	Gordonia
Unknown	Unknown
Nocardiaceae	Rhodococcus
Nocardiaceae	Rhodococcus
Mycobacteriaceae	Mycolicibacterium
Mycobacteriaceae	Mycolicibacterium
Mycobacteriaceae	Mycolicibacterium
Mycobacteriaceae	Mycolicibacterium
Mycobacteriaceae	Mycolicibacterium
Mycobacteriaceae	Mycolicibacterium
Mycobacteriaceae	Mycobacterium
Mycobacteriaceae	Mycobacterium
Gordoniaceae	Gordonia
Gordoniaceae	Gordonia
Gordoniaceae	Gordonia

Gordoniaceae	Gordonia
Gordoniaceae	Gordonia
Gordoniaceae	Gordonia
Gordoniaceae	Gordonia
Nocardiaceae	Nocardia
Tsukamurellaceae	Tsukamurella
Tsukamurellaceae	Tsukamurella
Gordoniaceae	Gordonia
Gordoniaceae	Gordonia
Gordoniaceae	Gordonia
Gordoniaceae	Gordonia
Gordoniaceae	Gordonia
Gordoniaceae	Gordonia
Gordoniaceae	Gordonia
Gordoniaceae	Gordonia
Gordoniaceae	Gordonia
Gordoniaceae	Gordonia
Gordoniaceae	Gordonia
Gordoniaceae	Gordonia
Gordoniaceae	Gordonia
Comamonadaceae	Acidovorax
Pseudomonadaceae	Pseudomonas
Alcaligenaceae	Achromobacter
Flavobacteriaceae	Cellulophaga
Vibrionaceae	Vibrio
Caulobacteraceae	Caulobacter
Caulobacteraceae	Caulobacter
Erythrobacteraceae	Erythrobacter
Unknown	Unknown
Oceanospirillaceae	Nitrincola
Pseudomonadaceae	Pseudomonas
Rhizobiaceae	Sinorhizobium
Rhizobiaceae	Sinorhizobium
Rhizobiaceae	Sinorhizobium
Pseudomonadaceae	Pseudomonas
Aeromonadaceae	Aeromonas
Synechococcaceae	Synechococcus
Synechococcaceae	Synechococcus
Unknown	Unknown
Synechococcaceae	Synechococcus
Synechococcaceae	Synechococcus
Synechococcaceae	Synechococcus
Xanthomonadaceae	Xanthomonas
Xanthomonadaceae	Xanthomonas
Xanthomonadaceae	Xanthomonas
Xanthomonadaceae	Xanthomonas

Xanthomonadaceae	Xanthomonas
Xanthomonadaceae	Xanthomonas
Aeromonadaceae	Aeromonas
Erwiniaceae	Pantoea
Enterobacteriaceae	Salmonella
Enterobacteriaceae	Salmonella
Morganellaceae	Proteus
Enterobacteriaceae	Raoultella
Enterobacteriaceae	Cronobacter
Enterobacteriaceae	Escherichia
Pseudomonadaceae	Pseudomonas
Pseudomonadaceae	Pseudomonas
Pseudomonadaceae	Pseudomonas
Unknown	Unknown
Pseudomonadaceae	Pseudomonas
Unknown	Unknown
Microcystaceae	Microcystis
Microcystaceae	Microcystis
Brucellaceae	Ochrobactrum
Sphingomonadaceae	Sphingomonas
Unknown	Unknown
Unknown	Unknown
Unknown	Unknown
Flavobacteriaceae	Flavobacterium
Alcaligenaceae	Achromobacter
Enterobacteriaceae	Enterobacter
Enterobacteriaceae	Escherichia
Enterobacteriaceae	Escherichia
Enterobacteriaceae	Escherichia
Enterobacteriaceae	Salmonella
Enterobacteriaceae	Salmonella
Enterobacteriaceae	Cronobacter
Enterobacteriaceae	Escherichia
Enterobacteriaceae	Klebsiella
Enterobacteriaceae	Klebsiella
Enterobacteriaceae	Klebsiella
Enterobacteriaceae	Klebsiella
Enterobacteriaceae	Klebsiella
Enterobacteriaceae	Klebsiella
Enterobacteriaceae	Escherichia
Aeromonadaceae	Aeromonas
Enterobacteriaceae	Salmonella
Enterobacteriaceae	Salmonella
Erwiniaceae	Pantoea
Unknown	Unknown
Enterobacteriaceae	Escherichia

Enterobacteriaceae	Escherichia
Enterobacteriaceae	Escherichia
Enterobacteriaceae	Escherichia
Pseudomonadaceae	Pseudomonas
Yersiniaceae	Serratia
Burkholderiaceae	Burkholderia
Xanthomonadaceae	Xanthomonas
Xanthomonadaceae	Xanthomonas
Pseudomonadaceae	Pseudomonas
Pseudomonadaceae	Pseudomonas
Pseudomonadaceae	Pseudomonas
Pseudomonadaceae	Pseudomonas
Pseudomonadaceae	Pseudomonas
Pseudomonadaceae	Pseudomonas
Pseudomonadaceae	Pseudomonas
Pseudomonadaceae	Pseudomonas
Pseudomonadaceae	Pseudomonas
Pseudomonadaceae	Pseudomonas
Unknown	Unknown
Unknown	Unknown
Unknown	Unknown
Pseudomonadaceae	Pseudomonas
Pseudomonadaceae	Pseudomonas
Pseudomonadaceae	Pseudomonas
Pasteurellaceae	Mannheimia
Pasteurellaceae	Aggregatibacter
Pasteurellaceae	Aggregatibacter
Xanthomonadaceae	Xanthomonas
Comamonadaceae	Delftia
Unknown	Unknown
Enterobacteriaceae	Cronobacter
Enterobacteriaceae	Klebsiella
Enterobacteriaceae	Klebsiella
Pseudomonadaceae	Pseudomonas
Gordoniaceae	Gordonia
Gordoniaceae	Gordonia
Gordoniaceae	Gordonia
Gordoniaceae	Gordonia
Gordoniaceae	Gordonia
Gordoniaceae	Gordonia
Gordoniaceae	Gordonia
Gordoniaceae	Gordonia
Gordoniaceae	Gordonia
Gordoniaceae	Gordonia
Gordoniaceae	Gordonia
Gordoniaceae	Gordonia

Gordoniaceae	Gordonia
Gordoniaceae	Gordonia
Gordoniaceae	Gordonia
Gordoniaceae	Gordonia
Gordoniaceae	Gordonia
Gordoniaceae	Gordonia
Mycobacteriaceae	Mycolicibacterium
Unknown	Unknown
Mycobacteriaceae	Mycolicibacterium
Mycobacteriaceae	Mycolicibacterium
Corynebacteriaceae	Corynebacterium
Corynebacteriaceae	Corynebacterium
Corynebacteriaceae	Corynebacterium
Corynebacteriaceae	Corynebacterium
Corynebacteriaceae	Corynebacterium
Corynebacteriaceae	Corynebacterium
Corynebacteriaceae	Corynebacterium
Corynebacteriaceae	Corynebacterium
Corynebacteriaceae	Corynebacterium
Corynebacteriaceae	Corynebacterium
Corynebacteriaceae	Corynebacterium
Gordoniaceae	Gordonia
Gordoniaceae	Gordonia
Gordoniaceae	Gordonia
Gordoniaceae	Gordonia
Nocardiaceae	Rhodococcus
Gordoniaceae	Gordonia
Gordoniaceae	Gordonia
Gordoniaceae	Gordonia
Gordoniaceae	Gordonia
Gordoniaceae	Gordonia
Gordoniaceae	Gordonia
Mycobacteriaceae	Mycolicibacterium
Mycobacteriaceae	Mycolicibacterium
Listeriaceae	Listeria
Unknown	Unknown
Listeriaceae	Listeria
Listeriaceae	Listeria
Listeriaceae	Listeria
Listeriaceae	Listeria
Listeriaceae	Listeria
Listeriaceae	Listeria
Listeriaceae	Listeria
Listeriaceae	Listeria
Lactobacillaceae	Lactobacillus
Lactobacillaceae	Lactobacillus

Lactobacillaceae	Lactobacillus
Lactobacillaceae	Lactobacillus
Lactobacillaceae	Lactobacillus
Lactobacillaceae	Lactobacillus
Lactobacillaceae	Lactobacillus
Streptococcaceae	Lactococcus
Streptococcaceae	Lactococcus
Streptococcaceae	Lactococcus
Streptococcaceae	Lactococcus
Streptococcaceae	Lactococcus
Streptococcaceae	Lactococcus
Streptococcaceae	Lactococcus
Streptococcaceae	Lactococcus
Streptococcaceae	Lactococcus
Enterococcaceae	Enterococcus
Enterococcaceae	Enterococcus
Enterococcaceae	Enterococcus
Enterococcaceae	Enterococcus
Enterococcaceae	Enterococcus
Enterococcaceae	Enterococcus
Enterococcaceae	Enterococcus
Enterococcaceae	Enterococcus
Enterococcaceae	Enterococcus
Enterococcaceae	Enterococcus
Unknown	Unknown
Unknown	Unknown
Enterococcaceae	Enterococcus
Enterococcaceae	Enterococcus
Enterococcaceae	Enterococcus
Enterococcaceae	Enterococcus
Enterococcaceae	Enterococcus
Enterococcaceae	Enterococcus
Enterococcaceae	Enterococcus
Enterococcaceae	Enterococcus
Enterococcaceae	Enterococcus
Enterococcaceae	Enterococcus
Enterococcaceae	Enterococcus
Enterococcaceae	Enterococcus
Enterococcaceae	Enterococcus
Enterococcaceae	Enterococcus
Enterococcaceae	Enterococcus
Enterococcaceae	Enterococcus
Enterococcaceae	Enterococcus
Streptococcaceae	Streptococcus
Enterococcaceae	Enterococcus
Enterococcaceae	Enterococcus

Enterococcaceae	Enterococcus
Enterococcaceae	Enterococcus
Enterococcaceae	Enterococcus
Enterococcaceae	Enterococcus
Enterococcaceae	Enterococcus
Aeromonadaceae	Aeromonas
Aeromonadaceae	Aeromonas
Aeromonadaceae	Aeromonas
Aeromonadaceae	Aeromonas
Aeromonadaceae	Aeromonas
Aeromonadaceae	Aeromonas
Aeromonadaceae	Aeromonas
Aeromonadaceae	Aeromonas
Aeromonadaceae	Aeromonas
Aeromonadaceae	Aeromonas
Aeromonadaceae	Aeromonas
Aeromonadaceae	Aeromonas
Aeromonadaceae	Aeromonas
Aeromonadaceae	Aeromonas
Aeromonadaceae	Aeromonas
Aeromonadaceae	Aeromonas
Aeromonadaceae	Aeromonas
Vibrionaceae	Vibrio
Vibrionaceae	Vibrio
Vibrionaceae	Vibrio
Vibrionaceae	Vibrio
Vibrionaceae	Vibrio
Vibrionaceae	Vibrio
Vibrionaceae	Vibrio
Vibrionaceae	Vibrio
Vibrionaceae	Vibrio
Vibrionaceae	Vibrio
Enterobacteriaceae	Escherichia
Pseudomonadaceae	Pseudomonas
Pseudomonadaceae	Pseudomonas
Pseudomonadaceae	Pseudomonas
Pseudomonadaceae	Pseudomonas
Pseudomonadaceae	Pseudomonas
Pelagibacteraceae	Candidatus Pelagibacter
Pelagibacteraceae	Candidatus Pelagibacter
Pelagibacteraceae	Candidatus Pelagibacter
Pseudoalteromonadaceae	Pseudoalteromonas
Alteromonadaceae	Marinobacter
Pseudoalteromonadaceae	Pseudoalteromonas
Pseudoalteromonadaceae	Pseudoalteromonas
Pelagibacteraceae	Candidatus Pelagibacter

Pelagibacteraceae	Candidatus Pelagibacter
Enterobacteriaceae	Salmonella
Erwiniaceae	Pantoea
Colwelliaceae	Colwellia
Unknown	Unknown
Unknown	Candidatus Puniceispirillum
Unknown	Unknown
Xanthomonadaceae	Xanthomonas
Aeromonadaceae	Aeromonas
Aeromonadaceae	Aeromonas
Aeromonadaceae	Aeromonas
Vibrionaceae	Vibrio
Vibrionaceae	Enterovibrio
Gordoniaceae	Gordonia
Gordoniaceae	Gordonia
Gordoniaceae	Gordonia
Gordoniaceae	Gordonia
Gordoniaceae	Gordonia
Gordoniaceae	Gordonia
Gordoniaceae	Gordonia
Gordoniaceae	Gordonia
Gordoniaceae	Gordonia
Gordoniaceae	Gordonia
Gordoniaceae	Gordonia
Gordoniaceae	Gordonia
Gordoniaceae	Gordonia
Gordoniaceae	Gordonia
Gordoniaceae	Gordonia
Gordoniaceae	Gordonia
Gordoniaceae	Gordonia
Gordoniaceae	Gordonia
Gordoniaceae	Gordonia
Gordoniaceae	Gordonia
Gordoniaceae	Gordonia
Gordoniaceae	Gordonia
Gordoniaceae	Gordonia
Gordoniaceae	Gordonia
Unknown	Unknown
Unknown	Unknown
Bacillaceae	Bacillus
Bacillaceae	Bacillus
Bacillaceae	Bacillus
Bacillaceae	Bacillus
Bacillaceae	Bacillus
Bacillaceae	Bacillus
Bacillaceae	Bacillus

Bacillaceae	Bacillus
Streptococcaceae	Streptococcus
Streptococcaceae	Streptococcus
Streptococcaceae	Streptococcus
Streptococcaceae	Streptococcus
Streptococcaceae	Streptococcus
Streptococcaceae	Streptococcus
Streptococcaceae	Streptococcus
Streptococcaceae	Streptococcus
Streptococcaceae	Streptococcus
Streptococcaceae	Streptococcus
Streptococcaceae	Streptococcus
Streptococcaceae	Streptococcus
Streptococcaceae	Streptococcus
Streptococcaceae	Streptococcus
Streptococcaceae	Streptococcus
Streptomycetaceae	Streptomyces
Streptomycetaceae	Streptomyces
Streptomycetaceae	Streptomyces
Streptomycetaceae	Streptomyces
Streptomycetaceae	Streptomyces
Streptomycetaceae	Streptomyces
Streptomycetaceae	Streptomyces
Streptomycetaceae	Streptomyces
Streptomycetaceae	Streptomyces
Streptomycetaceae	Streptomyces
Streptomycetaceae	Streptomyces
Streptomycetaceae	Streptomyces
Streptomycetaceae	Streptomyces
Streptomycetaceae	Streptomyces
Streptomycetaceae	Streptomyces
Streptomycetaceae	Streptomyces
Streptomycetaceae	Streptomyces
Streptomycetaceae	Streptomyces
Streptomycetaceae	Streptomyces
Streptomycetaceae	Streptomyces
Streptomycetaceae	Streptomyces
Streptomycetaceae	Streptomyces
Streptomycetaceae	Streptomyces
Streptomycetaceae	Streptomyces
Streptomycetaceae	Streptomyces
Streptomycetaceae	Streptomyces
Streptomycetaceae	Streptomyces
Streptomycetaceae	Streptomyces
Streptomycetaceae	Streptomyces
Streptomycetaceae	Streptomyces

Streptomycetaceae	Streptomyces
Streptomycetaceae	Streptomyces
Streptomycetaceae	Streptomyces
Streptomycetaceae	Streptomyces
Streptomycetaceae	Streptomyces
Streptomycetaceae	Streptomyces
Streptomycetaceae	Streptomyces
Streptomycetaceae	Streptomyces
Streptomycetaceae	Streptomyces
Streptomycetaceae	Streptomyces
Streptomycetaceae	Streptomyces
Streptomycetaceae	Streptomyces
Streptomycetaceae	Streptomyces
Streptomycetaceae	Streptomyces
Streptomycetaceae	Streptomyces
Streptomycetaceae	Streptomyces
Streptomycetaceae	Streptomyces
Streptomycetaceae	Streptomyces
Streptomycetaceae	Streptomyces
Streptomycetaceae	Streptomyces
Streptomycetaceae	Streptomyces
Streptomycetaceae	Streptomyces
Streptomycetaceae	Streptomyces
Streptomycetaceae	Streptomyces
Streptomycetaceae	Streptomyces
Enterococcaceae	Enterococcus
Enterococcaceae	Enterococcus
Enterococcaceae	Enterococcus
Enterococcaceae	Enterococcus
Enterococcaceae	Enterococcus
Enterococcaceae	Enterococcus
Unknown	Unknown
Enterococcaceae	Enterococcus
Enterococcaceae	Enterococcus
Enterococcaceae	Enterococcus
Enterococcaceae	Enterococcus
Enterococcaceae	Enterococcus
Enterococcaceae	Enterococcus
Enterococcaceae	Enterococcus
Enterococcaceae	Enterococcus
Enterococcaceae	Enterococcus
Unknown	Unknown
Enterococcaceae	Enterococcus
Unknown	Unknown
Enterococcaceae	Enterococcus
Enterococcaceae	Enterococcus

Enterococcaceae	Enterococcus
Unknown	Unknown
Streptococcaceae	Lactococcus
Leuconostocaceae	Leuconostoc
Leuconostocaceae	Leuconostoc
Leuconostocaceae	Leuconostoc
Leuconostocaceae	Leuconostoc
Streptococcaceae	Streptococcus
Streptococcaceae	Streptococcus
Lactobacillaceae	Lactobacillus
Lactobacillaceae	Lactobacillus
Lactobacillaceae	Lactobacillus
Lactobacillaceae	Lactobacillus
Lactobacillaceae	Lactobacillus
Lactobacillaceae	Lactobacillus
Lactobacillaceae	Lactobacillus
Lactobacillaceae	Lactobacillus
Lactobacillaceae	Lactobacillus
Lactobacillaceae	Lactobacillus
Lactobacillaceae	Lactobacillus
Lactobacillaceae	Lactobacillus
Lactobacillaceae	Lactobacillus
Lactobacillaceae	Lactobacillus
Lactobacillaceae	Lactobacillus
Lactobacillaceae	Lactobacillus
Lactobacillaceae	Lactobacillus
Lactobacillaceae	Lactobacillus
Lactobacillaceae	Lactobacillus
Lactobacillaceae	Lactobacillus
Lactobacillaceae	Lactobacillus
Bacillaceae	Bacillus
Bacillaceae	Bacillus
Bacillaceae	Bacillus
Bacillaceae	Bacillus
Bacillaceae	Virgibacillus
Bacillaceae	Bacillus
Bacillaceae	Bacillus
Bacillaceae	Bacillus
Bacillaceae	Bacillus
Bacillaceae	Bacillus
Unknown	Unknown
Bacillaceae	Bacillus
Bacillaceae	Bacillus
Unknown	Unknown
Bacillaceae	Bacillus
Bacillaceae	Bacillus

Bacillaceae	Bacillus
Bacillaceae	Bacillus
Bacillaceae	Bacillus
Bacillaceae	Bacillus
Bacillaceae	Bacillus
Bacillaceae	Bacillus
Unknown	Unknown
Bacillaceae	Bacillus
Bacillaceae	Bacillus
Bacillaceae	Bacillus
Bacillaceae	Bacillus
Bacillaceae	Bacillus
Unknown	Unknown
Bacillaceae	Bacillus
Bacillaceae	Bacillus
Bacillaceae	Bacillus
Bacillaceae	Bacillus
Bacillaceae	Bacillus
Streptococcaceae	Lactococcus
Streptococcaceae	Lactococcus
Streptococcaceae	Lactococcus
Streptococcaceae	Lactococcus
Enterococcaceae	Enterococcus
Enterococcaceae	Enterococcus
Listeriaceae	Brochothrix
Listeriaceae	Brochothrix
Enterococcaceae	Enterococcus
Enterococcaceae	Enterococcus
Enterococcaceae	Enterococcus
Streptococcaceae	Streptococcus
Listeriaceae	Brochothrix
Enterococcaceae	Enterococcus
Enterococcaceae	Enterococcus
Staphylococcaceae	Staphylococcus
Staphylococcaceae	Staphylococcus
Staphylococcaceae	Staphylococcus
Staphylococcaceae	Staphylococcus
Staphylococcaceae	Staphylococcus
Staphylococcaceae	Staphylococcus
Staphylococcaceae	Staphylococcus
Staphylococcaceae	Staphylococcus
Staphylococcaceae	Staphylococcus
Staphylococcaceae	Staphylococcus
Alcaligenaceae	Bordetella
Alcaligenaceae	Bordetella
Alcaligenaceae	Bordetella

Alcaligenaceae	Bordetella
Alcaligenaceae	Bordetella
Pseudomonadaceae	Pseudomonas
Pseudomonadaceae	Pseudomonas
Pseudomonadaceae	Pseudomonas
Pseudomonadaceae	Pseudomonas
Pseudomonadaceae	Pseudomonas
Pseudomonadaceae	Pseudomonas
Pseudomonadaceae	Pseudomonas
Unknown	Unknown
Yersiniaceae	Yersinia
Alcaligenaceae	Bordetella
Streptococcaceae	Streptococcus
Streptococcaceae	Streptococcus
Streptococcaceae	Streptococcus
Streptococcaceae	Streptococcus
Streptococcaceae	Streptococcus
Streptococcaceae	Streptococcus
Streptococcaceae	Streptococcus
Streptococcaceae	Streptococcus
Streptococcaceae	Streptococcus
Streptococcaceae	Streptococcus
Streptococcaceae	Streptococcus
Streptococcaceae	Streptococcus
Streptococcaceae	Streptococcus
Streptococcaceae	Streptococcus
Streptococcaceae	Streptococcus
Streptococcaceae	Streptococcus
Streptococcaceae	Streptococcus
Streptococcaceae	Streptococcus
Streptococcaceae	Streptococcus
Microbacteriaceae	Microbacterium
Microbacteriaceae	Microbacterium
Microbacteriaceae	Microbacterium
Microbacteriaceae	Microbacterium
Microbacteriaceae	Microbacterium
Microbacteriaceae	Microbacterium
Microbacteriaceae	Microbacterium
Microbacteriaceae	Microbacterium
Microbacteriaceae	Microbacterium
Micrococcaceae	Arthrobacter
Micrococcaceae	Arthrobacter
Micrococcaceae	Arthrobacter
Micrococcaceae	Arthrobacter
Micrococcaceae	Arthrobacter
Micrococcaceae	Arthrobacter

Micrococcaceae	Arthrobacter
Micrococcaceae	Arthrobacter
Micrococcaceae	Arthrobacter
Micrococcaceae	Arthrobacter
Bacillaceae	Bacillus
Bacillaceae	Bacillus
Bacillaceae	Bacillus
Bacillaceae	Bacillus
Unknown	Unknown
Bacillaceae	Bacillus
Bacillaceae	Bacillus
Bacillaceae	Bacillus
Unknown	Unknown
Bacillaceae	Bacillus
Bacillaceae	Bacillus
Bacillaceae	Bacillus
Bacillaceae	Bacillus
Bacillaceae	Bacillus
Bacillaceae	Bacillus
Bacillaceae	Bacillus
Bacillaceae	Bacillus
Moraxellaceae	Acinetobacter
Moraxellaceae	Acinetobacter
Moraxellaceae	Acinetobacter
Moraxellaceae	Acinetobacter
Pseudomonadaceae	Pseudomonas
Pseudomonadaceae	Pseudomonas
Unknown	Unknown
Pseudomonadaceae	Pseudomonas
Pseudomonadaceae	Pseudomonas
Burkholderiaceae	Ralstonia
Burkholderiaceae	Burkholderia
Burkholderiaceae	Burkholderia
Burkholderiaceae	Burkholderia
Burkholderiaceae	Burkholderia
Unknown	Unknown
Unknown	Unknown
Caulobacteraceae	Caulobacter
Caulobacteraceae	Caulobacter
Caulobacteraceae	Caulobacter
Caulobacteraceae	Caulobacter
Caulobacteraceae	Caulobacter
Caulobacteraceae	Caulobacter
Caulobacteraceae	Caulobacter
Caulobacteraceae	Caulobacter
Caulobacteraceae	Caulobacter

Caulobacteraceae	Caulobacter
Pectobacteriaceae	Pectobacterium
Pectobacteriaceae	Pectobacterium
Enterobacteriaceae	Cronobacter
Enterobacteriaceae	Cronobacter
Aeromonadaceae	Aeromonas
Aeromonadaceae	Aeromonas
Aeromonadaceae	Aeromonas
Aeromonadaceae	Aeromonas
Enterobacteriaceae	Salmonella
Yersiniaceae	Yersinia
Yersiniaceae	Yersinia
Yersiniaceae	Yersinia
Yersiniaceae	Yersinia
Aeromonadaceae	Aeromonas
Aeromonadaceae	Aeromonas
Vibrionaceae	Vibrio
Vibrionaceae	Vibrio
Vibrionaceae	Vibrio
Pectobacteriaceae	Dickeya
Enterobacteriaceae	Cronobacter
Pasteurellaceae	Pasteurella
Enterobacteriaceae	Klebsiella
Enterobacteriaceae	Klebsiella
Enterobacteriaceae	Klebsiella
Enterobacteriaceae	Enterobacter
Enterobacteriaceae	Salmonella
Enterobacteriaceae	Salmonella
Unknown	Unknown
Enterobacteriaceae	Salmonella
Enterobacteriaceae	Escherichia
Enterobacteriaceae	Unknown
Enterobacteriaceae	Escherichia
Pasteurellaceae	Haemophilus
Pasteurellaceae	Haemophilus
Pasteurellaceae	Pasteurella
Pasteurellaceae	Mannheimia
Pasteurellaceae	Mannheimia
Pasteurellaceae	Mannheimia
Bacillaceae	Bacillus
Bacillaceae	Bacillus
Bacillaceae	Bacillus
Aphanizomenonaceae	Nodularia
Aphanizomenonaceae	Nodularia
Aphanizomenonaceae	Nodularia
Acidithiobacillaceae	Acidithiobacillus

Unknown	Unknown
Synechococcaceae	Synechococcus
Erwiniaceae	Erwinia
Erwiniaceae	Erwinia
Enterobacteriaceae	Escherichia
Enterobacteriaceae	Escherichia
Unknown	Unknown
Unknown	Unknown
Rhizobiaceae	Rhizobium
Pseudomonadaceae	Pseudomonas
Unknown	Unknown
Xanthomonadaceae	Xanthomonas
Burkholderiaceae	Burkholderia
Synechococcaceae	Synechococcus
Synechococcaceae	Synechococcus
Unknown	Unknown
Rhodobacteraceae	Rhodobacter
Rhodobacteraceae	Rhodovulum
Synechococcaceae	Synechococcus
Synechococcaceae	Synechococcus
Synechococcaceae	Synechococcus
Synechococcaceae	Synechococcus
Synechococcaceae	Synechococcus
Synechococcaceae	Synechococcus
Synechococcaceae	Synechococcus
Alcaligenaceae	Achromobacter
Alcaligenaceae	Achromobacter
Alcaligenaceae	Achromobacter
Pseudomonadaceae	Pseudomonas
Pseudomonadaceae	Pseudomonas
Pseudomonadaceae	Pseudomonas
Pseudomonadaceae	Pseudomonas
Pseudomonadaceae	Pseudomonas
Pseudomonadaceae	Pseudomonas
Pseudomonadaceae	Pseudomonas
Synechococcaceae	Synechococcus
Synechococcaceae	Synechococcus
Synechococcaceae	Synechococcus
Synechococcaceae	Synechococcus
Synechococcaceae	Synechococcus
Synechococcaceae	Synechococcus
Synechococcaceae	Synechococcus
Staphylococcaceae	Staphylococcus
Staphylococcaceae	Staphylococcus
Staphylococcaceae	Staphylococcus
Staphylococcaceae	Staphylococcus

Staphylococcaceae	Staphylococcus
Unknown	Unknown
Staphylococcaceae	Staphylococcus
Staphylococcaceae	Staphylococcus
Staphylococcaceae	Staphylococcus
Staphylococcaceae	Staphylococcus
Staphylococcaceae	Staphylococcus
Staphylococcaceae	Staphylococcus
Staphylococcaceae	Staphylococcus
Staphylococcaceae	Staphylococcus
Staphylococcaceae	Staphylococcus
Staphylococcaceae	Staphylococcus
Streptomycetaceae	Streptomyces
Streptomycetaceae	Streptomyces
Streptomycetaceae	Streptomyces
Streptomycetaceae	Streptomyces
Streptomycetaceae	Streptomyces
Streptomycetaceae	Streptomyces
Streptomycetaceae	Streptomyces
Streptomycetaceae	Streptomyces
Streptomycetaceae	Streptomyces
Streptomycetaceae	Streptomyces
Streptomycetaceae	Streptomyces
Streptomycetaceae	Streptomyces
Streptomycetaceae	Streptomyces
Streptomycetaceae	Streptomyces
Streptomycetaceae	Streptomyces
Streptomycetaceae	Streptomyces
Streptomycetaceae	Streptomyces
Streptomycetaceae	Streptomyces
Bacillaceae	Bacillus
Bacillaceae	Bacillus
Bacillaceae	Bacillus
Bacillaceae	Bacillus
Bacillaceae	Bacillus
Bacillaceae	Bacillus
Bacillaceae	Bacillus
Bacillaceae	Bacillus
Bacillaceae	Bacillus
Bacillaceae	Bacillus
Bacillaceae	Bacillus
Bacillaceae	Bacillus
Bacillaceae	Bacillus
Listeriaceae	Listeria
Listeriaceae	Listeria
Listeriaceae	Listeria

Listeriaceae	Listeria
Unknown	Unknown
Listeriaceae	Listeria
Listeriaceae	Listeria
Listeriaceae	Listeria
Bacillaceae	Bacillus
Bacillaceae	Bacillus
Peptostreptococcaceae	Clostridioides
Clostridiaceae	Clostridium
Peptostreptococcaceae	Clostridioides
Clostridiaceae	Clostridium
Clostridiaceae	Clostridium
Clostridiaceae	Clostridium
Clostridiaceae	Clostridium
Peptostreptococcaceae	Clostridioides
Peptostreptococcaceae	Clostridioides
Clostridiaceae	Clostridium
Clostridiaceae	Clostridium
Clostridiaceae	Clostridium
Peptostreptococcaceae	Clostridioides
Clostridiaceae	Clostridium
Unknown	Unknown
Unknown	Unknown
Unknown	Unknown
Unknown	Unknown
Unknown	Unknown
Unknown	Unknown
Unknown	Unknown
Unknown	Unknown
Erwiniaceae	Pantoea
Enterobacteriaceae	Klebsiella
Enterobacteriaceae	Klebsiella
Enterobacteriaceae	Klebsiella
Enterobacteriaceae	Klebsiella
Enterobacteriaceae	Escherichia
Enterobacteriaceae	Cronobacter
Xanthomonadaceae	Stenotrophomonas
Aeromonadaceae	Aeromonas
Aeromonadaceae	Aeromonas
Aeromonadaceae	Aeromonas
Aeromonadaceae	Aeromonas
Aeromonadaceae	Aeromonas
Aeromonadaceae	Aeromonas
Aeromonadaceae	Aeromonas
Aeromonadaceae	Aeromonas
Enterobacteriaceae	Escherichia

Enterobacteriaceae	Escherichia
Enterobacteriaceae	Escherichia
Enterobacteriaceae	Escherichia
Enterobacteriaceae	Escherichia
Enterobacteriaceae	Shigella
Enterobacteriaceae	Escherichia
Enterobacteriaceae	Cronobacter
Moraxellaceae	Acinetobacter
Moraxellaceae	Acinetobacter
Moraxellaceae	Acinetobacter
Moraxellaceae	Acinetobacter
Moraxellaceae	Acinetobacter
Moraxellaceae	Acinetobacter
Moraxellaceae	Acinetobacter
Moraxellaceae	Acinetobacter
Moraxellaceae	Acinetobacter
Moraxellaceae	Acinetobacter
Moraxellaceae	Acinetobacter
Moraxellaceae	Acinetobacter
Moraxellaceae	Acinetobacter
Moraxellaceae	Acinetobacter
Moraxellaceae	Acinetobacter
Moraxellaceae	Acinetobacter
Moraxellaceae	Acinetobacter
Moraxellaceae	Acinetobacter
Unknown	Unknown
Moraxellaceae	Acinetobacter
Moraxellaceae	Acinetobacter
Rhizobiaceae	Sinorhizobium
Rhizobiaceae	Sinorhizobium
Rhizobiaceae	Rhizobium
Rhizobiaceae	Sinorhizobium
Acaryochloridaceae	Acaryochloris
Acaryochloridaceae	Acaryochloris
Rhodobacteraceae	Ruegeria
Rhodobacteraceae	Ruegeria
Rhizobiaceae	Rhizobium
Unknown	Unknown
Unknown	Unknown
Rhizobiaceae	Agrobacterium
Bacillaceae	Bacillus
Bacillaceae	Bacillus
Bacillaceae	Bacillus
Unknown	Unknown
Bacillaceae	Bacillus
Bacillaceae	Bacillus

Bacillaceae	Bacillus
Unknown	Unknown
Bacillaceae	Bacillus
Bacillaceae	Bacillus
Streptomycetaceae	Streptomyces
Streptomycetaceae	Streptomyces
Streptomycetaceae	Streptomyces
Streptomycetaceae	Streptomyces
Streptomycetaceae	Streptomyces
Streptomycetaceae	Streptomyces
Streptomycetaceae	Streptomyces
Streptomycetaceae	Streptomyces
Streptomycetaceae	Streptomyces
Streptomycetaceae	Streptomyces
Streptomycetaceae	Streptomyces
Streptomycetaceae	Streptomyces
Streptomycetaceae	Streptomyces
Streptomycetaceae	Streptomyces
Streptomycetaceae	Streptomyces
Streptomycetaceae	Streptomyces
Streptomycetaceae	Streptomyces
Streptomycetaceae	Streptomyces
Streptomycetaceae	Streptomyces
Streptomycetaceae	Streptomyces
Micrococcaceae	Arthrobacter
Micrococcaceae	Arthrobacter
Micrococcaceae	Arthrobacter
Micrococcaceae	Arthrobacter
Micrococcaceae	Arthrobacter
Micrococcaceae	Arthrobacter
Micrococcaceae	Arthrobacter
Micrococcaceae	Arthrobacter
Micrococcaceae	Arthrobacter
Micrococcaceae	Arthrobacter
Micrococcaceae	Arthrobacter
Micrococcaceae	Arthrobacter
Micrococcaceae	Arthrobacter
Micrococcaceae	Arthrobacter
Micrococcaceae	Arthrobacter
Micrococcaceae	Arthrobacter
Micrococcaceae	Arthrobacter
Pseudomonadaceae	Pseudomonas
Pseudomonadaceae	Pseudomonas
Pseudomonadaceae	Pseudomonas
Pseudomonadaceae	Pseudomonas
Pseudomonadaceae	Pseudomonas

Pseudomonadaceae	Pseudomonas
Pseudomonadaceae	Pseudomonas
Pseudomonadaceae	Pseudomonas
Unknown	Unknown
Unknown	Unknown
Burkholderiaceae	Burkholderia
Burkholderiaceae	Burkholderia
Unknown	Unknown
Alteromonadaceae	Unknown
Burkholderiaceae	Burkholderia
Burkholderiaceae	Ralstonia
Mycobacteriaceae	Mycolicibacterium
Mycobacteriaceae	Mycolicibacterium
Mycobacteriaceae	Mycolicibacterium
Mycobacteriaceae	Mycolicibacterium
Mycobacteriaceae	Mycolicibacterium
Mycobacteriaceae	Mycolicibacterium
Mycobacteriaceae	Mycolicibacterium
Mycobacteriaceae	Mycolicibacterium
Gordoniaceae	Gordonia
Gordoniaceae	Gordonia
Gordoniaceae	Gordonia
Tsukamurellaceae	Tsukamurella
Nocardiaceae	Rhodococcus
Gordoniaceae	Gordonia
Nocardiaceae	Rhodococcus
Nocardiaceae	Nocardia
Nocardiaceae	Rhodococcus
Nocardiaceae	Rhodococcus
Nocardiaceae	Rhodococcus
Nocardiaceae	Rhodococcus
Gordoniaceae	Gordonia
Corynebacteriaceae	Corynebacterium
Nocardiaceae	Rhodococcus
Nocardiaceae	Rhodococcus
Gordoniaceae	Gordonia
Gordoniaceae	Gordonia
Gordoniaceae	Gordonia
Gordoniaceae	Gordonia
Corynebacteriaceae	Corynebacterium
Corynebacteriaceae	Corynebacterium
Corynebacteriaceae	Corynebacterium
Corynebacteriaceae	Corynebacterium
Corynebacteriaceae	Corynebacterium
Corynebacteriaceae	Corynebacterium
Nocardiaceae	Rhodococcus

Gordoniaceae	Gordonia
Corynebacteriaceae	Corynebacterium
Corynebacteriaceae	Corynebacterium
Corynebacteriaceae	Corynebacterium
Nocardiaceae	Rhodococcus
Corynebacteriaceae	Corynebacterium
Vibrionaceae	Vibrio
Vibrionaceae	Vibrio
Vibrionaceae	Vibrio
Vibrionaceae	Vibrio
Vibrionaceae	Vibrio
Vibrionaceae	Vibrio
Vibrionaceae	Vibrio
Vibrionaceae	Vibrio
Streptococcaceae	Streptococcus
Streptococcaceae	Streptococcus
Streptococcaceae	Streptococcus
Streptococcaceae	Streptococcus
Streptococcaceae	Streptococcus
Streptococcaceae	Streptococcus
Streptococcaceae	Streptococcus
Micrococcaceae	Arthrobacter
Micrococcaceae	Arthrobacter
Micrococcaceae	Arthrobacter
Micrococcaceae	Arthrobacter
Micrococcaceae	Arthrobacter
Micrococcaceae	Arthrobacter
Lactobacillaceae	Lactobacillus
Lactobacillaceae	Lactobacillus
Lactobacillaceae	Lactobacillus
Lactobacillaceae	Lactobacillus
Lactobacillaceae	Lactobacillus
Lactobacillaceae	Lactobacillus
Lactobacillaceae	Lactobacillus
Lactobacillaceae	Lactobacillus
Lactobacillaceae	Lactobacillus
Lactobacillaceae	Lactobacillus
Leuconostocaceae	Leuconostoc
Lactobacillaceae	Lactobacillus
Lactobacillaceae	Lactobacillus
Lactobacillaceae	Lactobacillus
Lactobacillaceae	Lactobacillus
Lactobacillaceae	Lactobacillus
Lactobacillaceae	Lactobacillus
Lactobacillaceae	Lactobacillus
Unknown	Unknown

Lactobacillaceae	Lactobacillus
Lactobacillaceae	Lactobacillus
Lactobacillaceae	Lactobacillus
Lactobacillaceae	Lactobacillus
Lactobacillaceae	Lactobacillus
Lactobacillaceae	Lactobacillus
Staphylococcaceae	Staphylococcus
Staphylococcaceae	Staphylococcus
Staphylococcaceae	Staphylococcus
Unknown	Unknown
Staphylococcaceae	Staphylococcus
Staphylococcaceae	Staphylococcus
Staphylococcaceae	Staphylococcus
Staphylococcaceae	Staphylococcus
Staphylococcaceae	Staphylococcus
Staphylococcaceae	Staphylococcus
Staphylococcaceae	Staphylococcus
Staphylococcaceae	Staphylococcus
Staphylococcaceae	Staphylococcus
Staphylococcaceae	Staphylococcus
Gordoniaceae	Gordonia
Gordoniaceae	Gordonia
Gordoniaceae	Gordonia
Gordoniaceae	Gordonia
Gordoniaceae	Gordonia
Gordoniaceae	Gordonia
Gordoniaceae	Gordonia
Gordoniaceae	Gordonia
Gordoniaceae	Gordonia
Gordoniaceae	Gordonia
Staphylococcaceae	Staphylococcus
Staphylococcaceae	Staphylococcus
Staphylococcaceae	Staphylococcus
Staphylococcaceae	Staphylococcus
Staphylococcaceae	Staphylococcus
Staphylococcaceae	Staphylococcus
Staphylococcaceae	Staphylococcus
Unknown	Unknown
Unknown	Unknown
Staphylococcaceae	Staphylococcus
Unknown	Unknown
Unknown	Unknown
Staphylococcaceae	Staphylococcus
Staphylococcaceae	Staphylococcus
Staphylococcaceae	Staphylococcus
Staphylococcaceae	Staphylococcus

Unknown	Unknown
Unknown	Unknown
Unknown	Unknown
Staphylococcaceae	Staphylococcus
Staphylococcaceae	Staphylococcus
Staphylococcaceae	Staphylococcus
Staphylococcaceae	Staphylococcus
Staphylococcaceae	Staphylococcus
Microbacteriaceae	Microbacterium
Microbacteriaceae	Microbacterium
Microbacteriaceae	Microbacterium
Microbacteriaceae	Microbacterium
Microbacteriaceae	Microbacterium
Microbacteriaceae	Microbacterium
Micrococcaceae	Arthrobacter
Micrococcaceae	Arthrobacter
Micrococcaceae	Arthrobacter
Micrococcaceae	Arthrobacter
Micrococcaceae	Arthrobacter
Micrococcaceae	Arthrobacter
Micrococcaceae	Arthrobacter
Micrococcaceae	Arthrobacter
Flavobacteriaceae	Flavobacterium
Flavobacteriaceae	Flavobacterium
Clostridiaceae	Clostridium
Clostridiaceae	Clostridium
Clostridiaceae	Clostridium
Clostridiaceae	Clostridium
Clostridiaceae	Clostridium
Unknown	Unknown
Bacteroidaceae	Bacteroides
Flavobacteriaceae	Croceibacter
Bacillaceae	Bacillus
Thermaceae	Thermus
Thermaceae	Thermus
Thermaceae	Thermus
Eggerthellaceae	Eggerthella
Leuconostocaceae	Weissella
Leuconostocaceae	Leuconostoc
Leuconostocaceae	Weissella
Leuconostocaceae	Weissella
Pseudomonadaceae	Pseudomonas
Pseudomonadaceae	Pseudomonas
Vibrionaceae	Vibrio
Vibrionaceae	Vibrio
Vibrionaceae	Vibrio

Vibrionaceae	Vibrio
Vibrionaceae	Vibrio
Synechococcaceae	Synechococcus
Pseudomonadaceae	Pseudomonas
Unknown	Unknown
Enterobacteriaceae	Cronobacter
Unknown	Unknown
Streptomycetaceae	Streptomyces
Streptomycetaceae	Streptomyces
Streptomycetaceae	Streptomyces
Streptomycetaceae	Streptomyces
Streptomycetaceae	Streptomyces
Microbacteriaceae	Microbacterium
Microbacteriaceae	Microbacterium
Microbacteriaceae	Microbacterium
Microbacteriaceae	Microbacterium
Microbacteriaceae	Microbacterium
Microbacteriaceae	Microbacterium
Microbacteriaceae	Microbacterium
Microbacteriaceae	Microbacterium
Microbacteriaceae	Microbacterium
Microbacteriaceae	Microbacterium
Microbacteriaceae	Microbacterium
Microbacteriaceae	Microbacterium
Microbacteriaceae	Microbacterium
Streptomycetaceae	Streptomyces
Streptomycetaceae	Streptomyces
Streptomycetaceae	Streptomyces
Streptomycetaceae	Streptomyces
Streptomycetaceae	Streptomyces
Streptomycetaceae	Streptomyces
Streptomycetaceae	Streptomyces
Streptomycetaceae	Streptomyces
Streptomycetaceae	Streptomyces
Streptomycetaceae	Streptomyces
Streptomycetaceae	Streptomyces
Streptomycetaceae	Streptomyces
Streptomycetaceae	Streptomyces
Streptomycetaceae	Streptomyces
Streptomycetaceae	Streptomyces
Pasteurellaceae	Mannheimia
Unknown	Unknown
Alcaligenaceae	Bordetella
Pseudomonadaceae	Pseudomonas
Pseudomonadaceae	Pseudomonas
Brucellaceae	Brucella

Brucellaceae	Brucella
Brucellaceae	Brucella
Erwiniaceae	Pantoea
Enterobacteriaceae	Escherichia
Enterobacteriaceae	Escherichia
Rhizobiaceae	Agrobacterium
Rhizobiaceae	Agrobacterium
Pelagibacteraceae	Candidatus Pelagibacter
Rhodobacteraceae	Dinoroseobacter
Rhodobacteraceae	Sulfitobacter
Rhodobacteraceae	Epibacterium
Unknown	Unknown
Unknown	Nitratiruptor
Unknown	Unknown
Vibrionaceae	Vibrio
Vibrionaceae	Vibrio
Microcoleaceae	Planktothrix
Unknown	Unknown
Rhizobiaceae	Sinorhizobium
Brucellaceae	Ochrobactrum
Rhizobiaceae	Agrobacterium
Streptococcaceae	Lactococcus
Streptococcaceae	Lactococcus
Streptococcaceae	Lactococcus
Streptococcaceae	Lactococcus
Streptococcaceae	Lactococcus
Unknown	Unknown
Ruminococcaceae	Faecalibacterium
Ruminococcaceae	Faecalibacterium
Aeromonadaceae	Aeromonas
Aeromonadaceae	Aeromonas
Aeromonadaceae	Aeromonas
Vibrionaceae	Vibrio
Vibrionaceae	Vibrio
Vibrionaceae	Vibrio
Vibrionaceae	Vibrio
Unknown	Unknown
Unknown	Unknown
Unknown	Unknown
Ruminococcaceae	Faecalibacterium
Ruminococcaceae	Faecalibacterium
Flavobacteriaceae	Nonlabens
Oceanospirillaceae	Marinomonas
Alcaligenaceae	Achromobacter
Alcaligenaceae	Achromobacter
Alcaligenaceae	Achromobacter

Alcaligenaceae	Achromobacter
Pseudomonadaceae	Pseudomonas
Burkholderiaceae	Burkholderia
Burkholderiaceae	Burkholderia
Unknown	Unknown
Unknown	Unknown
Bacteroidaceae	Bacteroides
Unknown	Unknown
Comamonadaceae	Curvibacter
Caulobacteraceae	Caulobacter
Pelagibacteraceae	Candidatus Pelagibacter
Unknown	Unknown
Rhodobacteraceae	Rhodobacter
Rhodobacteraceae	Rhodobacter
Vibrionaceae	Vibrio
Vibrionaceae	Vibrio
Vibrionaceae	Vibrio
Vibrionaceae	Vibrio
Pseudoalteromonadaceae	Pseudoalteromonas
Vibrionaceae	Vibrio
Vibrionaceae	Vibrio
Vibrionaceae	Salinivibrio
Vibrionaceae	Vibrio
Alteromonadaceae	Alteromonas
Microcystaceae	Microcystis
Vibrionaceae	Vibrio
Shewanellaceae	Shewanella
Shewanellaceae	Shewanella
Microcystaceae	Microcystis
Flavobacteriaceae	Nonlabens
Unknown	Unknown
Streptococcaceae	Streptococcus
Streptococcaceae	Streptococcus
Streptococcaceae	Streptococcus
Streptococcaceae	Streptococcus
Unknown	Unknown
Unknown	Unknown
Streptococcaceae	Streptococcus
Streptococcaceae	Streptococcus
Streptococcaceae	Streptococcus
Streptococcaceae	Streptococcus
Streptococcaceae	Streptococcus
Streptococcaceae	Streptococcus
Streptococcaceae	Streptococcus
Staphylococcaceae	Staphylococcus
Streptococcaceae	Streptococcus

Streptococcaceae	Streptococcus
Streptococcaceae	Streptococcus
Streptococcaceae	Streptococcus
Streptococcaceae	Streptococcus
Streptococcaceae	Lactococcus
Streptococcaceae	Streptococcus
Micrococcaceae	Arthrobacter
Micrococcaceae	Arthrobacter
Micrococcaceae	Arthrobacter
Micrococcaceae	Arthrobacter
Micrococcaceae	Arthrobacter
Streptococcaceae	Streptococcus
Enterococcaceae	Enterococcus
Enterococcaceae	Enterococcus
Enterococcaceae	Enterococcus
Bacillaceae	Bacillus
Bacillaceae	Bacillus
Bacillaceae	Bacillus
Bacillaceae	Bacillus
Unknown	Unknown
Bacillaceae	Bacillus
Bacillaceae	Geobacillus
Bacillaceae	Bacillus
Bacillaceae	Bacillus
Listeriaceae	Listeria
Listeriaceae	Listeria
Listeriaceae	Listeria
Listeriaceae	Listeria
Listeriaceae	Listeria
Unknown	Unknown
Unknown	Unknown
Unknown	Unknown
Unknown	Unknown
Alcaligenaceae	Achromobacter
Pseudomonadaceae	Pseudomonas
Pseudomonadaceae	Pseudomonas
Pseudomonadaceae	Pseudomonas
Pseudomonadaceae	Pseudomonas
Vibrionaceae	Vibrio
Vibrionaceae	Vibrio
Vibrionaceae	Vibrio
Vibrionaceae	Vibrio
Pseudomonadaceae	Pseudomonas
Erwiniaceae	Erwinia
Erwiniaceae	Erwinia
Thermaceae	Thermus

Thermaceae	Thermus
Thermaceae	Thermus
Thermaceae	Thermus
Pseudomonadaceae	Pseudomonas
Pseudomonadaceae	Pseudomonas
Pseudomonadaceae	Pseudomonas
Streptomycetaceae	Streptomyces
Streptomycetaceae	Streptomyces
Streptomycetaceae	Streptomyces
Streptomycetaceae	Streptomyces
Streptomycetaceae	Streptomyces
Streptomycetaceae	Streptomyces
Streptomycetaceae	Streptomyces
Streptomycetaceae	Streptomyces
Streptomycetaceae	Streptomyces
Streptomycetaceae	Streptomyces
Streptomycetaceae	Streptomyces
Streptomycetaceae	Streptomyces
Vibrionaceae	Vibrio
Vibrionaceae	Vibrio
Vibrionaceae	Vibrio
Vibrionaceae	Vibrio
Vibrionaceae	Vibrio
Vibrionaceae	Vibrio
Vibrionaceae	Enterovibrio
Vibrionaceae	Enterovibrio
Shewanellaceae	Shewanella
Aeromonadaceae	Aeromonas
Aeromonadaceae	Aeromonas
Erwiniaceae	Erwinia
Aeromonadaceae	Aeromonas
Unknown	Unknown
Unknown	Unknown
Unknown	Unknown
Unknown	Unknown
Unknown	Unknown
Unknown	Unknown
Unknown	Unknown
Unknown	Unknown
Unknown	Unknown
Unknown	Unknown
Unknown	Unknown
Unknown	Unknown
Unknown	Unknown
Unknown	Unknown
Unknown	Unknown

Unknown	Unknown
Listeriaceae	Listeria
Listeriaceae	Listeria
Listeriaceae	Listeria
Bacillaceae	Bacillus
Unknown	Unknown
Bacillaceae	Bacillus
Bacillaceae	Geobacillus
Bacillaceae	Bacillus
Bacillaceae	Bacillus
Bacillaceae	Bacillus
Listeriaceae	Listeria
Listeriaceae	Listeria
Bacillaceae	Bacillus
Bacillaceae	Bacillus
Bacillaceae	Bacillus
Unknown	Unknown
Thermaceae	Thermus
Flavobacteriaceae	Flavobacterium
Vibrionaceae	Vibrio
Vibrionaceae	Vibrio
Vibrionaceae	Vibrio
Flavobacteriaceae	Cellulophaga
Flavobacteriaceae	Cellulophaga
Flavobacteriaceae	Cellulophaga
Gordoniaceae	Gordonia
Gordoniaceae	Gordonia
Gordoniaceae	Gordonia
Gordoniaceae	Gordonia
Rhodobacteraceae	Sulfitobacter
Rhodobacteraceae	Ruegeria
Rhodobacteraceae	Ruegeria
Rhodobacteraceae	Roseobacter
Rhodobacteraceae	Ruegeria
Rhodobacteraceae	Paracoccus
Rhodobacteraceae	Paracoccus
Rhizobiaceae	Sinorhizobium
Rhizobiaceae	Rhizobium
Streptomycetaceae	Streptomyces
Streptomycetaceae	Streptomyces
Streptomycetaceae	Streptomyces
Streptomycetaceae	Streptomyces
Streptomycetaceae	Streptomyces
Streptomycetaceae	Streptomyces
Streptomycetaceae	Streptomyces
Streptomycetaceae	Streptomyces

Streptomycetaceae	Streptomyces
Streptomycetaceae	Streptomyces
Streptomycetaceae	Streptomyces
Xanthomonadaceae	Stenotrophomonas
Pseudomonadaceae	Pseudomonas
Pseudomonadaceae	Pseudomonas
Micrococcaceae	Arthrobacter
Micrococcaceae	Arthrobacter
Micrococcaceae	Arthrobacter
Streptococcaceae	Streptococcus
Streptococcaceae	Streptococcus
Paenibacillaceae	Brevibacillus
Paenibacillaceae	Brevibacillus
Paenibacillaceae	Brevibacillus
Clostridiaceae	Clostridium
Flavobacteriaceae	Flavobacterium
Ruminococcaceae	Faecalibacterium
Bacillaceae	Bacillus
Bacillaceae	Bacillus
Unknown	Unknown
Unknown	Unknown
Unknown	Unknown
Bacillaceae	Exiguobacterium
Unknown	Unknown
Unknown	Unknown
Listeriaceae	Listeria
Unknown	Unknown
Bacillaceae	Bacillus
Flavobacteriaceae	Cellulophaga
Bacillaceae	Anoxybacillus
Paenibacillaceae	Paenibacillus
Paenibacillaceae	Paenibacillus
Paenibacillaceae	Paenibacillus
Vibrionaceae	Vibrio
Vibrionaceae	Vibrio
Vibrionaceae	Vibrio
Bacillaceae	Exiguobacterium
Bacillaceae	Exiguobacterium
Bacillaceae	Bacillus
Bacillaceae	Bacillus
Bacillaceae	Bacillus
Unknown	Unknown
Bacillaceae	Geobacillus
Bacillaceae	Caldibacillus
Bacillaceae	Bacillus
Bacillaceae	Bacillus

Bacillaceae	Bacillus
Bacillaceae	Bacillus
Bacillaceae	Bacillus
Bacillaceae	Bacillus
Bacillaceae	Bacillus
Unknown	Unknown
Bacillaceae	Bacillus
Bacillaceae	Bacillus
Bacillaceae	Bacillus
Bacillaceae	Bacillus
Clostridiaceae	Clostridium
Peptostreptococcaceae	Clostridioides
Unknown	Unknown
Peptostreptococcaceae	Clostridioides
Clostridiaceae	Clostridium
Clostridiaceae	Clostridium
Clostridiaceae	Clostridium
Clostridiaceae	Clostridium
Clostridiaceae	Clostridium
Clostridiaceae	Clostridium
Clostridiaceae	Clostridium
Clostridiaceae	Clostridium
Vibrionaceae	Vibrio
Vibrionaceae	Vibrio
Vibrionaceae	Vibrio
Vibrionaceae	Vibrio
Vibrionaceae	Vibrio
Shewanellaceae	Shewanella
Flavobacteriaceae	Riemerella
Halomonadaceae	Halomonas
Unknown	Unknown
Moraxellaceae	Acinetobacter
Moraxellaceae	Acinetobacter
Moraxellaceae	Acinetobacter
Moraxellaceae	Acinetobacter
Microbacteriaceae	Microbacterium
Microbacteriaceae	Microbacterium
Microbacteriaceae	Microbacterium
Microbacteriaceae	Microbacterium
Microbacteriaceae	Microbacterium
Microbacteriaceae	Microbacterium
Microbacteriaceae	Microbacterium
Microbacteriaceae	Microbacterium
Microbacteriaceae	Microbacterium
Microbacteriaceae	Microbacterium
Streptomycetaceae	Streptomyces

Nocardiaceae	Rhodococcus
Nocardiaceae	Rhodococcus
Nocardiaceae	Rhodococcus
Nocardiaceae	Rhodococcus
Synechococcaceae	Synechococcus
Synechococcaceae	Synechococcus
Synechococcaceae	Synechococcus
Synechococcaceae	Synechococcus
Prochloraceae	Prochlorococcus
Synechococcaceae	Synechococcus
Synechococcaceae	Synechococcus
Pseudoalteromonadaceae	Pseudoalteromonas
Vibrionaceae	Vibrio
Vibrionaceae	Vibrio
Vibrionaceae	Vibrio
Streptococcaceae	Streptococcus
Lactobacillaceae	Lactobacillus
Microbacteriaceae	Microbacterium
Microbacteriaceae	Microbacterium
Microbacteriaceae	Microbacterium
Microbacteriaceae	Microbacterium
Microbacteriaceae	Microbacterium
Microbacteriaceae	Microbacterium
Microbacteriaceae	Microbacterium
Streptomycetaceae	Streptomyces
Streptomycetaceae	Streptomyces
Streptococcaceae	Streptococcus
Streptococcaceae	Streptococcus
Streptococcaceae	Streptococcus
Ruminococcaceae	Faecalibacterium
Unknown	Unknown
Unknown	Unknown
Unknown	Unknown
Unknown	Unknown
Unknown	Unknown
Micrococcaceae	Arthrobacter
Propionibacteriaceae	Propionibacterium
Propionibacteriaceae	Propionibacterium
Propionibacteriaceae	Propionibacterium
Corynebacteriaceae	Corynebacterium
Pasteurellaceae	Pasteurella
Nocardiaceae	Rhodococcus
Gordoniaceae	Gordonia
Gordoniaceae	Gordonia
Streptococcaceae	Streptococcus
Erysipelotrichaceae	Erysipelothrix

Lactobacillaceae	Lactobacillus
Lactobacillaceae	Lactobacillus
Lactobacillaceae	Lactobacillus
Propionibacteriaceae	Propionibacterium
Propionibacteriaceae	Propionibacterium
Propionibacteriaceae	Propionibacterium
Microbacteriaceae	Microbacterium
Microbacteriaceae	Microbacterium
Thermoanaerobacterales Family III. Incertae Sedis	Thermoanaerobacterium
Streptococcaceae	Streptococcus
Bacillaceae	Bacillus
Clostridiaceae	Clostridium
Clostridiaceae	Clostridium
Lactobacillaceae	Lactobacillus
Lactobacillaceae	Lactobacillus
Lactobacillaceae	Lactobacillus
Lactobacillaceae	Lactobacillus
Micrococcaceae	Arthrobacter
Enterococcaceae	Enterococcus
Lactobacillaceae	Lactobacillus
Ruminococcaceae	Faecalibacterium
Clostridiaceae	Clostridium
Paenibacillaceae	Paenibacillus
Bacillaceae	Geobacillus
Listeriaceae	Listeria
Unknown	Unknown
Unknown	Unknown
Microbacteriaceae	Microbacterium
Microbacteriaceae	Microbacterium
Microbacteriaceae	Microbacterium
Microbacteriaceae	Microbacterium
Microbacteriaceae	Microbacterium
Microbacteriaceae	Microbacterium
Microbacteriaceae	Microbacterium
Micrococcaceae	Arthrobacter
Micrococcaceae	Arthrobacter
Micrococcaceae	Arthrobacter
Micrococcaceae	Arthrobacter
Microcystaceae	Microcystis
Bacillaceae	Bacillus
Bacillaceae	Bacillus
Unknown	Unknown
Microcoleaceae	Planktothrix
Microbacteriaceae	Pontimonas
Nocardiaceae	Rhodococcus
Micrococcaceae	Paeniglutamicibacter

Micrococcaceae	Paeniglutamicibacter
Micrococcaceae	Arthrobacter
Micrococcaceae	Arthrobacter
Micrococcaceae	Arthrobacter
Micrococcaceae	Sinomonas
Micrococcaceae	Arthrobacter
Micrococcaceae	Arthrobacter
Micrococcaceae	Arthrobacter
Micrococcaceae	Arthrobacter
Micrococcaceae	Arthrobacter
Microbacteriaceae	Microbacterium
Microbacteriaceae	Microbacterium
Brevibacteriaceae	Brevibacterium
Xanthomonadaceae	Xanthomonas
Thermaceae	Thermus
Thermaceae	Meiothermus
Thermaceae	Thermus
Lactobacillaceae	Lactobacillus
Lactobacillaceae	Lactobacillus
Lactobacillaceae	Lactobacillus
Lactobacillaceae	Lactobacillus
Leuconostocaceae	Weissella
Lactobacillaceae	Lactobacillus
Rhizobiaceae	Rhizobium
Rhizobiaceae	Rhizobium
Erwiniaceae	Erwinia
Enterobacteriaceae	Cronobacter
Erwiniaceae	Erwinia
Erwiniaceae	Erwinia
Erwiniaceae	Erwinia
Erwiniaceae	Erwinia
Vibrionaceae	Vibrio
Erwiniaceae	Erwinia
Yersiniaceae	Serratia
Yersiniaceae	Serratia
Microbacteriaceae	Microbacterium
Nocardiopsaceae	Thermobifida
Nocardiopsaceae	Thermobifida
Bacillaceae	Bacillus
Bacillaceae	Bacillus
Bacillaceae	Bacillus
Bacillaceae	Exiguobacterium
Rhodobacteraceae	Pelagibaca
Sphingomonadaceae	Sphingobium
Rhodobacteraceae	Ruegeria
Rhodobacteraceae	Ruegeria

Rhodobacteraceae	Paracoccus
Rhodobacteraceae	Roseobacter
Rhodobacteraceae	Roseobacter
Vibrionaceae	Vibrio
Shewanellaceae	Shewanella
Vibrionaceae	Vibrio
Vibrionaceae	Vibrio
Alteromonadaceae	Alteromonas
Vibrionaceae	Vibrio
Pseudomonadaceae	Pseudomonas
Flavobacteriaceae	Flavobacterium
Pseudomonadaceae	Pseudomonas
Unknown	Unknown
Micrococcaceae	Arthrobacter
Micrococcaceae	Arthrobacter
Micrococcaceae	Arthrobacter
Micrococcaceae	Arthrobacter
Bacillaceae	Bacillus
Bacillaceae	Bacillus
Unknown	Unknown
Paenibacillaceae	Brevibacillus
Micrococcaceae	Arthrobacter
Unknown	Unknown
Unknown	Unknown
Synechococcaceae	Synechococcus
Synechococcaceae	Synechococcus
Unknown	Unknown
Streptomycetaceae	Streptomyces
Streptomycetaceae	Streptomyces
Streptomycetaceae	Streptomyces
Unknown	Unknown
Unknown	Unknown
Unknown	Unknown
Unknown	Unknown
Unknown	Unknown
Synechococcaceae	Synechococcus
Synechococcaceae	Synechococcus
Streptococcaceae	Lactococcus
Unknown	Unknown
Flavobacteriaceae	Croceibacter
Prochloraceae	Prochlorococcus
Synechococcaceae	Synechococcus
Synechococcaceae	Synechococcus
Paenibacillaceae	Paenibacillus
Brevibacteriaceae	Brevibacterium
Peptostreptococcaceae	Clostridioides

Streptomycetaceae	Streptomyces
Rhodobacteraceae	Dinoroseobacter
Unknown	Unknown
Pseudomonadaceae	Pseudomonas
Pseudomonadaceae	Pseudomonas
Microbacteriaceae	Clavibacter
Xanthomonadaceae	Xylella
Pseudomonadaceae	Pseudomonas
Pseudomonadaceae	Pseudomonas
Actinomycetaceae	Actinomyces
Bifidobacteriaceae	Bifidobacterium
Yersiniaceae	Yersinia
Thermaceae	Thermus
Thermaceae	Thermus
Lactobacillaceae	Lactobacillus
Staphylococcaceae	Staphylococcus
Burkholderiaceae	Ralstonia
Unknown	Unknown
Unknown	Unknown
Unknown	Unknown
Xanthomonadaceae	Xanthomonas
Intrasporangiaceae	Tetrasphaera
Sphingomonadaceae	Citromicrobium
Aerococcaceae	Aerococcus
Bacillaceae	Bacillus
Unknown	Unknown
Ruminococcaceae	Faecalibacterium
Unknown	Unknown
Unknown	Unknown
Unknown	Unknown
Ruminococcaceae	Faecalibacterium
Unknown	Unknown
Unknown	Unknown
Bacillaceae	Bacillus
Unknown	Unknown
Microbacteriaceae	Rathayibacter
Micrococcaceae	Arthrobacter
Enterococcaceae	Enterococcus
Streptococcaceae	Streptococcus
Enterococcaceae	Enterococcus

Host species
Mycolicibacterium smegmatis
Mycolicibacterium smegmatis
Mycolicibacterium smegmatis
Mycolicibacterium smegmatis
Mycolicibacterium smegmatis
Mycolicibacterium smegmatis
Mycolicibacterium smegmatis
Mycolicibacterium smegmatis
Mycolicibacterium smegmatis
Mycolicibacterium smegmatis
Mycolicibacterium smegmatis
Mycolicibacterium smegmatis
Mycolicibacterium smegmatis
Mycolicibacterium smegmatis
Mycolicibacterium smegmatis
Mycolicibacterium smegmatis
Mycolicibacterium smegmatis
Mycolicibacterium smegmatis
Mycolicibacterium smegmatis
Mycolicibacterium smegmatis
Mycolicibacterium smegmatis
Mycolicibacterium smegmatis
Mycolicibacterium smegmatis
Mycolicibacterium smegmatis
Mycolicibacterium smegmatis
Mycolicibacterium smegmatis
Unknown
Mycolicibacterium smegmatis
Gordonia terrae
Gordonia terrae
Gordonia terrae
Gordonia terrae
Gordonia terrae
Gordonia terrae
Unknown
Gordonia terrae
Gordonia terrae
Gordonia terrae
Gordonia terrae
Gordonia westfalica
Gordonia terrae
Tsukamurella paurometabola
Gordonia terrae
Gordonia terrae
Gordonia terrae

Rhodococcus erythropolis
Unknown
Mycolicibacterium smegmatis
Gordonia terrae
Gordonia terrae
Gordonia alkanivorans
Gordonia terrae
Gordonia terrae
Gordonia terrae
Gordonia rubripertincta
Gordonia terrae
Gordonia terrae
Gordonia terrae
Gordonia terrae
Gordonia terrae
Gordonia terrae
Gordonia terrae
Gordonia terrae
Gordonia terrae
Mycolicibacterium smegmatis
Mycolicibacterium smegmatis
Unknown
Mycolicibacterium smegmatis
Mycolicibacterium smegmatis
Mycolicibacterium smegmatis
Mycolicibacterium smegmatis
Mycolicibacterium smegmatis
Mycolicibacterium smegmatis
Mycolicibacterium smegmatis
Mycolicibacterium smegmatis
Mycolicibacterium smegmatis
Gordonia terrae
Gordonia rubripertincta
Gordonia terrae
Gordonia terrae
Mycolicibacterium smegmatis
Mycolicibacterium smegmatis
Mycolicibacterium smegmatis
Mycolicibacterium smegmatis
Mycolicibacterium smegmatis
Mycolicibacterium smegmatis
Mycolicibacterium smegmatis
Mycolicibacterium smegmatis
Mycolicibacterium smegmatis
Unknown
Mycolicibacterium smegmatis

Mycolicibacterium smegmatis
Mycobacterium tuberculosis
Unknown
Mycolicibacterium smegmatis
Mycolicibacterium smegmatis
Mycolicibacterium smegmatis
Mycolicibacterium smegmatis
Mycolicibacterium smegmatis
Mycolicibacterium smegmatis
Mycolicibacterium smegmatis
Mycolicibacterium smegmatis
Mycolicibacterium smegmatis
Mycolicibacterium smegmatis
Mycolicibacterium smegmatis
Mycolicibacterium smegmatis
Mycolicibacterium smegmatis
Mycolicibacterium smegmatis
Mycolicibacterium smegmatis
Mycolicibacterium smegmatis
Mycolicibacterium smegmatis
Arthrobacter sp. ATCC 21022
Arthrobacter globiformis
Arthrobacter globiformis
Arthrobacter globiformis
Arthrobacter globiformis
Arthrobacter globiformis
Arthrobacter globiformis
Gordonia malaquae
Rhodococcus erythropolis
Gordonia terrae
Gordonia terrae
Gordonia neofelifaecis
Unknown
Rhodococcus hoagii
Rhodococcus hoagii
Mycolicibacterium smegmatis
Mycolicibacterium smegmatis
Mycolicibacterium smegmatis
Mycolicibacterium smegmatis
Mycolicibacterium smegmatis
Mycolicibacterium smegmatis
Unknown
Unknown
Gordonia sp.
Gordonia sp.
Gordonia malaquae

Gordonia terrae
Gordonia terrae
Gordonia terrae
Gordonia terrae
Nocardia asteroides
Tsukamurella inchonensis
Tsukamurella inchonensis
Gordonia terrae
Gordonia terrae
Gordonia terrae
Gordonia terrae
Gordonia terrae
Gordonia terrae
Gordonia terrae
Gordonia terrae
Gordonia terrae
Gordonia terrae
Gordonia rubripertincta
Gordonia terrae
Gordonia terrae
Acidovorax citrulli
Pseudomonas aeruginosa
Achromobacter animicus
Unknown
Vibrio coralliilyticus
Caulobacter vibrioides
Caulobacter vibrioides
Erythrobacter litoralis
Unknown
Nitrincola sp.
Pseudomonas sp.
Sinorhizobium meliloti
Sinorhizobium sp. LM21
Sinorhizobium sp. LM21
Pseudomonas fluorescens
Aeromonas hydrophila
Synechococcus sp. WH 7803
Synechococcus sp.
Unknown
Synechococcus sp.
Synechococcus sp.
Synechococcus sp.
Xanthomonas euvesicatoria
Xanthomonas oryzae
Xanthomonas oryzae
Unknown

Xanthomonas oryzae
Xanthomonas oryzae
Unknown
Pantoea sp.
Salmonella enterica
Salmonella enterica
Proteus mirabilis
Raoultella ornithinolytica
Cronobacter sakazakii
Escherichia coli
Pseudomonas fluorescens
Pseudomonas sp.
Pseudomonas fluorescens
Unknown
Pseudomonas syringae
Unknown
Unknown
Microcystis aeruginosa
Ochrobactrum anthropi
Sphingomonas paucimobilis
Unknown
Unknown
Unknown
Flavobacterium sp.
Achromobacter xylosoxidans
Enterobacter sp. CT7
Escherichia coli
Escherichia coli
Escherichia coli
Salmonella enterica
Salmonella enterica
Cronobacter sakazakii
Escherichia coli
Klebsiella pneumoniae
Klebsiella pneumoniae
Klebsiella pneumoniae
Klebsiella pneumoniae
Klebsiella pneumoniae
Klebsiella pneumoniae
Escherichia coli
Aeromonas salmonicida
Salmonella enterica
Salmonella enterica
Pantoea sp.
Unknown
Escherichia coli

Escherichia coli
Escherichia coli
Escherichia coli
Pseudomonas syringae
Serratia marcescens
Burkholderia ambifaria
Xanthomonas campestris
Xanthomonas campestris
Pseudomonas aeruginosa
Pseudomonas aeruginosa
Pseudomonas aeruginosa
Pseudomonas aeruginosa
Pseudomonas aeruginosa
Pseudomonas aeruginosa
Pseudomonas aeruginosa
Pseudomonas aeruginosa
Pseudomonas aeruginosa
Unknown
Unknown
Unknown
Unknown
Pseudomonas aeruginosa
Pseudomonas aeruginosa
Pseudomonas aeruginosa
Mannheimia haemolytica
Aggregatibacter actinomycetemcomitans
Aggregatibacter actinomycetemcomitans
Xanthomonas citri
Delftia acidovorans
Unknown
Cronobacter sakazakii
Klebsiella pneumoniae
Klebsiella pneumoniae
Pseudomonas fluorescens
Gordonia terrae
Gordonia terrae
Gordonia terrae
Gordonia terrae
Gordonia terrae
Gordonia terrae
Gordonia terrae
Gordonia terrae
Gordonia terrae
Gordonia terrae
Gordonia terrae
Gordonia terrae

Gordonia terrae
Gordonia terrae
Gordonia terrae
Gordonia terrae
Gordonia rubripertincta
Gordonia terrae
Mycolicibacterium smegmatis
Unknown
Mycolicibacterium smegmatis
Mycolicibacterium smegmatis
Corynebacterium vitaeruminis
Corynebacterium vitaeruminis
Corynebacterium vitaeruminis
Corynebacterium vitaeruminis
Corynebacterium vitaeruminis
Corynebacterium vitaeruminis
Corynebacterium glutamicum
Corynebacterium xerosis
Corynebacterium xerosis
Corynebacterium glutamicum
Corynebacterium glutamicum
Gordonia terrae
Gordonia terrae
Gordonia terrae
Gordonia terrae
Rhodococcus erythropolis
Gordonia terrae
Gordonia terrae
Gordonia terrae
Gordonia terrae
Gordonia terrae
Gordonia terrae
Mycolicibacterium smegmatis
Mycolicibacterium smegmatis
Listeria monocytogenes
Unknown
Listeria monocytogenes
Listeria monocytogenes
Listeria monocytogenes
Listeria monocytogenes
Listeria monocytogenes
Listeria monocytogenes
Listeria monocytogenes
Listeria monocytogenes
Lactobacillus casei
Lactobacillus casei

Lactobacillus paracasei
Lactobacillus paracasei
Lactobacillus paracasei
Lactobacillus paracasei
Lactobacillus paracasei
Lactococcus lactis
Lactococcus lactis
Lactococcus lactis
Lactococcus lactis
Lactococcus lactis
Lactococcus lactis
Lactococcus lactis
Lactococcus lactis
Lactococcus lactis
Enterococcus faecalis
Enterococcus faecalis
Enterococcus faecalis
Enterococcus faecalis
Enterococcus faecalis
Enterococcus faecalis
Enterococcus faecalis
Enterococcus faecalis
Enterococcus faecalis
Enterococcus faecalis
Unknown
Unknown
Enterococcus faecalis
Enterococcus faecalis
Enterococcus faecalis
Enterococcus faecalis
Enterococcus faecalis
Enterococcus faecalis
Enterococcus faecalis
Enterococcus faecium
Enterococcus faecalis
Enterococcus faecalis
Enterococcus faecalis
Enterococcus faecalis
Enterococcus faecalis
Enterococcus faecalis
Enterococcus faecalis
Enterococcus faecalis
Enterococcus faecalis
Streptococcus pneumoniae
Enterococcus faecalis
Enterococcus faecalis

Enterococcus faecalis
Enterococcus faecalis
Enterococcus faecalis
Enterococcus faecalis
Enterococcus faecalis
Aeromonas salmonicida
Aeromonas sp.
Aeromonas salmonicida
Aeromonas salmonicida
Aeromonas hydrophila
Aeromonas salmonicida
Aeromonas hydrophila
Aeromonas salmonicida
Aeromonas salmonicida
Aeromonas salmonicida
Aeromonas salmonicida
Aeromonas salmonicida
Aeromonas salmonicida
Aeromonas hydrophila
Aeromonas hydrophila
Aeromonas salmonicida
Aeromonas salmonicida
Vibrio pelagius
Vibrio sp. YD38
Vibrio alginolyticus
Vibrio sagamiensis
Vibrio cyclitrophicus
Vibrio cyclitrophicus
Vibrio cyclitrophicus
Vibrio lentus
Vibrio sp. 12G05
Vibrio sp. F12
Escherichia coli
Pseudomonas fluorescens
Pseudomonas tolaasii
Pseudomonas sp.
Pseudomonas putida
Pseudomonas fluorescens
Candidatus Pelagibacter sp. FZCC0015
Candidatus Pelagibacter ubique
Candidatus Pelagibacter ubique
Pseudoalteromonas sp. Bq0
Marinobacter sp.
Pseudoalteromonas marina
Pseudoalteromonas marina
Candidatus Pelagibacter sp. HTCC7211

Candidatus Pelagibacter sp. FZCC0015
Unknown
Pantoea agglomerans
Colwellia psychrerythraea
Unknown
Candidatus Puniceispirillum marinum
Unknown
Xanthomonas arboricola
Aeromonas hydrophila
Aeromonas hydrophila
Aeromonas hydrophila
Vibrio cholerae
Enterovibrio norvegicus
Gordonia terrae
Gordonia terrae
Gordonia terrae
Gordonia terrae
Gordonia terrae
Gordonia terrae
Gordonia terrae
Gordonia terrae
Gordonia terrae
Gordonia sputi
Gordonia terrae
Gordonia terrae
Gordonia terrae
Gordonia terrae
Gordonia terrae
Gordonia terrae
Gordonia terrae
Gordonia terrae
Gordonia terrae
Gordonia terrae
Gordonia terrae
Gordonia terrae
Gordonia terrae
Gordonia terrae
Unknown
Unknown
Bacillus cereus
Bacillus thuringiensis
Bacillus cereus
Bacillus cereus
Bacillus cereus
Bacillus cereus
Bacillus cereus

Bacillus cereus
Streptococcus equi
Streptococcus pyogenes
Streptococcus pyogenes
Streptococcus pyogenes
Streptococcus pyogenes
Streptococcus pyogenes
Streptococcus pyogenes
Streptococcus pyogenes
Streptococcus dysgalactiae
Streptococcus dysgalactiae
Streptococcus dysgalactiae
Unknown
Streptococcus suis
Streptococcus suis
Streptococcus suis
Unknown
Streptomyces griseofuscus
Streptomyces griseus
Streptomyces lividans
Streptomyces griseus
Streptomyces griseus
Streptomyces griseus
Streptomyces griseus
Streptomyces griseus
Streptomyces griseus
Streptomyces lividans
Streptomyces griseus
Streptomyces griseus
Streptomyces platensis
Streptomyces lividans
Streptomyces lividans
Streptomyces griseus
Streptomyces griseus
Streptomyces griseus
Streptomyces griseus
Streptomyces griseus
Streptomyces griseofuscus
Streptomyces viridochromogenes
Streptomyces griseus
Streptomyces griseus
Streptomyces griseus
Streptomyces griseus
Streptomyces scabiei
Streptomyces scabiei
Streptomyces toxytricini

Streptomyces xanthochromogenes
Streptomyces xanthochromogenes
Streptomyces xanthochromogenes
Streptomyces xanthochromogenes
Streptomyces xanthochromogenes
Streptomyces venezuelae
Streptomyces venezuelae
Streptomyces venezuelae
Streptomyces venezuelae
Streptomyces venezuelae
Streptomyces griseofuscus
Streptomyces lividans
Streptomyces sp. ZL12
Streptomyces lividans
Streptomyces venezuelae
Streptomyces toxytricini
Streptomyces griseus
Streptomyces griseus
Streptomyces griseus
Streptomyces griseus
Streptomyces avermitilis
Streptomyces mirabilis
Streptomyces flavovirens
Streptomyces griseus
Streptomyces griseus
Enterococcus faecium
Enterococcus faecalis
Enterococcus faecalis
Enterococcus faecalis
Enterococcus faecalis
Enterococcus faecalis
Unknown
Enterococcus faecalis
Enterococcus faecalis
Enterococcus faecalis
Enterococcus faecalis
Enterococcus faecalis
Enterococcus faecium
Enterococcus faecium
Enterococcus faecalis
Enterococcus faecalis
Unknown
Enterococcus faecalis
Unknown
Enterococcus faecalis
Enterococcus faecalis

Enterococcus faecalis
Unknown
Lactococcus lactis
Leuconostoc mesenteroides
Leuconostoc mesenteroides
Leuconostoc mesenteroides
Leuconostoc mesenteroides
Streptococcus henryi
Streptococcus iniae
Lactobacillus pentosus
Lactobacillus plantarum
Lactobacillus plantarum
Lactobacillus plantarum
Lactobacillus plantarum
Lactobacillus plantarum
Lactobacillus plantarum
Lactobacillus plantarum
Lactobacillus plantarum
Lactobacillus brevis
Lactobacillus plantarum
Lactobacillus plantarum
Lactobacillus plantarum
Lactobacillus plantarum
Lactobacillus plantarum
Lactobacillus plantarum
Lactobacillus brevis
Lactobacillus brevis
Unknown
Lactobacillus brevis
Lactobacillus brevis
Bacillus thuringiensis
Bacillus thuringiensis
Bacillus thuringiensis
Bacillus thuringiensis
Virgibacillus halotolerans
Bacillus amyloliquefaciens
Bacillus subtilis
Bacillus subtilis
Bacillus thuringiensis
Bacillus cereus
Unknown
Bacillus thuringiensis
Bacillus cereus
Unknown
Bacillus cereus
Bacillus cereus

Bacillus cereus
Bacillus thuringiensis
Bacillus thuringiensis
Bacillus thuringiensis
Bacillus thuringiensis
Bacillus cereus
Unknown
Unknown
Bacillus thuringiensis
Bacillus thuringiensis
Bacillus thuringiensis
Bacillus thuringiensis
Unknown
Bacillus thuringiensis
Bacillus thuringiensis
Bacillus cereus
Bacillus cereus
Bacillus cereus
Lactococcus lactis
Lactococcus lactis
Lactococcus lactis
Lactococcus lactis
Enterococcus faecium
Enterococcus faecalis
Brochothrix thermosphacta
Brochothrix thermosphacta
Enterococcus faecalis
Enterococcus faecium
Enterococcus faecium
Streptococcus ictaluri
Brochothrix thermosphacta
Enterococcus faecium
Enterococcus faecium
Staphylococcus pseudintermedius
Staphylococcus pseudintermedius
Staphylococcus aureus
Staphylococcus pseudintermedius
Staphylococcus pseudintermedius
Staphylococcus sciuri
Staphylococcus sciuri
Staphylococcus epidermidis
Staphylococcus epidermidis
Staphylococcus epidermidis
Bordetella bronchiseptica
Bordetella bronchiseptica
Bordetella bronchiseptica

Bordetella bronchiseptica
Bordetella bronchiseptica
Pseudomonas aeruginosa
Pseudomonas aeruginosa
Pseudomonas aeruginosa
Pseudomonas aeruginosa
Pseudomonas aeruginosa
Pseudomonas aeruginosa
Pseudomonas aeruginosa
Unknown
Yersinia enterocolitica
Bordetella bronchiseptica
Streptococcus anginosus
Streptococcus pyogenes
Streptococcus dysgalactiae
Streptococcus suis
Streptococcus suis
Streptococcus suis
Streptococcus suis
Streptococcus orisratti
Streptococcus suis
Streptococcus equi
Streptococcus urinalis
Streptococcus constellatus
Streptococcus agalactiae
Streptococcus anginosus
Streptococcus porcinus
Streptococcus agalactiae
Streptococcus urinalis
Streptococcus agalactiae
Streptococcus agalactiae
Microbacterium foliorum
Microbacterium foliorum
Microbacterium foliorum
Microbacterium foliorum
Microbacterium foliorum
Microbacterium foliorum
Microbacterium foliorum
Microbacterium foliorum
Microbacterium foliorum
Arthrobacter sp. ATCC 21022
Arthrobacter sp. ATCC 21022
Arthrobacter sp. ATCC 21022
Arthrobacter sp. ATCC 21022
Arthrobacter sp. ATCC 21022
Arthrobacter sp. ATCC 21022

Arthrobacter sp. ATCC 21022
Arthrobacter sp. ATCC 21022
Arthrobacter sp. ATCC 21022
Arthrobacter sp. ATCC 21022
Bacillus anthracis
Bacillus anthracis
Bacillus anthracis
Bacillus anthracis
Unknown
Bacillus anthracis
Bacillus thuringiensis
Bacillus cereus
Unknown
Bacillus pumilus
Bacillus pumilus
Bacillus pumilus
Bacillus pumilus
Bacillus pumilus
Bacillus pumilus
Bacillus pumilus
Bacillus pumilus
Acinetobacter baumannii
Acinetobacter baumannii
Acinetobacter baumannii
Acinetobacter baumannii
Pseudomonas aeruginosa
Pseudomonas aeruginosa
Unknown
Pseudomonas aeruginosa
Pseudomonas aeruginosa
Ralstonia solanacearum
Burkholderia cepacia
Unknown
Burkholderia cenocepacia
Burkholderia cenocepacia
Unknown
Unknown
Caulobacter vibrioides
Caulobacter vibrioides
Caulobacter vibrioides
Caulobacter vibrioides
Caulobacter vibrioides
Caulobacter vibrioides
Caulobacter vibrioides
Caulobacter vibrioides
Caulobacter vibrioides

Caulobacter vibrioides
Pectobacterium carotovorum
Pectobacterium carotovorum
Cronobacter sakazakii
Unknown
Aeromonas salmonicida
Aeromonas hydrophila
Aeromonas hydrophila
Aeromonas hydrophila
Salmonella enterica
Unknown
Yersinia enterocolitica
Unknown
Yersinia enterocolitica
Aeromonas hydrophila
Aeromonas hydrophila
Unknown
Vibrio diazotrophicus
Vibrio cyclitrophicus
Dickeya dadantii
Cronobacter sakazakii
Pasteurella multocida
Klebsiella pneumoniae
Klebsiella pneumoniae
Klebsiella pneumoniae
Enterobacter cloacae
Salmonella enterica
Salmonella enterica
Unknown
Salmonella enterica
Escherichia coli
Unknown
Escherichia sp.
Haemophilus influenzae
Haemophilus influenzae
Pasteurella multocida
Mannheimia haemolytica
Mannheimia haemolytica
Mannheimia haemolytica
Bacillus mycoides
Bacillus thuringiensis
Bacillus thuringiensis
Nodularia spumigena
Nodularia sp. (in: Bacteria)
Nodularia spumigena
Acidithiobacillus caldus

Unknown
Synechococcus sp. CB0204
Erwinia amylovora
Erwinia amylovora
Escherichia sp.
Escherichia coli
Unknown
Unknown
Rhizobium etli
Pseudomonas aeruginosa
Unknown
Xanthomonas citri
Burkholderia thailandensis
Synechococcus sp.
Synechococcus sp.
Unknown
Unknown
Unknown
Synechococcus sp. TCC793
Synechococcus sp. CB0202
Synechococcus sp.
Unknown
Synechococcus sp.
Synechococcus sp.
Synechococcus sp. CB0101
Achromobacter xylosoxidans
Achromobacter xylosoxidans
Achromobacter xylosoxidans
Pseudomonas syringae
Pseudomonas syringae
Pseudomonas syringae
Pseudomonas syringae
Pseudomonas syringae
Pseudomonas aeruginosa
Pseudomonas fluorescens
Synechococcus sp.
Unknown
Unknown
Unknown
Unknown
Unknown
Unknown
Staphylococcus aureus
Staphylococcus aureus
Staphylococcus pseudintermedius
Staphylococcus pseudintermedius

Staphylococcus pseudintermedius
Unknown
Staphylococcus pseudintermedius
Staphylococcus pseudintermedius
Staphylococcus pseudintermedius
Staphylococcus aureus
Staphylococcus epidermidis
Staphylococcus epidermidis
Staphylococcus epidermidis
Staphylococcus aureus
Staphylococcus aureus
Staphylococcus capitis
Streptomyces coelicolor
Streptomyces venezuelae
Streptomyces venezuelae
Streptomyces venezuelae
Unknown
Streptomyces griseus
Streptomyces xanthochromogenes
Streptomyces griseus
Streptomyces xanthochromogenes
Streptomyces lividans
Streptomyces xanthochromogenes
Streptomyces xanthochromogenes
Streptomyces venezuelae
Streptomyces venezuelae
Streptomyces viridochromogenes
Streptomyces coelicolor
Streptomyces sp.
Streptomyces platensis
Bacillus megaterium
Bacillus megaterium
Bacillus megaterium
Bacillus megaterium
Bacillus megaterium
Bacillus megaterium
Bacillus megaterium
Bacillus megaterium
Bacillus thuringiensis
Bacillus cereus
Bacillus thuringiensis
Bacillus cereus
Bacillus thuringiensis
Listeria monocytogenes
Listeria monocytogenes
Listeria sp.

Listeria monocytogenes
Unknown
Unknown
Listeria monocytogenes
Listeria monocytogenes
Bacillus thuringiensis
Bacillus cereus
Clostridioides difficile
Unknown
Clostridioides difficile
Unknown
Unknown
Unknown
Unknown
Clostridioides difficile
Clostridioides difficile
Unknown
Unknown
Unknown
Clostridioides difficile
Unknown
Unknown
Unknown
Unknown
Unknown
Unknown
Unknown
Unknown
Unknown
Pantoea sp.
Klebsiella pneumoniae
Klebsiella aerogenes
Klebsiella pneumoniae
Unknown
Escherichia coli
Cronobacter sakazakii
Stenotrophomonas maltophilia
Aeromonas salmonicida
Aeromonas sp.
Aeromonas salmonicida
Aeromonas salmonicida
Aeromonas salmonicida
Aeromonas salmonicida
Aeromonas salmonicida
Aeromonas salmonicida
Escherichia coli

Escherichia coli
Escherichia coli
Escherichia coli
Escherichia coli
Shigella sonnei
Escherichia coli
Cronobacter malonaticus
Acinetobacter baumannii
Acinetobacter johnsonii
Acinetobacter baumannii
Acinetobacter baumannii
Acinetobacter johnsonii
Acinetobacter johnsonii
Unknown
Acinetobacter baumannii
Acinetobacter baumannii
Acinetobacter baumannii
Acinetobacter baumannii
Acinetobacter baumannii
Acinetobacter baumannii
Acinetobacter johnsonii
Unknown
Acinetobacter baumannii
Acinetobacter baumannii
Unknown
Unknown
Acinetobacter baumannii
Acinetobacter baumannii
Sinorhizobium meliloti
Sinorhizobium meliloti
Rhizobium etli
Sinorhizobium sp. LM21
Acaryochloris marina
Acaryochloris marina
Ruegeria pomeroyi
Ruegeria pomeroyi
Unknown
Unknown
Unknown
Agrobacterium tumefaciens
Bacillus cereus
Bacillus thuringiensis
Bacillus thuringiensis
Unknown
Unknown
Bacillus thuringiensis

Bacillus thuringiensis
Unknown
Bacillus thuringiensis
Bacillus thuringiensis
Streptomyces platensis
Streptomyces platensis
Streptomyces xanthochromogenes
Streptomyces xanthochromogenes
Streptomyces xanthochromogenes
Streptomyces coelicolor
Streptomyces azureus
Streptomyces azureus
Streptomyces lividans
Streptomyces cattleya
Streptomyces platensis
Streptomyces mirabilis
Streptomyces griseus
Streptomyces xanthochromogenes
Streptomyces griseus
Streptomyces griseus
Streptomyces azureus
Streptomyces xanthochromogenes
Streptomyces lividans
Streptomyces lividans
Arthrobacter sp. ATCC 21022
Arthrobacter sp. ATCC 21022
Arthrobacter sp. ATCC 21022
Arthrobacter sp. ATCC 21022
Arthrobacter sp. ATCC 21022
Arthrobacter globiformis
Arthrobacter globiformis
Arthrobacter sp. ATCC 21022
Arthrobacter sp. ATCC 21022
Arthrobacter sp. ATCC 21022
Arthrobacter sp. ATCC 21022
Arthrobacter sp. ATCC 21022
Arthrobacter sp. ATCC 21022
Arthrobacter sp. ATCC 21022
Arthrobacter sp. ATCC 21022
Arthrobacter sp. ATCC 21022
Arthrobacter sp. ATCC 21022
Pseudomonas aeruginosa
Unknown
Pseudomonas aeruginosa
Pseudomonas aeruginosa
Unknown

Pseudomonas aeruginosa
Unknown
Pseudomonas aeruginosa
Unknown
Unknown
Burkholderia cenocepacia
Burkholderia thailandensis
Unknown
Unknown
Burkholderia cenocepacia
Ralstonia solanacearum
Mycolicibacterium smegmatis
Mycolicibacterium smegmatis
Mycolicibacterium smegmatis
Mycolicibacterium smegmatis
Mycolicibacterium smegmatis
Mycolicibacterium smegmatis
Mycolicibacterium smegmatis
Mycolicibacterium smegmatis
Gordonia terrae
Gordonia terrae
Gordonia terrae
Tsukamurella paurometabola
Rhodococcus erythropolis
Gordonia malaquae
Rhodococcus hoagii
Nocardia brasiliensis
Rhodococcus hoagii
Rhodococcus erythropolis
Rhodococcus erythropolis
Rhodococcus erythropolis
Gordonia neofelifaecis
[Brevibacterium] flavum
Rhodococcus hoagii
Rhodococcus hoagii
Gordonia malaquae
Gordonia terrae
Gordonia terrae
Gordonia terrae
Corynebacterium xerosis
Corynebacterium xerosis
Corynebacterium xerosis
Corynebacterium xerosis
Corynebacterium xerosis
Corynebacterium xerosis
Rhodococcus erythropolis

Gordonia terrae
Corynebacterium simulans
Corynebacterium xerosis
Corynebacterium vitaeruminis
Rhodococcus hoagii
Corynebacterium glutamicum
Vibrio vulnificus
Vibrio parahaemolyticus
Vibrio parahaemolyticus
Vibrio parahaemolyticus
Vibrio parahaemolyticus
Vibrio parahaemolyticus
Vibrio parahaemolyticus
Vibrio alginolyticus
Streptococcus parauberis
Streptococcus uberis
Streptococcus parauberis
Streptococcus parauberis
Streptococcus parauberis
Streptococcus parauberis
Streptococcus parauberis
Arthrobacter sp. ATCC 21022
Arthrobacter sp. ATCC 21022
Unknown
Arthrobacter sp. ATCC 21022
Arthrobacter sp.
Arthrobacter sp. ATCC 21022
Lactobacillus delbrueckii
Lactobacillus delbrueckii
Lactobacillus delbrueckii
Lactobacillus delbrueckii
Lactobacillus delbrueckii
Lactobacillus delbrueckii
Lactobacillus delbrueckii
Lactobacillus plantarum
Lactobacillus delbrueckii
Lactobacillus delbrueckii
Leuconostoc pseudomesenteroides
Lactobacillus delbrueckii
Lactobacillus delbrueckii
Lactobacillus delbrueckii
Lactobacillus delbrueckii
Lactobacillus delbrueckii
Lactobacillus delbrueckii
Lactobacillus delbrueckii
Unknown

Lactobacillus helveticus
Lactobacillus johnsonii
Lactobacillus johnsonii
Lactobacillus gasseri
Lactobacillus gasseri
Lactobacillus gasseri
Staphylococcus haemolyticus
Staphylococcus haemolyticus
Staphylococcus haemolyticus
Unknown
Staphylococcus epidermidis
Staphylococcus hominis
Staphylococcus aureus
Staphylococcus aureus
Staphylococcus aureus
Staphylococcus aureus
Staphylococcus aureus
Staphylococcus aureus
Staphylococcus warneri
Staphylococcus pseudintermedius
Gordonia malaquae
Gordonia terrae
Gordonia terrae
Gordonia terrae
Gordonia terrae
Gordonia terrae
Gordonia terrae
Gordonia terrae
Gordonia terrae
Gordonia terrae
Staphylococcus hyicus
Staphylococcus aureus
Staphylococcus aureus
Staphylococcus aureus
Staphylococcus aureus
Staphylococcus aureus
Staphylococcus sp.
Unknown
Unknown
Staphylococcus capitis
Unknown
Unknown
Staphylococcus epidermidis
Staphylococcus epidermidis
Staphylococcus epidermidis
Staphylococcus aureus

Unknown
Unknown
Unknown
Staphylococcus epidermidis
Staphylococcus aureus
Staphylococcus sp.
Staphylococcus aureus
Staphylococcus hyicus
Microbacterium foliorum
Microbacterium foliorum
Microbacterium foliorum
Microbacterium foliorum
Microbacterium foliorum
Microbacterium natoriense
Arthrobacter sp. ATCC 21022
Arthrobacter sp. ATCC 21022
Arthrobacter sp. ATCC 21022
Arthrobacter sp. ATCC 21022
Arthrobacter sp. ATCC 21022
Arthrobacter sp. ATCC 21022
Arthrobacter sp. ATCC 21022
Arthrobacter sp. ATCC 21022
Flavobacterium psychrophilum
Flavobacterium psychrophilum
Clostridium perfringens
Clostridium perfringens
Clostridium perfringens
Unknown
Unknown
Unknown
Bacteroides dorei
Croceibacter atlanticus
Bacillus cereus
Thermus thermophilus
Thermus scotoductus
Thermus scotoductus
Eggerthella lenta
Weissella cibaria
Leuconostoc pseudomesenteroides
Weissella sp.
Weissella ceti
Pseudomonas aeruginosa
Pseudomonas aeruginosa
Vibrio cyclitrophicus
Vibrio cyclitrophicus
Vibrio cyclitrophicus

Vibrio cyclitrophicus
Vibrio diazotrophicus
Synechococcus sp. CB0208
Pseudomonas aeruginosa
Unknown
Cronobacter sakazakii
Unknown
Streptomyces scabiei
Streptomyces griseus
Streptomyces griseus
Streptomyces griseus
Streptomyces griseus
Microbacterium paraoxydans
Microbacterium paraoxydans
Microbacterium paraoxydans
Microbacterium paraoxydans
Microbacterium foliorum
Microbacterium foliorum
Microbacterium foliorum
Microbacterium foliorum
Microbacterium foliorum
Microbacterium foliorum
Microbacterium foliorum
Microbacterium foliorum
Microbacterium foliorum
Streptomyces griseus
Streptomyces griseus
Streptomyces griseus
Streptomyces griseus
Streptomyces griseus
Streptomyces scabiei
Streptomyces scabiei
Streptomyces lividans
Streptomyces viridochromogenes
Streptomyces griseus
Streptomyces griseus
Streptomyces griseus
Streptomyces lividans
Streptomyces griseus
Streptomyces platensis
Mannheimia haemolytica
Unknown
Bordetella bronchiseptica
Pseudomonas putida
Pseudomonas lactis
Brucella abortus

Brucella sp.
Brucella canis
Pantoea agglomerans
Escherichia coli
Escherichia coli
Agrobacterium sp. H13-3
Agrobacterium tumefaciens
Candidatus Pelagibacter ubique
Dinoroseobacter shibae
Sulfitobacter sp. CB2047
Epibacterium mobile
Unknown
Nitratiruptor sp. SB155-2
Unknown
Vibrio sp.
Vibrio maritimus
Planktothrix agardhii
Unknown
Sinorhizobium meliloti
Ochrobactrum intermedium
Agrobacterium tumefaciens
Lactococcus lactis
Lactococcus lactis
Lactococcus lactis
Lactococcus lactis
Lactococcus lactis
Unknown
Faecalibacterium prausnitzii
Faecalibacterium prausnitzii
Aeromonas hydrophila
Aeromonas salmonicida
Aeromonas hydrophila
Vibrio parahaemolyticus
Vibrio cyclitrophicus
Vibrio parahaemolyticus
Vibrio parahaemolyticus
Unknown
Unknown
Unknown
Faecalibacterium prausnitzii
Faecalibacterium prausnitzii
Unknown
Marinomonas sp.
Achromobacter xylosoxidans
Achromobacter xylosoxidans
Achromobacter xylosoxidans

Achromobacter xylosoxidans
Pseudomonas aeruginosa
Unknown
Burkholderia pseudomallei
Unknown
Unknown
Bacteroides fragilis
Unknown
Curvibacter sp.
Caulobacter vibrioides
Candidatus Pelagibacter ubique
Unknown
Rhodobacter capsulatus
Rhodobacter capsulatus
Vibrio splendidus
Vibrio splendidus
Vibrio lentus
Vibrio cyclitrophicus
Pseudoalteromonas sp. YD6
Vibrio splendidus
Vibrio splendidus
Salinivibrio sp.
Vibrio tasmaniensis
Alteromonas gracilis
Microcystis elabens
Vibrio parahaemolyticus
Shewanella putrefaciens
Shewanella putrefaciens
Unknown
Unknown
Unknown
Streptococcus thermophilus
Streptococcus thermophilus
Streptococcus thermophilus
Streptococcus thermophilus
Unknown
Unknown
Streptococcus gordonii
Streptococcus parasanguinis
Streptococcus thermophilus
Streptococcus thermophilus
Streptococcus dysgalactiae
Streptococcus pyogenes
Streptococcus pyogenes
Staphylococcus aureus
Streptococcus suis

Streptococcus azizii
Streptococcus pyogenes
Streptococcus dysgalactiae
Streptococcus dysgalactiae
Lactococcus lactis
Unknown
Arthrobacter globiformis
Arthrobacter globiformis
Arthrobacter globiformis
Arthrobacter globiformis
Arthrobacter globiformis
Streptococcus thoraltensis
Enterococcus faecium
Enterococcus faecalis
Enterococcus faecalis
Bacillus cereus
Bacillus cereus
Bacillus cereus
Bacillus cohnii
Unknown
Bacillus halmapalus
Geobacillus kaustophilus
Bacillus pumilus
Bacillus subtilis
Listeria monocytogenes
Listeria monocytogenes
Listeria monocytogenes
Listeria monocytogenes
Listeria monocytogenes
Unknown
Unknown
Unknown
Unknown
Achromobacter xylosoxidans
Pseudomonas aeruginosa
Pseudomonas aeruginosa
Unknown
Pseudomonas aeruginosa
Vibrio harveyi
Vibrio parahaemolyticus
Vibrio alginolyticus
Vibrio alginolyticus
Pseudomonas aeruginosa
Erwinia amylovora
Erwinia amylovora
Thermus sp. TC4

Thermus thermophilus
Thermus thermophilus
Thermus thermophilus
Pseudomonas aeruginosa
Pseudomonas aeruginosa
Pseudomonas aeruginosa
Streptomyces griseus
Streptomyces griseus
Streptomyces griseus
Streptomyces griseus
Streptomyces lividans
Streptomyces griseus
Streptomyces griseus
Streptomyces griseus
Streptomyces scabiei
Streptomyces scabiei
Streptomyces sp.
Streptomyces lividans
Vibrio natriegens
Vibrio alginolyticus
Vibrio alginolyticus
Vibrio parahaemolyticus
Vibrio parahaemolyticus
Vibrio lentus
Enterovibrio norvegicus
Enterovibrio norvegicus
Shewanella sp. 4
Aeromonas salmonicida
Aeromonas salmonicida
Erwinia amylovora
Aeromonas sp.
Unknown
Unknown
Unknown
Unknown
Unknown
Unknown
Unknown
Unknown
Unknown
Unknown
Unknown
Unknown
Unknown
Unknown
Unknown

Unknown
Listeria monocytogenes
Listeria monocytogenes
Listeria monocytogenes
Bacillus anthracis
Unknown
Bacillus subtilis
Unknown
Bacillus megaterium
Bacillus megaterium
Bacillus megaterium
Listeria monocytogenes
Listeria monocytogenes
Bacillus paralicheniformis
Bacillus pseudalcaliphilus
Bacillus subtilis
Unknown
Thermus thermophilus
Flavobacterium sp.
Vibrio cholerae
Vibrio cholerae
Vibrio cholerae
Cellulophaga baltica
Cellulophaga baltica
Cellulophaga baltica
Gordonia sp.
Gordonia sp.
Gordonia malaquae
Gordonia terrae
Sulfitobacter sp. EE-36
Ruegeria pomeroyi
Ruegeria pomeroyi
Roseobacter denitrificans
Ruegeria pomeroyi
Paracoccus thiocyanatus
Paracoccus kondratievae
Sinorhizobium meliloti
Unknown
Streptomyces azureus
Streptomyces venezuelae
Streptomyces venezuelae
Streptomyces coelicolor
Streptomyces coelicolor
Unknown
Streptomyces griseus
Streptomyces griseus

Streptomyces griseus
Streptomyces lividans
Streptomyces griseus
Stenotrophomonas maltophilia
Pseudomonas aeruginosa
Pseudomonas aeruginosa
Arthrobacter sp. ATCC 21022
Arthrobacter sp. ATCC 21022
Arthrobacter sp. ATCC 21022
Streptococcus porcinus
Streptococcus agalactiae
Brevibacillus laterosporus
Brevibacillus laterosporus
Brevibacillus laterosporus
Unknown
Flavobacterium sp. B183
Faecalibacterium prausnitzii
Bacillus megaterium
Bacillus megaterium
Unknown
Unknown
Unknown
Exiguobacterium aurantiacum
Unknown
Unknown
Listeria weihenstephanensis
Unknown
Bacillus pseudalcaliphilus
Unknown
Anoxybacillus caldiproteolyticus
Paenibacillus larvae
Paenibacillus larvae
Paenibacillus larvae
Vibrio cholerae
Vibrio cholerae
Unknown
Exiguobacterium alkaliphilum
Exiguobacterium aurantiacum
Bacillus subtilis
Bacillus subtilis
Bacillus megaterium
Unknown
Geobacillus sp. E263
Caldibacillus debilis
Bacillus sp. (in: Bacteria)
Bacillus alcalophilus

Bacillus cereus
Bacillus thuringiensis
Bacillus cereus
Bacillus cereus
Bacillus thuringiensis
Unknown
Bacillus cereus
Bacillus cereus
Bacillus thuringiensis
Bacillus anthracis
Unknown
Clostridioides difficile
Unknown
Clostridioides difficile
Clostridium sporogenes
Clostridium saccharoperbutylacetonicum
Clostridium perfringens
Clostridium perfringens
Clostridium perfringens
Clostridium perfringens
Clostridium perfringens
Unknown
Vibrio cholerae
Vibrio cholerae
Vibrio lentus
Vibrio splendidus
Vibrio metschnikovii
Shewanella inventionis
Riemerella anatipestifer
Halomonas ventosae
Unknown
Acinetobacter baumannii
Unknown
Acinetobacter johnsonii
Acinetobacter johnsonii
Microbacterium foliorum
Microbacterium foliorum
Microbacterium foliorum
Microbacterium foliorum
Microbacterium foliorum
Microbacterium foliorum
Microbacterium foliorum
Microbacterium foliorum
Microbacterium paraoxydans
Microbacterium foliorum
Streptomyces sp.

Rhodococcus erythropolis
Rhodococcus erythropolis
Rhodococcus erythropolis
Rhodococcus erythropolis
Unknown
Synechococcus sp. CB0101
Synechococcus sp. CB0101
Synechococcus sp. CB0101
Prochlorococcus marinus
Synechococcus sp. CB0201
Synechococcus sp.
Pseudoalteromonas sp.
Vibrio alginolyticus
Vibrio parahaemolyticus
Vibrio sp.
Streptococcus thermophilus
Lactobacillus delbrueckii
Microbacterium paraoxydans
Microbacterium paraoxydans
Microbacterium foliorum
Microbacterium foliorum
Microbacterium paraoxydans
Microbacterium foliorum
Microbacterium foliorum
Streptomyces scabiei
Streptomyces scabiei
Streptococcus porci
Streptococcus constellatus
Streptococcus equi
Faecalibacterium prausnitzii
Unknown
Unknown
Unknown
Unknown
Unknown
Arthrobacter sp. ATCC 21022
Propionibacterium freudenreichii
Propionibacterium freudenreichii
Propionibacterium freudenreichii
Corynebacterium pseudotuberculosis
Pasteurella multocida
Rhodococcus erythropolis
Gordonia malaquae
Gordonia rubripertincta
Streptococcus uberis
Erysipelothrix rhusiopathiae

Lactobacillus fermentum
Lactobacillus gasseri
Lactobacillus johnsonii
Propionibacterium freudenreichii
Propionibacterium freudenreichii
Propionibacterium freudenreichii
Microbacterium foliorum
Microbacterium foliorum
Thermoanaerobacterium saccharolyticum
Streptococcus constellatus
Bacillus pumilus
Clostridium perfringens
Clostridium perfringens
Lactobacillus brevis
Lactobacillus brevis
Lactobacillus brevis
Lactobacillus jensenii
Arthrobacter globiformis
Enterococcus faecalis
Lactobacillus plantarum
Faecalibacterium prausnitzii
Clostridium tyrobutyricum
Paenibacillus polymyxa
Geobacillus stearothermophilus
Unknown
Unknown
Unknown
Microbacterium foliorum
Microbacterium foliorum
Microbacterium paraoxydans
Microbacterium foliorum
Microbacterium paraoxydans
Microbacterium foliorum
Microbacterium paraoxydans
Arthrobacter globiformis
Arthrobacter globiformis
Arthrobacter globiformis
Arthrobacter globiformis
Microcystis aeruginosa
Bacillus pumilus
Bacillus thuringiensis
Unknown
Planktothrix agardhii
Pontimonas salivibrio
Rhodococcus hoagii
Paeniglutamicibacter sulfureus

Paeniglutamicibacter sulfureus
Arthrobacter sp. ATCC 21022
Arthrobacter globiformis
Arthrobacter globiformis
Sinomonas atrocyanea
Arthrobacter globiformis
Arthrobacter globiformis
Arthrobacter globiformis
Arthrobacter sp.
Arthrobacter globiformis
Microbacterium paraoxydans
Microbacterium oxydans
Brevibacterium fuscum
Xanthomonas citri
Thermus thermophilus
Meiothermus sp.
Thermus thermophilus
Lactobacillus plantarum
Lactobacillus plantarum
Lactobacillus delbrueckii
Lactobacillus delbrueckii
Weissella ceti
Lactobacillus brevis
Rhizobium etli
Rhizobium etli
Erwinia amylovora
Cronobacter sakazakii
Erwinia amylovora
Erwinia amylovora
Erwinia amylovora
Erwinia amylovora
Vibrio alginolyticus
Erwinia amylovora
Serratia marcescens
Serratia marcescens
Microbacterium foliorum
Thermobifida fusca
Thermobifida fusca
Bacillus sp. (in: Bacteria)
Bacillus bogoriensis
Bacillus megaterium
Exiguobacterium alkaliphilum
Pelagibaca abyssi
Sphingobium sp. IP1
Ruegeria pomeroyi
Ruegeria pomeroyi

Paracoccus marcusii
Roseobacter denitrificans
Roseobacter denitrificans
Vibrio sp. F13
Unknown
Vibrio sp. YD21
Vibrio maritimus
Alteromonas sp.
Vibrio cyclitrophicus
Pseudomonas syringae
Flavobacterium psychrophilum
Pseudomonas syringae
Unknown
Arthrobacter globiformis
Arthrobacter globiformis
Arthrobacter sp. ATCC 21022
Arthrobacter sp. ATCC 21022
Bacillus pseudofirmus
Bacillus cereus
Unknown
Brevibacillus laterosporus
Arthrobacter globiformis
Unknown
Unknown
Synechococcus sp.
Synechococcus sp.
Unknown
Streptomyces lividans
Streptomyces lividans
Streptomyces sp.
Unknown
Unknown
Unknown
Unknown
Unknown
Synechococcus sp.
Synechococcus sp. CB0101
Lactococcus garvieae
Unknown
Croceibacter atlanticus
Prochlorococcus marinus
Synechococcus sp. CB0204
Synechococcus sp. WH 7803
Unknown
Brevibacterium iodinum
Clostridioides difficile

Streptomyces lividans
Dinoroseobacter shibae
Unknown
Pseudomonas syringae
Pseudomonas aeruginosa
Clavibacter michiganensis
Xylella fastidiosa
Pseudomonas syringae
Pseudomonas syringae
Unknown
Bifidobacterium thermophilum
Yersinia enterocolitica
Thermus thermophilus
Thermus thermophilus
Lactobacillus rhamnosus
Staphylococcus epidermidis
Unknown
Unknown
Unknown
Unknown
Xanthomonas euvesicatoria
Tetrasphaera jenkinsii
Citromicrobium sp. RCC1878
Aerococcus viridans
Bacillus cereus
Unknown
Faecalibacterium prausnitzii
Unknown
Unknown
Unknown
Faecalibacterium prausnitzii
Unknown
Unknown
Bacillus sp. (in: Bacteria)
Unknown
Rathayibacter toxicus
Arthrobacter sp. ATCC 21022
Enterococcus faecalis
Streptococcus sp. 'group C'
Enterococcus faecalis
