# Supplementary material for: PhaLP: A Database for the Study of Phage Lytic Proteins and Their Evolution
Source: Viruses. 2021 Jun 26;13(7):1240. doi: 10.3390/v13071240 (PMC8310338; doi:10.3390/v13071240)
Supplement: Supplementary file 1 [file viruses-13-01240-s001.zip › viruses-1263339-supplementary.pdf]

viruses

MDPI

Supplementary material

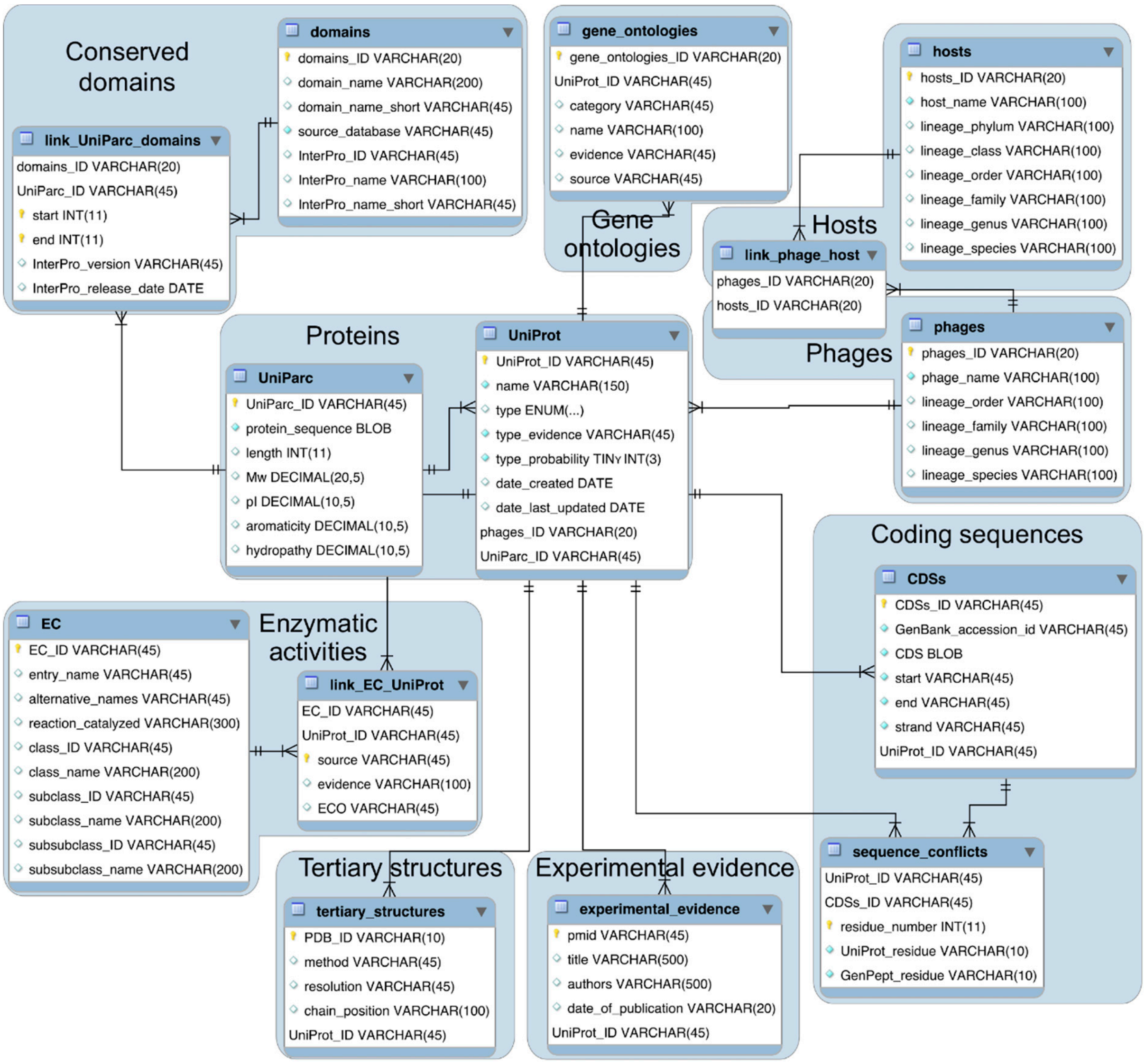

S1 Fig. Enhanced entity-relationship (EER) diagram of the MySQL-based PhaLP database. Each table is represented by a box containing a list of its respective column names and their corresponding data type, with the maximum length between brackets. Columns that are (part of) the primary key of a table are indicated with a key symbol in front of the column name. Foreign keys used to link to another table have no symbol. Other columns are indicated with blue diamonds (filled if the column is obligatory, empty if optional). Relationships between tables are indicated with a crow's foot notation. A relationship is indicated by a line with a double perpendicular line at the side of a 'one' table and a crow's foot at the side of a 'many' table. The 'one-to-many' relationship between 'phages' and 'UniProt' for example can be interpreted as: one phage can be linked to many UniProt entries, but each UniProt entry can only be linked to

one phage. The nine data types are illustrated on the EER diagram as blue boxes and group the tables that contribute to each data type.

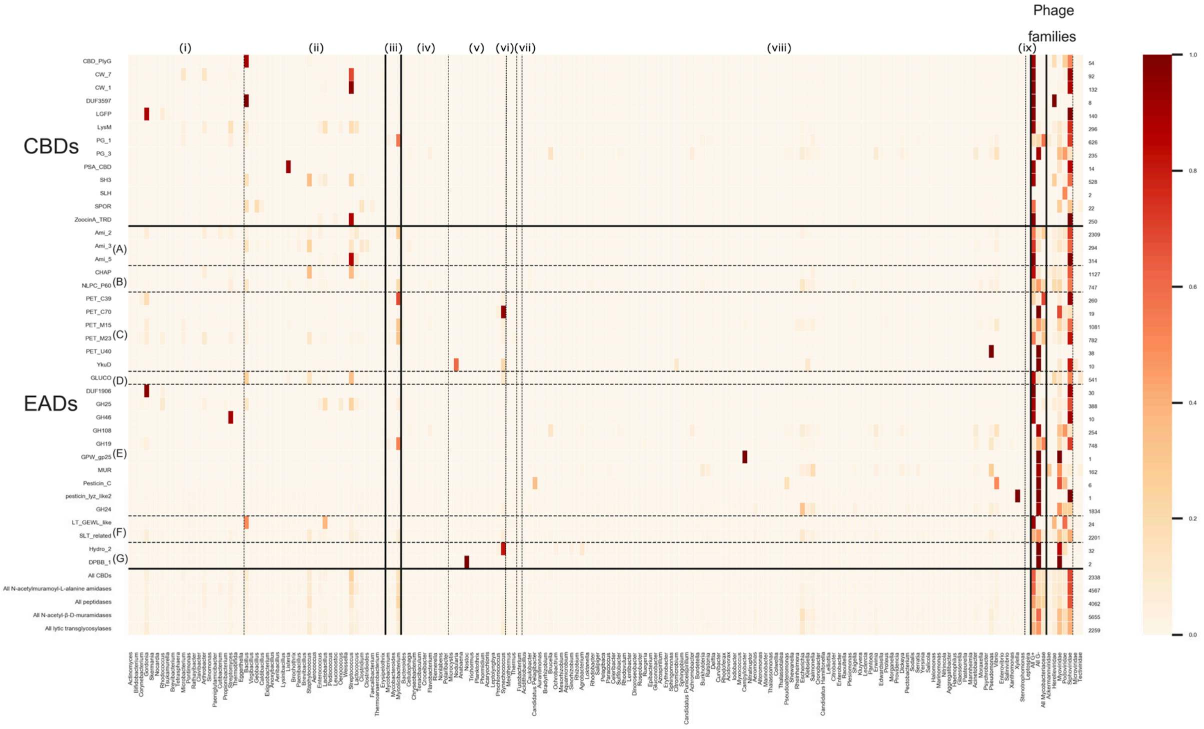

S2 Fig. Distribution of bacterial genera across EADs and CBDs. The color bar on the right denotes the probability that a phage lytic protein is associated with a specific host, given that it contains a certain domain (dark red = 1; light yellow = 0). The examined phyla from left to right, separated by dashed and full lines, are: (i) Actinobacteria without Mycobacteriaceae (family), (ii) Firmicutes, (iii) Mycobacteriaceae (family), (iv) Bacteroidetes, (v) Cyanobacteria, (vi) Deinococcus-Thermus, (vii) Fusobacteria, (viii) Proteobacteria and (ix) Spirochaetes. The enzymatic domains from top to bottom, separated by dashed lines, are: (A) N-acetylmuramoyl-L-alanine amidases, (B) domains with mixed N-acetylmuramoyl-L-alanine amidases and peptidase activity, (C) peptidase domains, (D) N-acetyl-β--D-glucosaminidase domains, (E) N-acetyl-β-D-muramidase domains, (F) domains with N-acetyl-β-D-muramidase and lytic transglycosylase activity and (G) lytic transglycosylase domains. On the bottom, probabilities are visualized grouped given the domain type. On the right, the overall probability of a given Gram-type as well as phage family are set out for each domain.

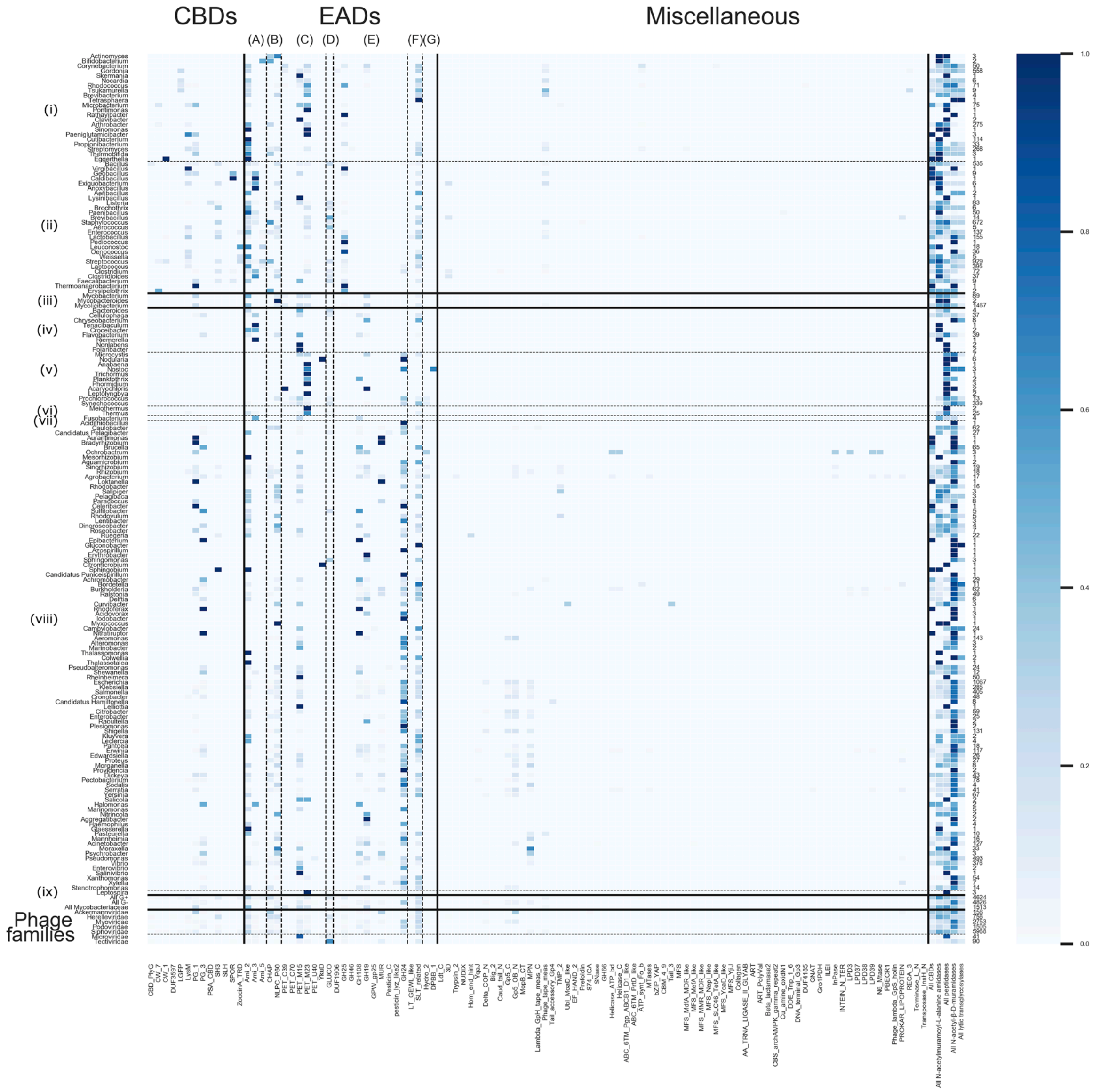

S3 Fig. Distribution of all domains across bacterial genera. The color bar on the right denotes the probability that a domain occurs for a phage lytic protein given its bacterial host (dark blue = 1; white = 0). The examined host phyla from top to bottom, separated by dashed and full lines, are: (i) Actinobacteria without Mycobacteriaceae (family), (ii) Firmicutes, (iii) Mycobacteriaceae (family), (iv) Bacteroidetes, (v) Cyanobacteria, (vi) Deinococcus-Thermus, (vii) Fusobacteria, (viii) Proteobacteria and (ix) Spirochaetes. The enzymatic domains from left to right, separated by dashed lines, are: (A) N-acetylmuramoyl-L-alanine amidases, (B) domains with mixed N-acetylmuramoyl-L-alanine amidases and peptidase activity, (C) peptidase domains, (D) N-acetyl-β-D-glucosaminidase domains, (E) N-acetyl-β-D-muramidase domains, (F) domains with N-acetyl-β-D-muramidase and lytic transglycosylase activity and (G) lytic transglycosylase domains. On the bottom, probabilities are visualized grouped given the host Gram-types as well as

the phage families and on the right, the overall probability of domains of a given activity are set out for each bacterial host.

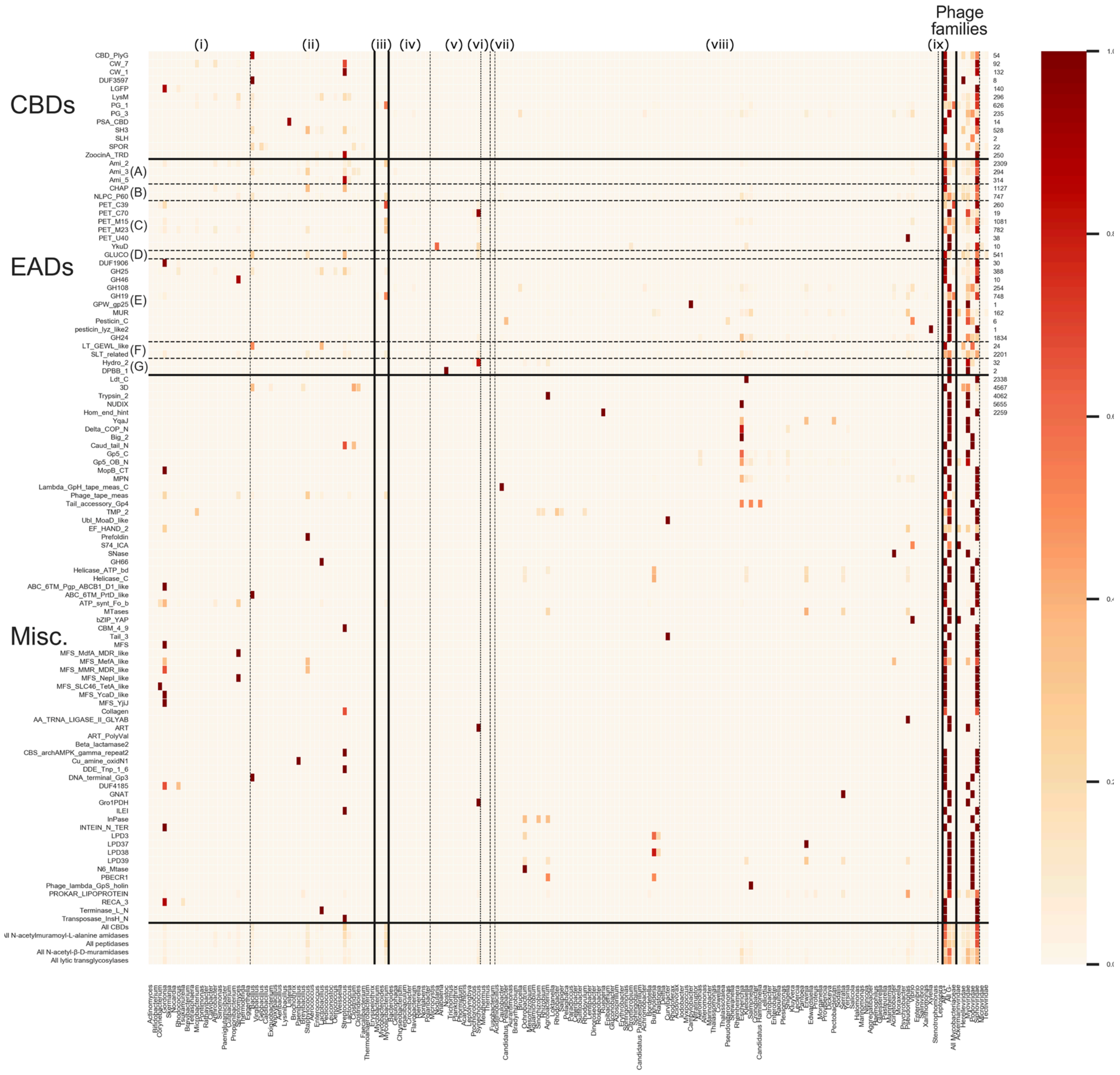

S4 Fig. Distribution of bacterial genera across all domains. The color bar on the right denotes the probability that a phage lytic protein is associated with a specific host, given that it contains a certain domain (dark red = 1; light yellow = 0). The examined phyla from left to right, separated by dashed and full lines, are: (i) Actinobacteria without Mycobacteriaceae (family), (ii) Firmicutes, (iii) Mycobacteriaceae (family), (iv) Bacteroidetes, (v) Cyanobacteria, (vi) Deinococcus-Thermus, (vii) Fusobacteria, (viii) Proteobacteria and (ix) Spirochaetes. The enzymatic domains from top to bottom, separated by dashed lines, are: (A) N-acetylmuramoyl-L-alanine amidases, (B) domains with mixed N-acetylmuramoyl-L-alanine amidases and peptidase activity, (C) peptidase domains, (D) N-acetyl-β-D-glucosaminidase domains, (E) N-acetyl-β-D-muramidase domains, (F) domains with N-acetyl-β-D-muramidase and lytic

transglycosylase activity and (G) lytic transglycosylase domains. On the bottom, probabilities are visualized grouped given the domain type. On the right, the overall probability of a given Gram-type as well as phage family are set out for each domain.

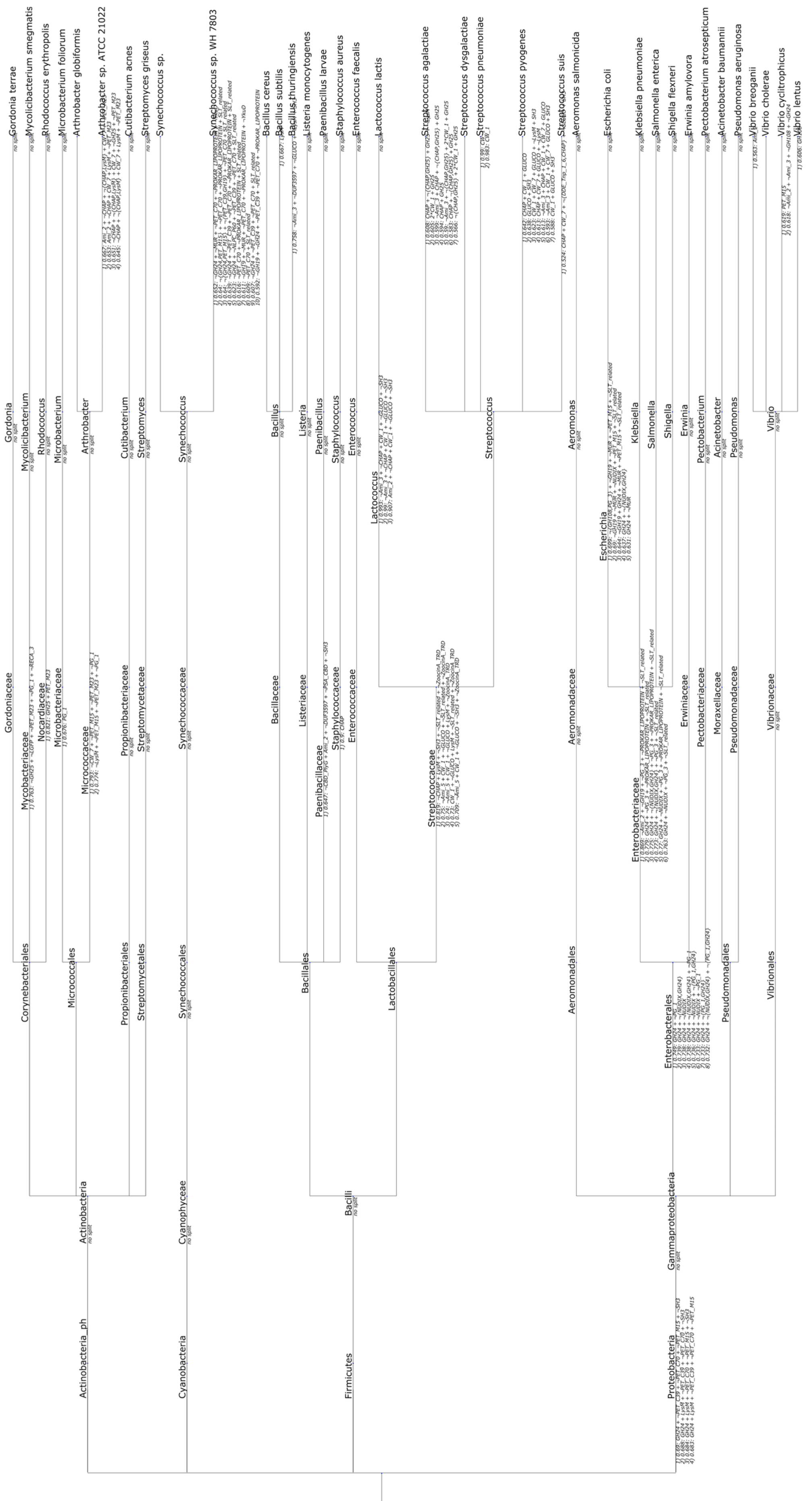

Gordonia terrae
Mycolicibacterium smegmatis
Rhodococcus erythropolis
Microbacterium foliorum
Arthrobacter globiformis
Arthrobacter sp. ATCC 21022
Cutibacterium acnes
Streptomyces griseus
Synechococcus sp.
Synechococcus sp. WH 7803
Bacillus cereus
Bacillus subtilis
Bacillus thuringiensis
Listeria monocytogenes
Paenibacillus larvae
Staphylococcus aureus
Enterococcus faecalis
Lactococcus lactis
Streptococcus agalactiae
Streptococcus dysgalactiae
Streptococcus pneumoniae
Streptococcus pyogenes
Streptococcus suis
Aeromonas salmonicida
Escherichia coli
Klebsiella pneumoniae
Salmonella enterica
Shigella flexneri
Erwinia amylovora
Pectobacterium atrosepticum
Acinetobacter baumannii
Pseudomonas aeruginosa
Vibrio breoganii
Vibrio cholerae
Vibrio cyclitrophicus
Vibrio lentus
Gordonia
Mycolicibacterium
Rhodococcus
Microbacterium
Arthrobacter
Cutibacterium
Streptomyces
Synechococcus
Bacillus
Listeria
Paenibacillus
Staphylococcus
Enterococcus
Lactococcus
Streptococcus
Aeromonas
Escherichia
Klebsiella
Salmonella
Shigella
Erwinia
Pectobacterium
Acinetobacter
Pseudomonas
Vibrio
Gordoniaceae
Mycobacteriaceae
Nocardiaceae
Microbacteriaceae
Micrococcaceae
Propionibacteriaceae
Streptomycetaceae
Synechococcaceae
Bacillaceae
Listeriaceae
Paenibacillaceae
Staphylococcaceae
Enterococcaceae
Streptococcaceae
Aeromonadaceae
Enterobacteriaceae
Erwiniaceae
Pectobacteriaceae
Moraxellaceae
Pseudomonadaceae
Vibrionaceae
Corynebacteriales
Micrococcales
Propionibacteriales
Streptomycetales
Synechococcales
Bacillales
Lactobacillales
Aeromonadales
Enterobacterales
Pseudomonadales
Vibrionales
Actinobacteria
Cyanophyceae
Bacilli
Gammaproteobacteria
Actinobacteria_ph
Cyanobacteria
Firmicutes
Proteobacteria

S5 Fig. Decision rules for phage lytic proteins targeting bacteria from all clades that have more than 25 related proteins in PhaLP as predicted by SkopeRules. For each branch, a set of decision rules is set out describing the domains necessary in a protein for the SkopeRules machine learning model to predict it as belonging to that branch. The '+' describes domains that should be present regardless of order, while subsequent domains in the architecture are grouped between curly brackets. When a domain should be absent instead of present, this is denoted by a negation sign '¬'. Each rule is preceded by its resulting F-score. Only rules with precision and recall greater than or equal to 0.5 are printed.

S1 File. Pairwise comparison of overlapping domain profiles. The excel file shows a pairwise comparison of each pair of domain profiles. Left (columns E to HD): the average overlap between each pair was calculated as the fraction between the overlapping section and the largest domain profile. Click on the cell to see all digits. Right (columns HG to PF): the absolute number of overlaps between each pair of domain profiles is also displayed. The cells are colored according to a scale. Left: 1 = white, 0.5 is red, 0 is black. Right: maximum = white, 1 is red, 0 is black.

S2 File. Design tree for phage lytic proteins targeting bacteria from all clades that have more than 25 related proteins in PhaLP. Per position, square brackets contain different domains that can occur at that position. To simplify the designs, CBD homorepeats were condensed to a single occurrence of the domain. To accommodate for architectures of one up to three domains, the subscript 'x0-1' has been added to indicate domains that either do not occur or occur once. The spreadsheet also provides the F-score as a measure of how many of the actual architectures fit the rule, as well the support, signifying the total amount of proteins corresponding to this branch.

S3 File. Cluster analysis of the pairwise protein sequence similarity between endolysins. The first tab contains a navigable heatmap with normalized similarity scores as illustrated in Fig 8. The next 45 tabs contain annotations on architecture, accession and host of each protein in the respective cluster. Cluster 45 is composed of all remaining clusters.
